# Genomic and immunological profiles of small-cell lung cancer between East Asians and Caucasian

**DOI:** 10.1186/s12935-022-02588-w

**Published:** 2022-04-29

**Authors:** Anqi Lin, Ningning Zhou, Weiliang Zhu, Jiexia Zhang, Ting Wei, Linlang Guo, Peng Luo, Jian Zhang

**Affiliations:** 1 Department of Oncology, Zhujiang Hospital, Southern Medical University, 253 Industrial Avenue, Guangzhou, 510282 Guangdong China; 2 Department of Medical Oncology, State Key Laboratory of Oncology in South China, Collaborative Innovation Center for Cancer Medicine, Sun Yat-Sen University Cancer Center, Guangzhou, China; 3 Department of Medicine, National Clinical Research Center for Respiratory Disease, State Key Laboratory of Respiratory Disease, Guangzhou Institute of Respiratory Disease, Guangzhou, China; 4 Department of Pathology, Zhujiang Hospital, Southern Medical University, Guangzhou, Guangdong China

**Keywords:** SCLC, East Asian, Caucasian, Genomic, Immune-infiltrating

## Abstract

The characterization of immunological and genomic differences in small-cell lung cancer (SCLC) between East Asian (EA) and Caucasian patients can reveal important clinical therapies for EA patients with SCLC. By sequencing and analyzing a molecular and immunological dataset of 98-SCLC patients of EA ancestry, immunogenicity, including DNA damage repair alterations and tumor mutation burden (TMB), was found to be significantly higher in the EA cohort than in the Caucasian cohort. The epithelial-mesenchymal transition (EMT) was the signaling signature with the predominant frequency of mutations across all patients in the EA cohort. Analysis of tumor-infiltrated immune cells revealed that resting lymphocytes were significantly enriched in the EA cohort. Compound-targeting analysis showed that topoisomerase inhibitors might be capable of targeting *TP53* and *RB1* comutations in EA SCLC patients. EA SCLC patients who harbored *COL6A6* mutations had poor survival, while Caucasian SCLC patients with *OTOF*, *ANKRD30B*, and *TECPR2* mutations were identified to have a shorter survival.

**Supplementary Information:**

The online version contains supplementary material available at 10.1186/s12935-022-02588-w.

## Introduction

Small-cell lung cancer (SCLC) accounts for 13 ~ 20% of lung cancers and is characterized by rapid growth, expression of neuroendocrine markers, early spread and secondary therapeutic resistance [1–4]. Approximately one-third of patients diagnosed with early-stage disease are commonly cured with standard chemotherapy or radiotherapy, while the majority of patients have only a few treatment options, such as palliative care [3]. The 5-year overall survival (OS) rate for SCLC is extremely low (5–10%) [5]. Several studies have characterized the genomic profile of SCLC and discovered therapeutic implications and new candidate alterations, such as *BRAF*, *KIT* and *PI3K/AKT/mTOR* [1, 5–8]. Additionally, *NOTCH* family genes, acting as tumor suppressors, are capable of regulating neuroendocrine differentiation involving tumor pathogenesis [1]. Therefore, understanding the key biological signaling pathways may stratify vulnerabilities and define new therapeutic targets. Additionally, previous studies indicated that the majority of SCLC harbors *RB1/TP53* co-mutations, suggesting that inactivation of *RB1* and *TP53* is a prerequisite in SCLC [1, 9]. Thus, further analysis of *RB1/TP53* co-mutations has critical value for characterizing biological features and designing optional treatments.

Recently, immunotherapy, particularly immune checkpoint inhibitors (ICIs), has been incorporated in first-line treatment for SCLC and substantially improves the median survival of SCLC [10]. In addition, ICI efficacy was associated with high tumor immunogenicity, inflammatory expression profiles and immune checkpoint expression [11].

To date, genomic studies of SCLC have focused on a single ancestry (East Asian (EA) or Caucasian patients) [1, 4]. However, SCLC may differ substantially among EA and Caucasian individuals in terms of the genomic characteristics, tumor microenvironment (TME), and critical biological pathways.

To better understand the ancestry disparities among the EA and Caucasian populations and to portray a comprehensive genomic and immunological profile of EA SCLC patients, we sequenced the transcriptomes (n = 59) and whole exomes (n = 98) of 98 EA SCLC patients from China. To discover new genomic targets enriched in the EA cohort, we compared our data to published whole-exome sequencing (WES) data of a Caucasian cohort consisting of 45 patients (reported by George et al.) [1] by analyzing the clinical, immunological and genomic features.

## Methods

### Sample collection, gene sequencing and public dataset processing

The Institutional Review Boards (IRBs) of the First Affiliated Hospital of Guangzhou Medical University, the Sun Yat-sen University Cancer Center, and the Zhujiang Hospital of Southern Medical University approved this study. A total of 98 samples from EA SCLC patients were provided by these hospitals under IRB-approved protocols with informed consent. These 98 tumor samples from EA SCLC patients were collected retrospectively from surgical material. We used blood or adjacent normal tissues as a matched control. TNM stage, sex, smoking history, and age were collected. Detailed information is provided in the Additional file: Supplemental methods 16.

Processed WES data and RNA sequencing (RNA-seq) files of 45 Caucasian SCLC patients from a 2015 Nature study were downloaded from cBioPortal (https://www.cbioportal.org/study/summary?id=sclc_ucologne_2015) [1]. The SCLC cell lines described in this study were derived from the Genomics of Drug Sensitivity in Cancer (GDSC) database [12] and had drug sensitivity and WES data.

### Immune profiling analysis

The CIBERSORT algorithm [13] was supplied with mRNA data of EA and Caucasian SCLC patients. The proportion of twenty-two tumor-infiltrating immune cells was used in downstream analysis. Then, expression values were selected for CIBERSORT analysis using default parameters (perm = 1,000; QN = F). Marker genes [14, 15] related to immune cells, antigen presentation, cytotoxicity, cytokines, and immune checkpoints were collected from previous studies and used to evaluate the immune signatures of SCLC. To compare differentially immune cells and immune-related genes, a linear model of the limma package was supplied.

### Mutational landscape and DDR-related analysis

The Complexheatmap package [16] was used to visualize the waterfall plot of mutations in EA and Caucasian SCLC patients. Nonsynonymous mutation types were determined using the maftools package [17]. The summary plot of the MAF files and figures of somatic interactions were generated by the maftools package. Tumor mutation burden (TMB) values were calculated according to a previous study [18]. A list of hallmark and DNA damage response (DDR) genes was collected from the Molecular Signatures Database (https://www.gsea-msigdb.org/gsea/msigdb/index.jsp) [19] and used for signaling alteration analysis. The DDR signature scores were calculated using the gene set variation analysis (GSVA) package [20] with the single-sample gene set enrichment analysis (ssGSEA) method. According to the median age or TMB value, SCLC patients were classified into groups: older vs young and high TMB vs low TMB. Driver gene annotations were downloaded from the Network of Cancer Genes (NCG) database [21].

### Compound-targeting analysis

To identify which inhibitors/compounds may be useful for targeting cells with *TP53* and *RB1* co-mutations, we applied the Broad Institute’s Connectivity Map (CMap) build 02 [22], which is a public online analytical tool (https://portals.broadinstitute.org/cmap/) that allows the analyzer to predict potential inhibitors/compounds based on upregulated and downregulated genes in a gene expression signature.

To further discover the mechanism of action (MoA) [23] and inhibitors/compounds, we analyzed them using CMap tools (https://clue.io/). The CMap method is similar to the gene set enrichment analysis (GSEA) algorithm, which can identify similarities and connectivities (range: − 1 to 1) based on differential gene expression data.

### Statistical analysis

All analyses were performed in R (version 3.6.1). The Mann–Whitney U test was used for the comparison of two continuous variables. Fisher’s exact test was supplied with two categorical variables. P values were controlled for false discovery rate (FDR), and an FDR less than 0.05 was considered statistically significant; all statistical tests were two-sided. Survival analysis was performed using the Kaplan–Meier method, and the log-rank test p-value was calculated. A Cox regression model was used in univariable analyses.

## Results

### Genomic profile of the EA and Caucasian SCLC cohorts

**Fig. 1:**
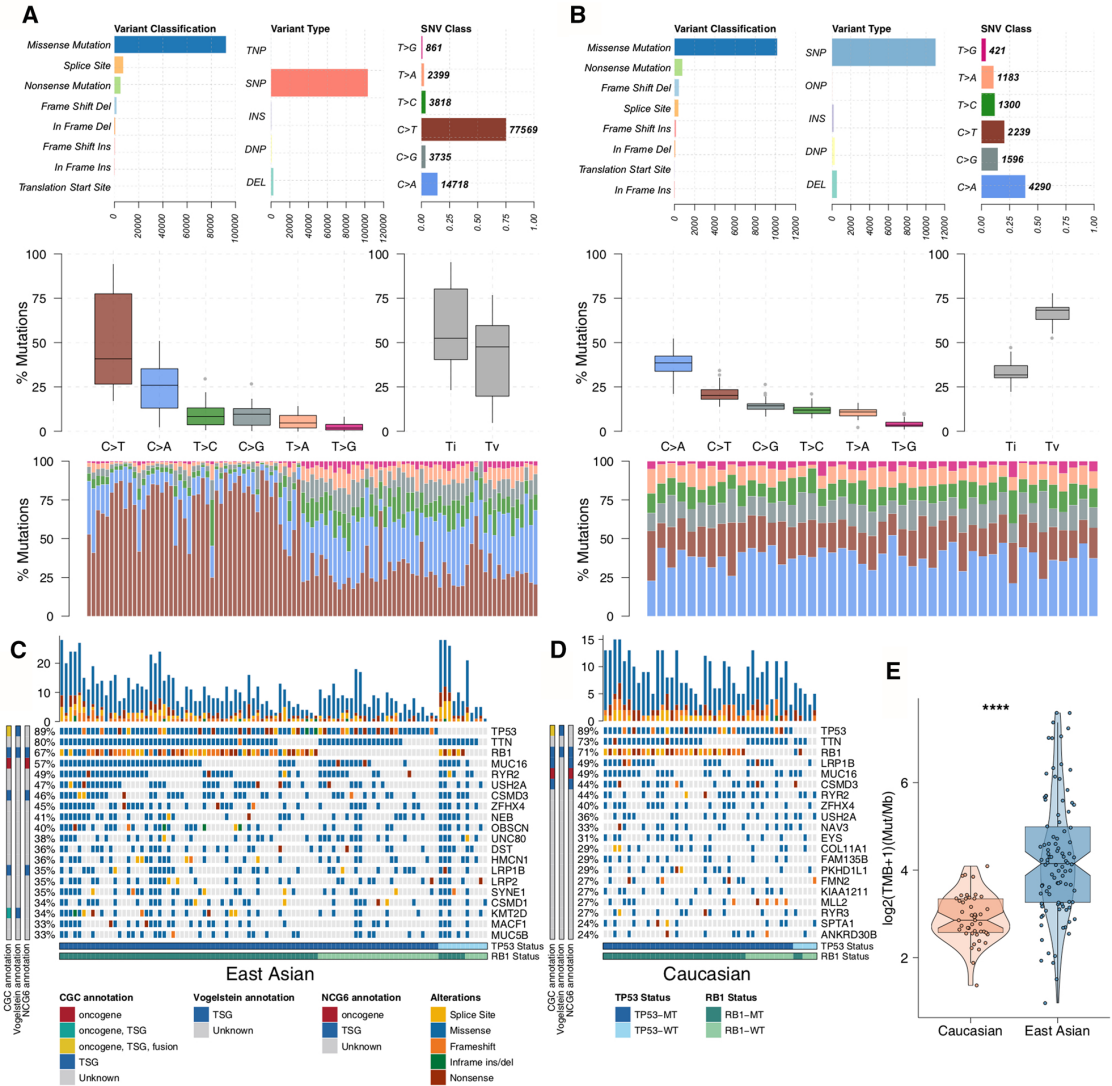
Genomic alterations in SCLC. An overview of variant classification, variant type, SNV class, and base substitution fractions of the East Asian (**a**) and Caucasian (**b**) cohorts. Oncoplot summarizing mutations for the top 20 mutated genes in the East Asian (**c**) and Caucasian (**d**) cohorts. **e** Comparison of the TMB in the East Asian and Caucasian cohorts

With the Mutect2 algorithm [24], 103,100 single-nucleotide polymorphisms (SNPs) and 2926 short insertions/deletions (indels) were identified in the EA cohort (Fig. 1a), and the majority were missense mutations (n = 92,103). We identified 11,029 SNPs and 676 indels in the Caucasian cohort (Fig. 1b). In particular, C > T was identified predominantly in the EA cohort, whereas C > A was the predominant single-nucleotide variant (SNV) in the Caucasian cohort (Fig. 1a,b). The top 20 mutated genes in the EA cohort are shown in Fig. 1c and *TP53* (89%), *TTN* (80%), *RB1* (67%), *MUC16* (57%), and *RYR2* (49%) were the most frequently mutated genes. The top 20 mutations in the Caucasian cohort are shown in Fig. 1d, and *TP53* (89%), *TTN* (73%), *RB1* (71%), *LRP1B* (49%), and *MUC16* (49%) were the most frequently mutated genes. We observed higher co-occurrence and exclusiveness gene pairs in the top 20 mutations of the EA cohort than of the Caucasian cohort (Additional file 1: Fig. S1). Oncogenes and tumor suppressor genes (TSGs) play a role in cancer evolution and development. For example, *LRP1B* and *MUC16* exhibited co-occurrence in the EA cohort (Additional file 1: Fig. S1a; Additional file 10: Table S1), while there was no co-occurrence/mutual exclusivity of oncogenes/TSGs in the Caucasian cohort (Additional file 1: Fig. S1b; Additional file 11: Table S2). The TMB was significantly higher in EA SCLC patients than in Caucasian SCLC patients (median 16.75 vs 6.24 per Mb; mean 30.95 vs 7.03 per Mb; FDR < 0.0001; Fig. 1e).

### *RB1* and *TP53* mutations in SCLC

**Fig. 2:**
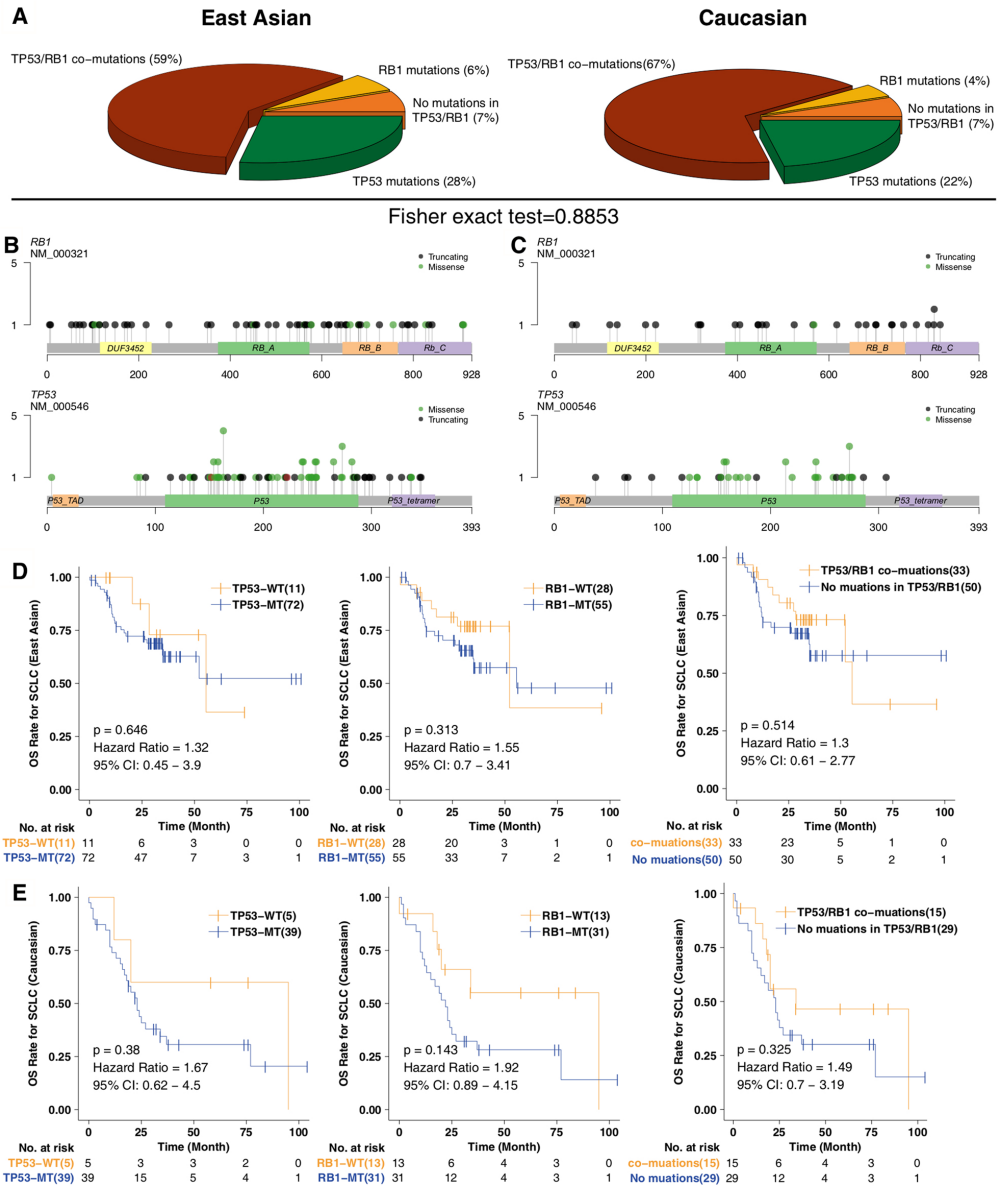
An overview of *TP53*/*RB1* co-mutations in SCLC. **a** The proportion of *TP53* mutations, *RB1* mutations, *TP53*/*RB1* co-mutations and no alterations in the East Asian and Caucasian cohorts. The mutation sites of *TP53* and *RB1* in the East Asian (**b**) and Caucasian (**c**) cohorts Kaplan–Meier survival analysis evaluated the relationship between the *TP53*/*RB1*/commutation statuses and OS of patients in the East Asian (**d)** and Caucasian (**e**) cohorts

Alterations in *RB1* and *TP53* occurred in approximately 65% and approximately 90% of the SCLC patients, respectively [1]. We identified that 59% of patients in the EA cohort had co-occurrent *TP53* and *RB1* mutations; 28% had only a *TP53* mutation, 6% had only an *RB1* mutation, and 7% had no alterations in *TP53* or *RB1* (Fig. 2a). The Caucasian cohort harbored more patients with alterations in both *TP53* and *RB1* (67%), although there was no significant difference between cohorts. In the EA cohort (Fig. 2b), multiple missense mutations occurred in the DNA-rich and tetramerization domains, while truncating mutations in *RB1* affected the DUF3452 and RB B, RB A and RB C domains. Similarly, *RB1* and *TP53* mutations affected the same domain in the Caucasian cohort (Fig. 2c). Subsequently, we explored the association between *RB1* and *TP53* mutations and the survival of SCLC patients. In the EA cohort, *TP53*, *RB1*, and *TP53*/*RB1* co-mutations exhibited no significant associations with a survival benefit (Fig. 2d). In addition, there were no correlations between survival and alterations in *RB1*, *TP53* or both *RB1* and *TP53* in the Caucasian cohort (Fig. 2e).

### Hallmark pathway alterations and significantly mutated drivers

**Fig. 3:**
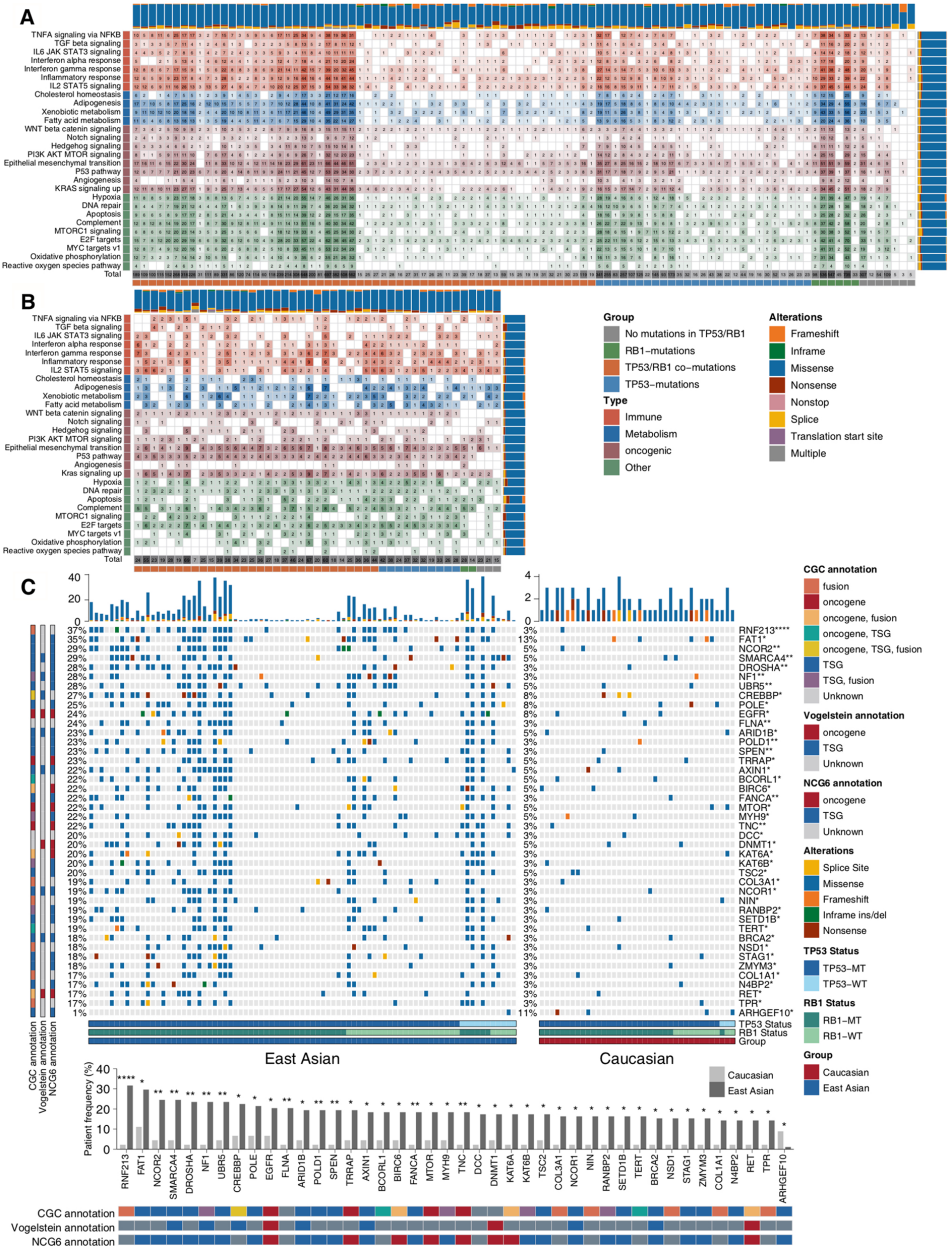
Genomic alterations in key biological signaling pathways in the East Asian (**a**) and Caucasian (**b**) cohorts. **c** Significantly mutated driver genes in the East Asian and Caucasian cohorts

For each SCLC patient, we calculated the frequency of patients harboring at least 1 alteration in each of the 28 signaling pathways in both the EA and Caucasian cohorts (Fig. 3a–b). Epithelial-mesenchymal transition (EMT) was the signaling pathway that was most frequently mutated (10.2% of all alterations) across all patients in the EA cohort, followed by *E2F* targets, *KRAS* signaling, *P53* signaling, *IL2/STAT5* signaling, hypoxia, adipogenesis, interferon-gamma response, and inflammatory response pathways, which were altered in 8.9%, 7.4%, 7.4%, 7.0%, 6.6%, 6.5%, 6.4% and 6.2% of all alterations, respectively. In contrast, reactive oxygen species pathways harbored the lowest number of alterations (Fig. 3a). Alterations in *EMT* signaling were identified most predominantly in the Caucasian cohort, while only some genes were altered in the angiogenesis and reactive oxygen species signaling pathways. More detailed information on the mutation frequencies of hallmark pathways is shown in Fig. 3b. Subsequently, with annotations from the Network of Cancer Genes (NCG) database, we compared the differences in frequencies of driver genes between the EA and Caucasian cohorts. Of the 42 significantly mutated genes between the two cohorts (all p < 0.05; Fig. 3c), TSGs were commonly involved (up to 54.8%). For alterations commonly found in the EA cohort, TSGs, such as *FAT1*, *NCOR2*, *SMARCA4*, *UBR5*, and *CREBBP*, showed higher alteration rates, while *ARHGEF10* exhibited a lower alteration rate than those found in the Caucasian cohort. Additionally, a well-known oncogene (*EGFR*) had higher numbers of alterations (mainly missense mutations) in the EA cohort than in the Caucasian cohort, followed by other oncogenes (*TRRAP*, *MTOR*, *TNC*, *DNMT1*, and *RET*). For mutual exclusivity and co-occurrence driver analyses, alterations in *MTOR*, *SPEN*, *NCOR1*, *BRCA2*, *POLE*, *EGFR*, *ARID1B,* and some other genes frequently co-occurred in the EA cohort (Additional file 2: Fig. S2a; Additional file 12: Table S3), whereas there were no significant mutual exclusivity gene pairs. In contrast, there were a few comutated gene pairs, namely, *BRCA2*, *FANCA* and *ZMYM3*, in the Caucasian cohort (Additional file 2: Fig. S2b; Additional file 13: Table S4).

### DNA damage repair pathway alterations and correlation with TMB

**Fig. 4:**
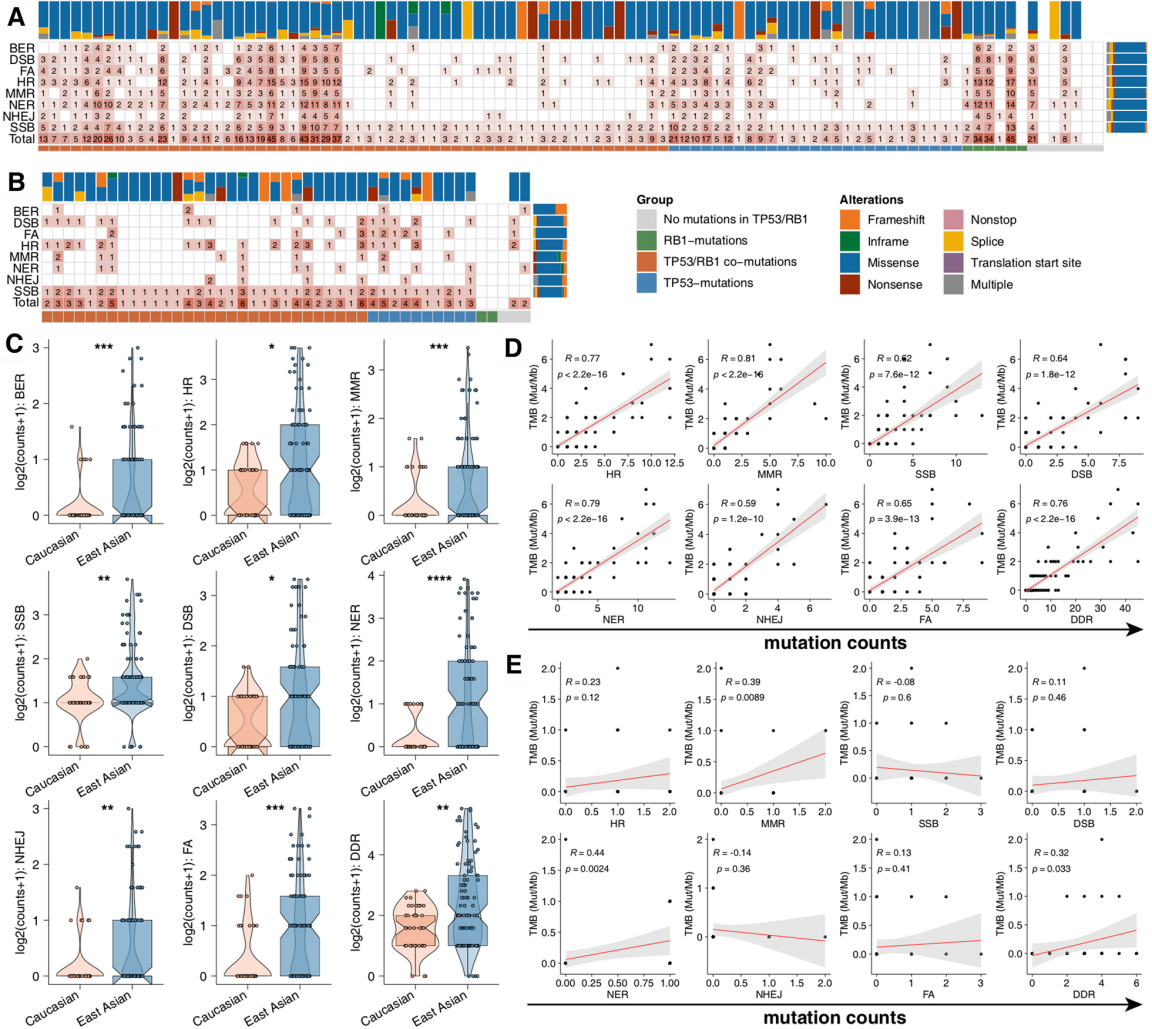
An overview of alterations in DDR signaling pathways in SCLC. Alterations in DDR signaling pathways in the East Asian (**a**) and Caucasian (**b**) cohorts. **c** Comparison of the number of mutations in each DDR pathway between the East Asian and Caucasian cohorts. The correlation between the number of mutations in each DDR pathway and TMB in the East Asian (**d**) and Caucasian (**e**) cohorts

Mutations in DDR pathways have been identified to affect the efficacy of platinum-based chemotherapy and immunotherapy for SCLC. For each DDR pathway, we computed the alteration frequencies of SCLC samples with at least 1 mutation in each of 8 signaling pathways (Fig. 4a–b). Homologous recombination (HR) was the pathway harboring the highest alteration frequencies among total mutations (32.2%), followed by single-strand breaks (SSB; 29.5%) and nucleotide excision repair (NER; 28.3%). However, nonhomologous end joining (NHEJ) pathways harbored the lowest fraction of alteration frequencies in the EA cohort (Fig. 4a). Next, we applied the same analytic pipeline in the Caucasian cohort. Genomic alterations in SSB signaling were highest, while mutations in base excision repair (BER) were the lowest (Fig. 4b). Additionally, some pathways, such as SSB and NHEJ, had mutations distributed among each SCLC patient in either the EA or Caucasian cohort (Fig. 4a, b). Additionally, the number of alterations in each DDR signaling pathway was higher in the EA cohort than in the Caucasia cohort (all adjusted p < 0.05; Fig. 4c). Next, we discovered that there was a significantly positive correlation between TMB and each DDR signaling pathway mutations (p < 0.05; Fig. 4d). By contrast, we discovered that only NER and DDR mutations were significantly related to higher TMB in the Caucasian cohort (Fig. 4e). Based on the ssGSEA method, none of the DDR signaling pathway alterations were significantly correlated with TMB in both the EA and Caucasian cohorts (Additional file 3: Fig. S3a–b).

### Significantly altered genes in key pathways, driver gene landscape and correlation with clinical benefit

**Fig. 5:**
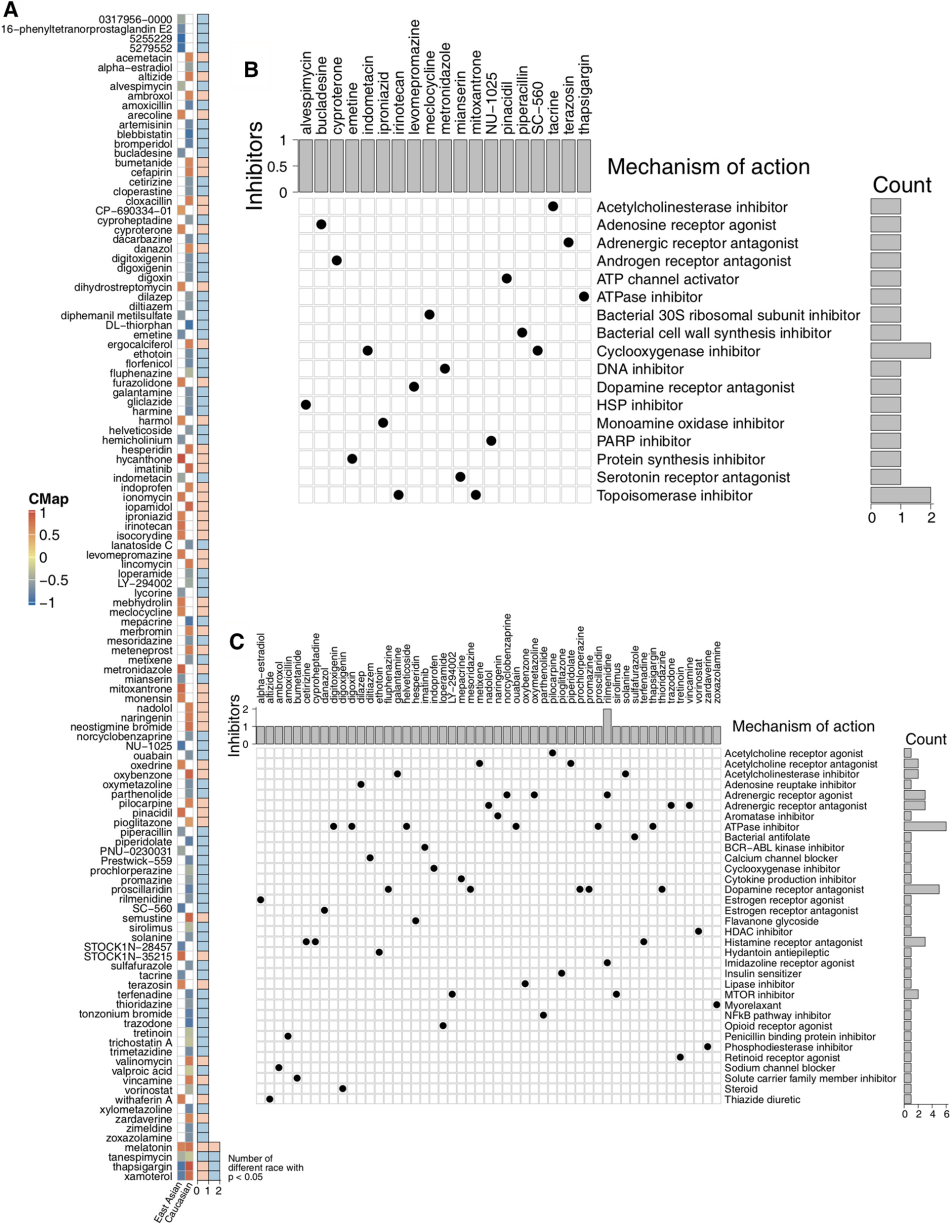
Correlation of *TP53*/*RB1* co-mutations with drug sensitivities: CMap analysis. **a** Heatmap showing the enrichment score (positive in blue, negative in red) of each compound from CMap for each cancer type. Compounds are sorted from right to left by descending number of cancer types significantly enriched. Heatmap showing each compound (perturbagen) from the CMap that shares MoAs (rows) and sorted by descending number of compounds with shared MoAs in the East Asian (**b**) and Caucasian (**c**) cohorts

The top twenty altered driver genes of the EA and Caucasian cohorts are characterized in Fig. 5a–b. We discovered up to 60% TSGs were driver genes in the EA cohort (Additional file 4: Fig. S4a), especially the top 5 altered mutations (*TP53*, *RB1*, *CSMD3*, and *LPR1B*). Up to 25% of oncogenes were commonly altered in the Caucasian cohort (Additional file 4: Fig. S4b), including *MUC16* (50%), *PREX2* (16%), *ZNF521* (14%), *ALK* (11%), *CTNND2* (11%) and *MUC4* (11%). Particularly interesting alterations across the EA and Caucasian cohorts were *TP53* and *RB1*. The main alteration type of *TP53* in the EA cohort was missense mutations (66.0%), followed by nonsense mutations (16.0%) and frameshift mutations (9.6%). In the Caucasian cohort, *TP53* was commonly altered as missense mutations (69.0%) and frameshift mutations (16.7%). In the EA cohort, *RB1* was frequently altered as frameshift mutations (31.5%), followed by nonsense mutations (28.8%), splice sites (23.3%), and missense mutations (16.4%). *RB1* had nearly equal distribution of the three mutation types (37.5% nonsense mutations, 31.3% spice site mutations and 28.1% frameshift mutations) in the Caucasian cohort. Through exploring significant alterations of key pathways, we combined several hallmark pathways and their genes to map nonsynonymous mutations. Multiple genes of each of the five pathways (DNA repair, EMT, G2M checkpoint, hypoxia, and *KRAS* signaling pathways) were significantly mutated in the EA and Caucasian cohorts (Additional file 4: Fig. S4c). For example, in the G2M checkpoint signaling pathway, mutations in TSGs, including *POLE* (26% vs 10%), *BRCA2* (19% vs 3%) and *STAG1* (19% vs 3%), occurred more commonly in the EA cohort than in the Caucasian cohort (all Fisher’s exact test p < 0.05). We identified *EGFR*, a well-known oncogene, as being mutated at a higher rate in EA patients than in Caucasian patients (25% vs 10%, Fisher’s exact test p < 0.05). *IGFBP2*, a protein in the EMT signaling pathway, was significantly more mutated in the Caucasian cohort than in the EA cohort (20% vs 4%, p < 0.05). Subsequently, we analyzed the potential association among driver genes, clinical phenotypes, and survival of SCLC patients using univariable Cox regression models. In the EA cohort, alterations in *APC*, *NSD3*, *KDM5C*, *CNTRL*, *GRM3*, *CTNND1*, *FANCG*, *MET*, and *SRGAP3* were associated with a significantly poor survival, but mutations in *TP53* or *RB1* conferred no survival benefits (Additional file 5: Fig. S5a). Subsequently, we identified two different driver genes (*FCRL4* and *PTPRT*) to stratify the Caucasian SCLC patients with the same analytical model (Additional file 5: Fig. S5b). However, alterations in *TP53* or *RB1* exhibited no potential associations with patient survival in the Caucasian cohort. Furthermore, we discovered four alterations with totally different prognosis values in the EA and Caucasian cohorts. For instance, driver gene mutations in *OTOF*, *ANKRD30B*, and *TECPR2* correlated with significantly shorter survival in the Caucasian cohort (Additional file 5: Fig. S5c), but these mutations showed no survival benefits in the EA cohort (Additional file 5: Fig. S5d). In contrast, alterations in *COL6A6* were associated with poor OS in EA SCLC patients (Additional file 5: Fig. S5d), while this mutation had no correlation with OS in Caucasian patients (Additional file 5: Fig. S5c).

### CMap algorithm identifies potential inhibitors/compounds associated with co-mutations in *RB1* and *TP53*

We applied the CMap algorithm for identifying associations among different groups and conditions to discover potential inhibitors/compounds targeting signaling pathways correlated with co-mutations in *TP53* and *RB1* (Fig. 5a; Additional file 14: Table S5, Additional file 15: Table S6). The adrenergic receptor antagonist terazosin, the ATP channel activator pinacidil, the topoisomerase inhibitors mitoxantrone and irinotecan, the heat shock protein (HSP) inhibitor alvespimycin, and the PARP inhibitor (PARPi) NU − 1025 showed significant correlations with *TP53* and *RB1* co-mutations in the EA cohort. We identified two compounds, the NFκB pathway inhibitor parthenolide and the ATPase inhibitor thapsigargin, that were significantly enriched in *TP53* and *RB1* co-mutations in the Caucasian cohort, but these inhibitors exhibited significantly negative correlations with *TP53* and *RB1* co-mutations among the EA cohort. Subsequently, applying CMap MoA analysis to the EA (Fig. 5b) and Caucasian cohorts (Fig. 5c), we discovered 17 mechanisms shared by 19 inhibitors/compounds in the EA cohort (Fig. 5b). Two compounds (irinotecan and mitoxantrone) shared MoAs of topoisomerase inhibitor. We identified SC − 560 and indometacin as cyclooxygenase inhibitors. In the Caucasian cohort (Fig. 5c), we found 34 mechanisms shared by 51 inhibitors/compounds, such as dopamine receptor antagonists (thioridazine, promazine, and prochlorperazine), ATPase inhibitors (proscillaridin, thapsigargin, ouabain, digoxin, helveticoside, and digitoxigenin), MTOR inhibitors (sirolimus and LY-294002) and an NFκB pathway inhibitor (parthenolide). Additionally, we calculated the drug sensitivity associated with more than two mutations from the top 20 mutated genes in the EA and Caucasian cohorts (Additional file 6: Fig. S6a, b).

### Immune profile analysis

**Fig. 6:**
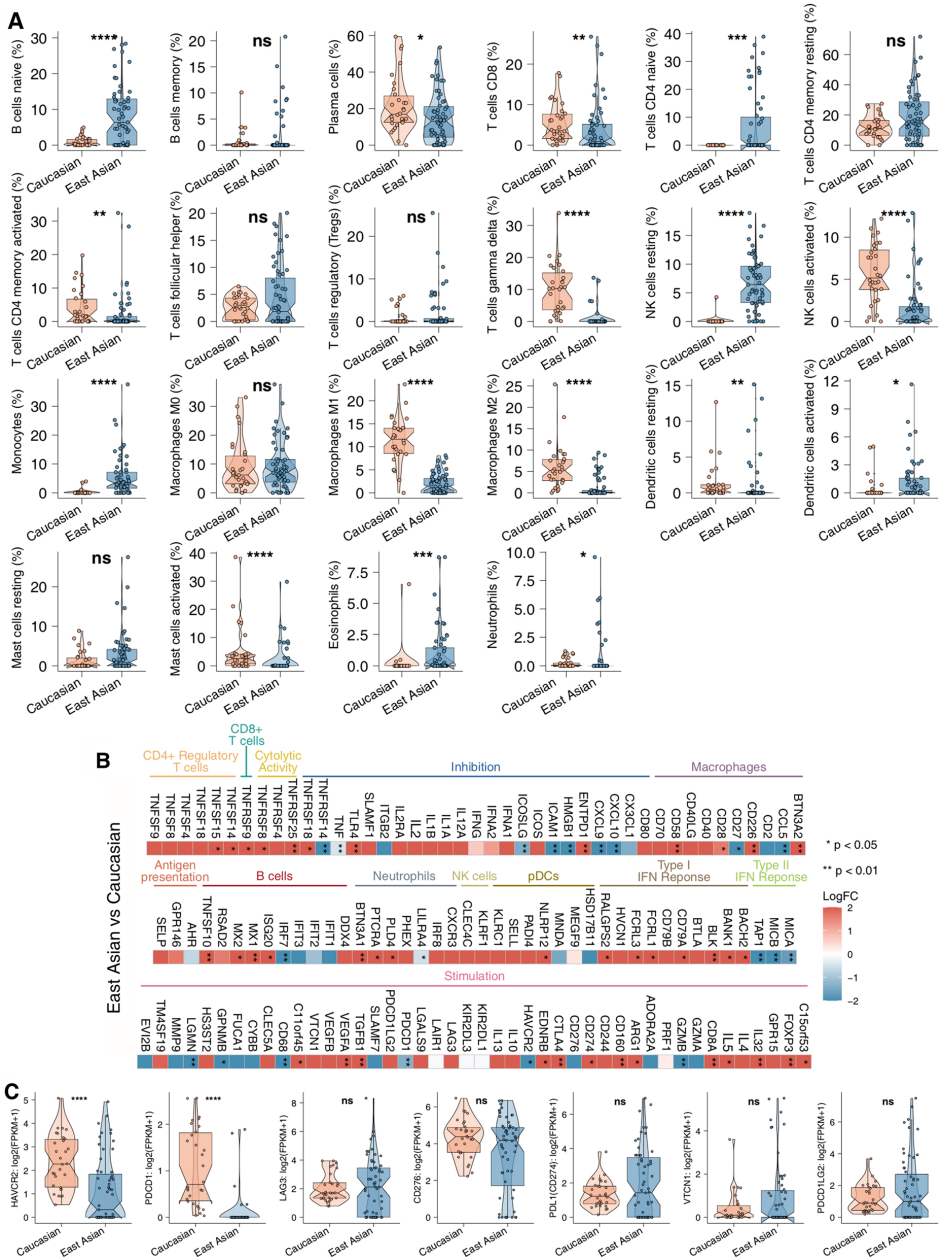
Immunological profiles in SCLC. **a** The difference in the contents of immune cells (CIBERSORT) between the East Asian and Caucasian cohorts. **b** Heatmap depicting the mean differences in immune-related gene mRNA expression levels between the East Asian and Caucasian SCLC cohorts. The y-axis indicates tumor-infiltrating leukocytes, immune signatures, or gene names. Each square represents the fold change or difference in each indicated tumor-infiltrating leukocyte, immune signature, or immune-related gene between the East Asian and Caucasian SCLC cohorts. Red indicates upregulation, while blue indicates downregulation. **c** The expression levels of immune checkpoints in the East Asian SCLC cohort versus the Caucasian SCLC cohort (*P < 0.05; **P < 0.01; ***P < 0.001; ****P < 0.0001)

CIBERSORT, an algorithm to evaluate the fractions of 22 immune cells, was applied to characterize the proportion of tumor-infiltrated immune cells in the EA and Caucasian cohorts. In total, 16 immune cells among the EA and Caucasian cohorts were characterized to be significantly different (Fig. 6a). For example, several immune cells, such as naïve B cells, naïve CD4 + T cells, resting memory CD4 + T cells, resting natural killer cells (NKs), monocytes, activated dendritic cells (DCs), eosinophils, and neutrophils, were significantly enriched in the EA populations, while some cells, such as plasma cells, CD8 + T cells, activated memory CD4 + T cells, activated NKs, M1-type macrophages, M2-type macrophages, resting DCs and activated mast cells, accounted for significantly more immune cells in the Caucasian cohort than in the EA cohort. In addition, we compared the differences in the immune-infiltrated signature between the EA and Caucasian cohorts. The mean differences (log fold change) in the 22 immune cells between clinical and genomic features in the EA and Caucasian cohorts are shown in Additional file 7: Fig. S7a, b. Figure 6b shows the mean differences (log fold change) in immune-related mRNA expression levels between EA and Caucasian SCLC patients. Several inhibitory mediators, such as *VEGFA*, *TGFB1*, and *FOXP3*, were significantly upregulated in EA SCLC patients, but some antigen presentation genes, such as *MICA*, *MICB*, and *TAP1*, were significantly downregulated in the EA cohort. Additionally, chemokines (*CXCL9* and *CXCL10*) and cytolytic activity-related genes (*GZMB*) were commonly downregulated in the EA cohort. The mean differences (log fold change) in the clinical and genomic features of immune-related genes in the EA and Caucasian cohorts are shown in Additional file 8: Fig. S8a, b. Notably, among the immune checkpoint-related genes in both the EA and Caucasian cohorts, we discovered that two genes (*PDCD1* and *HAVCR2*) were significantly enriched in the Caucasian cohort compared with the EA cohort (Fig. 6c). The correlation analysis between the TMB, DDR, and immune cells in the EA and Caucasian cohort was shown in Additional file 9: Fig. S9. In the Caucasian cohort, we found that there was a positive correlation between the proportions of the activated NK cells and the DDR mutation counts or TMB. Similarly, the abundance of the activated DCs was positively associated with the DDR or, SSB, or MMR mutation counts. The proportion of the follicular helper T cells was positively correlated with the TMB (Additional file 9: Fig. S9a). In the EA cohort, there was a negative correlation between the proportion of the monocytes and the HR, DSB, NHEJ, FA, or DDR mutation counts. Additionally, we found that the abundance of the resting NK cells was positively correlated with the BER mutation counts (Additional file 9: Fig. S9b).

## Discussion

Here, we performed a comprehensive clinical, genomic, and immunological analysis based on the WES and transcriptome data in EA SCLC patients and further compared the results with a previously published dataset of Caucasian SCLC patients (reported by George et al.). We identified that *LRP1B* and *MUC16* were co-occurrent in the EA cohort, while there was no co-occurrence/mutual exclusivity of oncogenes/TSG in the Caucasian cohort. *LRP1B* plays a critical role in cell adhesion, focal adhesion, and tight junction disruption and further inhibits tumor cell migration and proliferation [25–27]. *MUC16*, a well-known mechanical barrier gene, serves as a serum biomarker among various cancers [28]. Ge et al. found that mutations in a panel of five genes, including mutations in *LRP1B* and *MUC16*, predicted poor survival in colorectal cancer, and *LRP1B* and *MUC16* mutations may be involved in tumor metastasis by regulating focal adhesion and cell adhesion [29]. Furthermore, there were no significant differences in the mutation frequencies of two known alterations (e.g., *TP53* and *RB1*) between the EA and Caucasian cohorts.

In the EA SCLC cohort, the number of alterations in gene related to EMT signaling were the highest among the critical biological signaling pathways. Through the EMT mechanism, cancer cells can obtain a motile phenotype, mediate tumor cell metastasis and secondary resistance to common chemotherapies or targeted treatments [30]. Additionally, EMT is associated with poor survival in SCLC [30, 31], and EMT plays a key role in the activation of several oncogenic signaling pathways, such as TGFβ/Akt and MET signaling pathways [30–32]. *CREBBP* mutation rates were notably higher (27%) in the EA cohort than in the Caucasian cohort (8%). *CREBBP* acts as an ubiquitous transcriptional coactivator and histone modifier [1, 33], and *CREBBP* inactivation can promote cell growth in SCLC [33]. Importantly, *CREBBP* is frequently mutated in SCLC [34]. Moreover, treatment with pracinostat, a histone deacetylase inhibitor (HDACi), can increase E-cadherin and acetylated *H3K27*, further reversing the function of *CREBBP* mutations [35, 36]. Additionally, significantly higher mutation rates were identified for *EGFR* in EA SCLC tumors (25%) than in the Caucasian tumors (10%). Studies have found that EGFR tyrosine kinase inhibitor (TKI)-resistant tumors transformed from non-small cell lung cancer (NSCLC) into SCLC and were sensitive to standard therapies for SCLC [37–39].

This study is interesting given the critical role of *TP53*/*RB1* co-mutations in SCLC tumors. Compared to the *TP53*/*RB1* co-mutations in the Caucasian cohort, an 8% decrease in *TP53*/*RB1* co-mutations was identified in the EA cohort (67% vs 59%), which is consistent with results from other studies [1, 4, 40]. However, mutations, such as *TP53*, *RB1*, and *TP53*/*RB1* comutation, were not found to be significantly associated with clinical benefits in both the EA and Caucasian cohorts. A mutation in *OTOF*, a calcium-sensing protein triggering cell membrane fusion and regulating exocytosis, was significantly associated with poor OS in the Caucasian cohort [41, 42]. APC mutations were significantly correlated with shorter OS in the EA cohort, meditating faster tumorigenesis [43]. Mondaca et al. found that APC alterations were associated with the clinical outcomes of colorectal cancer patients [44]. Evidence has indicated that *NSD3* has crucial effects on cancer cell proliferation and invasion via multiple signaling pathways [45–47]. In the EA cohort, *NSD3* mutations were detected to be correlated with poor OS. Consistent with the previous study, *KDM5C* mutations had prognostic implications in EA SCLC patients [48]. A GRM3 mutation was previously shown to upregulate MAPK pathway activity [49], and its presence was correlated with shorter survival. *CTNND1* was previously identified to bind and stabilize cadherins, further regulating Wnt/β-catenin signaling pathway activity during tumor progression [50, 51], and the *CTNND1* mutation was associated with adverse OS in the EA cohort. *FANCG* was shown to play an important role in the activation of the Fanconi anemia (FA) pathway with the localization of the nuclear FA complex (including *FANCG*) [52], and it can also interact with *FANCD1* (*BRCA2*) [53, 54]. In our findings, we identified that a *FANCD1* mutation was associated with unfavorable OS in the EA SCLC cohort. In lung cancer, *MET* alterations have been suggested to be associated with a poor prognosis [55], and their presence was associated with shorter OS in the EA SCLC cohort. Long et.al reported that *COL6A6* interacted with *P4HA3* to suppress the growth and metastasis of pituitary adenoma via blocking the PI3K-Akt pathway [56]. Additionally, Qiao et.al. Indicated that that *COL6A6* was a tumor suppressor gene in NSCLC and was involved in NSCLC tumorigenesis by regulating the JAK signalling pathway [57].

It is crucial to characterize the immunological profile of SCLC in the EA population, as this landscape might indicate the molecular mechanism of response, efficacy and resistance to specific immunotherapy and provide novel and potential implications of combination therapy. For example, high TMB and alterations in DDR were previously identified to be strongly correlated with better survival in patients who underwent ICI treatments [11, 58, 59]. Through accumulating incorrect DNA damage, tumors harboring higher DDR mutations commonly had a higher TMB level [59]. Notably, significantly higher TMB levels were observed in the EA cohort (median 16.75 Mut/Mb; mean 30.95 Mut/Mb) than those in the Caucasian cohort (median 6.24 Mut/Mb; mean 7.03 Mut/Mb). Additionally, the number of DDR alterations was significantly higher in EA SCLC tumors than in Caucasian SCLC tumors. We found that high TMB was significantly positively correlated with high DDR alterations. Preclinical SCLC models were sensitive to PARP inhibition alone and the efficacy of chemotherapy was also enhanced by the addition of a PARP inhibitor [60]. Additionally, recent studies have shown that the efficacy of immunotherapy is related to a high TMB, high genomic instability, and high immunogenicity in tumor cells [61]. Moreover, a subset of patients responded to the anti-PD-1 agent nivolumab or pembrolizumab when administered as the third or later treatment line (response rates 12–20%) and experienced very prolonged responses, as median durations of response were 17.9 months and not-reached (after 7.7 months of follow-up), respectively [62, 63]. An important observation from this study is that East Asian SCLC patients have high mutation counts of DDR signaling pathways and TMB, which raises the question of combination approaches using PARPis and ICIs [64].

In addition to TMB and DDR alterations, the inflammatory gene expression profile (GEP), specific immune cells (e.g., CD4 + T cells, CD8 + T cells), and immune checkpoint expression levels played a critical role in SCLC treated with ICIs [11, 65]. Using the CIBERSORT algorithm, there were higher proportions of resting-type immune cells, such as naïve B cells, naïve CD4 + T cells, resting memory CD4 + T cells, resting NKs and resting DCs, in the EA SCLC cohort than in the Caucasian cohort. TGF-β signaling, containing *TGFB1*, has been reported to disrupt the recruitment and infiltration of CD8 + T cells into the center of tumors [66]. Treatment with PD-(L) 1 can facilitate T-cell infiltration, provoke antitumor immunity and attenuate tumor progression [66]. *FOXP3*, a conventional biomarker for regulatory T cells (Tregs), can attenuate effective T cell (Teff) activity and is associated with clinical benefits in several tumors [67–69]. Emerging studies have indicated that *VEGFA* overexpression tends to involve a suppressive tumor microenvironment (TME) and decreased antitumor immunity [70, 71], further mediating primary resistance to the anti-PD-(L) 1 regimen. Here, we discovered that there was a high expression level of several suppressive mediators, such as *VEGFA*, *TGFB1* and *FOXP3*, in the EA cohort. In contrast, chemokines (*CXCL9* and *CXCL10*) and a cytolytic activity-related gene (*GZMB*) were commonly downregulated in the EA cohort. Chemokines, such as *CXCL9* and *CXCL10*, serve as key factors that recruit Teffs into the center of the tumor, further promoting antitumor immunity and disrupting tumor cell proliferation and invasion [11, 72–74].

Using the CMap algorithm, we identified potential inhibitors/compounds that may be capable of targeting *TP53*/*RB1* co-mutations, such as the topoisomerase inhibitors mitoxantrone and irinotecan, the HSP inhibitor alvespimycin, and the PARPi NU − 1025. Alterations in DDR signaling pathways have significance in the usage of genotoxic agents, such as platinum-based chemotherapy and PARPi [75–78]. Additionally, unrepaired DNA mediates immune priming by multiple molecular mechanisms and upregulates PD-(L) 1 expression [79]. Furthermore, PARPi was involved in the development of the inflammatory TME and further promoted a productive immune response [79–81].

However, this study had certain limitations. First, due to the limited number of Caucasian SCLC patients, this finding might need a large population for validation. Second, intratumor heterogeneity was a crucial metric for tumor evolution, but our analyses were based only on single biopsies/samples; therefore, our findings cannot portray the whole evolution of SCLC. Third, this study lacks copy number variation and proteomics analyses to validate our findings. Finally, animal and laboratory experiments are necessary to further illustrate and validate our findings.

## Conclusions

In summary, the present findings portray the clinical, immunological, and genomic profile differences among EA and Caucasian SCLC patients and might provide clinical implications for EA SCLC patients with novel alterations, potential biological signaling pathways and new immunological factors to target.

## Supplementary Information

**Additional file 1: Figure S1.** Related to Fig. 1c–d; heatmap showing mutually exclusive and co-occurring mutations in the East Asian (**a**) and Caucasian (**b**) cohorts.

**Additional file 2: Figure S2.** Related to Fig. 3c; heatmap showing mutually exclusive and co-occurring mutations in the East Asian (**a**) and Caucasian (**b**) cohorts.

**Additional file 3: Figure S3.** Related to Fig. 4; the correlation between the ssGSEA scores of each DDR signaling pathway and TMB in the East Asian (**a**) and Caucasian (**b**) cohorts.

**Additional file 4: Figure S4.** Top 20 mutated drivers and alterations in several key biological pathways in SCLC. An overview of the top 20 mutated driver genes in the East Asian (**a**) and Caucasian (**b**) cohorts. **c** Significantly mutated genes in the key biological signaling pathways between the East Asian and Caucasian cohorts.

**Additional file 5: Figure** **S5**.The distinct prognosis of significant driver gene mutations in SCLC. The univariable Cox regression model includes clinical characteristics and driver gene mutations (mutation rate ≥ 10%) in the East Asian (**a**) and Caucasian (**b**) cohorts. Several driver gene mutations (mutation rate ≥ 10%) were associated with a distinct prognosis between the East Asian (**c**) and Caucasian (**d**) cohorts.

**Additional file 6: Figure S6.** Related to Additional file 5: Fig. S5; heatmap depicting the mean differences in drug sensitivity (GDSC database) between the top 20 mutated genes and the corresponding wild-type gene in the East Asian (**a**) and Caucasian (**b**) cohorts. The y-axis indicates different drugs in the GDSC database, and the x-axis of the heatmap indicates different mutation statuses of the top 20 mutated genes. Red indicates upregulation, while blue indicates downregulation.

**Additional file 7: Figure S7.** Related to Fig. 6a; heatmap depicting the mean differences in the contents of 22 immune cells between different clinical characteristics and mutation status in the East Asian (**a**) and Caucasian (**b**) cohorts. The y-axis indicates different immune cells calculated by the CIBERSORT algorithm, and the x-axis of the heatmap indicates clinical characteristics and mutation status. Red indicates upregulation, while blue indicates downregulation.

**Additional file 8: Figure S8.** Related to Fig. 6b; heatmap depicting the mean differences in the contents of immune-related gene mRNA expression between different clinical characteristics and mutation statuses in the East Asian (**a**) and Caucasian (**b**) cohorts. The y-axis indicates immune-related gene mRNA expression, and the x-axis of the heatmap indicates clinical characteristics and mutation status. Red indicates upregulation, while blue indicates downregulation.

**Additional file 9: Figure S9.** Heatmap depicting the correlation rho between the proportion of immune cells and mutations in each DDR pathway and TMB in the East Asian (**a**) and Caucasian (**b**) cohorts.

**Additional file 10: Table S1.** Related to Additional file 1: Fig. S1a. The results of co-occurrence/mutual exclusivity of oncogenes/TSGs in the East Asian cohort (Top20 mutated genes).

**Additional file 11: Table S2.** Related to Additional file 1: Fig. S1b. The results of the co-occurrence/mutual exclusivity of oncogenes/TSGs in the Caucasian cohort (Top20 mutated genes).

**Additional file 12: Table S3.** Related to Additional file 2: Fig. S2a. The results of the co-occurrence/mutual exclusivity of oncogenes/TSGs in the East Asian cohort (significantly mutated driver genes).

**Additional file 13:** **Table S4.** Related to Additional file 2: Fig. S2b. The results of the co-occurrence/mutual exclusivity of oncogenes/TSGs in the Caucasian cohort (significantly mutated driver genes).

**Additional file 14: Table S5.** Related to Additional file 5: Fig. S5a. The results of the CMap analysis of the East Asian cohort (TP53/RB1 co-mutations vs No alterations in TP53/RB1).

**Additional file 15:** **Table S6.** Related to Additional file 5: Fig. S5aThe results of the CMap analysis of the Caucasian cohort (TP53/RB1 co-mutations vs No alterations in TP53/RB1).

**Additional file 16: **Supplemental methods.

## Data Availability

All the data generated or analyzed during this study are included in this published article (https://www.cbioportal.org/study/summary?id=sclc_ucologne_2015) and our supplementary files. All other relevant data are available from the authors of this study upon request.

## Abbreviations

SCLC: Small-cell lung cancer
EA: East Asian TMB: tumor mutation burden
EMT: Epithelial-mesenchymal transition
OS: Overall survival ICIs: immune checkpoint inhibitors
TME: Tumor microenvironment
WES: Whole-exome sequencing
IRBs: Institutional Review Boards
RNA-seq: RNA sequencing
GDSC: Genomics of Drug Sensitivity in Cancer
DDR: DNA damage response
ssGSEA: Single-sample gene set enrichment analysis
NCG: Network of Cancer Genes
CMap: Connectivity Map
MoA: Mechanism of action
GSEA: Gene set enrichment analysis
FDR: False discovery rate
SNPs: Single-nucleotide polymorphisms
SNV: Single-nucleotide variant
TSGs: Tumor suppressor genes
HR: Homologous recombination
SSB: Single-strand breaks
NHEJ: Nonhomologous end joining
NER: Nucleotide excision repair
BER: Base excision repair
HSP: Heat shock protein
PARPi: PARP inhibitor
DCs: Dendritic cells
NKs: Natural killer cells
