# Supplementary figures and images for: Genomic and immunological profiles of small-cell lung cancer between East Asians and Caucasian
Source: Cancer Cell Int. 2022 Apr 29;22:173. doi: 10.1186/s12935-022-02588-w (PMC9052616; doi:10.1186/s12935-022-02588-w)

**A**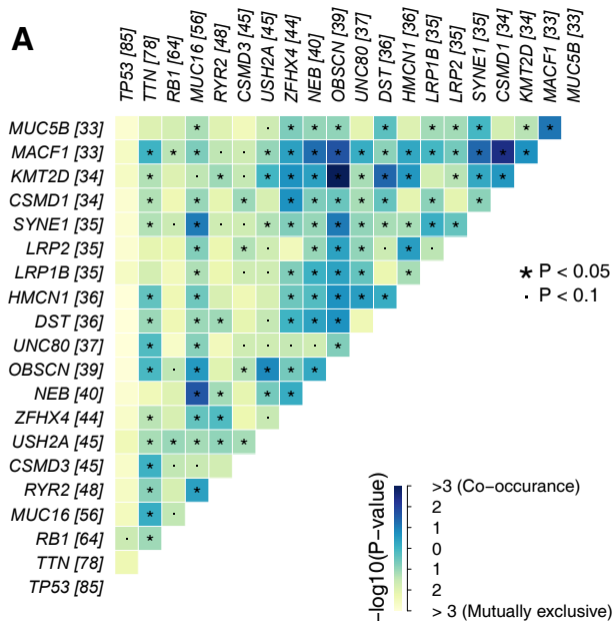**B**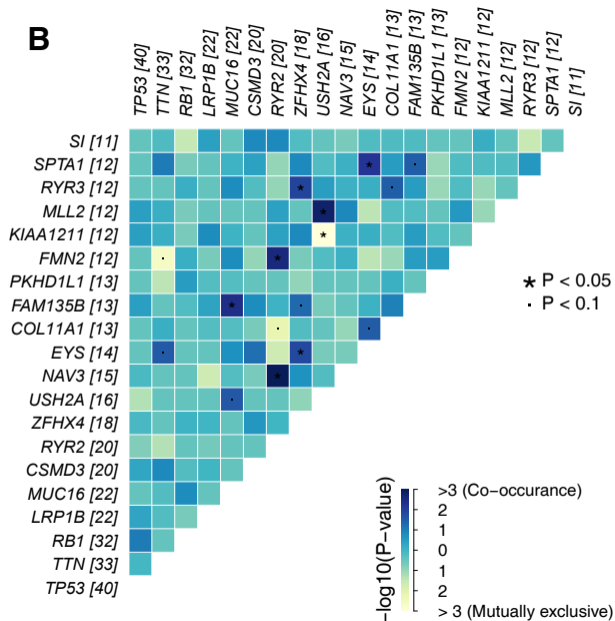

Supplement: Supplementary file 1 — Additional file 1: Figure S1. Related to Fig. 1c–d; heatmap showing mutually exclusive and co-occurring mutations in the East Asian (a) and Caucasian (b) cohorts. [file 12935_2022_2588_MOESM1_ESM.pdf]

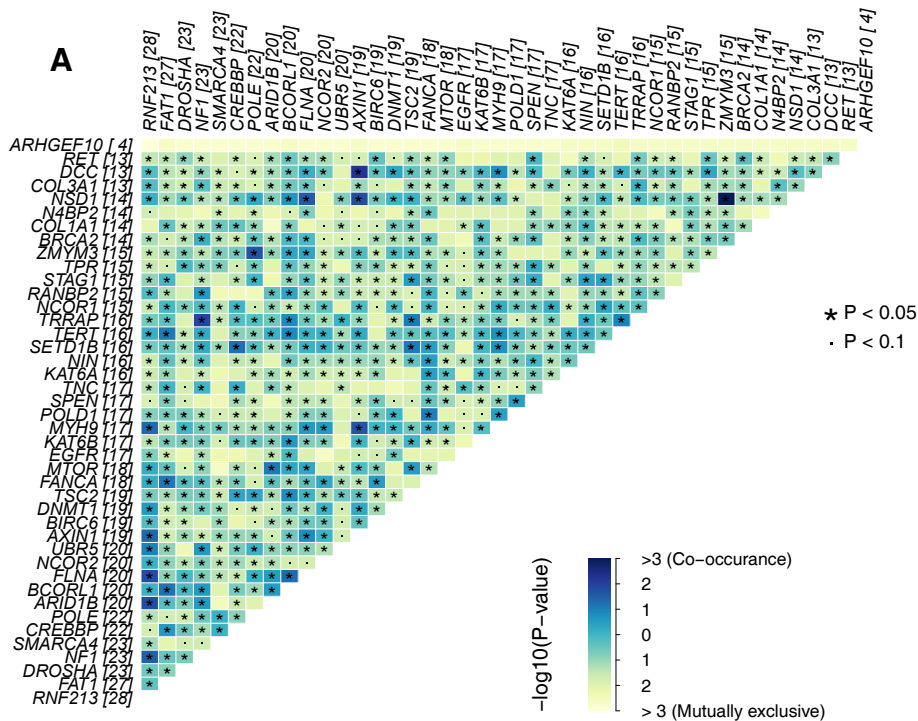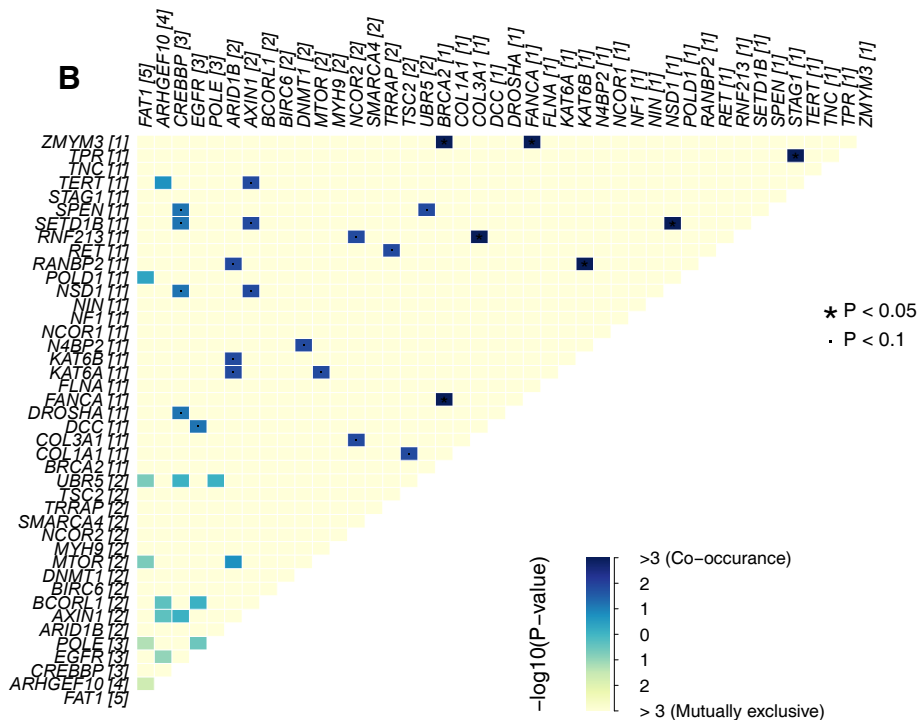

Supplement: Supplementary file 2 — Additional file 2: Figure S2. Related to Fig. 3c; heatmap showing mutually exclusive and co-occurring mutations in the East Asian (a) and Caucasian (b) cohorts. [file 12935_2022_2588_MOESM2_ESM.pdf]

**A East Asian**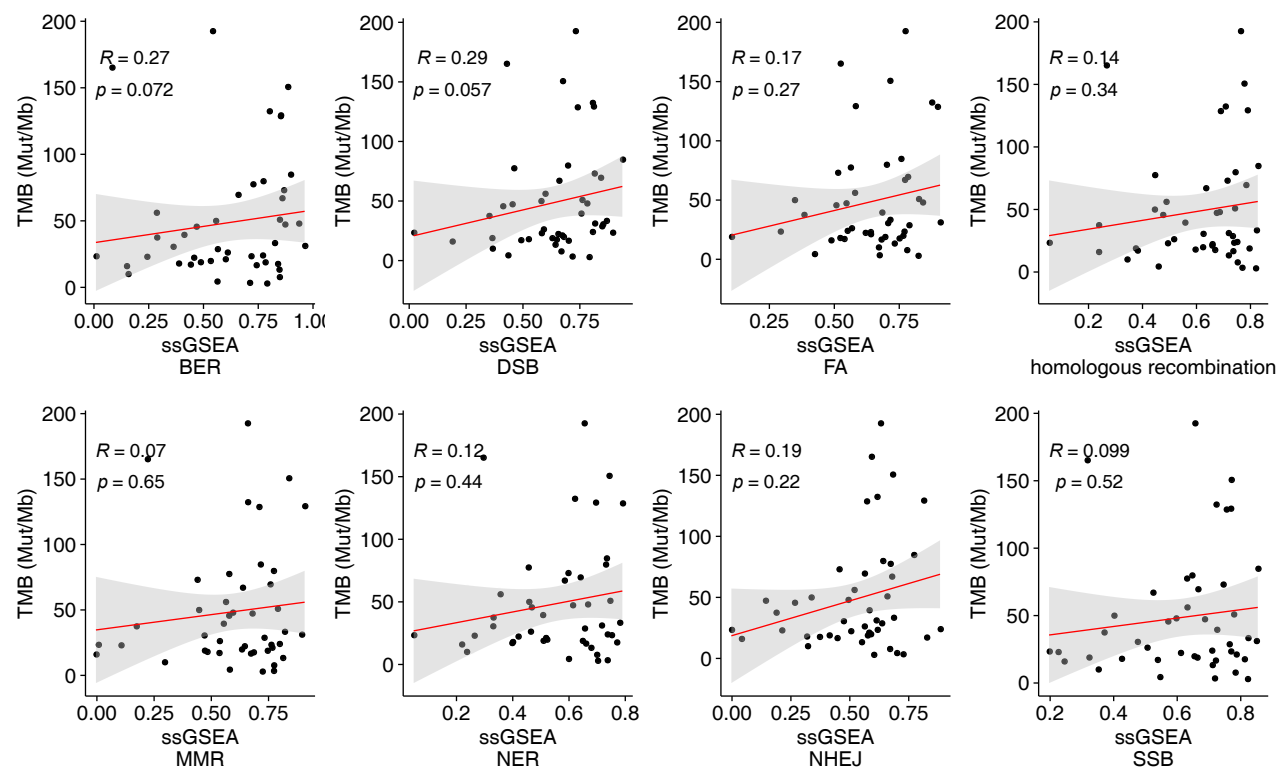**B Caucasian**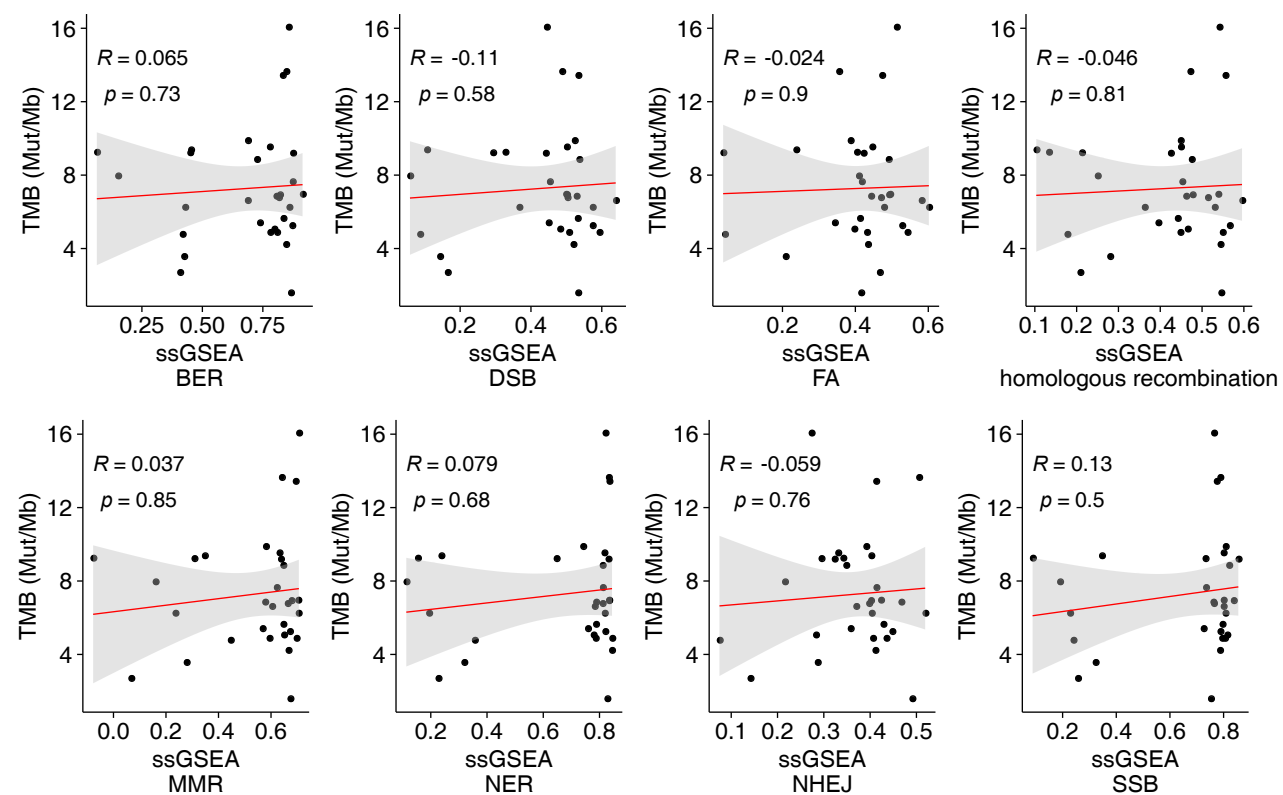

Supplement: Supplementary file 3 — Additional file 3: Figure S3. Related to Fig. 4; the correlation between the ssGSEA scores of each DDR signaling pathway and TMB in the East Asian (a) and Caucasian (b) cohorts. [file 12935_2022_2588_MOESM3_ESM.pdf]

## East Asian

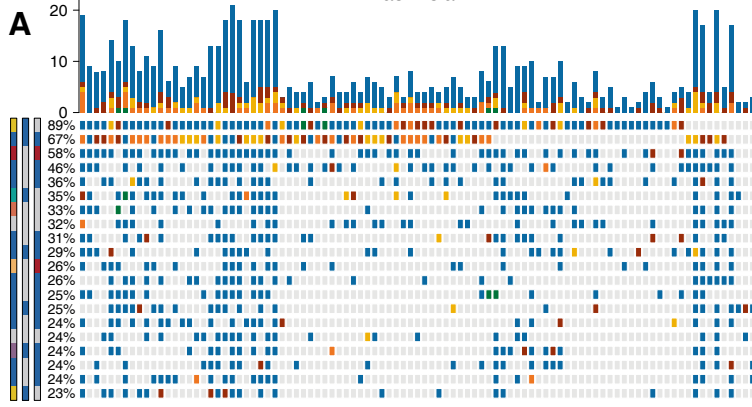

## Caucasian

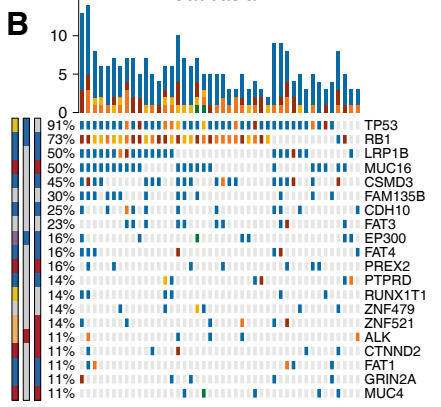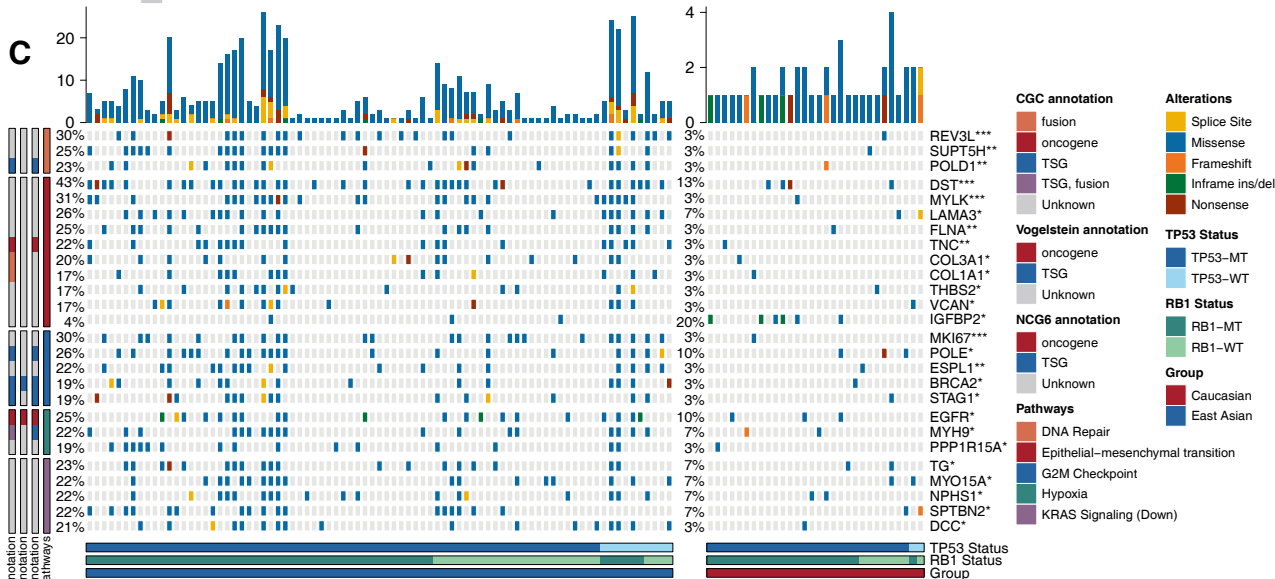

## East Asian

## Caucasian

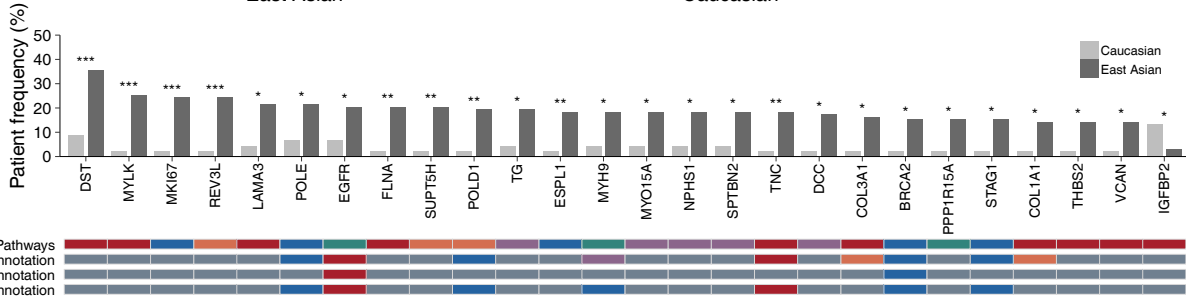

Supplement: Supplementary file 4 — Additional file 4: Figure S4. Top 20 mutated drivers and alterations in several key biological pathways in SCLC. An overview of the top 20 mutated driver genes in the East Asian (a) and Caucasian (b) cohorts. c Significantly mutated genes in the key biological signaling pathways between the East Asian and Caucasian cohorts. [file 12935_2022_2588_MOESM4_ESM.pdf]

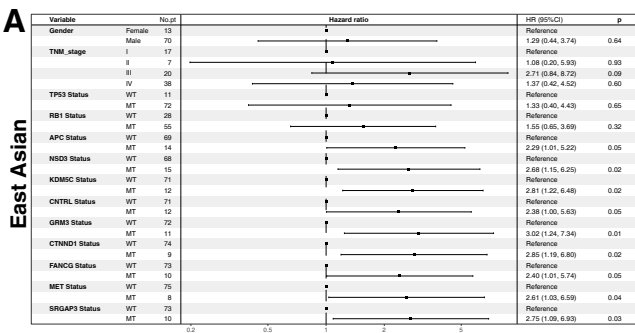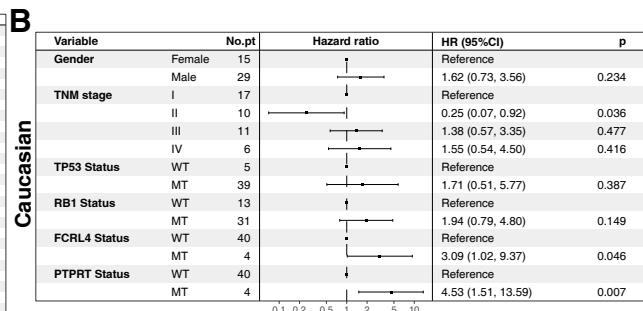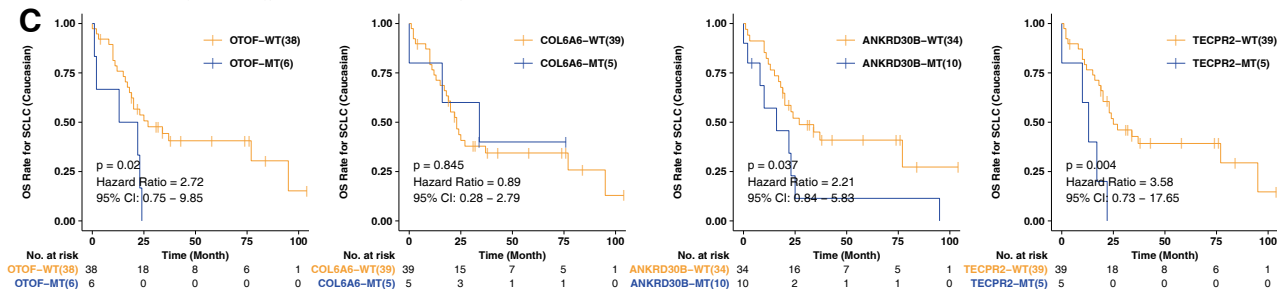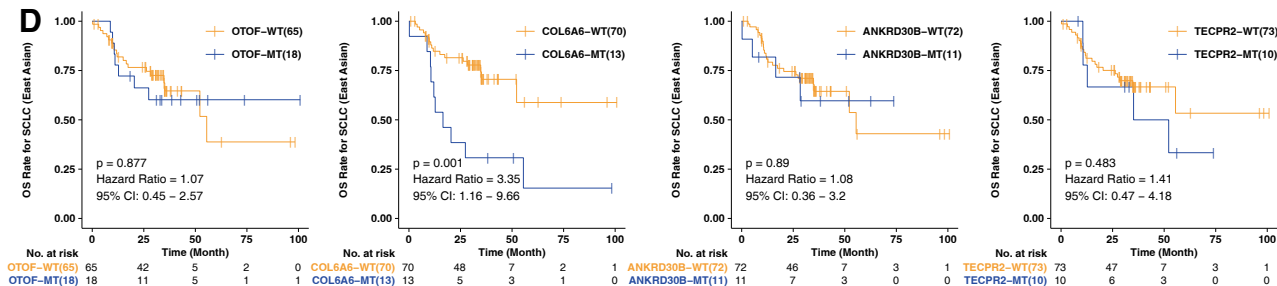

Supplement: Supplementary file 5 — Additional file 5: Figure S5.The distinct prognosis of significant driver gene mutations in SCLC. The univariable Cox regression model includes clinical characteristics and driver gene mutations (mutation rate ≥ 10%) in the East Asian (a) and Caucasian (b) cohorts. Several driver gene mutations (mutation rate ≥ 10%) were associated with a distinct prognosis between the East Asian (c) and Caucasian (d) cohorts. [file 12935_2022_2588_MOESM5_ESM.pdf]

**A**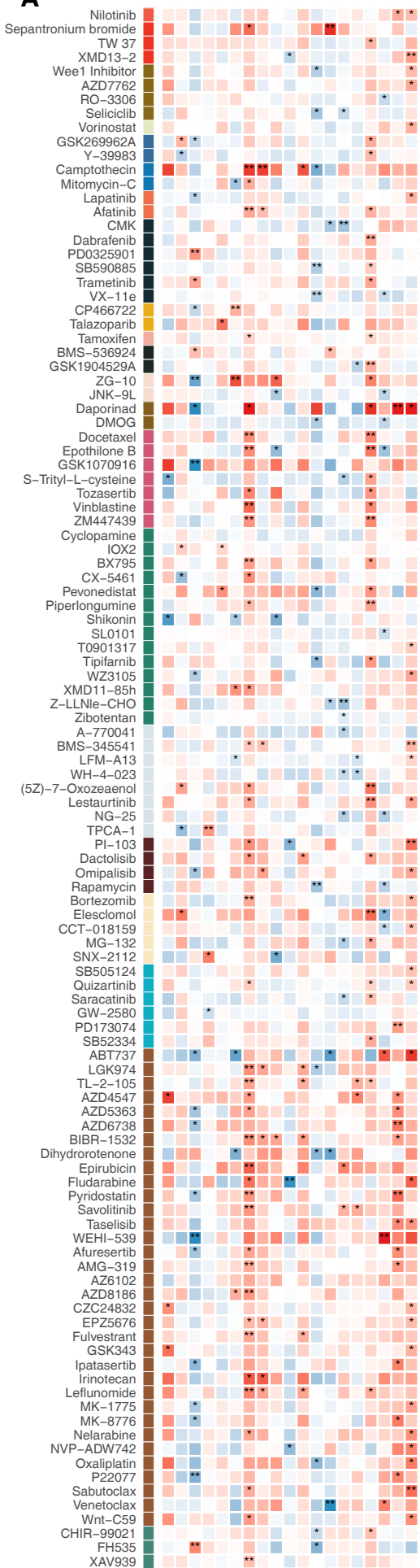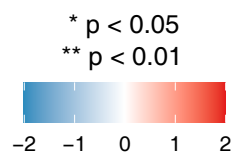**B**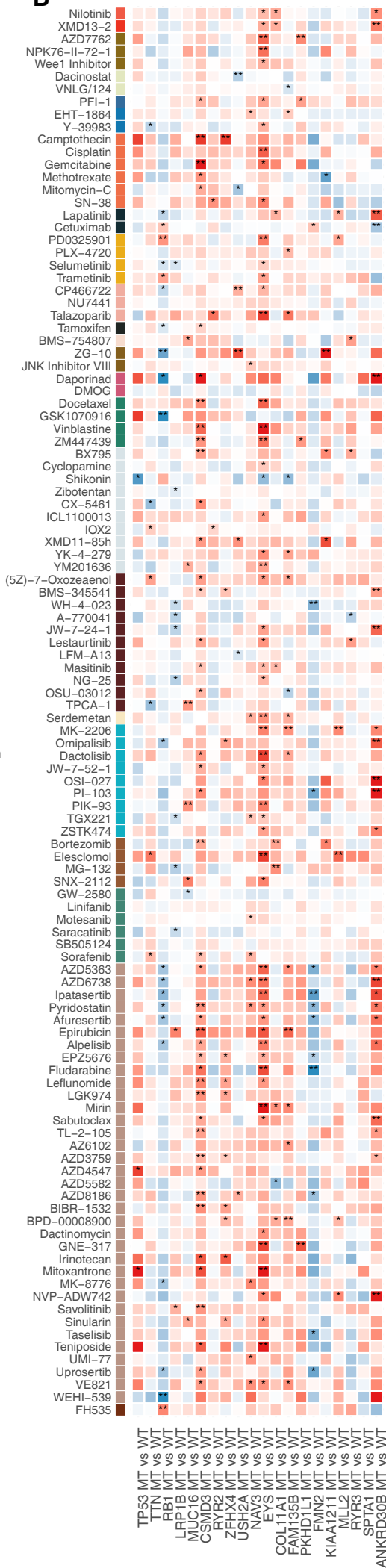

Supplement: Supplementary file 6 — Additional file 6: Figure S6. Related to Additional file 5: Fig. S5; heatmap depicting the mean differences in drug sensitivity (GDSC database) between the top 20 mutated genes and the corresponding wild-type gene in the East Asian (a) and Caucasian (b) cohorts. The y-axis indicates different drugs in the GDSC database, and the x-axis of the heatmap indicates different mutation statuses of the top 20 mutated genes. Red indicates upregulation, while blue indicates downregulation. [file 12935_2022_2588_MOESM6_ESM.pdf]

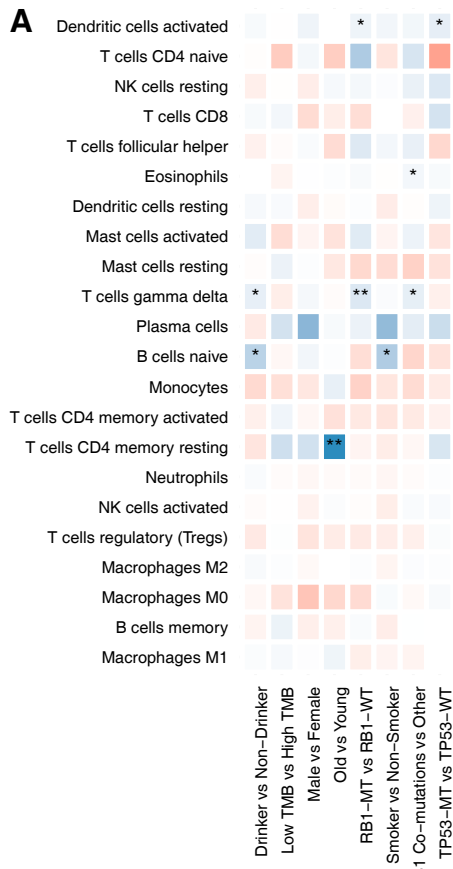

\* p < 0.05

\*\* p < 0.01

LogFC

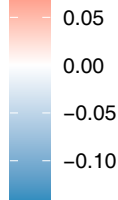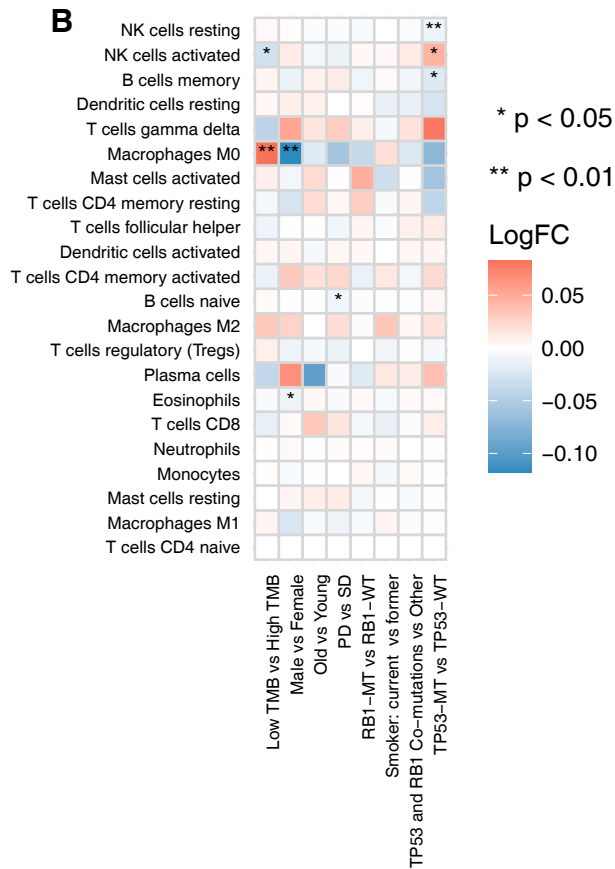

\* p < 0.05

\*\* p < 0.01

LogFC

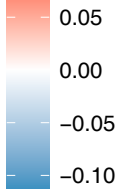

Supplement: Supplementary file 7 — Additional file 7: Figure S7. Related to Fig. 6a; heatmap depicting the mean differences in the contents of 22 immune cells between different clinical characteristics and mutation status in the East Asian (a) and Caucasian (b) cohorts. The y-axis indicates different immune cells calculated by the CIBERSORT algorithm, and the x-axis of the heatmap indicates clinical characteristics and mutation status. Red indicates upregulation, while blue indicates downregulation. [file 12935_2022_2588_MOESM7_ESM.pdf]

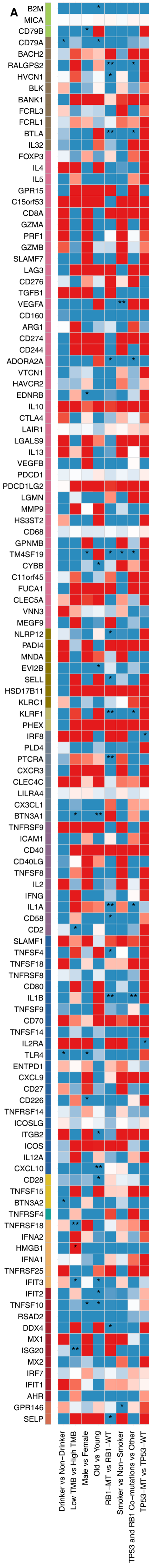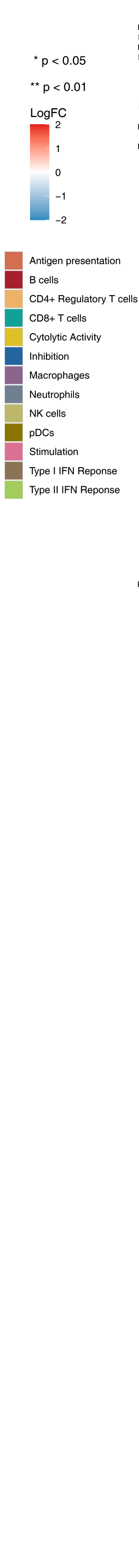

Supplement: Supplementary file 8 — Additional file 8: Figure S8. Related to Fig. 6b; heatmap depicting the mean differences in the contents of immune-related gene mRNA expression between different clinical characteristics and mutation statuses in the East Asian (a) and Caucasian (b) cohorts. The y-axis indicates immune-related gene mRNA expression, and the x-axis of the heatmap indicates clinical characteristics and mutation status. Red indicates upregulation, while blue indicates downregulation. [file 12935_2022_2588_MOESM8_ESM.pdf]
