# Supplementary material for: Genomic and immunological profiles of small-cell lung cancer between East Asians and Caucasian
Source: Cancer Cell Int. 2022 Apr 29;22:173. doi: 10.1186/s12935-022-02588-w (PMC9052616; doi:10.1186/s12935-022-02588-w)
Supplement: Supplementary file 10 — Additional file 10: Table S1. Related to Additional file 1: Fig. S1a. The results of co-occurrence/mutual exclusivity of oncogenes/TSGs in the East Asian cohort (Top20 mutated genes). [file 12935_2022_2588_MOESM10_ESM.pdf]

Supplementary Table.1 Related to Supplementary Fig. 1a The results of co-occurrence/mutual exclusivity of oncogenes/TSGs in the EA cohort (Top20 mutated genes).

gene1	gene2	pValue	oddsRatio	00	11	01	10	Event	pair	event_ratio
KMT2D	OBSCN	8.49876E-07	9.824415208	51	25	14	9	Co_Occurence	KMT2D, OBSCN	25/23
CSMD1	MACF1	3.40775E-06	8.749043912	54	22	11	12	Co_Occurence	CSMD1, MACF1	22/23
OBSCN	MACF1	2.24193E-05	7.019181728	50	23	10	16	Co_Occurence	MACF1, OBSCN	23/26
NEB	MUC16	2.59751E-05	7.218846114	36	33	23	7	Co_Occurence	MUC16, NEB	33/30
KMT2D	DST	4.92594E-05	6.526540499	51	22	14	12	Co_Occurence	DST, KMT2D	22/26
MACF1	SYNE1	5.05413E-05	6.353913251	52	21	14	12	Co_Occurence	MACF1, SYNE1	21/26
MACF1	NEB	6.3288E-05	6.482421706	49	23	17	10	Co_Occurence	MACF1, NEB	23/27
MUC5B	MACF1	8.77076E-05	6.133634101	53	20	13	13	Co_Occurence	MACF1, MUC5B	20/26
SYNE1	OBSCN	0.000102677	5.633472909	48	23	16	12	Co_Occurence	OBSCN, SYNE1	23/28
MUC16	SYNE1	0.000108784	6.494174412	37	29	6	27	Co_Occurence	MUC16, SYNE1	29/33
USH2A	OBSCN	0.000179285	5.152420636	42	27	12	18	Co_Occurence	OBSCN, USH2A	27/30
KMT2D	MACF1	0.000238958	5.59645448	52	20	13	14	Co_Occurence	KMT2D, MACF1	20/27
ZFHX4	CSMD1	0.000243138	5.298160256	45	24	10	20	Co_Occurence	CSMD1, ZFHX4	24/30
ZFHX4	KMT2D	0.000243138	5.298160256	45	24	10	20	Co_Occurence	KMT2D, ZFHX4	24/30
DST	OBSCN	0.000245895	5.099216017	47	23	16	13	Co_Occurence	DST, OBSCN	23/29
HMCN1	OBSCN	0.000245895	5.099216017	47	23	16	13	Co_Occurence	HMCN1, OBSCN	23/29
KMT2D	CSMD1	0.000326424	5.104098851	51	20	14	14	Co_Occurence	CSMD1, KMT2D	20/28
HMCN1	KMT2D	0.000333629	5.27919529	50	21	13	15	Co_Occurence	HMCN1, KMT2D	21/28
OBSCN	MUC16	0.000352812	5.328042497	35	31	25	8	Co_Occurence	MUC16, OBSCN	31/33
HMCN1	LRP2	0.000443974	4.810713579	49	21	14	15	Co_Occurence	HMCN1, LRP2	21/29
UNC80	HMCN1	0.000448758	4.935572407	48	22	14	15	Co_Occurence	HMCN1, UNC80	22/29
MUC16	RYR2	0.00050561	4.572447289	31	36	12	20	Co_Occurence	MUC16, RYR2	36/32
KMT2D	NEB	0.000524876	4.702527013	47	22	18	12	Co_Occurence	KMT2D, NEB	22/30
MACF1	ZFHX4	0.000525139	4.842280411	45	23	21	10	Co_Occurence	MACF1, ZFHX4	23/31
NEB	DST	0.000561546	4.702806222	46	23	13	17	Co_Occurence	DST, NEB	23/30
LRP1B	OBSCN	0.000568698	4.597234625	47	22	17	13	Co_Occurence	LRP1B, OBSCN	22/30
LRP2	OBSCN	0.000568698	4.597234625	47	22	17	13	Co_Occurence	LRP2, OBSCN	22/30
MACF1	HMCN1	0.000737023	4.721057333	50	20	16	13	Co_Occurence	HMCN1, MACF1	20/29
KMT2D	SYNE1	0.000754216	4.676580227	50	20	15	14	Co_Occurence	KMT2D, SYNE1	20/29
OBSCN	NEB	0.000768944	4.327409029	44	24	16	15	Co_Occurence	NEB, OBSCN	24/31
NEB	ZFHX4	0.000951	4.163172368	41	26	18	14	Co_Occurence	NEB, ZFHX4	26/32
HMCN1	DST	0.000992524	4.403991927	48	21	15	15	Co_Occurence	DST, HMCN1	21/30
UNC80	MACF1	0.000994495	4.359635512	49	20	13	17	Co_Occurence	MACF1, UNC80	20/30
TTN	MUC16	0.000994624	5.928778372	16	51	5	27	Co_Occurence	MUC16, TTN	51/32
SYNE1	LRP1B	0.001064586	4.282955851	49	20	15	15	Co_Occurence	LRP1B, SYNE1	20/30
NEB	LRP1B	0.001194325	4.254206697	46	22	13	18	Co_Occurence	LRP1B, NEB	22/31

TTN	CSMD3	0.001219582	6.875534159	18	42	3	36	Co_Occurence	CSMD3, TTN	42/39	
MACF1	TTN	0.001347827	13.65701646	20	32	46	1	Co_Occurence	MACF1, TTN	32/47	
ZFHX4	DST	0.001434495	4.231728445	43	24	12	20	Co_Occurence	DST, ZFHX4	24/32	
USH2A	KMT2D	0.001591614	4.02431747	43	23	11	22	Co_Occurence	KMT2D, USH2A	23/33	
MACF1	LRP1B	0.001672682	4.171584839	50	19	16	14	Co_Occurence	LRP1B, MACF1	19/30	
MUC5B	SYNE1	0.001672682	4.171584839	50	19	16	14	Co_Occurence	MUC5B, SYNE1	19/30	
OBSCN	TTN	0.001970431	8.421811731	19	37	41	2	Co_Occurence	OBSCN, TTN	37/43	
UNC80	TTN	0.002405048	7.604334084	19	35	43	2	Co_Occurence	TTN, UNC80	35/45	
ZFHX4	RYR2	0.002450695	3.611835418	36	29	19	15	Co_Occurence	RYR2, ZFHX4	29/34	
NEB	HMCN1	0.002624296	3.869505595	45	22	14	18	Co_Occurence	HMCN1, NEB	22/32	
MUC5B	DST	0.003566591	3.852033413	49	19	17	14	Co_Occurence	DST, MUC5B	19/31	
TTN	HMCN1	0.004284665	7.222939503	19	34	2	44	Co_Occurence	HMCN1, TTN	34/46	
UNC80	LRP1B	0.004399389	3.633250797	47	20	15	17	Co_Occurence	LRP1B, UNC80	20/32	
ZFHX4	MUC16	0.004478273	3.399721954	31	32	24	12	Co_Occurence	MUC16, ZFHX4	32/36	
CSMD1	DST	0.004533183	3.526276965	48	19	17	15	Co_Occurence	CSMD1, DST	19/32	
SYNE1	LRP2	0.00459002	3.512413674	48	19	16	16	Co_Occurence	LRP2, SYNE1	19/32	
CSMD1	OBSCN	0.005268683	3.411620818	46	20	19	14	Co_Occurence	CSMD1, OBSCN	20/33	
ZFHX4	HMCN1	0.005885121	3.490266363	42	23	13	21	Co_Occurence	HMCN1, ZFHX4	23/34	
MUC16	HMCN1	0.006265379	3.472087846	34	27	9	29	Co_Occurence	HMCN1, MUC16	27/38	
MACF1	LRP2	0.007178128	3.411612461	49	18	17	15	Co_Occurence	LRP2, MACF1	18/32	
NEB	USH2A	0.007324993	3.209093481	39	25	20	15	Co_Occurence	NEB, USH2A	25/35	
OBSCN	ZFHX4	0.007363283	3.160228276	40	24	20	15	Co_Occurence	OBSCN, ZFHX4	24/35	
MACF1	MUC16	0.009426667	3.483114653	35	25	31	8	Co_Occurence	MACF1, MUC16	25/39	
CSMD1	NEB	0.009602547	3.173679581	45	20	20	14	Co_Occurence	CSMD1, NEB	20/34	
MUC5B	ZFHX4	0.0097334	3.230369769	43	21	23	12	Co_Occurence	MUC5B, ZFHX4	21/35	
UNC80	OBSCN	0.010281703	3.167811425	44	21	18	16	Co_Occurence	OBSCN, UNC80	21/34	
ZFHX4	LRP1B	0.010639653	3.190321628	42	22	13	22	Co_Occurence	LRP1B, ZFHX4	22/35	
ZFHX4	SYNE1	0.010639653	3.190321628	42	22	13	22	Co_Occurence	SYNE1, ZFHX4	22/35	
LRP2	MUC16	0.010939547	3.234676116	34	26	30	9	Co_Occurence	LRP2, MUC16	26/39	
MUC16	UNC80	0.012669574	3.037229926	33	27	10	29	Co_Occurence	MUC16, UNC80	27/39	
RYR2	TTN	0.013778491	3.878588548	16	43	35	5	Co_Occurence	RYR2, TTN	43/40	
MACF1	DST	0.014008706	3.159147565	48	18	18	15	Co_Occurence	DST, MACF1	18/33	
CSMD1	LRP1B	0.014141934	3.136126457	48	18	17	16	Co_Occurence	CSMD1, LRP1B	18/33	
CSMD1	SYNE1	0.014141934	3.136126457	48	18	17	16	Co_Occurence	CSMD1, SYNE1	18/33	
MUC5B	OBSCN	0.015660141	3.082954867	46	19	20	14	Co_Occurence	MUC5B, OBSCN	19/34	
USH2A	RYR2	0.015808869	2.769673541	34	28	20	17	Co_Occurence	RYR2, USH2A	28/37	
UNC80	LRP2	0.016242905	2.998193519	46	19	16	18	Co_Occurence	LRP2, UNC80	19/34	
UNC80	SYNE1	0.016242905	2.998193519	46	19	16	18	Co_Occurence	SYNE1, UNC80	19/34	
MUC5B	NEB	0.017501109	2.874743603	45	19	21	14	Co_Occurence	MUC5B, NEB	19/35	

USH2A	MACF1	0.01782905	3.026248188	42	21	12	24	Co_Occurence	MACF1, USH2A	21/36	
LRP2	NEB	0.018051779	2.899529707	44	20	20	15	Co_Occurence	LRP2, NEB	20/35	
SYNE1	NEB	0.018051779	2.899529707	44	20	20	15	Co_Occurence	NEB, SYNE1	20/35	
MUC16	CSMD1	0.018661561	3.012341933	34	25	9	31	Co_Occurence	CSMD1, MUC16	25/40	
RB1	USH2A	0.019640525	2.983198094	25	35	10	29	Co_Occurence	RB1, USH2A	35/39	
CSMD3	CSMD1	0.021134316	2.730186607	41	21	13	24	Co_Occurence	CSMD1, CSMD3	21/37	
MUC16	DST	0.021161988	2.829504318	33	26	10	30	Co_Occurence	DST, MUC16	26/40	
TTN	DST	0.021291442	4.344005861	18	33	3	45	Co_Occurence	DST, TTN	33/48	
RYR2	KMT2D	0.021862379	2.721081613	39	22	12	26	Co_Occurence	KMT2D, RYR2	22/38	
RB1	TTN	0.023031256	3.147473143	12	55	23	9	Co_Occurence	RB1, TTN	55/32	
DST	RYR2	0.023284566	2.661542057	38	23	25	13	Co_Occurence	DST, RYR2	23/38	
MUC5B	LRP1B	0.025365324	2.801307355	48	17	18	16	Co_Occurence	LRP1B, MUC5B	17/34	
MUC5B	LRP2	0.025365324	2.801307355	48	17	18	16	Co_Occurence	LRP2, MUC5B	17/34	
NEB	RYR2	0.025520724	2.582621173	36	25	23	15	Co_Occurence	NEB, RYR2	25/38	
USH2A	MUC16	0.027049454	2.543545068	29	31	25	14	Co_Occurence	MUC16, USH2A	31/39	
USH2A	CSMD3	0.02782803	2.496443057	35	26	19	19	Co_Occurence	CSMD3, USH2A	26/38	
USH2A	TTN	0.028342306	3.329002376	16	40	38	5	Co_Occurence	TTN, USH2A	40/43	
UNC80	CSMD1	0.028630055	2.694331402	46	18	16	19	Co_Occurence	CSMD1, UNC80	18/35	
UNC80	KMT2D	0.028630055	2.694331402	46	18	16	19	Co_Occurence	KMT2D, UNC80	18/35	
HMCN1	LRP1B	0.028953118	2.676816383	46	18	17	18	Co_Occurence	HMCN1, LRP1B	18/35	
DST	SYNE1	0.028953118	2.676816383	46	18	17	18	Co_Occurence	DST, SYNE1	18/35	
HMCN1	SYNE1	0.028953118	2.676816383	46	18	17	18	Co_Occurence	HMCN1, SYNE1	18/35	
MUC5B	MUC16	0.031059573	2.803688497	34	24	32	9	Co_Occurence	MUC16, MUC5B	24/41	
LRP1B	MUC16	0.034534776	2.634982416	33	25	31	10	Co_Occurence	LRP1B, MUC16	25/41	
LRP2	CSMD3	0.036846242	2.476079148	40	21	24	14	Co_Occurence	CSMD3, LRP2	21/38	
SYNE1	USH2A	0.036846242	2.476079148	40	21	24	14	Co_Occurence	SYNE1, USH2A	21/38	
SYNE1	TTN	0.037645176	4.122861496	18	32	46	3	Co_Occurence	SYNE1, TTN	32/49	
CSMD1	TTN	0.037877562	3.910035155	18	31	47	3	Co_Occurence	CSMD1, TTN	31/50	
KMT2D	TTN	0.037877562	3.910035155	18	31	47	3	Co_Occurence	KMT2D, TTN	31/50	
OBSCN	CSMD3	0.039126914	2.459366077	38	23	22	16	Co_Occurence	CSMD3, OBSCN	23/38	
KMT2D	MUC5B	0.044860055	2.485034574	48	16	17	18	Co_Occurence	KMT2D, MUC5B	16/35	
LRP2	KMT2D	0.045326487	2.584172642	47	17	17	18	Co_Occurence	KMT2D, LRP2	17/35	
MACF1	RB1	0.045853104	2.710176001	28	26	38	7	Co_Occurence	MACF1, RB1	26/45	
ZFHX4	TTN	0.046790166	3.164308025	16	39	39	5	Co_Occurence	TTN, ZFHX4	39/44	
LRP2	LRP1B	0.050079833	2.390921076	46	17	18	18	Co_Occurence	LRP1B, LRP2	17/36	
RB1	OBSCN	0.052904822	2.525310421	26	30	9	34	Co_Occurence	OBSCN, RB1	30/43	
MACF1	RYR2	0.054078949	2.50354998	39	21	27	12	Co_Occurence	MACF1, RYR2	21/39	
MUC16	KMT2D	0.054995633	2.452439472	33	24	10	32	Co_Occurence	KMT2D, MUC16	24/42	
MUC16	RB1	0.056468568	2.355285632	20	41	23	15	Co_Occurence	MUC16, RB1	41/38	

CSMD3	RB1	0.056848992	2.449997645	24	34	30	11	Co_Occurence	CSMD3, RB1	34/41
CSMD3	KMT2D	0.059650309	2.2661015	40	20	14	25	Co_Occurence	CSMD3, KMT2D	20/39
DST	USH2A	0.061277533	2.255585724	39	21	24	15	Co_Occurence	DST, USH2A	21/39
ZFHX4	UNC80	0.063710022	2.207046148	39	21	16	23	Co_Occurence	UNC80, ZFHX4	21/39
USH2A	ZFHX4	0.066988631	2.282696504	35	25	19	20	Co_Occurence	USH2A, ZFHX4	25/39
CSMD3	MUC16	0.070991345	2.136938879	28	30	26	15	Co_Occurence	CSMD3, MUC16	30/41
RB1	TP53	0.077534762	2.831135377	8	58	27	6	Co_Occurence	RB1, TP53	58/33
RB1	SYNE1	0.078074316	2.44110263	27	27	8	37	Co_Occurence	RB1, SYNE1	27/45
LRP2	DST	0.081029651	2.217811608	45	17	19	18	Co_Occurence	DST, LRP2	17/37
MACF1	CSMD3	0.093245346	2.072050405	40	19	26	14	Co_Occurence	CSMD3, MACF1	19/40
MUC5B	USH2A	0.093245346	2.072050405	40	19	26	14	Co_Occurence	MUC5B, USH2A	19/40
LRP1B	CSMD3	0.095389798	2.064276867	39	20	25	15	Co_Occurence	CSMD3, LRP1B	20/40
SYNE1	CSMD3	0.095389798	2.064276867	39	20	25	15	Co_Occurence	CSMD3, SYNE1	20/40
LRP1B	USH2A	0.095389798	2.064276867	39	20	25	15	Co_Occurence	LRP1B, USH2A	20/40
LRP2	USH2A	0.095389798	2.064276867	39	20	25	15	Co_Occurence	LRP2, USH2A	20/40
NEB	UNC80	0.095482424	2.04534027	41	19	18	21	Co_Occurence	NEB, UNC80	19/39
UNC80	CSMD3	0.097427659	2.062433349	38	21	24	16	Co_Occurence	CSMD3, UNC80	21/40
UNC80	USH2A	0.097427659	2.062433349	38	21	24	16	Co_Occurence	UNC80, USH2A	21/40
SYNE1	RYR2	0.098164788	2.040379913	37	21	27	14	Co_Occurence	RYR2, SYNE1	21/41
CSMD3	RYR2	0.108625304	1.976373365	32	26	22	19	Co_Occurence	CSMD3, RYR2	26/41
MUC5B	CSMD1	0.118998914	2.045647294	47	15	19	18	Co_Occurence	CSMD1, MUC5B	15/37
KMT2D	LRP1B	0.120418445	2.134614194	46	16	19	18	Co_Occurence	KMT2D, LRP1B	16/37
LRP1B	TTN	0.121599161	2.776674084	17	31	47	4	Co_Occurence	LRP1B, TTN	31/51
RB1	MUC5B	0.122021142	2.147168552	27	25	8	39	Co_Occurence	MUC5B, RB1	25/47
RYR2	RB1	0.14046878	2.027603381	22	35	29	13	Co_Occurence	RB1, RYR2	35/42
USH2A	CSMD1	0.143468317	1.887485127	39	19	15	26	Co_Occurence	CSMD1, USH2A	19/41
ZFHX4	RB1	0.145264386	1.904124084	23	32	32	12	Co_Occurence	RB1, ZFHX4	32/44
HMCN1	USH2A	0.145999974	1.887483511	38	20	25	16	Co_Occurence	HMCN1, USH2A	20/41
HMCN1	RYR2	0.150134067	1.854784252	36	21	27	15	Co_Occurence	HMCN1, RYR2	21/42
MUC5B	TTN	0.19113188	2.494077153	17	29	49	4	Co_Occurence	MUC5B, TTN	29/53
MUC5B	HMCN1	0.193012292	1.774959967	45	15	21	18	Co_Occurence	HMCN1, MUC5B	15/39
KMT2D	RB1	0.194298623	1.840595109	26	25	39	9	Co_Occurence	KMT2D, RB1	25/48
NEB	RB1	0.204024629	1.797073846	24	29	35	11	Co_Occurence	NEB, RB1	29/46
MUC5B	RYR2	0.210400715	1.721880499	37	19	29	14	Co_Occurence	MUC5B, RYR2	19/43
OBSCN	RYR2	0.223179145	1.68326971	34	22	26	17	Co_Occurence	OBSCN, RYR2	22/43
CSMD1	LRP2	0.26819277	1.765715592	45	15	20	19	Co_Occurence	CSMD1, LRP2	15/39
UNC80	MUC5B	0.274804761	1.657896314	44	15	18	22	Co_Occurence	MUC5B, UNC80	15/40
CSMD1	HMCN1	0.276445341	1.645561346	44	15	21	19	Co_Occurence	CSMD1, HMCN1	15/40
DST	CSMD3	0.299512634	1.582918537	37	19	26	17	Co_Occurence	CSMD3, DST	19/43

CSMD1	RYR2	0.299645943	1.565144834	36	19	29	15	Co_Occurence	CSMD1, RYR2	19/44
LRP2	TTN	0.30454419	1.98689183	16	30	48	5	Co_Occurence	LRP2, TTN	30/53
CSMD3	NEB	0.305063235	1.603942226	35	21	19	24	Co_Occurence	CSMD3, NEB	21/43
TP53	DST	0.368578399	0.521495459	7	29	7	56	lutually_Exclusiv	DST, TP53	29/63
TP53	HMCN1	0.368578399	0.521495459	7	29	7	56	lutually_Exclusiv	HMCN1, TP53	29/63
TP53	UNC80	0.373584388	0.548950033	7	30	7	55	lutually_Exclusiv	TP53, UNC80	30/62
LRP2	RB1	0.380441063	1.595055387	25	25	39	10	Co_Occurence	LRP2, RB1	25/49
LRP1B	DST	0.384001305	1.528864869	43	15	21	20	Co_Occurence	DST, LRP1B	15/41
TP53	OBSCN	0.393155801	0.606987319	7	32	7	53	lutually_Exclusiv	OBSCN, TP53	32/60
LRP1B	RYR2	0.409334151	1.427933442	35	19	29	16	Co_Occurence	LRP1B, RYR2	19/45
LRP2	RYR2	0.409334151	1.427933442	35	19	29	16	Co_Occurence	LRP2, RYR2	19/45
ZFHX4	CSMD3	0.426083991	1.386615304	32	22	23	22	Co_Occurence	CSMD3, ZFHX4	22/45
TP53	TTN	0.487743338	1.591679935	4	68	10	17	Co_Occurence	TP53, TTN	68/27
CSMD1	RB1	0.50700651	1.493954397	25	24	40	10	Co_Occurence	CSMD1, RB1	24/50
DST	RB1	0.516230033	1.393913867	24	25	39	11	Co_Occurence	DST, RB1	25/50
HMCN1	RB1	0.516230033	1.393913867	24	25	39	11	Co_Occurence	HMCN1, RB1	25/50
DST	UNC80	0.524410553	1.327252201	41	15	22	21	Co_Occurence	DST, UNC80	15/43
HMCN1	CSMD3	0.533870912	1.32942055	36	18	27	18	Co_Occurence	CSMD3, HMCN1	18/45
MUC5B	TP53	0.541637981	0.623832867	8	27	58	6	lutually_Exclusiv	MUC5B, TP53	27/64
CSMD1	TP53	0.547459602	0.657880973	8	28	57	6	lutually_Exclusiv	CSMD1, TP53	28/63
TP53	LRP2	0.556017095	0.693130698	8	29	6	56	lutually_Exclusiv	LRP2, TP53	29/62
TP53	SYNE1	0.556017095	0.693130698	8	29	6	56	lutually_Exclusiv	SYNE1, TP53	29/62
TP53	NEB	0.558435006	0.637638895	7	33	7	52	lutually_Exclusiv	NEB, TP53	33/59
USH2A	TP53	0.56549796	1.592568224	9	40	45	5	Co_Occurence	TP53, USH2A	40/50
NEB	TTN	0.61726438	1.461120265	14	33	45	7	Co_Occurence	NEB, TTN	33/52
LRP1B	RB1	0.661345764	1.305565465	24	24	40	11	Co_Occurence	LRP1B, RB1	24/51
LRP2	ZFHX4	0.672609854	1.29083407	37	17	27	18	Co_Occurence	LRP2, ZFHX4	17/45
MUC5B	CSMD3	0.675489393	1.19857917	37	16	29	17	Co_Occurence	CSMD3, MUC5B	16/46
RYR2	UNC80	0.682856762	1.198918026	33	19	18	29	Co_Occurence	RYR2, UNC80	19/47
MUC16	TP53	0.772110496	1.356806345	7	49	36	7	Co_Occurence	MUC16, TP53	49/43
ZFHX4	TP53	0.773823951	0.772888025	7	37	48	7	lutually_Exclusiv	TP53, ZFHX4	37/55
CSMD3	TP53	0.776987252	0.81026322	7	38	47	7	lutually_Exclusiv	CSMD3, TP53	38/54
UNC80	RB1	0.828256033	0.842948639	21	23	41	14	lutually_Exclusiv	RB1, UNC80	23/55
TP53	RYR2	1	0.932502131	7	41	7	44	lutually_Exclusiv	RYR2, TP53	41/51
LRP1B	TP53	1	0.981992187	9	30	55	5	lutually_Exclusiv	LRP1B, TP53	30/60
KMT2D	TP53	1	0.932810928	9	29	56	5	lutually_Exclusiv	KMT2D, TP53	29/61
MACF1	TP53	1	0.885331096	9	28	57	5	lutually_Exclusiv	MACF1, TP53	28/62
