# Supplementary material for: Genomic and immunological profiles of small-cell lung cancer between East Asians and Caucasian
Source: Cancer Cell Int. 2022 Apr 29;22:173. doi: 10.1186/s12935-022-02588-w (PMC9052616; doi:10.1186/s12935-022-02588-w)
Supplement: Supplementary file 11 — Additional file 11: Table S2. Related to Additional file 1: Fig. S1b. The results of the co-occurrence/mutual exclusivity of oncogenes/TSGs in the Caucasian cohort (Top20 mutated genes). [file 12935_2022_2588_MOESM11_ESM.pdf]

Supplementary Table.2 Related to Supplementary Fig. 1b The results of the co-occurrence/mutual exclusivity of oncogenes/TSGs in the Caucasian cohort (Top20 mutated genes).

gene1	gene2	pValue	oddsRatio	00	11	01	10	Event	pair	event_ratio
NAV3	RYR2	0.010204325	6.122955167	21	11	9	4	Co Occurence	NAV3, RYR2	11/13
MLL2	USH2A	0.014003912	5.953635673	25	8	8	4	Co Occurence	MLL2, USH2A	8/12
FMN2	RYR2	0.018790252	5.745656248	22	9	11	3	Co Occurence	FMN2, RYR2	9/14
MUC16	FAM135B	0.023042895	5.337533788	20	10	3	12	Co Occurence	FAM135B, MUC16	10/15
SPTA1	EYS	0.028797624	4.975872919	26	7	7	5	Co Occurence	EYS, SPTA1	7/12
KIAA1211	USH2A	0.033251346	0.113584343	18	1	15	11	Mutually Exclusive	KIAA1211, USH2A	1/26
RYR3	ZFHX4	0.040975951	4.429558567	23	8	10	4	Co Occurence	RYR3, ZFHX4	8/14
ZFHX4	EYS	0.046944668	4.241930041	22	9	5	9	Co Occurence	EYS, ZFHX4	9/14
FMN2	TTN	0.055204104	0.231356236	6	6	27	6	Mutually Exclusive	FMN2, TTN	6/33
USH2A	MUC16	0.065387177	3.49360658	18	11	11	5	Co Occurence	MUC16, USH2A	11/16
TTN	EYS	0.070014993	6.904254633	11	13	1	20	Co Occurence	EYS, TTN	13/21
COL11A1	EYS	0.072415266	4.016187181	25	7	7	6	Co Occurence	COL11A1, EYS	7/13
RYR3	COL11A1	0.075672781	3.590673798	26	6	7	6	Co Occurence	COL11A1, RYR3	6/13
SPTA1	FAM135B	0.075672781	3.590673798	26	6	7	6	Co Occurence	FAM135B, SPTA1	6/13
FAM135B	ZFHX4	0.093872868	3.414487703	22	8	10	5	Co Occurence	FAM135B, ZFHX4	8/15
COL11A1	RYR2	0.099796255	0.272529404	15	3	17	10	Mutually Exclusive	COL11A1, RYR2	3/27
CSMD3	EYS	0.107057994	3.182512788	20	9	5	11	Co Occurence	CSMD3, EYS	9/16
TP53	RB1	0.135888502	4.322464009	3	30	2	10	Co Occurence	RB1, TP53	30/12
SPTA1	TTN	0.135956499	5.337192976	11	11	22	1	Co Occurence	SPTA1, TTN	11/23
FAM135B	COL11A1	0.149300382	2.97675975	25	6	7	7	Co Occurence	COL11A1, FAM135B	6/14
MLL2	NAV3	0.173601812	2.603998848	24	6	9	6	Co Occurence	MLL2, NAV3	6/15
KIAA1211	ZFHX4	0.175186667	2.732541136	22	7	11	5	Co Occurence	KIAA1211, ZFHX4	7/16
SPTA1	ZFHX4	0.175186667	2.732541136	22	7	11	5	Co Occurence	SPTA1, ZFHX4	7/16
CSMD3	SI	0.175707094	2.759894916	21	7	4	13	Co Occurence	CSMD3, SI	7/17
RYR2	SI	0.175707094	2.759894916	21	7	4	13	Co Occurence	RYR2, SI	7/17
TTN	CSMD3	0.176772623	3.108188933	9	17	3	16	Co Occurence	CSMD3, TTN	17/19
MUC16	RB1	0.189388481	2.824531981	9	18	14	4	Co Occurence	MUC16, RB1	18/18
KIAA1211	LRP1B	0.189388481	2.653712697	19	8	14	4	Co Occurence	KIAA1211, LRP1B	8/18
FMN2	MUC16	0.189388481	2.653712697	19	8	14	4	Co Occurence	FMN2, MUC16	8/18
RYR3	MUC16	0.189388481	2.653712697	19	8	14	4	Co Occurence	MUC16, RYR3	8/18
FAM135B	CSMD3	0.191470459	2.606771382	20	8	12	5	Co Occurence	CSMD3, FAM135B	8/17
RYR2	EYS	0.201964301	0.383227557	15	4	10	16	Mutually Exclusive	EYS, RYR2	4/26
NAV3	LRP1B	0.20781147	0.390775243	13	5	17	10	Mutually Exclusive	LRP1B, NAV3	5/27
EYS	MUC16	0.20781147	2.441123209	18	9	13	5	Co Occurence	EYS, MUC16	9/18
NAV3	ZFHX4	0.217566187	2.242172714	20	8	10	7	Co Occurence	NAV3, ZFHX4	8/17
SI	RYR3	0.239984849	0.214951297	23	1	11	10	Mutually Exclusive	RYR3, SI	1/21

ZFHX4	CSMD3	0.241204721	2.088682498	17	10	10	8	Co Occurence	CSMD3, ZFHX4	10/18
SI	RB1	0.251016659	0.378517584	8	6	26	5	Mutually Exclusive	RB1, SI	6/31
MLL2	FMN2	0.253601126	2.58946096	26	5	7	7	Co Occurence	FMN2, MLL2	5/14
SPTA1	RYR3	0.253601126	2.58946096	26	5	7	7	Co Occurence	RYR3, SPTA1	5/14
PKHD1L1	TTN	0.284614727	0.456788486	7	8	25	5	Mutually Exclusive	PKHD1L1, TTN	8/30
SPTA1	COL11A1	0.284614727	2.189197037	25	5	8	7	Co Occurence	COL11A1, SPTA1	5/15
FMN2	FAM135B	0.284614727	2.189197037	25	5	8	7	Co Occurence	FAM135B, FMN2	5/15
KIAA1211	FAM135B	0.284614727	2.189197037	25	5	8	7	Co Occurence	FAM135B, KIAA121	5/15
RYR3	FAM135B	0.284614727	2.189197037	25	5	8	7	Co Occurence	FAM135B, RYR3	5/15
FMN2	PKHD1L1	0.284614727	2.189197037	25	5	8	7	Co Occurence	FMN2, PKHD1L1	5/15
PKHD1L1	RB1	0.28715043	2.82024253	11	11	21	2	Co Occurence	PKHD1L1, RB1	11/23
EYS	FMN2	0.287229671	0.357485248	21	2	10	12	Mutually Exclusive	EYS, FMN2	2/22
EYS	MLL2	0.287229671	0.357485248	21	2	10	12	Mutually Exclusive	EYS, MLL2	2/22
USH2A	RYR3	0.296328983	2.254988641	23	6	6	10	Co Occurence	RYR3, USH2A	6/16
TP53	FAM135B	0.300713152	Inf	5	13	0	27	Co Occurence	FAM135B, TP53	13/27
KIAA1211	TP53	0.30335197	Inf	5	12	28	0	Co Occurence	KIAA1211, TP53	12/28
MLL2	TP53	0.30335197	Inf	5	12	28	0	Co Occurence	MLL2, TP53	12/28
SI	LRP1B	0.314097945	2.177476326	19	7	15	4	Co Occurence	LRP1B, SI	7/19
RYR2	TTN	0.319979663	0.472496742	5	13	20	7	Mutually Exclusive	RYR2, TTN	13/27
SPTA1	CSMD3	0.319979663	2.116416708	20	7	13	5	Co Occurence	CSMD3, SPTA1	7/18
KIAA1211	RYR2	0.319979663	2.116416708	20	7	13	5	Co Occurence	KIAA1211, RYR2	7/18
MLL2	RYR2	0.319979663	2.116416708	20	7	13	5	Co Occurence	MLL2, RYR2	7/18
TP53	USH2A	0.33006755	0.329946106	2	13	3	27	Mutually Exclusive	TP53, USH2A	13/30
FAM135B	LRP1B	0.336772393	2.024036807	18	8	14	5	Co Occurence	FAM135B, LRP1B	8/19
LRP1B	TP53	0.34625323	4.29064138	4	21	19	1	Co Occurence	LRP1B, TP53	21/20
CSMD3	TP53	0.362394711	3.528706701	4	19	21	1	Co Occurence	CSMD3, TP53	19/22
SI	KIAA1211	0.448313875	1.829850474	26	4	8	7	Co Occurence	KIAA1211, SI	4/15
COL11A1	TTN	0.459578249	2.454210311	10	11	22	2	Co Occurence	COL11A1, TTN	11/24
FMN2	COL11A1	0.459578249	0.407463042	22	2	11	10	Mutually Exclusive	COL11A1, FMN2	2/21
RYR3	PKHD1L1	0.459578249	0.407463042	22	2	11	10	Mutually Exclusive	PKHD1L1, RYR3	2/21
SPTA1	PKHD1L1	0.459578249	0.407463042	22	2	11	10	Mutually Exclusive	PKHD1L1, SPTA1	2/21
RB1	FMN2	0.459578249	2.454210311	11	10	2	22	Co Occurence	FMN2, RB1	10/24
RB1	RYR3	0.459578249	2.454210311	11	10	2	22	Co Occurence	RB1, RYR3	10/24
KIAA1211	TTN	0.466015034	2.139932101	10	10	23	2	Co Occurence	KIAA1211, TTN	10/25
MLL2	TTN	0.466015034	2.139932101	10	10	23	2	Co Occurence	MLL2, TTN	10/25
MLL2	KIAA1211	0.466015034	0.467304547	23	2	10	10	Mutually Exclusive	KIAA1211, MLL2	2/20
RYR3	KIAA1211	0.466015034	0.467304547	23	2	10	10	Mutually Exclusive	KIAA1211, RYR3	2/20
EYS	KIAA1211	0.470275747	1.876019598	24	5	7	9	Co Occurence	EYS, KIAA1211	5/16
EYS	RYR3	0.470275747	1.876019598	24	5	7	9	Co Occurence	EYS, RYR3	5/16

PKHD1L1	COL11A1	0.472573728	1.847410393	24	5	8	8	Co Occurence	COL11A1, PKHD1L	5/16	
COL11A1	NAV3	0.491747428	0.507395814	20	3	12	10	Mutually Exclusive	COL11A1, NAV3	3/22	
NAV3	FMN2	0.495862833	1.624142884	23	5	7	10	Co Occurence	FMN2, NAV3	5/17	
NAV3	KIAA1211	0.495862833	1.624142884	23	5	7	10	Co Occurence	KIAA1211, NAV3	5/17	
NAV3	RYR3	0.495862833	1.624142884	23	5	7	10	Co Occurence	NAV3, RYR3	5/17	
NAV3	SPTA1	0.495862833	1.624142884	23	5	7	10	Co Occurence	NAV3, SPTA1	5/17	
FMN2	CSMD3	0.50218505	0.538679975	17	4	16	8	Mutually Exclusive	CSMD3, FMN2	4/24	
RYR3	RYR2	0.50218505	0.538679975	17	4	16	8	Mutually Exclusive	RYR2, RYR3	4/24	
SPTA1	RYR2	0.50218505	0.538679975	17	4	16	8	Mutually Exclusive	RYR2, SPTA1	4/24	
FAM135B	EYS	0.502447482	1.580062406	23	5	9	8	Co Occurence	EYS, FAM135B	5/17	
KIAA1211	MUC16	0.513633089	1.660587072	18	7	15	5	Co Occurence	KIAA1211, MUC16	7/20	
SPTA1	MUC16	0.513633089	1.660587072	18	7	15	5	Co Occurence	MUC16, SPTA1	7/20	
ZFHX4	RB1	0.513644223	1.728791413	9	14	18	4	Co Occurence	RB1, ZFHX4	14/22	
PKHD1L1	ZFHX4	0.513644223	0.578438783	18	4	14	9	Mutually Exclusive	PKHD1L1, ZFHX4	4/23	
FAM135B	RYR2	0.515100547	1.684610069	19	7	13	6	Co Occurence	FAM135B, RYR2	7/19	
USH2A	ZFHX4	0.527138541	0.566649372	16	5	13	11	Mutually Exclusive	USH2A, ZFHX4	5/24	
LRP1B	ZFHX4	0.549897072	1.54691413	15	10	8	12	Co Occurence	LRP1B, ZFHX4	10/20	
CSMD3	LRP1B	0.554443567	1.540189285	14	11	11	9	Co Occurence	CSMD3, LRP1B	11/20	
ZFHX4	RYR2	0.558651327	1.442337818	16	9	11	9	Co Occurence	RYR2, ZFHX4	9/20	
TTN	TP53	0.598087675	1.965821187	2	30	10	3	Co Occurence	TP53, TTN	30/13	
TP53	ZFHX4	0.633704356	2.894430592	4	17	1	23	Co Occurence	TP53, ZFHX4	17/24	
RYR2	TP53	0.642318984	0.500658309	2	17	23	3	Mutually Exclusive	RYR2, TP53	17/26	
NAV3	TP53	0.651076849	2.12157277	4	14	26	1	Co Occurence	NAV3, TP53	14/27	
TTN	SI	0.699080296	1.850990731	10	9	2	24	Co Occurence	SI, TTN	9/26	
SI	COL11A1	0.703639685	1.570266153	25	4	9	7	Co Occurence	COL11A1, SI	4/16	
SI	FAM135B	0.703639685	1.570266153	25	4	9	7	Co Occurence	FAM135B, SI	4/16	
RYR3	FMN2	0.704980682	1.546312141	25	4	8	8	Co Occurence	FMN2, RYR3	4/16	
SPTA1	FMN2	0.704980682	1.546312141	25	4	8	8	Co Occurence	FMN2, SPTA1	4/16	
SPTA1	KIAA1211	0.704980682	1.546312141	25	4	8	8	Co Occurence	KIAA1211, SPTA1	4/16	
SPTA1	MLL2	0.704980682	1.546312141	25	4	8	8	Co Occurence	MLL2, SPTA1	4/16	
EYS	SI	0.717110609	1.361583867	24	4	7	10	Co Occurence	EYS, SI	4/17	
KIAA1211	RB1	0.721072645	0.754923996	9	8	24	4	Mutually Exclusive	KIAA1211, RB1	8/28	
MLL2	RB1	0.721072645	0.754923996	9	8	24	4	Mutually Exclusive	MLL2, RB1	8/28	
SPTA1	RB1	0.721072645	0.754923996	9	8	24	4	Mutually Exclusive	RB1, SPTA1	8/28	
KIAA1211	COL11A1	0.721072645	1.324636659	24	4	9	8	Co Occurence	COL11A1, KIAA121	4/17	
MLL2	COL11A1	0.721072645	1.324636659	24	4	9	8	Co Occurence	COL11A1, MLL2	4/17	
MLL2	FAM135B	0.721072645	1.324636659	24	4	9	8	Co Occurence	FAM135B, MLL2	4/17	
KIAA1211	PKHD1L1	0.721072645	1.324636659	24	4	9	8	Co Occurence	KIAA1211, PKHD1L	4/17	
FAM135B	RB1	0.725298817	1.50173756	10	10	22	3	Co Occurence	FAM135B, RB1	10/25	

SI	NAV3	0.726236073	0.693062698	22	3	12	8	Mutually Exclusive	NAV3, SI	3/20
FMN2	USH2A	0.728383241	1.416944804	22	5	11	7	Co Occurence	FMN2, USH2A	5/18
SPTA1	USH2A	0.728383241	1.416944804	22	5	11	7	Co Occurence	SPTA1, USH2A	5/18
SI	ZFHX4	0.732294357	1.337120048	21	5	13	6	Co Occurence	SI, ZFHX4	5/19
FMN2	ZFHX4	0.735443836	0.684346635	19	4	14	8	Mutually Exclusive	FMN2, ZFHX4	4/22
EYS	USH2A	0.737848673	0.63965099	19	4	12	10	Mutually Exclusive	EYS, USH2A	4/22
TTN	LRP1B	0.738110125	1.474413036	7	17	5	16	Co Occurence	LRP1B, TTN	17/21
FMN2	LRP1B	0.738110125	0.678236	16	5	17	7	Mutually Exclusive	FMN2, LRP1B	5/24
TTN	MUC16	0.738110125	1.474413036	7	17	5	16	Co Occurence	MUC16, TTN	17/21
COL11A1	ZFHX4	0.739452262	1.41705457	20	6	12	7	Co Occurence	COL11A1, ZFHX4	6/19
CSMD3	RYR3	0.74086345	1.347857809	19	6	6	14	Co Occurence	CSMD3, RYR3	6/20
EYS	NAV3	0.742529947	0.732339931	20	4	11	10	Mutually Exclusive	EYS, NAV3	4/21
USH2A	FAM135B	0.7431987	0.745611077	20	4	9	12	Mutually Exclusive	FAM135B, USH2A	4/21
CSMD3	RB1	0.744789762	1.401044309	8	15	17	5	Co Occurence	CSMD3, RB1	15/22
COL11A1	CSMD3	0.744789762	0.713753301	17	5	15	8	Mutually Exclusive	COL11A1, CSMD3	5/23
NAV3	USH2A	0.746305168	1.32466856	20	6	10	9	Co Occurence	NAV3, USH2A	6/19
LRP1B	RB1	0.749447349	0.761031766	6	15	17	7	Mutually Exclusive	LRP1B, RB1	15/24
COL11A1	LRP1B	0.749447349	1.314005597	17	7	15	6	Co Occurence	COL11A1, LRP1B	7/21
PKHD1L1	LRP1B	0.749447349	1.314005597	17	7	15	6	Co Occurence	LRP1B, PKHD1L1	7/21
COL11A1	MUC16	0.749447349	1.314005597	17	7	15	6	Co Occurence	COL11A1, MUC16	7/21
PKHD1L1	MUC16	0.749447349	1.314005597	17	7	15	6	Co Occurence	MUC16, PKHD1L1	7/21
NAV3	CSMD3	0.756881376	0.766512423	16	6	14	9	Mutually Exclusive	CSMD3, NAV3	6/23
NAV3	MUC16	0.757529692	1.298358735	16	8	14	7	Co Occurence	MUC16, NAV3	8/21
RYR2	LRP1B	0.766613297	0.759993281	12	9	13	11	Mutually Exclusive	LRP1B, RYR2	9/24
MUC16	TP53	1	1.486664985	3	20	20	2	Co Occurence	MUC16, TP53	20/22
FMN2	TP53	1	1.504356001	4	11	29	1	Co Occurence	FMN2, TP53	11/30
RYR3	TP53	1	1.504356001	4	11	29	1	Co Occurence	RYR3, TP53	11/30
SPTA1	TP53	1	1.504356001	4	11	29	1	Co Occurence	SPTA1, TP53	11/30
NAV3	RB1	1	1.174324964	9	11	21	4	Co Occurence	NAV3, RB1	11/25
EYS	RB1	1	1.022219448	9	10	22	4	Co Occurence	EYS, RB1	10/26
COL11A1	RB1	1	0.882964572	9	9	23	4	Mutually Exclusive	COL11A1, RB1	9/27
ZFHX4	MUC16	1	1.075149903	14	9	13	9	Co Occurence	MUC16, ZFHX4	9/22
TTN	ZFHX4	1	0.91191917	7	13	5	20	Mutually Exclusive	TTN, ZFHX4	13/25
MLL2	ZFHX4	1	1.096588418	20	5	13	7	Co Occurence	MLL2, ZFHX4	5/20
PKHD1L1	EYS	1	0.978263524	22	4	10	9	Mutually Exclusive	EYS, PKHD1L1	4/19
TP53	COL11A1	1	1.695802454	4	12	1	28	Co Occurence	COL11A1, TP53	12/29
TP53	PKHD1L1	1	1.695802454	4	12	1	28	Co Occurence	PKHD1L1, TP53	12/29
SI	PKHD1L1	1	0.902065602	24	3	10	8	Mutually Exclusive	PKHD1L1, SI	3/18
EYS	TP53	1	1.901025336	4	13	27	1	Co Occurence	EYS, TP53	13/28

SI	TP53		1	1.325297292	4	10	30	1	Co Occurence	SI, TP53	10/31
RB1	TTN		1	0.77108653	3	23	10	9	Mutually Exclusive	RB1, TTN	23/19
USH2A	TTN		1	1.139491989	8	12	21	4	Co Occurence	TTN, USH2A	12/25
NAV3	TTN		1	1	8	11	22	4	Mutually Exclusive	NAV3, TTN	11/26
FAM135B	TTN		1	1.296871312	9	10	23	3	Co Occurence	FAM135B, TTN	10/26
RYR3	TTN		1	1.122126929	9	9	24	3	Co Occurence	RYR3, TTN	9/27
RYR2	RB1		1	0.909374624	7	14	18	6	Mutually Exclusive	RB1, RYR2	14/24
USH2A	RB1		1	0.841436717	8	11	21	5	Mutually Exclusive	RB1, USH2A	11/26
MUC16	LRP1B		1	1.088800205	12	11	11	11	Co Occurence	LRP1B, MUC16	11/22
USH2A	LRP1B		1	1.06977476	15	8	14	8	Co Occurence	LRP1B, USH2A	8/22
EYS	LRP1B		1	1.065116411	16	7	15	7	Co Occurence	EYS, LRP1B	7/22
MLL2	LRP1B		1	1.061040662	17	6	16	6	Co Occurence	LRP1B, MLL2	6/22
RYR3	LRP1B		1	1.061040662	17	6	16	6	Co Occurence	LRP1B, RYR3	6/22
SPTA1	LRP1B		1	1.061040662	17	6	16	6	Co Occurence	LRP1B, SPTA1	6/22
CSMD3	MUC16		1	1.081406785	13	10	12	10	Co Occurence	CSMD3, MUC16	10/22
RYR2	MUC16		1	1.081406785	13	10	12	10	Co Occurence	MUC16, RYR2	10/22
MLL2	MUC16		1	1.061040662	17	6	16	6	Co Occurence	MLL2, MUC16	6/22
SI	MUC16		1	0.836715935	17	5	17	6	Mutually Exclusive	MUC16, SI	5/23
RYR2	CSMD3		1	1.040410711	14	9	11	11	Co Occurence	CSMD3, RYR2	9/22
USH2A	CSMD3		1	0.958177275	16	7	13	9	Mutually Exclusive	CSMD3, USH2A	7/22
PKHD1L1	CSMD3		1	1.099656812	18	6	14	7	Co Occurence	CSMD3, PKHD1L1	6/21
KIAA1211	CSMD3		1	0.860073958	18	5	15	7	Mutually Exclusive	CSMD3, KIAA1211	5/22
MLL2	CSMD3		1	0.860073958	18	5	15	7	Mutually Exclusive	CSMD3, MLL2	5/22
USH2A	RYR2		1	0.958177275	16	7	13	9	Mutually Exclusive	RYR2, USH2A	7/22
PKHD1L1	RYR2		1	1.099656812	18	6	14	7	Co Occurence	PKHD1L1, RYR2	6/21
COL11A1	USH2A		1	1.188443503	21	5	11	8	Co Occurence	COL11A1, USH2A	5/19
PKHD1L1	USH2A		1	1.188443503	21	5	11	8	Co Occurence	PKHD1L1, USH2A	5/19
SI	USH2A		1	1.046515022	22	4	12	7	Co Occurence	SI, USH2A	4/19
FAM135B	NAV3		1	0.851553046	21	4	11	9	Mutually Exclusive	FAM135B, NAV3	4/20
PKHD1L1	NAV3		1	0.851553046	21	4	11	9	Mutually Exclusive	NAV3, PKHD1L1	4/20
PKHD1L1	FAM135B		1	1.132548271	23	4	9	9	Co Occurence	FAM135B, PKHD1L	4/18
MLL2	PKHD1L1		1	0.77108653	23	3	10	9	Mutually Exclusive	MLL2, PKHD1L1	3/19
KIAA1211	FMN2		1	0.891164782	24	3	9	9	Mutually Exclusive	FMN2, KIAA1211	3/18
SI	FMN2		1	1.040710039	25	3	9	8	Co Occurence	FMN2, SI	3/17
RYR3	MLL2		1	0.891164782	24	3	9	9	Mutually Exclusive	MLL2, RYR3	3/18
SI	MLL2		1	1.040710039	25	3	9	8	Co Occurence	MLL2, SI	3/17
SI	SPTA1		1	1.040710039	25	3	9	8	Co Occurence	SI, SPTA1	3/17
