# Supplementary material for: Genomic and immunological profiles of small-cell lung cancer between East Asians and Caucasian
Source: Cancer Cell Int. 2022 Apr 29;22:173. doi: 10.1186/s12935-022-02588-w (PMC9052616; doi:10.1186/s12935-022-02588-w)
Supplement: Supplementary file 12 — Additional file 12: Table S3. Related to Additional file 2: Fig. S2a. The results of the co-occurrence/mutual exclusivity of oncogenes/TSGs in the East Asian cohort (significantly mutated driver genes). [file 12935_2022_2588_MOESM12_ESM.pdf]

Supplementary Table.3 Related to Supplementary Fig. 2a The results of the co-occurrence/mutual exclusivity of oncogenes/TSGs in the EA cohort (significantly mutated driver genes).

gene1	gene2	pValue	oddsRatio	00	11	01	10	Event	pair	event_rati
NSD1	ZMYM3	1.99567E-08	54.55625501	67	11	4	3	Co_Occurence	NSD1, ZMYM3	11/7
AXIN1	DCC	2.2398E-07	40.5216358	64	11	2	8	Co_Occurence	AXIN1, DCC	11/10
NF1	TRRAP	5.69945E-07	24.0674252	59	13	3	10	Co_Occurence	NF1, TRRAP	13/13
ARID1B	RNF213	9.02217E-07	16.85191958	53	16	12	4	Co_Occurence	ARID1B, RNF213	16/16
FLNA	RNF213	9.02217E-07	16.85191958	53	16	12	4	Co_Occurence	FLNA, RNF213	16/16
NSD1	AXIN1	9.39197E-07	26.89975494	63	11	8	3	Co_Occurence	AXIN1, NSD1	11/11
MYH9	AXIN1	1.47299E-06	19.6646263	61	12	7	5	Co_Occurence	AXIN1, MYH9	12/12
ZMYM3	POLE	1.48299E-06	22.61230122	60	12	10	3	Co_Occurence	POLE, ZMYM3	12/13
NSD1	FLNA	2.00119E-06	23.6890398	62	11	9	3	Co_Occurence	FLNA, NSD1	11/12
NF1	RNF213	2.41729E-06	12.6002722	51	17	11	6	Co_Occurence	NF1, RNF213	17/17
RNF213	AXIN1	3.6939E-06	14.63896448	53	15	4	13	Co_Occurence	AXIN1, RNF213	15/17
MYH9	RNF213	3.86816E-06	17.20891183	54	14	14	3	Co_Occurence	MYH9, RNF213	14/17
FLNA	BCORL1	4.08149E-06	14.633269	58	13	7	7	Co_Occurence	BCORL1, FLNA	13/14
CREBBP	SETD1B	5.15457E-06	16.79882605	59	12	4	10	Co_Occurence	CREBBP, SETD1B	12/14
FAT1	BCORL1	6.08678E-06	12.7088813	53	15	5	12	Co_Occurence	BCORL1, FAT1	15/17
FAT1	FANCA	7.51668E-06	13.92927289	54	14	4	13	Co_Occurence	FANCA, FAT1	14/17
TERT	FAT1	8.30044E-06	16.28188382	55	13	14	3	Co_Occurence	FAT1, TERT	13/17
TSC2	SETD1B	9.06245E-06	15.88636128	61	11	5	8	Co_Occurence	SETD1B, TSC2	11/13
TSC2	TRRAP	9.06245E-06	15.88636128	61	11	5	8	Co_Occurence	TRRAP, TSC2	11/13
MTOR	ARID1B	9.17744E-06	14.03652994	59	12	8	6	Co_Occurence	ARID1B, MTOR	12/14
POLD1	FANCA	1.05271E-05	15.14240521	61	11	7	6	Co_Occurence	FANCA, POLD1	11/13
RNF213	UBR5	1.2069E-05	11.5401287	52	15	5	13	Co_Occurence	RNF213, UBR5	15/18
TRRAP	TERT	1.2845E-05	16.50722701	63	10	6	6	Co_Occurence	TERT, TRRAP	10/12
TERT	BCORL1	1.87601E-05	13.96483147	60	11	9	5	Co_Occurence	BCORL1, TERT	11/14
TRRAP	BCORL1	1.87601E-05	13.96483147	60	11	9	5	Co_Occurence	BCORL1, TRRAP	11/14
TSC2	BCORL1	2.21968E-05	11.88638476	58	12	8	7	Co_Occurence	BCORL1, TSC2	12/15
AXIN1	FLNA	2.21968E-05	11.88638476	58	12	8	7	Co_Occurence	AXIN1, FLNA	12/15
DCC	MYH9	2.81551E-05	16.96597974	64	9	8	4	Co_Occurence	DCC, MYH9	9/12
SETD1B	MYH9	2.8759E-05	14.01098082	62	10	7	6	Co_Occurence	MYH9, SETD1B	10/13
TERT	MYH9	2.8759E-05	14.01098082	62	10	7	6	Co_Occurence	MYH9, TERT	10/13
NSD1	BCORL1	3.49228E-05	14.50633968	61	10	10	4	Co_Occurence	BCORL1, NSD1	10/14
RNF213	DNMT1	4.31763E-05	10.03586744	52	14	5	14	Co_Occurence	DNMT1, RNF213	14/19
ARID1B	NF1	4.38906E-05	9.829072872	55	13	10	7	Co_Occurence	ARID1B, NF1	13/17
BCORL1	NF1	4.38906E-05	9.829072872	55	13	10	7	Co_Occurence	BCORL1, NF1	13/17
UBR5	NF1	4.38906E-05	9.829072872	55	13	10	7	Co_Occurence	NF1, UBR5	13/17
RANBP2	NF1	4.49054E-05	12.72559991	58	11	12	4	Co_Occurence	NF1, RANBP2	11/16

CREBBP	FAT1	4.58434E-05	8.803304781	51	15	12	7	Co_Occurence	CREBBP, FAT1	15/19
KAT6B	BCORL1	4.68692E-05	11.50063555	59	11	9	6	Co_Occurence	BCORL1, KAT6B	11/15
MYH9	FLNA	4.68692E-05	11.50063555	59	11	9	6	Co_Occurence	FLNA, MYH9	11/15
MYH9	NCOR2	4.68692E-05	11.50063555	59	11	9	6	Co_Occurence	MYH9, NCOR2	11/15
STAG1	TSC2	4.90652E-05	12.92120419	61	10	9	5	Co_Occurence	STAG1, TSC2	10/14
BCORL1	ARID1B	4.93697E-05	10.26703314	57	12	8	8	Co_Occurence	ARID1B, BCORL1	12/16
FLNA	ARID1B	4.93697E-05	10.26703314	57	12	8	8	Co_Occurence	ARID1B, FLNA	12/16
FANCA	BIRC6	5.27922E-05	11.097709	59	11	8	7	Co_Occurence	BIRC6, FANCA	11/15
MTOR	TSC2	5.27922E-05	11.097709	59	11	8	7	Co_Occurence	MTOR, TSC2	11/15
KAT6A	FANCA	5.95923E-05	12.12390441	61	10	8	6	Co_Occurence	FANCA, KAT6A	10/14
NIN	FANCA	5.95923E-05	12.12390441	61	10	8	6	Co_Occurence	FANCA, NIN	10/14
SETD1B	FANCA	5.95923E-05	12.12390441	61	10	8	6	Co_Occurence	FANCA, SETD1B	10/14
POLD1	MYH9	6.34864E-05	11.88059761	61	10	7	7	Co_Occurence	MYH9, POLD1	10/14
SPEN	POLD1	6.34864E-05	11.88059761	61	10	7	7	Co_Occurence	POLD1, SPEN	10/14
STAG1	NIN	8.1474E-05	12.83423379	63	9	7	6	Co_Occurence	NIN, STAG1	9/13
NCOR1	SETD1B	8.1474E-05	12.83423379	63	9	7	6	Co_Occurence	NCOR1, SETD1B	9/13
STAG1	SETD1B	8.1474E-05	12.83423379	63	9	7	6	Co_Occurence	SETD1B, STAG1	9/13
BCORL1	RANBP2	9.14925E-05	11.48136225	60	10	5	10	Co_Occurence	BCORL1, RANBP2	10/15
BCORL1	ZMYM3	9.14925E-05	11.48136225	60	10	5	10	Co_Occurence	BCORL1, ZMYM3	10/15
FLNA	ZMYM3	9.14925E-05	11.48136225	60	10	5	10	Co_Occurence	FLNA, ZMYM3	10/15
TSC2	CREBBP	9.25921E-05	9.251424428	56	12	10	7	Co_Occurence	CREBBP, TSC2	12/17
TSC2	POLE	9.25921E-05	9.251424428	56	12	10	7	Co_Occurence	POLE, TSC2	12/17
DNMT1	DCC	9.60631E-05	13.28883341	62	9	4	10	Co_Occurence	DCC, DNMT1	9/14
MTOR	BCORL1	0.000106099	9.733448195	58	11	9	7	Co_Occurence	BCORL1, MTOR	11/16
MTOR	FLNA	0.000106099	9.733448195	58	11	9	7	Co_Occurence	FLNA, MTOR	11/16
FANCA	NCOR2	0.000106099	9.733448195	58	11	9	7	Co_Occurence	FANCA, NCOR2	11/16
NSD1	POLE	0.00010909	11.78126481	59	10	12	4	Co_Occurence	NSD1, POLE	10/16
TPR	DCC	0.000111857	14.05904627	65	8	5	7	Co_Occurence	DCC, TPR	8/12
DNMT1	AXIN1	0.000111867	9.582079494	58	11	8	8	Co_Occurence	AXIN1, DNMT1	11/16
AXIN1	TERT	0.000115737	10.64611137	60	10	6	9	Co_Occurence	AXIN1, TERT	10/15
NF1	SETD1B	0.000121354	10.05590879	57	11	5	12	Co_Occurence	NF1, SETD1B	11/17
NF1	TERT	0.000121354	10.05590879	57	11	5	12	Co_Occurence	NF1, TERT	11/17
FANCA	MYH9	0.000129685	10.27412886	60	10	7	8	Co_Occurence	FANCA, MYH9	10/15
FANCA	RNF213	0.00014403	8.724060532	52	13	15	5	Co_Occurence	FANCA, RNF213	13/20
MTOR	RNF213	0.00014403	8.724060532	52	13	15	5	Co_Occurence	MTOR, RNF213	13/20
NCOR1	MYH9	0.000159801	11.10788772	62	9	8	6	Co_Occurence	MYH9, NCOR1	9/14
STAG1	SPEN	0.000159801	11.10788772	62	9	8	6	Co_Occurence	SPEN, STAG1	9/14
TPR	SPEN	0.000159801	11.10788772	62	9	8	6	Co_Occurence	SPEN, TPR	9/14
NCOR1	TNC	0.000159801	11.10788772	62	9	8	6	Co_Occurence	NCOR1, TNC	9/14

TNC	CREBBP	0.000165287	9.15377904	57	11	11	6	Co_Occurence	CREBBP, TNC	11/17
DCC	FLNA	0.000165327	11.93318566	61	9	11	4	Co_Occurence	DCC, FLNA	9/15
TERT	RNF213	0.000167492	9.609333365	53	12	16	4	Co_Occurence	RNF213, TERT	12/20
TERT	KAT6A	0.000169711	10.8851157	62	9	7	7	Co_Occurence	KAT6A, TERT	9/14
TERT	NIN	0.000169711	10.8851157	62	9	7	7	Co_Occurence	NIN, TERT	9/14
TRRAP	NIN	0.000169711	10.8851157	62	9	7	7	Co_Occurence	NIN, TRRAP	9/14
RNF213	NSD1	0.000178722	11.24053595	54	11	3	17	Co_Occurence	NSD1, RNF213	11/20
BRCA2	NF1	0.000182121	10.72112637	58	10	13	4	Co_Occurence	BRCA2, NF1	10/17
FLNA	POLE	0.000199622	7.977317992	55	12	10	8	Co_Occurence	FLNA, POLE	12/18
UBR5	POLE	0.000199622	7.977317992	55	12	10	8	Co_Occurence	POLE, UBR5	12/18
RNF213	BCORL1	0.000201966	8.232339903	51	14	6	14	Co_Occurence	BCORL1, RNF213	14/20
RNF213	NCOR2	0.000201966	8.232339903	51	14	6	14	Co_Occurence	NCOR2, RNF213	14/20
CREBBP	SMARCA4	0.000207484	7.417330224	53	13	10	9	Co_Occurence	CREBBP, SMARCA4	13/19
POLE	SMARCA4	0.000207484	7.417330224	53	13	10	9	Co_Occurence	POLE, SMARCA4	13/19
DCC	NIN	0.000209745	12.18136185	64	8	8	5	Co_Occurence	DCC, NIN	8/13
DCC	TERT	0.000209745	12.18136185	64	8	8	5	Co_Occurence	DCC, TERT	8/13
COL3A1	TRRAP	0.000209745	12.18136185	64	8	8	5	Co_Occurence	COL3A1, TRRAP	8/13
FLNA	SETD1B	0.000212713	9.459132383	59	10	6	10	Co_Occurence	FLNA, SETD1B	10/16
NCOR2	SETD1B	0.000212713	9.459132383	59	10	6	10	Co_Occurence	NCOR2, SETD1B	10/16
ARID1B	TERT	0.000212713	9.459132383	59	10	6	10	Co_Occurence	ARID1B, TERT	10/16
FLNA	TERT	0.000212713	9.459132383	59	10	6	10	Co_Occurence	FLNA, TERT	10/16
NCOR2	TERT	0.000212713	9.459132383	59	10	6	10	Co_Occurence	NCOR2, TERT	10/16
UBR5	TRRAP	0.000212713	9.459132383	59	10	6	10	Co_Occurence	TRRAP, UBR5	10/16
TSC2	FLNA	0.000221465	8.402572927	57	11	9	8	Co_Occurence	FLNA, TSC2	11/17
AXIN1	NCOR2	0.000221465	8.402572927	57	11	9	8	Co_Occurence	AXIN1, NCOR2	11/17
DNMT1	NCOR2	0.000221465	8.402572927	57	11	9	8	Co_Occurence	DNMT1, NCOR2	11/17
DNMT1	NSD1	0.000235745	10.52024293	61	9	5	10	Co_Occurence	DNMT1, NSD1	9/15
TSC2	NSD1	0.000235745	10.52024293	61	9	5	10	Co_Occurence	NSD1, TSC2	9/15
KAT6B	AXIN1	0.000248251	9.012980062	59	10	9	7	Co_Occurence	AXIN1, KAT6B	10/16
MYH9	BIRC6	0.000248251	9.012980062	59	10	9	7	Co_Occurence	BIRC6, MYH9	10/16
MYH9	DNMT1	0.000248251	9.012980062	59	10	9	7	Co_Occurence	DNMT1, MYH9	10/16
POLD1	DNMT1	0.000248251	9.012980062	59	10	9	7	Co_Occurence	DNMT1, POLD1	10/16
KAT6B	TSC2	0.000248251	9.012980062	59	10	9	7	Co_Occurence	KAT6B, TSC2	10/16
NCOR1	CREBBP	0.000277575	9.312845443	58	10	12	5	Co_Occurence	CREBBP, NCOR1	10/17
STAG1	POLE	0.000277575	9.312845443	58	10	12	5	Co_Occurence	POLE, STAG1	10/17
FAT1	TNC	0.000278101	8.214253008	53	12	5	15	Co_Occurence	FAT1, TNC	12/20
DROSHA	MYH9	0.000289763	8.267862188	56	11	6	12	Co_Occurence	DROSHA, MYH9	11/18
NF1	TNC	0.000289763	8.267862188	56	11	6	12	Co_Occurence	NF1, TNC	11/18
FANCA	NCOR1	0.000294696	9.754872385	61	9	6	9	Co_Occurence	FANCA, NCOR1	9/15

FANCA	RANBP2	0.000294696	9.754872385	61	9	6	9	Co_Occurence	FANCA, RANBP2	9/15
KAT6B	KAT6A	0.000328283	9.416101913	61	9	7	8	Co_Occurence	KAT6A, KAT6B	9/15
SPEN	NIN	0.000328283	9.416101913	61	9	7	8	Co_Occurence	NIN, SPEN	9/15
KAT6B	SETD1B	0.000328283	9.416101913	61	9	7	8	Co_Occurence	KAT6B, SETD1B	9/15
KAT6B	TERT	0.000328283	9.416101913	61	9	7	8	Co_Occurence	KAT6B, TERT	9/15
POLD1	TERT	0.000328283	9.416101913	61	9	7	8	Co_Occurence	POLD1, TERT	9/15
NCOR1	FAT1	0.00033931	8.981960404	54	11	16	4	Co_Occurence	FAT1, NCOR1	11/20
STAG1	FAT1	0.00033931	8.981960404	54	11	16	4	Co_Occurence	FAT1, STAG1	11/20
BCORL1	DROSHA	0.000369511	7.138925776	54	12	11	8	Co_Occurence	BCORL1, DROSHA	12/19
FLNA	DROSHA	0.000369511	7.138925776	54	12	11	8	Co_Occurence	DROSHA, FLNA	12/19
KAT6B	DCC	0.000371158	10.70931515	63	8	5	9	Co_Occurence	DCC, KAT6B	8/14
SPEN	DCC	0.000371158	10.70931515	63	8	5	9	Co_Occurence	DCC, SPEN	8/14
SPEN	RET	0.000371158	10.70931515	63	8	5	9	Co_Occurence	RET, SPEN	8/14
BRCA2	BCORL1	0.000400054	9.441990575	60	9	11	5	Co_Occurence	BCORL1, BRCA2	9/16
BRCA2	FLNA	0.000400054	9.441990575	60	9	11	5	Co_Occurence	BRCA2, FLNA	9/16
COL1A1	NIN	0.000441776	10.05329539	63	8	8	6	Co_Occurence	COL1A1, NIN	8/14
NSD1	SETD1B	0.000441776	10.05329539	63	8	8	6	Co_Occurence	NSD1, SETD1B	8/14
BRCA2	TRRAP	0.000441776	10.05329539	63	8	8	6	Co_Occurence	BRCA2, TRRAP	8/14
NSD1	TRRAP	0.000441776	10.05329539	63	8	8	6	Co_Occurence	NSD1, TRRAP	8/14
EGFR	BCORL1	0.000449633	8.001574042	58	10	10	7	Co_Occurence	BCORL1, EGFR	10/17
TPR	DROSHA	0.000456552	8.471687242	57	10	13	5	Co_Occurence	DROSHA, TPR	10/18
NCOR1	NF1	0.000456552	8.471687242	57	10	13	5	Co_Occurence	NCOR1, NF1	10/18
AXIN1	MTOR	0.000492575	7.782571738	58	10	8	9	Co_Occurence	AXIN1, MTOR	10/17
RNF213	BIRC6	0.00051227	7.155489209	51	13	6	15	Co_Occurence	BIRC6, RNF213	13/21
AXIN1	NCOR1	0.000515765	8.667948309	60	9	6	10	Co_Occurence	AXIN1, NCOR1	9/16
DNMT1	NCOR1	0.000515765	8.667948309	60	9	6	10	Co_Occurence	DNMT1, NCOR1	9/16
AXIN1	ZMYM3	0.000515765	8.667948309	60	9	6	10	Co_Occurence	AXIN1, ZMYM3	9/16
DNMT1	ZMYM3	0.000515765	8.667948309	60	9	6	10	Co_Occurence	DNMT1, ZMYM3	9/16
TSC2	ZMYM3	0.000515765	8.667948309	60	9	6	10	Co_Occurence	TSC2, ZMYM3	9/16
RANBP2	RNF213	0.000524604	8.312296105	53	11	17	4	Co_Occurence	RANBP2, RNF213	11/21
COL3A1	RNF213	0.000568526	9.678756288	54	10	18	3	Co_Occurence	COL3A1, RNF213	10/21
DCC	RNF213	0.000568526	9.678756288	54	10	18	3	Co_Occurence	DCC, RNF213	10/21
MTOR	KAT6A	0.000596973	8.261736046	60	9	7	9	Co_Occurence	KAT6A, MTOR	9/16
FANCA	TERT	0.000596973	8.261736046	60	9	7	9	Co_Occurence	FANCA, TERT	9/16
MTOR	TERT	0.000596973	8.261736046	60	9	7	9	Co_Occurence	MTOR, TERT	9/16
ARID1B	FAT1	0.000625069	6.578711811	51	13	14	7	Co_Occurence	ARID1B, FAT1	13/21
TNC	EGFR	0.000626179	8.134263258	60	9	8	8	Co_Occurence	EGFR, TNC	9/16
MYH9	KAT6B	0.000626179	8.134263258	60	9	8	8	Co_Occurence	KAT6B, MYH9	9/16
TRRAP	CREBBP	0.00062658	7.658466857	57	10	12	6	Co_Occurence	CREBBP, TRRAP	10/18

SETD1B	POLE	0.00062658	7.658466857	57	10	12	6	Co_Occurence	POLE, SETD1B	10/18
TRRAP	POLE	0.00062658	7.658466857	57	10	12	6	Co_Occurence	POLE, TRRAP	10/18
DROSHA	FANCA	0.00062658	6.985426174	55	11	7	12	Co_Occurence	DROSHA, FANCA	11/19
NF1	FANCA	0.00062658	6.985426174	55	11	7	12	Co_Occurence	FANCA, NF1	11/19
COL3A1	NF1	0.000679177	8.997196166	58	9	14	4	Co_Occurence	COL3A1, NF1	9/18
RET	BRCA2	0.000720939	10.34331037	65	7	7	6	Co_Occurence	BRCA2, RET	7/13
COL3A1	N4BP2	0.000720939	10.34331037	65	7	7	6	Co_Occurence	COL3A1, N4BP2	7/13
DCC	NSD1	0.000720939	10.34331037	65	7	7	6	Co_Occurence	DCC, NSD1	7/13
NSD1	EGFR	0.000771328	8.829463622	62	8	9	6	Co_Occurence	EGFR, NSD1	8/15
BRCA2	KAT6B	0.000771328	8.829463622	62	8	9	6	Co_Occurence	BRCA2, KAT6B	8/15
COL1A1	KAT6B	0.000771328	8.829463622	62	8	9	6	Co_Occurence	COL1A1, KAT6B	8/15
NSD1	MYH9	0.000771328	8.829463622	62	8	9	6	Co_Occurence	MYH9, NSD1	8/15
BRCA2	SPEN	0.000771328	8.829463622	62	8	9	6	Co_Occurence	BRCA2, SPEN	8/15
ZMYM3	SETD1B	0.000853551	8.518799385	62	8	8	7	Co_Occurence	SETD1B, ZMYM3	8/15
NCOR1	TERT	0.000853551	8.518799385	62	8	8	7	Co_Occurence	NCOR1, TERT	8/15
NCOR1	TRRAP	0.000853551	8.518799385	62	8	8	7	Co_Occurence	NCOR1, TRRAP	8/15
RANBP2	TRRAP	0.000853551	8.518799385	62	8	8	7	Co_Occurence	RANBP2, TRRAP	8/15
FAT1	NIN	0.000861309	7.08057467	53	11	5	16	Co_Occurence	FAT1, NIN	11/21
FAT1	SETD1B	0.000861309	7.08057467	53	11	5	16	Co_Occurence	FAT1, SETD1B	11/21
FAT1	TRRAP	0.000861309	7.08057467	53	11	5	16	Co_Occurence	FAT1, TRRAP	11/21
NCOR2	NCOR1	0.000862896	7.773088101	59	9	6	11	Co_Occurence	NCOR1, NCOR2	9/17
ARID1B	RANBP2	0.000862896	7.773088101	59	9	6	11	Co_Occurence	ARID1B, RANBP2	9/17
FLNA	STAG1	0.000862896	7.773088101	59	9	6	11	Co_Occurence	FLNA, STAG1	9/17
BCORL1	TPR	0.000862896	7.773088101	59	9	6	11	Co_Occurence	BCORL1, TPR	9/17
FANCA	ARID1B	0.00087905	6.904752815	57	10	10	8	Co_Occurence	ARID1B, FANCA	10/18
FANCA	FLNA	0.00087905	6.904752815	57	10	10	8	Co_Occurence	FANCA, FLNA	10/18
BIRC6	AXIN1	0.000915582	6.819816132	57	10	9	9	Co_Occurence	AXIN1, BIRC6	10/18
TSC2	AXIN1	0.000915582	6.819816132	57	10	9	9	Co_Occurence	AXIN1, TSC2	10/18
FAT1	RNF213	0.000979451	5.443220712	46	16	12	11	Co_Occurence	FAT1, RNF213	16/23
BIRC6	DCC	0.001010345	8.54686191	61	8	5	11	Co_Occurence	BIRC6, DCC	8/16
TSC2	DCC	0.001010345	8.54686191	61	8	5	11	Co_Occurence	DCC, TSC2	8/16
BIRC6	RET	0.001010345	8.54686191	61	8	5	11	Co_Occurence	BIRC6, RET	8/16
NF1	NIN	0.001015215	6.963645767	56	10	6	13	Co_Occurence	NF1, NIN	10/19
COL1A1	CREBBP	0.001029277	7.768405594	58	9	13	5	Co_Occurence	COL1A1, CREBBP	9/18
NSD1	CREBBP	0.001029277	7.768405594	58	9	13	5	Co_Occurence	CREBBP, NSD1	9/18
BRCA2	POLE	0.001029277	7.768405594	58	9	13	5	Co_Occurence	BRCA2, POLE	9/18
SETD1B	AXIN1	0.001030086	7.335343046	59	9	10	7	Co_Occurence	AXIN1, SETD1B	9/17
TERT	DNMT1	0.001030086	7.335343046	59	9	10	7	Co_Occurence	DNMT1, TERT	9/17
NIN	TSC2	0.001030086	7.335343046	59	9	10	7	Co_Occurence	NIN, TSC2	9/17

TERT	TSC2	0.001030086	7.335343046	59	9	10	7	Co_Occurence	TERT, TSC2	9/17
MTOR	FAT1	0.001039875	6.739937886	52	12	15	6	Co_Occurence	FAT1, MTOR	12/21
FAT1	COL1A1	0.00105578	7.706466835	54	10	4	17	Co_Occurence	COL1A1, FAT1	10/21
SPEN	FANCA	0.001122672	7.135715775	59	9	9	8	Co_Occurence	FANCA, SPEN	9/17
TNC	FANCA	0.001122672	7.135715775	59	9	9	8	Co_Occurence	FANCA, TNC	9/17
MYH9	MTOR	0.001122672	7.135715775	59	9	9	8	Co_Occurence	MTOR, MYH9	9/17
NF1	FAT1	0.001189223	5.716574532	49	14	13	9	Co_Occurence	FAT1, NF1	14/22
DCC	NCOR1	0.001253887	8.952637268	64	7	8	6	Co_Occurence	DCC, NCOR1	7/14
DCC	RANBP2	0.001253887	8.952637268	64	7	8	6	Co_Occurence	DCC, RANBP2	7/14
RET	TPR	0.001253887	8.952637268	64	7	8	6	Co_Occurence	RET, TPR	7/14
BRCA2	FANCA	0.001282134	7.847655356	61	8	10	6	Co_Occurence	BRCA2, FANCA	8/16
N4BP2	FANCA	0.001282134	7.847655356	61	8	10	6	Co_Occurence	FANCA, N4BP2	8/16
CREBBP	KAT6B	0.00128557	6.473361949	56	10	7	12	Co_Occurence	CREBBP, KAT6B	10/19
POLE	KAT6B	0.00128557	6.473361949	56	10	7	12	Co_Occurence	KAT6B, POLE	10/19
RANBP2	EGFR	0.001470217	7.479465473	61	8	9	7	Co_Occurence	EGFR, RANBP2	8/16
ZMYM3	KAT6B	0.001470217	7.479465473	61	8	9	7	Co_Occurence	KAT6B, ZMYM3	8/16
NCOR1	POLD1	0.001470217	7.479465473	61	8	9	7	Co_Occurence	NCOR1, POLD1	8/16
STAG1	POLD1	0.001470217	7.479465473	61	8	9	7	Co_Occurence	POLD1, STAG1	8/16
NCOR1	SPEN	0.001470217	7.479465473	61	8	9	7	Co_Occurence	NCOR1, SPEN	8/16
NIN	KAT6A	0.00153739	7.364429079	61	8	8	8	Co_Occurence	KAT6A, NIN	8/16
SETD1B	KAT6A	0.00153739	7.364429079	61	8	8	8	Co_Occurence	KAT6A, SETD1B	8/16
SETD1B	NIN	0.00153739	7.364429079	61	8	8	8	Co_Occurence	NIN, SETD1B	8/16
TERT	SETD1B	0.00153739	7.364429079	61	8	8	8	Co_Occurence	SETD1B, TERT	8/16
TRRAP	SETD1B	0.00153739	7.364429079	61	8	8	8	Co_Occurence	SETD1B, TRRAP	8/16
RNF213	DROSHA	0.001544091	5.209623086	48	14	9	14	Co_Occurence	DROSHA, RNF213	14/23
COL3A1	BCORL1	0.00157369	7.729269372	60	8	12	5	Co_Occurence	BCORL1, COL3A1	8/17
DCC	BCORL1	0.00157369	7.729269372	60	8	12	5	Co_Occurence	BCORL1, DCC	8/17
RET	BCORL1	0.00157369	7.729269372	60	8	12	5	Co_Occurence	BCORL1, RET	8/17
COL3A1	FLNA	0.00157369	7.729269372	60	8	12	5	Co_Occurence	COL3A1, FLNA	8/17
DCC	NCOR2	0.00157369	7.729269372	60	8	12	5	Co_Occurence	DCC, NCOR2	8/17
NSD1	DROSHA	0.001573878	7.10565818	57	9	14	5	Co_Occurence	DROSHA, NSD1	9/19
COL1A1	SMARCA4	0.001573878	7.10565818	57	9	14	5	Co_Occurence	COL1A1, SMARCA4	9/19
BCORL1	AXIN1	0.0016097	6.048152657	56	10	9	10	Co_Occurence	AXIN1, BCORL1	10/19
FLNA	BIRC6	0.0016097	6.048152657	56	10	9	10	Co_Occurence	BIRC6, FLNA	10/19
NCOR2	BIRC6	0.0016097	6.048152657	56	10	9	10	Co_Occurence	BIRC6, NCOR2	10/19
BCORL1	DNMT1	0.0016097	6.048152657	56	10	9	10	Co_Occurence	BCORL1, DNMT1	10/19
UBR5	TSC2	0.0016097	6.048152657	56	10	9	10	Co_Occurence	TSC2, UBR5	10/19
CREBBP	DROSHA	0.001628903	5.529729445	52	12	11	10	Co_Occurence	CREBBP, DROSHA	12/21
POLE	NF1	0.001628903	5.529729445	52	12	11	10	Co_Occurence	NF1, POLE	12/21

TRRAP	ARID1B	0.001698846	6.574934412	58	9	11	7	Co_Occurence	ARID1B, TRRAP	9/18
NIN	BCORL1	0.001698846	6.574934412	58	9	11	7	Co_Occurence	BCORL1, NIN	9/18
SETD1B	BCORL1	0.001698846	6.574934412	58	9	11	7	Co_Occurence	BCORL1, SETD1B	9/18
TRRAP	FLNA	0.001698846	6.574934412	58	9	11	7	Co_Occurence	FLNA, TRRAP	9/18
SETD1B	UBR5	0.001698846	6.574934412	58	9	11	7	Co_Occurence	SETD1B, UBR5	9/18
POLD1	BIRC6	0.001909651	6.332874487	58	9	10	8	Co_Occurence	BIRC6, POLD1	9/18
SPEN	BIRC6	0.001909651	6.332874487	58	9	10	8	Co_Occurence	BIRC6, SPEN	9/18
EGFR	DNMT1	0.001909651	6.332874487	58	9	10	8	Co_Occurence	DNMT1, EGFR	9/18
MYH9	TSC2	0.001909651	6.332874487	58	9	10	8	Co_Occurence	MYH9, TSC2	9/18
NSD1	BIRC6	0.002043144	7.03738011	60	8	11	6	Co_Occurence	BIRC6, NSD1	8/17
COL1A1	DNMT1	0.002043144	7.03738011	60	8	11	6	Co_Occurence	COL1A1, DNMT1	8/17
COL1A1	TSC2	0.002043144	7.03738011	60	8	11	6	Co_Occurence	COL1A1, TSC2	8/17
RNF213	SETD1B	0.00204615	6.549455358	52	11	5	17	Co_Occurence	RNF213, SETD1B	11/22
RNF213	TRRAP	0.00204615	6.549455358	52	11	5	17	Co_Occurence	RNF213, TRRAP	11/22
KAT6B	DROSHA	0.002051575	5.882126452	55	10	13	7	Co_Occurence	DROSHA, KAT6B	10/20
POLD1	DROSHA	0.002051575	5.882126452	55	10	13	7	Co_Occurence	DROSHA, POLD1	10/20
KAT6B	NF1	0.002051575	5.882126452	55	10	13	7	Co_Occurence	KAT6B, NF1	10/20
MYH9	NF1	0.002051575	5.882126452	55	10	13	7	Co_Occurence	MYH9, NF1	10/20
MYH9	SMARCA4	0.002051575	5.882126452	55	10	13	7	Co_Occurence	MYH9, SMARCA4	10/20
DCC	KAT6A	0.002065997	7.867657737	63	7	9	6	Co_Occurence	DCC, KAT6A	7/15
DCC	TRRAP	0.002065997	7.867657737	63	7	9	6	Co_Occurence	DCC, TRRAP	7/15
RET	TRRAP	0.002065997	7.867657737	63	7	9	6	Co_Occurence	RET, TRRAP	7/15
POLE	TPR	0.002156831	6.387619516	57	9	6	13	Co_Occurence	POLE, TPR	9/19
CREBBP	ZMYM3	0.002156831	6.387619516	57	9	6	13	Co_Occurence	CREBBP, ZMYM3	9/19
NF1	DNMT1	0.002255203	6.019682935	54	11	8	12	Co_Occurence	DNMT1, NF1	11/20
DROSHA	TSC2	0.002255203	6.019682935	54	11	8	12	Co_Occurence	DROSHA, TSC2	11/20
NF1	TSC2	0.002255203	6.019682935	54	11	8	12	Co_Occurence	NF1, TSC2	11/20
BRCA2	NCOR1	0.002269024	7.591493023	63	7	8	7	Co_Occurence	BRCA2, NCOR1	7/15
NSD1	NCOR1	0.002269024	7.591493023	63	7	8	7	Co_Occurence	NCOR1, NSD1	7/15
NSD1	RANBP2	0.002269024	7.591493023	63	7	8	7	Co_Occurence	NSD1, RANBP2	7/15
COL1A1	STAG1	0.002269024	7.591493023	63	7	8	7	Co_Occurence	COL1A1, STAG1	7/15
N4BP2	STAG1	0.002269024	7.591493023	63	7	8	7	Co_Occurence	N4BP2, STAG1	7/15
NSD1	STAG1	0.002269024	7.591493023	63	7	8	7	Co_Occurence	NSD1, STAG1	7/15
BRCA2	TPR	0.002269024	7.591493023	63	7	8	7	Co_Occurence	BRCA2, TPR	7/15
BRCA2	ZMYM3	0.002269024	7.591493023	63	7	8	7	Co_Occurence	BRCA2, ZMYM3	7/15
COL1A1	ZMYM3	0.002269024	7.591493023	63	7	8	7	Co_Occurence	COL1A1, ZMYM3	7/15
BCORL1	CREBBP	0.00234961	5.839898441	54	11	11	9	Co_Occurence	BCORL1, CREBBP	11/20
UBR5	CREBBP	0.00234961	5.839898441	54	11	11	9	Co_Occurence	CREBBP, UBR5	11/20
RNF213	TSC2	0.002402821	5.232625286	50	12	7	16	Co_Occurence	RNF213, TSC2	12/23

STAG1	FANCA	0.002410634	6.642364919	60	8	10	7	Co_Occurence	FANCA, STAG1	8/17
TPR	FANCA	0.002410634	6.642364919	60	8	10	7	Co_Occurence	FANCA, TPR	8/17
FANCA	CREBBP	0.002438802	5.580187026	55	10	12	8	Co_Occurence	CREBBP, FANCA	10/20
KAT6B	FAT1	0.002549108	5.808139687	52	11	16	6	Co_Occurence	FAT1, KAT6B	11/22
POLD1	FAT1	0.002549108	5.808139687	52	11	16	6	Co_Occurence	FAT1, POLD1	11/22
SPEN	FAT1	0.002549108	5.808139687	52	11	16	6	Co_Occurence	FAT1, SPEN	11/22
NF1	DROSHA	0.002565276	4.941399027	51	12	11	11	Co_Occurence	DROSHA, NF1	12/22
NIN	KAT6B	0.002612152	6.461217338	60	8	9	8	Co_Occurence	KAT6B, NIN	8/17
TRRAP	KAT6B	0.002612152	6.461217338	60	8	9	8	Co_Occurence	KAT6B, TRRAP	8/17
KAT6A	MYH9	0.002612152	6.461217338	60	8	9	8	Co_Occurence	KAT6A, MYH9	8/17
TRRAP	MYH9	0.002612152	6.461217338	60	8	9	8	Co_Occurence	MYH9, TRRAP	8/17
NIN	POLD1	0.002612152	6.461217338	60	8	9	8	Co_Occurence	NIN, POLD1	8/17
SETD1B	POLD1	0.002612152	6.461217338	60	8	9	8	Co_Occurence	POLD1, SETD1B	8/17
KAT6A	SPEN	0.002612152	6.461217338	60	8	9	8	Co_Occurence	KAT6A, SPEN	8/17
TERT	SPEN	0.002612152	6.461217338	60	8	9	8	Co_Occurence	SPEN, TERT	8/17
SETD1B	TNC	0.002612152	6.461217338	60	8	9	8	Co_Occurence	SETD1B, TNC	8/17
TRRAP	TNC	0.002612152	6.461217338	60	8	9	8	Co_Occurence	TNC, TRRAP	8/17
ARID1B	DROSHA	0.002978994	5.265879113	53	11	12	9	Co_Occurence	ARID1B, DROSHA	11/21
FLNA	NF1	0.002978994	5.265879113	53	11	12	9	Co_Occurence	FLNA, NF1	11/21
NCOR2	NF1	0.002978994	5.265879113	53	11	12	9	Co_Occurence	NCOR2, NF1	11/21
KAT6B	ARID1B	0.003104211	5.672289247	57	9	11	8	Co_Occurence	ARID1B, KAT6B	9/19
MYH9	ARID1B	0.003104211	5.672289247	57	9	11	8	Co_Occurence	ARID1B, MYH9	9/19
TNC	ARID1B	0.003104211	5.672289247	57	9	11	8	Co_Occurence	ARID1B, TNC	9/19
MYH9	BCORL1	0.003104211	5.672289247	57	9	11	8	Co_Occurence	BCORL1, MYH9	9/19
KAT6B	FLNA	0.003104211	5.672289247	57	9	11	8	Co_Occurence	FLNA, KAT6B	9/19
POLD1	FLNA	0.003104211	5.672289247	57	9	11	8	Co_Occurence	FLNA, POLD1	9/19
KAT6B	NCOR2	0.003104211	5.672289247	57	9	11	8	Co_Occurence	KAT6B, NCOR2	9/19
POLD1	NCOR2	0.003104211	5.672289247	57	9	11	8	Co_Occurence	NCOR2, POLD1	9/19
NSD1	ARID1B	0.003138537	6.361528828	59	8	12	6	Co_Occurence	ARID1B, NSD1	8/18
COL1A1	BCORL1	0.003138537	6.361528828	59	8	12	6	Co_Occurence	BCORL1, COL1A1	8/18
N4BP2	FLNA	0.003138537	6.361528828	59	8	12	6	Co_Occurence	FLNA, N4BP2	8/18
NSD1	UBR5	0.003138537	6.361528828	59	8	12	6	Co_Occurence	NSD1, UBR5	8/18
DROSHA	NCOR1	0.003249867	5.840044527	56	9	6	14	Co_Occurence	DROSHA, NCOR1	9/20
NF1	TPR	0.003249867	5.840044527	56	9	6	14	Co_Occurence	NF1, TPR	9/20
SMARCA4	TPR	0.003249867	5.840044527	56	9	6	14	Co_Occurence	SMARCA4, TPR	9/20
DROSHA	ZMYM3	0.003249867	5.840044527	56	9	6	14	Co_Occurence	DROSHA, ZMYM3	9/20
SMARCA4	ZMYM3	0.003249867	5.840044527	56	9	6	14	Co_Occurence	SMARCA4, ZMYM3	9/20
DCC	EGFR	0.003252384	6.993011754	62	7	10	6	Co_Occurence	DCC, EGFR	7/16
COL3A1	KAT6B	0.003252384	6.993011754	62	7	10	6	Co_Occurence	COL3A1, KAT6B	7/16

COL3A1	TNC	0.003252384	6.993011754	62	7	10	6	Co_Occurence	COL3A1, TNC	7/16
EGFR	RNF213	0.003326407	5.37034673	51	11	17	6	Co_Occurence	EGFR, RNF213	11/23
POLD1	RNF213	0.003326407	5.37034673	51	11	17	6	Co_Occurence	POLD1, RNF213	11/23
BIRC6	MTOR	0.003326727	5.548196764	57	9	9	10	Co_Occurence	BIRC6, MTOR	9/19
COL1A1	KAT6A	0.003690217	6.665363765	62	7	9	7	Co_Occurence	COL1A1, KAT6A	7/16
N4BP2	KAT6A	0.003690217	6.665363765	62	7	9	7	Co_Occurence	KAT6A, N4BP2	7/16
BRCA2	NIN	0.003690217	6.665363765	62	7	9	7	Co_Occurence	BRCA2, NIN	7/16
N4BP2	NIN	0.003690217	6.665363765	62	7	9	7	Co_Occurence	N4BP2, NIN	7/16
NSD1	NIN	0.003690217	6.665363765	62	7	9	7	Co_Occurence	NIN, NSD1	7/16
AXIN1	TPR	0.003788721	5.954926393	59	8	7	11	Co_Occurence	AXIN1, TPR	8/18
BIRC6	TPR	0.003788721	5.954926393	59	8	7	11	Co_Occurence	BIRC6, TPR	8/18
DCC	COL3A1	0.003809858	7.662271984	65	6	7	7	Co_Occurence	COL3A1, DCC	6/14
RET	DCC	0.003809858	7.662271984	65	6	7	7	Co_Occurence	DCC, RET	6/14
RANBP2	NCOR1	0.003845347	6.563553731	62	7	8	8	Co_Occurence	NCOR1, RANBP2	7/16
ZMYM3	NCOR1	0.003845347	6.563553731	62	7	8	8	Co_Occurence	NCOR1, ZMYM3	7/16
ZMYM3	STAG1	0.003845347	6.563553731	62	7	8	8	Co_Occurence	STAG1, ZMYM3	7/16
CREBBP	TERT	0.004124296	5.397258832	56	9	7	13	Co_Occurence	CREBBP, TERT	9/20
POLE	TERT	0.004124296	5.397258832	56	9	7	13	Co_Occurence	POLE, TERT	9/20
DROSHA	FAT1	0.004211831	4.366313224	48	13	14	10	Co_Occurence	DROSHA, FAT1	13/24
MTOR	SETD1B	0.004224295	5.735223587	59	8	8	10	Co_Occurence	MTOR, SETD1B	8/18
MTOR	TRRAP	0.004224295	5.735223587	59	8	8	10	Co_Occurence	MTOR, TRRAP	8/18
TNC	KAT6B	0.004377461	5.665582261	59	8	9	9	Co_Occurence	KAT6B, TNC	8/18
SPEN	MYH9	0.004377461	5.665582261	59	8	9	9	Co_Occurence	MYH9, SPEN	8/18
TNC	SPEN	0.004377461	5.665582261	59	8	9	9	Co_Occurence	SPEN, TNC	8/18
POLE	CREBBP	0.004610852	4.622907978	52	11	11	11	Co_Occurence	CREBBP, POLE	11/22
UBR5	ARID1B	0.004806175	5.36025666	55	10	10	10	Co_Occurence	ARID1B, UBR5	10/20
UBR5	BCORL1	0.004806175	5.36025666	55	10	10	10	Co_Occurence	BCORL1, UBR5	10/20
UBR5	FLNA	0.004806175	5.36025666	55	10	10	10	Co_Occurence	FLNA, UBR5	10/20
RNF213	STAG1	0.004806394	5.63803959	52	10	5	18	Co_Occurence	RNF213, STAG1	10/23
FAT1	FLNA	0.004820317	4.888263464	50	12	8	15	Co_Occurence	FAT1, FLNA	12/23
FAT1	NCOR2	0.004820317	4.888263464	50	12	8	15	Co_Occurence	FAT1, NCOR2	12/23
FAT1	UBR5	0.004820317	4.888263464	50	12	8	15	Co_Occurence	FAT1, UBR5	12/23
MTOR	COL3A1	0.004924111	6.273714793	61	7	6	11	Co_Occurence	COL3A1, MTOR	7/17
FANCA	DCC	0.004924111	6.273714793	61	7	6	11	Co_Occurence	DCC, FANCA	7/17
MTOR	DCC	0.004924111	6.273714793	61	7	6	11	Co_Occurence	DCC, MTOR	7/17
MTOR	RET	0.004924111	6.273714793	61	7	6	11	Co_Occurence	MTOR, RET	7/17
RET	DROSHA	0.004973741	5.916809327	57	8	15	5	Co_Occurence	DROSHA, RET	8/20
DCC	NF1	0.004973741	5.916809327	57	8	15	5	Co_Occurence	DCC, NF1	8/20
MTOR	NF1	0.00536547	5.06798172	54	10	13	8	Co_Occurence	MTOR, NF1	10/21

POLD1	BRCA2	0.005733439	5.92123383	61	7	7	10	Co_Occurence	BRCA2, POLD1	7/17
SPEN	COL1A1	0.005733439	5.92123383	61	7	7	10	Co_Occurence	COL1A1, SPEN	7/17
TNC	COL1A1	0.005733439	5.92123383	61	7	7	10	Co_Occurence	COL1A1, TNC	7/17
SPEN	N4BP2	0.005733439	5.92123383	61	7	7	10	Co_Occurence	N4BP2, SPEN	7/17
KAT6B	NSD1	0.005733439	5.92123383	61	7	7	10	Co_Occurence	KAT6B, NSD1	7/17
NCOR1	ARID1B	0.00573927	5.37946923	58	8	12	7	Co_Occurence	ARID1B, NCOR1	8/19
ZMYM3	ARID1B	0.00573927	5.37946923	58	8	12	7	Co_Occurence	ARID1B, ZMYM3	8/19
STAG1	BCORL1	0.00573927	5.37946923	58	8	12	7	Co_Occurence	BCORL1, STAG1	8/19
NCOR1	FLNA	0.00573927	5.37946923	58	8	12	7	Co_Occurence	FLNA, NCOR1	8/19
RANBP2	FLNA	0.00573927	5.37946923	58	8	12	7	Co_Occurence	FLNA, RANBP2	8/19
TPR	FLNA	0.00573927	5.37946923	58	8	12	7	Co_Occurence	FLNA, TPR	8/19
RANBP2	UBR5	0.00573927	5.37946923	58	8	12	7	Co_Occurence	RANBP2, UBR5	8/19
STAG1	UBR5	0.00573927	5.37946923	58	8	12	7	Co_Occurence	STAG1, UBR5	8/19
ZMYM3	UBR5	0.00573927	5.37946923	58	8	12	7	Co_Occurence	UBR5, ZMYM3	8/19
AXIN1	CREBBP	0.005773318	4.883010289	54	10	12	9	Co_Occurence	AXIN1, CREBBP	10/21
AXIN1	POLE	0.005773318	4.883010289	54	10	12	9	Co_Occurence	AXIN1, POLE	10/21
COL3A1	BRCA2	0.006126548	6.628193783	64	6	8	7	Co_Occurence	BRCA2, COL3A1	6/15
DCC	BRCA2	0.006126548	6.628193783	64	6	8	7	Co_Occurence	BRCA2, DCC	6/15
COL3A1	NSD1	0.006126548	6.628193783	64	6	8	7	Co_Occurence	COL3A1, NSD1	6/15
STAG1	KAT6A	0.0061723	5.759587046	61	7	9	8	Co_Occurence	KAT6A, STAG1	7/17
ZMYM3	KAT6A	0.0061723	5.759587046	61	7	9	8	Co_Occurence	KAT6A, ZMYM3	7/17
NCOR1	NIN	0.0061723	5.759587046	61	7	9	8	Co_Occurence	NCOR1, NIN	7/17
RANBP2	NIN	0.0061723	5.759587046	61	7	9	8	Co_Occurence	NIN, RANBP2	7/17
TPR	NIN	0.0061723	5.759587046	61	7	9	8	Co_Occurence	NIN, TPR	7/17
RANBP2	SETD1B	0.0061723	5.759587046	61	7	9	8	Co_Occurence	RANBP2, SETD1B	7/17
STAG1	TERT	0.0061723	5.759587046	61	7	9	8	Co_Occurence	STAG1, TERT	7/17
ZMYM3	TERT	0.0061723	5.759587046	61	7	9	8	Co_Occurence	TERT, ZMYM3	7/17
STAG1	TRRAP	0.0061723	5.759587046	61	7	9	8	Co_Occurence	STAG1, TRRAP	7/17
TPR	TRRAP	0.0061723	5.759587046	61	7	9	8	Co_Occurence	TPR, TRRAP	7/17
ZMYM3	TRRAP	0.0061723	5.759587046	61	7	9	8	Co_Occurence	TRRAP, ZMYM3	7/17
FAT1	KAT6A	0.006217311	4.98287035	52	10	6	17	Co_Occurence	FAT1, KAT6A	10/23
NIN	AXIN1	0.006547355	5.13877401	58	8	11	8	Co_Occurence	AXIN1, NIN	8/19
TRRAP	AXIN1	0.006547355	5.13877401	58	8	11	8	Co_Occurence	AXIN1, TRRAP	8/19
KAT6A	BIRC6	0.006547355	5.13877401	58	8	11	8	Co_Occurence	BIRC6, KAT6A	8/19
NIN	BIRC6	0.006547355	5.13877401	58	8	11	8	Co_Occurence	BIRC6, NIN	8/19
SETD1B	BIRC6	0.006547355	5.13877401	58	8	11	8	Co_Occurence	BIRC6, SETD1B	8/19
SETD1B	DNMT1	0.006547355	5.13877401	58	8	11	8	Co_Occurence	DNMT1, SETD1B	8/19
POLE	COL1A1	0.006747583	5.291805939	57	8	6	14	Co_Occurence	COL1A1, POLE	8/20
SPEN	MTOR	0.006981292	5.026394334	58	8	10	9	Co_Occurence	MTOR, SPEN	8/19

AXIN1	COL3A1	0.007207142	5.671592306	60	7	6	12	Co_Occurence	AXIN1, COL3A1	7/18
TSC2	COL3A1	0.007207142	5.671592306	60	7	6	12	Co_Occurence	COL3A1, TSC2	7/18
TSC2	RET	0.007207142	5.671592306	60	7	6	12	Co_Occurence	RET, TSC2	7/18
AXIN1	DROSHA	0.007710596	4.432611604	53	10	13	9	Co_Occurence	AXIN1, DROSHA	10/22
DNMT1	DROSHA	0.007710596	4.432611604	53	10	13	9	Co_Occurence	DNMT1, DROSHA	10/22
AXIN1	SMARCA4	0.007710596	4.432611604	53	10	13	9	Co_Occurence	AXIN1, SMARCA4	10/22
BIRC6	SMARCA4	0.007710596	4.432611604	53	10	13	9	Co_Occurence	BIRC6, SMARCA4	10/22
DNMT1	SMARCA4	0.007710596	4.432611604	53	10	13	9	Co_Occurence	DNMT1, SMARCA4	10/22
NCOR2	CREBBP	0.00823626	4.323390897	53	10	12	10	Co_Occurence	CREBBP, NCOR2	10/22
BCORL1	POLE	0.00823626	4.323390897	53	10	12	10	Co_Occurence	BCORL1, POLE	10/22
RNF213	SMARCA4	0.008541292	3.996685235	47	13	10	15	Co_Occurence	RNF213, SMARCA4	13/25
NSD1	FANCA	0.008566088	5.309269429	60	7	11	7	Co_Occurence	FANCA, NSD1	7/18
NSD1	MTOR	0.008566088	5.309269429	60	7	11	7	Co_Occurence	MTOR, NSD1	7/18
COL3A1	RANBP2	0.009374988	5.820584765	63	6	9	7	Co_Occurence	COL3A1, RANBP2	6/16
DCC	STAG1	0.009374988	5.820584765	63	6	9	7	Co_Occurence	DCC, STAG1	6/16
COL3A1	TPR	0.009374988	5.820584765	63	6	9	7	Co_Occurence	COL3A1, TPR	6/16
COL3A1	ZMYM3	0.009374988	5.820584765	63	6	9	7	Co_Occurence	COL3A1, ZMYM3	6/16
DCC	ZMYM3	0.009374988	5.820584765	63	6	9	7	Co_Occurence	DCC, ZMYM3	6/16
NSD1	FAT1	0.009377447	5.177634287	53	9	18	5	Co_Occurence	FAT1, NSD1	9/23
FANCA	BCORL1	0.009458029	4.967500798	56	9	11	9	Co_Occurence	BCORL1, FANCA	9/20
FANCA	UBR5	0.009458029	4.967500798	56	9	11	9	Co_Occurence	FANCA, UBR5	9/20
NCOR1	KAT6B	0.009463759	5.113474521	60	7	10	8	Co_Occurence	KAT6B, NCOR1	7/18
STAG1	KAT6B	0.009463759	5.113474521	60	7	10	8	Co_Occurence	KAT6B, STAG1	7/18
TPR	KAT6B	0.009463759	5.113474521	60	7	10	8	Co_Occurence	KAT6B, TPR	7/18
RANBP2	MYH9	0.009463759	5.113474521	60	7	10	8	Co_Occurence	MYH9, RANBP2	7/18
STAG1	MYH9	0.009463759	5.113474521	60	7	10	8	Co_Occurence	MYH9, STAG1	7/18
TPR	MYH9	0.009463759	5.113474521	60	7	10	8	Co_Occurence	MYH9, TPR	7/18
ZMYM3	MYH9	0.009463759	5.113474521	60	7	10	8	Co_Occurence	MYH9, ZMYM3	7/18
RANBP2	POLD1	0.009463759	5.113474521	60	7	10	8	Co_Occurence	POLD1, RANBP2	7/18
TPR	POLD1	0.009463759	5.113474521	60	7	10	8	Co_Occurence	POLD1, TPR	7/18
RANBP2	SPEN	0.009463759	5.113474521	60	7	10	8	Co_Occurence	RANBP2, SPEN	7/18
RANBP2	TNC	0.009463759	5.113474521	60	7	10	8	Co_Occurence	RANBP2, TNC	7/18
TPR	TNC	0.009463759	5.113474521	60	7	10	8	Co_Occurence	TNC, TPR	7/18
DNMT1	BIRC6	0.009563793	4.918578136	56	9	10	10	Co_Occurence	BIRC6, DNMT1	9/20
DROSHA	SETD1B	0.009596999	4.932094	55	9	7	14	Co_Occurence	DROSHA, SETD1B	9/21
SMARCA4	SETD1B	0.009596999	4.932094	55	9	7	14	Co_Occurence	SETD1B, SMARCA4	9/21
COL1A1	BRCA2	0.009730287	5.730816429	63	6	8	8	Co_Occurence	BRCA2, COL1A1	6/16
NSD1	BRCA2	0.009730287	5.730816429	63	6	8	8	Co_Occurence	BRCA2, NSD1	6/16
NSD1	COL1A1	0.009730287	5.730816429	63	6	8	8	Co_Occurence	COL1A1, NSD1	6/16

NSD1	N4BP2	0.009730287	5.730816429	63	6	8	8	Co_Occurence	N4BP2, NSD1	6/16
KAT6A	ARID1B	0.009779694	4.639912054	57	8	12	8	Co_Occurence	ARID1B, KAT6A	8/20
SETD1B	ARID1B	0.009779694	4.639912054	57	8	12	8	Co_Occurence	ARID1B, SETD1B	8/20
KAT6A	FLNA	0.009779694	4.639912054	57	8	12	8	Co_Occurence	FLNA, KAT6A	8/20
NIN	FLNA	0.009779694	4.639912054	57	8	12	8	Co_Occurence	FLNA, NIN	8/20
NIN	NCOR2	0.009779694	4.639912054	57	8	12	8	Co_Occurence	NCOR2, NIN	8/20
TRRAP	NCOR2	0.009779694	4.639912054	57	8	12	8	Co_Occurence	NCOR2, TRRAP	8/20
NIN	UBR5	0.009779694	4.639912054	57	8	12	8	Co_Occurence	NIN, UBR5	8/20
TERT	UBR5	0.009779694	4.639912054	57	8	12	8	Co_Occurence	TERT, UBR5	8/20
COL3A1	ARID1B	0.010240539	5.160095319	59	7	13	6	Co_Occurence	ARID1B, COL3A1	7/19
DCC	ARID1B	0.010240539	5.160095319	59	7	13	6	Co_Occurence	ARID1B, DCC	7/19
RET	ARID1B	0.010240539	5.160095319	59	7	13	6	Co_Occurence	ARID1B, RET	7/19
RET	FLNA	0.010240539	5.160095319	59	7	13	6	Co_Occurence	FLNA, RET	7/19
RET	NCOR2	0.010240539	5.160095319	59	7	13	6	Co_Occurence	NCOR2, RET	7/19
TSC2	FAT1	0.010395457	4.21141028	50	11	16	8	Co_Occurence	FAT1, TSC2	11/24
CREBBP	NF1	0.01041519	4.164360659	51	11	12	11	Co_Occurence	CREBBP, NF1	11/23
MYH9	CREBBP	0.010489783	4.651654611	55	9	13	8	Co_Occurence	CREBBP, MYH9	9/21
SPEN	CREBBP	0.010489783	4.651654611	55	9	13	8	Co_Occurence	CREBBP, SPEN	9/21
MYH9	POLE	0.010489783	4.651654611	55	9	13	8	Co_Occurence	MYH9, POLE	9/21
RNF213	BRCA2	0.01117961	4.818971852	52	9	5	19	Co_Occurence	BRCA2, RNF213	9/24
ARID1B	BIRC6	0.011573299	4.401682161	55	9	10	11	Co_Occurence	ARID1B, BIRC6	9/21
BCORL1	BIRC6	0.011573299	4.401682161	55	9	10	11	Co_Occurence	BCORL1, BIRC6	9/21
FLNA	DNMT1	0.011573299	4.401682161	55	9	10	11	Co_Occurence	DNMT1, FLNA	9/21
ARID1B	TSC2	0.011573299	4.401682161	55	9	10	11	Co_Occurence	ARID1B, TSC2	9/21
NCOR2	TSC2	0.011573299	4.401682161	55	9	10	11	Co_Occurence	NCOR2, TSC2	9/21
BRCA2	BIRC6	0.012371139	4.797295075	59	7	12	7	Co_Occurence	BIRC6, BRCA2	7/19
BRCA2	DNMT1	0.012371139	4.797295075	59	7	12	7	Co_Occurence	BRCA2, DNMT1	7/19
BRCA2	TSC2	0.012371139	4.797295075	59	7	12	7	Co_Occurence	BRCA2, TSC2	7/19
N4BP2	TSC2	0.012371139	4.797295075	59	7	12	7	Co_Occurence	N4BP2, TSC2	7/19
EGFR	NF1	0.013019339	4.248750205	54	9	14	8	Co_Occurence	EGFR, NF1	9/22
POLD1	NF1	0.013019339	4.248750205	54	9	14	8	Co_Occurence	NF1, POLD1	9/22
NIN	COL3A1	0.013760383	5.171237302	62	6	7	10	Co_Occurence	COL3A1, NIN	6/17
SETD1B	COL3A1	0.013760383	5.171237302	62	6	7	10	Co_Occurence	COL3A1, SETD1B	6/17
SETD1B	DCC	0.013760383	5.171237302	62	6	7	10	Co_Occurence	DCC, SETD1B	6/17
NIN	RET	0.013760383	5.171237302	62	6	7	10	Co_Occurence	NIN, RET	6/17
MTOR	STAG1	0.013952111	4.582739077	59	7	8	11	Co_Occurence	MTOR, STAG1	7/19
FANCA	ZMYM3	0.013952111	4.582739077	59	7	8	11	Co_Occurence	FANCA, ZMYM3	7/19
MTOR	ZMYM3	0.013952111	4.582739077	59	7	8	11	Co_Occurence	MTOR, ZMYM3	7/19
MTOR	CREBBP	0.014342635	4.069787908	54	9	13	9	Co_Occurence	CREBBP, MTOR	9/22

RANBP2	FAT1	0.014691094	4.247183439	52	9	18	6	Co_Occurence	FAT1, RANBP2	9/24
ZMYM3	FAT1	0.014691094	4.247183439	52	9	18	6	Co_Occurence	FAT1, ZMYM3	9/24
RANBP2	BRCA2	0.014704462	5.03012584	62	6	8	9	Co_Occurence	BRCA2, RANBP2	6/17
STAG1	BRCA2	0.014704462	5.03012584	62	6	8	9	Co_Occurence	BRCA2, STAG1	6/17
NCOR1	COL1A1	0.014704462	5.03012584	62	6	8	9	Co_Occurence	COL1A1, NCOR1	6/17
TPR	COL1A1	0.014704462	5.03012584	62	6	8	9	Co_Occurence	COL1A1, TPR	6/17
RANBP2	N4BP2	0.014704462	5.03012584	62	6	8	9	Co_Occurence	N4BP2, RANBP2	6/17
TPR	N4BP2	0.014704462	5.03012584	62	6	8	9	Co_Occurence	N4BP2, TPR	6/17
ZMYM3	N4BP2	0.014704462	5.03012584	62	6	8	9	Co_Occurence	N4BP2, ZMYM3	6/17
TPR	NSD1	0.014704462	5.03012584	62	6	8	9	Co_Occurence	NSD1, TPR	6/17
NIN	EGFR	0.014792786	4.482555507	59	7	10	9	Co_Occurence	EGFR, NIN	7/19
TERT	EGFR	0.014792786	4.482555507	59	7	10	9	Co_Occurence	EGFR, TERT	7/19
TRRAP	EGFR	0.014792786	4.482555507	59	7	10	9	Co_Occurence	EGFR, TRRAP	7/19
NIN	MYH9	0.014792786	4.482555507	59	7	10	9	Co_Occurence	MYH9, NIN	7/19
KAT6A	POLD1	0.014792786	4.482555507	59	7	10	9	Co_Occurence	KAT6A, POLD1	7/19
SETD1B	SPEN	0.014792786	4.482555507	59	7	10	9	Co_Occurence	SETD1B, SPEN	7/19
TRRAP	SPEN	0.014792786	4.482555507	59	7	10	9	Co_Occurence	SPEN, TRRAP	7/19
KAT6A	TNC	0.014792786	4.482555507	59	7	10	9	Co_Occurence	KAT6A, TNC	7/19
NIN	TNC	0.014792786	4.482555507	59	7	10	9	Co_Occurence	NIN, TNC	7/19
TERT	TNC	0.014792786	4.482555507	59	7	10	9	Co_Occurence	TERT, TNC	7/19
NCOR2	ARID1B	0.015514323	3.937216712	54	9	11	11	Co_Occurence	ARID1B, NCOR2	9/22
UBR5	NCOR2	0.015514323	3.937216712	54	9	11	11	Co_Occurence	NCOR2, UBR5	9/22
BRCA2	DROSHA	0.017160181	4.86165515	56	8	15	6	Co_Occurence	BRCA2, DROSHA	8/21
COL1A1	NF1	0.017160181	4.86165515	56	8	15	6	Co_Occurence	COL1A1, NF1	8/21
NSD1	NF1	0.017160181	4.86165515	56	8	15	6	Co_Occurence	NF1, NSD1	8/21
BRCA2	SMARCA4	0.017160181	4.86165515	56	8	15	6	Co_Occurence	BRCA2, SMARCA4	8/21
NSD1	SMARCA4	0.017160181	4.86165515	56	8	15	6	Co_Occurence	NSD1, SMARCA4	8/21
MYH9	FAT1	0.017252079	4.200767139	51	10	17	7	Co_Occurence	FAT1, MYH9	10/24
BRCA2	ARID1B	0.01734254	4.362442279	58	7	13	7	Co_Occurence	ARID1B, BRCA2	7/20
COL1A1	UBR5	0.01734254	4.362442279	58	7	13	7	Co_Occurence	COL1A1, UBR5	7/20
RNF213	POLE	0.017787538	3.466136801	47	12	10	16	Co_Occurence	POLE, RNF213	12/26
DNMT1	KAT6B	0.01854791	4.501512839	57	8	9	11	Co_Occurence	DNMT1, KAT6B	8/20
AXIN1	SPEN	0.01854791	4.501512839	57	8	9	11	Co_Occurence	AXIN1, SPEN	8/20
MTOR	FANCA	0.018741236	4.457191761	57	8	10	10	Co_Occurence	FANCA, MTOR	8/20
NCOR2	DROSHA	0.018755803	3.92277974	52	10	13	10	Co_Occurence	DROSHA, NCOR2	10/23
FLNA	SMARCA4	0.018755803	3.92277974	52	10	13	10	Co_Occurence	FLNA, SMARCA4	10/23
NCOR2	SMARCA4	0.018755803	3.92277974	52	10	13	10	Co_Occurence	NCOR2, SMARCA4	10/23
CREBBP	STAG1	0.018785104	4.470464585	56	8	7	14	Co_Occurence	CREBBP, STAG1	8/21
FANCA	SMARCA4	0.019106498	3.715578962	53	9	14	9	Co_Occurence	FANCA, SMARCA4	9/23

KAT6B	RNF213	0.019267268	3.894668651	50	10	18	7	Co_Occurence	KAT6B, RNF213	10/25
TNC	RNF213	0.019267268	3.894668651	50	10	18	7	Co_Occurence	RNF213, TNC	10/25
COL3A1	MYH9	0.01949202	4.637654211	61	6	11	7	Co_Occurence	COL3A1, MYH9	6/18
DCC	POLD1	0.01949202	4.637654211	61	6	11	7	Co_Occurence	DCC, POLD1	6/18
COL3A1	SPEN	0.01949202	4.637654211	61	6	11	7	Co_Occurence	COL3A1, SPEN	6/18
FAT1	COL3A1	0.021114779	4.371787089	53	8	5	19	Co_Occurence	COL3A1, FAT1	8/24
FAT1	DCC	0.021114779	4.371787089	53	8	5	19	Co_Occurence	DCC, FAT1	8/24
FAT1	RET	0.021114779	4.371787089	53	8	5	19	Co_Occurence	FAT1, RET	8/24
BRCA2	KAT6A	0.021313008	4.466698789	61	6	10	8	Co_Occurence	BRCA2, KAT6A	6/18
NSD1	KAT6A	0.021313008	4.466698789	61	6	10	8	Co_Occurence	KAT6A, NSD1	6/18
BRCA2	SETD1B	0.021313008	4.466698789	61	6	10	8	Co_Occurence	BRCA2, SETD1B	6/18
COL1A1	SETD1B	0.021313008	4.466698789	61	6	10	8	Co_Occurence	COL1A1, SETD1B	6/18
N4BP2	SETD1B	0.021313008	4.466698789	61	6	10	8	Co_Occurence	N4BP2, SETD1B	6/18
BRCA2	TERT	0.021313008	4.466698789	61	6	10	8	Co_Occurence	BRCA2, TERT	6/18
COL1A1	TERT	0.021313008	4.466698789	61	6	10	8	Co_Occurence	COL1A1, TERT	6/18
NSD1	TERT	0.021313008	4.466698789	61	6	10	8	Co_Occurence	NSD1, TERT	6/18
COL1A1	TRRAP	0.021313008	4.466698789	61	6	10	8	Co_Occurence	COL1A1, TRRAP	6/18
EGFR	ARID1B	0.021562388	4.062546847	56	8	12	9	Co_Occurence	ARID1B, EGFR	8/21
POLD1	BCORL1	0.021562388	4.062546847	56	8	12	9	Co_Occurence	BCORL1, POLD1	8/21
SPEN	BCORL1	0.021562388	4.062546847	56	8	12	9	Co_Occurence	BCORL1, SPEN	8/21
TNC	BCORL1	0.021562388	4.062546847	56	8	12	9	Co_Occurence	BCORL1, TNC	8/21
SPEN	FLNA	0.021562388	4.062546847	56	8	12	9	Co_Occurence	FLNA, SPEN	8/21
SPEN	NCOR2	0.021562388	4.062546847	56	8	12	9	Co_Occurence	NCOR2, SPEN	8/21
TNC	UBR5	0.021562388	4.062546847	56	8	12	9	Co_Occurence	TNC, UBR5	8/21
SMARCA4	NCOR1	0.021831834	4.105165698	55	8	7	15	Co_Occurence	NCOR1, SMARCA4	8/22
NF1	STAG1	0.021831834	4.105165698	55	8	7	15	Co_Occurence	NF1, STAG1	8/22
NF1	ZMYM3	0.021831834	4.105165698	55	8	7	15	Co_Occurence	NF1, ZMYM3	8/22
STAG1	NCOR1	0.021943871	4.412490105	61	6	9	9	Co_Occurence	NCOR1, STAG1	6/18
TPR	NCOR1	0.021943871	4.412490105	61	6	9	9	Co_Occurence	NCOR1, TPR	6/18
TPR	RANBP2	0.021943871	4.412490105	61	6	9	9	Co_Occurence	RANBP2, TPR	6/18
TPR	STAG1	0.021943871	4.412490105	61	6	9	9	Co_Occurence	STAG1, TPR	6/18
ZMYM3	TPR	0.021943871	4.412490105	61	6	9	9	Co_Occurence	TPR, ZMYM3	6/18
AXIN1	FANCA	0.022221088	3.989820292	56	8	10	11	Co_Occurence	AXIN1, FANCA	8/21
POLE	KAT6A	0.024520668	3.852270368	55	8	8	14	Co_Occurence	KAT6A, POLE	8/22
CREBBP	NIN	0.024520668	3.852270368	55	8	8	14	Co_Occurence	CREBBP, NIN	8/22
POLE	NIN	0.024520668	3.852270368	55	8	8	14	Co_Occurence	NIN, POLE	8/22
RET	COL3A1	0.024660062	4.867283919	64	5	8	8	Co_Occurence	COL3A1, RET	5/16
RNF213	RET	0.025190295	4.080531412	52	8	5	20	Co_Occurence	RET, RNF213	8/25
FANCA	COL3A1	0.026775974	4.191164984	60	6	7	12	Co_Occurence	COL3A1, FANCA	6/19

FANCA	RET	0.026775974	4.191164984	60	6	7	12	Co_Occurence	FANCA, RET	6/19
MTOR	UBR5	0.028149383	3.599169666	55	8	12	10	Co_Occurence	MTOR, UBR5	8/22
TSC2	BIRC6	0.028645712	3.569872785	55	8	11	11	Co_Occurence	BIRC6, TSC2	8/22
TSC2	DNMT1	0.028645712	3.569872785	55	8	11	11	Co_Occurence	DNMT1, TSC2	8/22
COL1A1	EGFR	0.029810955	4.003788653	60	6	11	8	Co_Occurence	COL1A1, EGFR	6/19
BRCA2	MYH9	0.029810955	4.003788653	60	6	11	8	Co_Occurence	BRCA2, MYH9	6/19
NSD1	POLD1	0.029810955	4.003788653	60	6	11	8	Co_Occurence	NSD1, POLD1	6/19
NF1	KAT6A	0.030666576	3.535893995	54	8	8	15	Co_Occurence	KAT6A, NF1	8/23
DROSHA	NIN	0.030666576	3.535893995	54	8	8	15	Co_Occurence	DROSHA, NIN	8/23
DROSHA	TERT	0.030666576	3.535893995	54	8	8	15	Co_Occurence	DROSHA, TERT	8/23
SMARCA4	TRRAP	0.030666576	3.535893995	54	8	8	15	Co_Occurence	SMARCA4, TRRAP	8/23
RNF213	NCOR1	0.030722572	3.951019473	51	9	6	19	Co_Occurence	NCOR1, RNF213	9/25
RNF213	TPR	0.030722572	3.951019473	51	9	6	19	Co_Occurence	RNF213, TPR	9/25
RNF213	ZMYM3	0.030722572	3.951019473	51	9	6	19	Co_Occurence	RNF213, ZMYM3	9/25
KAT6A	NCOR1	0.031406813	3.91633113	60	6	9	10	Co_Occurence	KAT6A, NCOR1	6/19
TERT	RANBP2	0.031406813	3.91633113	60	6	9	10	Co_Occurence	RANBP2, TERT	6/19
TERT	TPR	0.031406813	3.91633113	60	6	9	10	Co_Occurence	TERT, TPR	6/19
NIN	ZMYM3	0.031406813	3.91633113	60	6	9	10	Co_Occurence	NIN, ZMYM3	6/19
COL3A1	CREBBP	0.033333993	4.337458546	57	7	15	6	Co_Occurence	COL3A1, CREBBP	7/21
RET	CREBBP	0.033333993	4.337458546	57	7	15	6	Co_Occurence	CREBBP, RET	7/21
DCC	POLE	0.033333993	4.337458546	57	7	15	6	Co_Occurence	DCC, POLE	7/21
DNMT1	POLE	0.034103024	3.602881622	53	9	13	10	Co_Occurence	DNMT1, POLE	9/23
CREBBP	POLD1	0.034188079	3.369916803	54	8	9	14	Co_Occurence	CREBBP, POLD1	8/23
POLE	POLD1	0.034188079	3.369916803	54	8	9	14	Co_Occurence	POLD1, POLE	8/23
POLE	SPEN	0.034188079	3.369916803	54	8	9	14	Co_Occurence	POLE, SPEN	8/23
AXIN1	RANBP2	0.034802901	4.138762364	58	7	8	12	Co_Occurence	AXIN1, RANBP2	7/20
BIRC6	RANBP2	0.034802901	4.138762364	58	7	8	12	Co_Occurence	BIRC6, RANBP2	7/20
DNMT1	RANBP2	0.034802901	4.138762364	58	7	8	12	Co_Occurence	DNMT1, RANBP2	7/20
AXIN1	STAG1	0.034802901	4.138762364	58	7	8	12	Co_Occurence	AXIN1, STAG1	7/20
BIRC6	STAG1	0.034802901	4.138762364	58	7	8	12	Co_Occurence	BIRC6, STAG1	7/20
DNMT1	STAG1	0.034802901	4.138762364	58	7	8	12	Co_Occurence	DNMT1, STAG1	7/20
DNMT1	TPR	0.034802901	4.138762364	58	7	8	12	Co_Occurence	DNMT1, TPR	7/20
TSC2	TPR	0.034802901	4.138762364	58	7	8	12	Co_Occurence	TPR, TSC2	7/20
BIRC6	ZMYM3	0.034802901	4.138762364	58	7	8	12	Co_Occurence	BIRC6, ZMYM3	7/20
DCC	COL1A1	0.034956459	4.27241002	63	5	9	8	Co_Occurence	COL1A1, DCC	5/17
RET	COL1A1	0.034956459	4.27241002	63	5	9	8	Co_Occurence	COL1A1, RET	5/17
DCC	N4BP2	0.034956459	4.27241002	63	5	9	8	Co_Occurence	DCC, N4BP2	5/17
RET	NSD1	0.034956459	4.27241002	63	5	9	8	Co_Occurence	NSD1, RET	5/17
TRRAP	FANCA	0.035800815	4.015340042	58	7	11	9	Co_Occurence	FANCA, TRRAP	7/20

NIN	MTOR	0.035800815	4.015340042	58	7	11	9	Co_Occurence	MTOR, NIN	7/20
SPEN	KAT6B	0.036159144	3.975865286	58	7	10	10	Co_Occurence	KAT6B, SPEN	7/20
COL3A1	DROSHA	0.036618571	4.001753551	56	7	16	6	Co_Occurence	COL3A1, DROSHA	7/22
DCC	DROSHA	0.036618571	4.001753551	56	7	16	6	Co_Occurence	DCC, DROSHA	7/22
RET	NF1	0.036618571	4.001753551	56	7	16	6	Co_Occurence	NF1, RET	7/22
COL3A1	SMARCA4	0.036618571	4.001753551	56	7	16	6	Co_Occurence	COL3A1, SMARCA4	7/22
DCC	SMARCA4	0.036618571	4.001753551	56	7	16	6	Co_Occurence	DCC, SMARCA4	7/22
BIRC6	DROSHA	0.038359698	3.287945644	52	9	14	10	Co_Occurence	BIRC6, DROSHA	9/24
AXIN1	NF1	0.038359698	3.287945644	52	9	14	10	Co_Occurence	AXIN1, NF1	9/24
TSC2	SMARCA4	0.038359698	3.287945644	52	9	14	10	Co_Occurence	SMARCA4, TSC2	9/24
NCOR1	BCORL1	0.03902615	3.761920722	57	7	13	8	Co_Occurence	BCORL1, NCOR1	7/21
RANBP2	NCOR2	0.03902615	3.761920722	57	7	13	8	Co_Occurence	NCOR2, RANBP2	7/21
STAG1	NCOR2	0.03902615	3.761920722	57	7	13	8	Co_Occurence	NCOR2, STAG1	7/21
TPR	NCOR2	0.03902615	3.761920722	57	7	13	8	Co_Occurence	NCOR2, TPR	7/21
ZMYM3	NCOR2	0.03902615	3.761920722	57	7	13	8	Co_Occurence	NCOR2, ZMYM3	7/21
KAT6A	RNF213	0.039156525	3.329009159	50	9	19	7	Co_Occurence	KAT6A, RNF213	9/26
NIN	RNF213	0.039156525	3.329009159	50	9	19	7	Co_Occurence	NIN, RNF213	9/26
CREBBP	ARID1B	0.039661573	3.219815768	52	9	11	13	Co_Occurence	ARID1B, CREBBP	9/24
CREBBP	FLNA	0.039661573	3.219815768	52	9	11	13	Co_Occurence	CREBBP, FLNA	9/24
POLE	NCOR2	0.039661573	3.219815768	52	9	11	13	Co_Occurence	NCOR2, POLE	9/24
AXIN1	KAT6A	0.041123026	3.624722565	57	7	9	12	Co_Occurence	AXIN1, KAT6A	7/21
DNMT1	NIN	0.041123026	3.624722565	57	7	9	12	Co_Occurence	DNMT1, NIN	7/21
DNMT1	TRRAP	0.041123026	3.624722565	57	7	9	12	Co_Occurence	DNMT1, TRRAP	7/21
BRCA2	CREBBP	0.041421311	3.663632262	56	7	15	7	Co_Occurence	BRCA2, CREBBP	7/22
N4BP2	POLE	0.041421311	3.663632262	56	7	15	7	Co_Occurence	N4BP2, POLE	7/22
FANCA	KAT6B	0.042250741	3.559842188	57	7	10	11	Co_Occurence	FANCA, KAT6B	7/21
MTOR	KAT6B	0.042250741	3.559842188	57	7	10	11	Co_Occurence	KAT6B, MTOR	7/21
MTOR	TNC	0.042250741	3.559842188	57	7	10	11	Co_Occurence	MTOR, TNC	7/21
FAT1	EGFR	0.045382644	3.07798646	50	9	8	18	Co_Occurence	EGFR, FAT1	9/26
NCOR1	COL3A1	0.047747195	3.794313773	62	5	8	10	Co_Occurence	COL3A1, NCOR1	5/18
NCOR1	RET	0.047747195	3.794313773	62	5	8	10	Co_Occurence	NCOR1, RET	5/18
RANBP2	RET	0.047747195	3.794313773	62	5	8	10	Co_Occurence	RANBP2, RET	5/18
ZMYM3	RET	0.047747195	3.794313773	62	5	8	10	Co_Occurence	RET, ZMYM3	5/18
FAT1	AXIN1	0.047810386	3.153359657	49	10	9	17	Co_Occurence	AXIN1, FAT1	10/26
FAT1	BIRC6	0.047810386	3.153359657	49	10	9	17	Co_Occurence	BIRC6, FAT1	10/26
FAT1	DNMT1	0.047810386	3.153359657	49	10	9	17	Co_Occurence	DNMT1, FAT1	10/26
UBR5	SMARCA4	0.048340738	2.936838868	51	9	14	11	Co_Occurence	SMARCA4, UBR5	9/25
POLE	DROSHA	0.048390021	3.155185364	50	10	13	12	Co_Occurence	DROSHA, POLE	10/25
COL1A1	DROSHA	0.048892241	3.37829143	55	7	16	7	Co_Occurence	COL1A1, DROSHA	7/23

N4BP2	SMARCA4	0.048892241	3.37829143	55	7	16	7	Co_Occurence	N4BP2, SMARCA4	7/23
KAT6A	BCORL1	0.049510532	3.293256105	56	7	13	9	Co_Occurence	BCORL1, KAT6A	7/22
KAT6A	NCOR2	0.049510532	3.293256105	56	7	13	9	Co_Occurence	KAT6A, NCOR2	7/22
KAT6A	UBR5	0.049510532	3.293256105	56	7	13	9	Co_Occurence	KAT6A, UBR5	7/22
AXIN1	EGFR	0.051836321	3.21216243	56	7	10	12	Co_Occurence	AXIN1, EGFR	7/22
BIRC6	EGFR	0.051836321	3.21216243	56	7	10	12	Co_Occurence	BIRC6, EGFR	7/22
BIRC6	KAT6B	0.051836321	3.21216243	56	7	10	12	Co_Occurence	BIRC6, KAT6B	7/22
AXIN1	POLD1	0.051836321	3.21216243	56	7	10	12	Co_Occurence	AXIN1, POLD1	7/22
DNMT1	SPEN	0.051836321	3.21216243	56	7	10	12	Co_Occurence	DNMT1, SPEN	7/22
TSC2	SPEN	0.051836321	3.21216243	56	7	10	12	Co_Occurence	SPEN, TSC2	7/22
SMARCA4	DROSHA	0.05418325	2.858604662	49	10	13	13	Co_Occurence	DROSHA, SMARCA4	10/26
SMARCA4	NF1	0.05418325	2.858604662	49	10	13	13	Co_Occurence	NF1, SMARCA4	10/26
FAT1	BRCA2	0.055451176	3.585692659	52	8	6	19	Co_Occurence	BRCA2, FAT1	8/25
TPR	CREBBP	0.055511125	3.1563312	55	7	15	8	Co_Occurence	CREBBP, TPR	7/23
NCOR1	POLE	0.055511125	3.1563312	55	7	15	8	Co_Occurence	NCOR1, POLE	7/23
N4BP2	RNF213	0.058906421	3.345088394	51	8	20	6	Co_Occurence	N4BP2, RNF213	8/26
FAT1	POLE	0.060504498	2.896315382	47	11	11	16	Co_Occurence	FAT1, POLE	11/27
AXIN1	ARID1B	0.061492228	3.21893781	54	8	12	11	Co_Occurence	ARID1B, AXIN1	8/23
DNMT1	ARID1B	0.061492228	3.21893781	54	8	12	11	Co_Occurence	ARID1B, DNMT1	8/23
AXIN1	UBR5	0.061492228	3.21893781	54	8	12	11	Co_Occurence	AXIN1, UBR5	8/23
BIRC6	UBR5	0.061492228	3.21893781	54	8	12	11	Co_Occurence	BIRC6, UBR5	8/23
DNMT1	UBR5	0.061492228	3.21893781	54	8	12	11	Co_Occurence	DNMT1, UBR5	8/23
COL3A1	KAT6A	0.063195745	3.401589	61	5	11	8	Co_Occurence	COL3A1, KAT6A	5/19
RET	SETD1B	0.063195745	3.401589	61	5	11	8	Co_Occurence	RET, SETD1B	5/19
COL3A1	TERT	0.063195745	3.401589	61	5	11	8	Co_Occurence	COL3A1, TERT	5/19
RET	AXIN1	0.063486266	3.812084203	59	6	13	7	Co_Occurence	AXIN1, RET	6/20
COL3A1	BIRC6	0.063486266	3.812084203	59	6	13	7	Co_Occurence	BIRC6, COL3A1	6/20
RET	DNMT1	0.063486266	3.812084203	59	6	13	7	Co_Occurence	DNMT1, RET	6/20
SPEN	DROSHA	0.063851714	3.091658617	53	8	15	9	Co_Occurence	DROSHA, SPEN	8/24
TNC	DROSHA	0.063851714	3.091658617	53	8	15	9	Co_Occurence	DROSHA, TNC	8/24
KAT6B	SMARCA4	0.063851714	3.091658617	53	8	15	9	Co_Occurence	KAT6B, SMARCA4	8/24
POLD1	SMARCA4	0.063851714	3.091658617	53	8	15	9	Co_Occurence	POLD1, SMARCA4	8/24
RNF213	CREBBP	0.06563735	2.6713149	46	11	11	17	Co_Occurence	CREBBP, RNF213	11/28
POLE	FANCA	0.066689027	2.982792022	53	8	10	14	Co_Occurence	FANCA, POLE	8/24
POLE	MTOR	0.066689027	2.982792022	53	8	10	14	Co_Occurence	MTOR, POLE	8/24
TPR	FAT1	0.067034983	3.022489779	51	8	19	7	Co_Occurence	FAT1, TPR	8/26
EGFR	NCOR1	0.068043857	3.508832567	59	6	9	11	Co_Occurence	EGFR, NCOR1	6/20
KAT6B	RANBP2	0.068043857	3.508832567	59	6	9	11	Co_Occurence	KAT6B, RANBP2	6/20
EGFR	STAG1	0.068043857	3.508832567	59	6	9	11	Co_Occurence	EGFR, STAG1	6/20

EGFR	TPR	0.068043857	3.508832567	59	6	9	11	Co_Occurence	EGFR, TPR	6/20
EGFR	ZMYM3	0.068043857	3.508832567	59	6	9	11	Co_Occurence	EGFR, ZMYM3	6/20
NCOR2	COL3A1	0.068991809	3.486179897	58	6	7	14	Co_Occurence	COL3A1, NCOR2	6/21
UBR5	RET	0.068991809	3.486179897	58	6	7	14	Co_Occurence	RET, UBR5	6/21
NCOR2	BCORL1	0.069207182	2.901486269	53	8	12	12	Co_Occurence	BCORL1, NCOR2	8/24
NCOR2	FLNA	0.069207182	2.901486269	53	8	12	12	Co_Occurence	FLNA, NCOR2	8/24
BRCA2	AXIN1	0.073759664	3.288122748	58	6	13	8	Co_Occurence	AXIN1, BRCA2	6/21
COL1A1	AXIN1	0.073759664	3.288122748	58	6	13	8	Co_Occurence	AXIN1, COL1A1	6/21
COL1A1	BIRC6	0.073759664	3.288122748	58	6	13	8	Co_Occurence	BIRC6, COL1A1	6/21
N4BP2	BIRC6	0.073759664	3.288122748	58	6	13	8	Co_Occurence	BIRC6, N4BP2	6/21
MTOR	DROSHA	0.076903353	2.735534465	52	8	15	10	Co_Occurence	DROSHA, MTOR	8/25
NCOR1	MTOR	0.077314184	3.168224667	58	6	12	9	Co_Occurence	MTOR, NCOR1	6/21
RANBP2	MTOR	0.077314184	3.168224667	58	6	12	9	Co_Occurence	MTOR, RANBP2	6/21
TPR	MTOR	0.077314184	3.168224667	58	6	12	9	Co_Occurence	MTOR, TPR	6/21
TRRAP	POLD1	0.079202811	3.111521763	58	6	11	10	Co_Occurence	POLD1, TRRAP	6/21
DNMT1	CREBBP	0.080387617	2.66546938	52	8	14	11	Co_Occurence	CREBBP, DNMT1	8/25
BIRC6	POLE	0.080387617	2.66546938	52	8	14	11	Co_Occurence	BIRC6, POLE	8/25
SPEN	RNF213	0.081113278	2.861121801	49	9	19	8	Co_Occurence	RNF213, SPEN	9/27
N4BP2	BCORL1	0.084884659	3.005531842	57	6	14	8	Co_Occurence	BCORL1, N4BP2	6/22
BRCA2	NCOR2	0.084884659	3.005531842	57	6	14	8	Co_Occurence	BRCA2, NCOR2	6/22
COL1A1	NCOR2	0.084884659	3.005531842	57	6	14	8	Co_Occurence	COL1A1, NCOR2	6/22
BRCA2	UBR5	0.084884659	3.005531842	57	6	14	8	Co_Occurence	BRCA2, UBR5	6/22
N4BP2	UBR5	0.084884659	3.005531842	57	6	14	8	Co_Occurence	N4BP2, UBR5	6/22
DCC	CREBBP	0.089210011	2.954481713	56	6	16	7	Co_Occurence	CREBBP, DCC	6/23
COL3A1	POLE	0.089210011	2.954481713	56	6	16	7	Co_Occurence	COL3A1, POLE	6/23
RET	POLE	0.089210011	2.954481713	56	6	16	7	Co_Occurence	POLE, RET	6/23
NCOR1	BIRC6	0.090643635	2.879036671	57	6	13	9	Co_Occurence	BIRC6, NCOR1	6/22
NCOR1	TSC2	0.090643635	2.879036671	57	6	13	9	Co_Occurence	NCOR1, TSC2	6/22
RANBP2	TSC2	0.090643635	2.879036671	57	6	13	9	Co_Occurence	RANBP2, TSC2	6/22
MYH9	EGFR	0.095569536	2.785637205	57	6	11	11	Co_Occurence	EGFR, MYH9	6/22
POLD1	EGFR	0.095569536	2.785637205	57	6	11	11	Co_Occurence	EGFR, POLD1	6/22
SPEN	EGFR	0.095569536	2.785637205	57	6	11	11	Co_Occurence	EGFR, SPEN	6/22
POLD1	KAT6B	0.095569536	2.785637205	57	6	11	11	Co_Occurence	KAT6B, POLD1	6/22
TNC	MYH9	0.095569536	2.785637205	57	6	11	11	Co_Occurence	MYH9, TNC	6/22
TNC	POLD1	0.095569536	2.785637205	57	6	11	11	Co_Occurence	POLD1, TNC	6/22
RET	SMARCA4	0.103815874	2.734766095	55	6	17	7	Co_Occurence	RET, SMARCA4	6/24
RANBP2	DROSHA	0.105110046	2.9096627	54	7	16	8	Co_Occurence	DROSHA, RANBP2	7/24
STAG1	DROSHA	0.105110046	2.9096627	54	7	16	8	Co_Occurence	DROSHA, STAG1	7/24
STAG1	SMARCA4	0.105110046	2.9096627	54	7	16	8	Co_Occurence	SMARCA4, STAG1	7/24

POLD1	ARID1B	0.105600208	2.916994551	55	7	13	10	Co_Occurence	ARID1B, POLD1	7/23
EGFR	FLNA	0.105600208	2.916994551	55	7	13	10	Co_Occurence	EGFR, FLNA	7/23
TNC	FLNA	0.105600208	2.916994551	55	7	13	10	Co_Occurence	FLNA, TNC	7/23
EGFR	UBR5	0.105600208	2.916994551	55	7	13	10	Co_Occurence	EGFR, UBR5	7/23
MYH9	UBR5	0.105600208	2.916994551	55	7	13	10	Co_Occurence	MYH9, UBR5	7/23
POLD1	UBR5	0.105600208	2.916994551	55	7	13	10	Co_Occurence	POLD1, UBR5	7/23
DNMT1	FANCA	0.106702804	2.873879092	55	7	11	12	Co_Occurence	DNMT1, FANCA	7/23
TSC2	FANCA	0.106702804	2.873879092	55	7	11	12	Co_Occurence	FANCA, TSC2	7/23
KAT6A	CREBBP	0.109969264	2.760920595	54	7	15	9	Co_Occurence	CREBBP, KAT6A	7/24
NCOR2	MTOR	0.116626167	2.608638505	54	7	11	13	Co_Occurence	MTOR, NCOR2	7/24
KAT6A	DROSHA	0.121076048	2.544088552	53	7	16	9	Co_Occurence	DROSHA, KAT6A	7/25
TRRAP	DROSHA	0.121076048	2.544088552	53	7	16	9	Co_Occurence	DROSHA, TRRAP	7/25
NIN	SMARCA4	0.121076048	2.544088552	53	7	16	9	Co_Occurence	NIN, SMARCA4	7/25
TERT	SMARCA4	0.121076048	2.544088552	53	7	16	9	Co_Occurence	SMARCA4, TERT	7/25
EGFR	COL3A1	0.123864672	3.073270271	60	5	8	12	Co_Occurence	COL3A1, EGFR	5/20
TNC	DCC	0.123864672	3.073270271	60	5	8	12	Co_Occurence	DCC, TNC	5/20
MYH9	RET	0.123864672	3.073270271	60	5	8	12	Co_Occurence	MYH9, RET	5/20
POLD1	RET	0.123864672	3.073270271	60	5	8	12	Co_Occurence	POLD1, RET	5/20
TNC	RET	0.123864672	3.073270271	60	5	8	12	Co_Occurence	RET, TNC	5/20
FAT1	N4BP2	0.12572482	2.519022182	51	7	7	20	Co_Occurence	FAT1, N4BP2	7/27
N4BP2	TERT	0.127153846	2.981638388	60	5	11	9	Co_Occurence	N4BP2, TERT	5/20
N4BP2	TRRAP	0.127153846	2.981638388	60	5	11	9	Co_Occurence	N4BP2, TRRAP	5/20
POLE	EGFR	0.127679555	2.443578989	53	7	10	15	Co_Occurence	EGFR, POLE	7/25
POLE	TNC	0.127679555	2.443578989	53	7	10	15	Co_Occurence	POLE, TNC	7/25
STAG1	RANBP2	0.128301284	2.952306411	60	5	10	10	Co_Occurence	RANBP2, STAG1	5/20
ZMYM3	RANBP2	0.128301284	2.952306411	60	5	10	10	Co_Occurence	RANBP2, ZMYM3	5/20
NF1	BIRC6	0.14092265	2.443345143	51	8	11	15	Co_Occurence	BIRC6, NF1	8/26
N4BP2	EGFR	0.142503329	2.69265571	59	5	12	9	Co_Occurence	EGFR, N4BP2	5/21
N4BP2	KAT6B	0.142503329	2.69265571	59	5	12	9	Co_Occurence	KAT6B, N4BP2	5/21
COL1A1	MYH9	0.142503329	2.69265571	59	5	12	9	Co_Occurence	COL1A1, MYH9	5/21
N4BP2	MYH9	0.142503329	2.69265571	59	5	12	9	Co_Occurence	MYH9, N4BP2	5/21
COL1A1	POLD1	0.142503329	2.69265571	59	5	12	9	Co_Occurence	COL1A1, POLD1	5/21
N4BP2	POLD1	0.142503329	2.69265571	59	5	12	9	Co_Occurence	N4BP2, POLD1	5/21
NSD1	SPEN	0.142503329	2.69265571	59	5	12	9	Co_Occurence	NSD1, SPEN	5/21
BRCA2	TNC	0.142503329	2.69265571	59	5	12	9	Co_Occurence	BRCA2, TNC	5/21
N4BP2	TNC	0.142503329	2.69265571	59	5	12	9	Co_Occurence	N4BP2, TNC	5/21
NSD1	TNC	0.142503329	2.69265571	59	5	12	9	Co_Occurence	NSD1, TNC	5/21
POLE	ARID1B	0.143033278	2.400311763	51	8	12	14	Co_Occurence	ARID1B, POLE	8/26
KAT6A	RANBP2	0.145512848	2.644440473	59	5	10	11	Co_Occurence	KAT6A, RANBP2	5/21

KAT6A	TPR	0.145512848	2.644440473	59	5	10	11	Co_Occurence	KAT6A, TPR	5/21
SETD1B	TPR	0.145512848	2.644440473	59	5	10	11	Co_Occurence	SETD1B, TPR	5/21
DNMT1	COL3A1	0.153884594	2.555091186	58	5	8	14	Co_Occurence	COL3A1, DNMT1	5/22
BCORL1	SMARCA4	0.157104127	2.199312414	50	8	15	12	Co_Occurence	BCORL1, SMARCA4	8/27
COL1A1	FANCA	0.162608802	2.4476023	58	5	13	9	Co_Occurence	COL1A1, FANCA	5/22
BRCA2	MTOR	0.162608802	2.4476023	58	5	13	9	Co_Occurence	BRCA2, MTOR	5/22
COL1A1	MTOR	0.162608802	2.4476023	58	5	13	9	Co_Occurence	COL1A1, MTOR	5/22
N4BP2	MTOR	0.162608802	2.4476023	58	5	13	9	Co_Occurence	MTOR, N4BP2	5/22
ZMYM3	POLD1	0.168084238	2.387315215	58	5	12	10	Co_Occurence	POLD1, ZMYM3	5/22
ZMYM3	SPEN	0.168084238	2.387315215	58	5	12	10	Co_Occurence	SPEN, ZMYM3	5/22
STAG1	TNC	0.168084238	2.387315215	58	5	12	10	Co_Occurence	STAG1, TNC	5/22
ZMYM3	TNC	0.168084238	2.387315215	58	5	12	10	Co_Occurence	TNC, ZMYM3	5/22
TRRAP	KAT6A	0.169948163	2.367901443	58	5	11	11	Co_Occurence	KAT6A, TRRAP	5/22
COL3A1	UBR5	0.174913083	2.347134151	57	5	15	8	Co_Occurence	COL3A1, UBR5	5/23
DCC	UBR5	0.174913083	2.347134151	57	5	15	8	Co_Occurence	DCC, UBR5	5/23
UBR5	NCOR1	0.175748497	2.630832263	56	6	9	14	Co_Occurence	NCOR1, UBR5	6/23
ARID1B	TPR	0.175748497	2.630832263	56	6	9	14	Co_Occurence	ARID1B, TPR	6/23
N4BP2	CREBBP	0.178088824	2.545355041	55	6	16	8	Co_Occurence	CREBBP, N4BP2	6/24
TERT	BIRC6	0.178680273	2.551124869	56	6	13	10	Co_Occurence	BIRC6, TERT	6/23
TRRAP	BIRC6	0.178680273	2.551124869	56	6	13	10	Co_Occurence	BIRC6, TRRAP	6/23
KAT6A	DNMT1	0.178680273	2.551124869	56	6	13	10	Co_Occurence	DNMT1, KAT6A	6/23
KAT6A	TSC2	0.178680273	2.551124869	56	6	13	10	Co_Occurence	KAT6A, TSC2	6/23
FANCA	EGFR	0.18029091	2.512988952	56	6	11	12	Co_Occurence	EGFR, FANCA	6/23
MTOR	EGFR	0.18029091	2.512988952	56	6	11	12	Co_Occurence	EGFR, MTOR	6/23
MTOR	POLD1	0.18029091	2.512988952	56	6	11	12	Co_Occurence	MTOR, POLD1	6/23
N4BP2	DROSHA	0.189015006	2.354947783	54	6	17	8	Co_Occurence	DROSHA, N4BP2	6/25
ARID1B	NIN	0.190407376	2.33006406	55	6	10	14	Co_Occurence	ARID1B, NIN	6/24
FAT1	SMARCA4	0.193107399	2.01811513	45	10	13	17	Co_Occurence	FAT1, SMARCA4	10/30
TSC2	EGFR	0.193981327	2.281882629	55	6	11	13	Co_Occurence	EGFR, TSC2	6/24
TSC2	POLD1	0.193981327	2.281882629	55	6	11	13	Co_Occurence	POLD1, TSC2	6/24
AXIN1	TNC	0.193981327	2.281882629	55	6	11	13	Co_Occurence	AXIN1, TNC	6/24
BIRC6	TNC	0.193981327	2.281882629	55	6	11	13	Co_Occurence	BIRC6, TNC	6/24
TSC2	TNC	0.193981327	2.281882629	55	6	11	13	Co_Occurence	TNC, TSC2	6/24
COL1A1	RNF213	0.211661003	2.354512671	50	7	21	7	Co_Occurence	COL1A1, RNF213	7/28
TNC	NCOR2	0.214233717	2.083363413	54	6	14	11	Co_Occurence	NCOR2, TNC	6/25
SPEN	UBR5	0.214233717	2.083363413	54	6	14	11	Co_Occurence	SPEN, UBR5	6/25
COL1A1	COL3A1	0.214508624	2.714910132	62	4	9	10	Co_Occurence	COL1A1, COL3A1	4/19
DNMT1	MTOR	0.217413129	2.057027763	54	6	12	13	Co_Occurence	DNMT1, MTOR	6/25
SPEN	NF1	0.220107442	2.250567624	52	7	16	10	Co_Occurence	NF1, SPEN	7/26

TNC	SMARCA4	0.220107442	2.250567624	52	7	16	10	Co_Occurence	SMARCA4, TNC	7/26
COL3A1	STAG1	0.23214982	2.433256658	61	4	11	9	Co_Occurence	COL3A1, STAG1	4/20
RET	STAG1	0.23214982	2.433256658	61	4	11	9	Co_Occurence	RET, STAG1	4/20
N4BP2	BRCA2	0.23397212	2.409264563	61	4	10	10	Co_Occurence	BRCA2, N4BP2	4/20
BIRC6	CREBBP	0.242358215	1.965940964	51	7	15	12	Co_Occurence	BIRC6, CREBBP	7/27
RET	TERT	0.255120472	2.197781547	60	4	12	9	Co_Occurence	RET, TERT	4/21
POLD1	COL3A1	0.282617521	1.998081357	59	4	9	13	Co_Occurence	COL3A1, POLD1	4/22
EGFR	RET	0.282617521	1.998081357	59	4	9	13	Co_Occurence	EGFR, RET	4/22
KAT6B	RET	0.282617521	1.998081357	59	4	9	13	Co_Occurence	KAT6B, RET	4/22
AXIN1	N4BP2	0.289293347	2.236984756	57	5	9	14	Co_Occurence	AXIN1, N4BP2	5/23
DNMT1	N4BP2	0.289293347	2.236984756	57	5	9	14	Co_Occurence	DNMT1, N4BP2	5/23
KAT6A	EGFR	0.295624165	2.136772293	57	5	12	11	Co_Occurence	EGFR, KAT6A	5/23
SETD1B	EGFR	0.295624165	2.136772293	57	5	12	11	Co_Occurence	EGFR, SETD1B	5/23
ARHGEF10	RNF213	0.297422689	0	53	0	28	4	Mutually_Exclusive	ARHGEF10, RNF213	0/32
N4BP2	ARID1B	0.301761885	2.054062878	56	5	15	9	Co_Occurence	ARID1B, N4BP2	5/24
COL1A1	FLNA	0.301761885	2.054062878	56	5	15	9	Co_Occurence	COL1A1, FLNA	5/24
N4BP2	NCOR2	0.301761885	2.054062878	56	5	15	9	Co_Occurence	N4BP2, NCOR2	5/24
NSD1	NCOR2	0.301761885	2.054062878	56	5	15	9	Co_Occurence	NCOR2, NSD1	5/24
KAT6B	EGFR	0.314897241	1.92747572	56	5	12	12	Co_Occurence	EGFR, KAT6B	5/24
ARID1B	STAG1	0.330071981	1.819015865	55	5	10	15	Co_Occurence	ARID1B, STAG1	5/25
UBR5	TPR	0.330071981	1.819015865	55	5	10	15	Co_Occurence	TPR, UBR5	5/25
KAT6A	SMARCA4	0.352522355	1.821153642	52	6	17	10	Co_Occurence	KAT6A, SMARCA4	6/27
EGFR	CREBBP	0.3598911	1.759905687	52	6	16	11	Co_Occurence	CREBBP, EGFR	6/27
DROSHA	EGFR	0.380769212	1.626210794	51	6	11	17	Co_Occurence	DROSHA, EGFR	6/28
SMARCA4	EGFR	0.380769212	1.626210794	51	6	11	17	Co_Occurence	EGFR, SMARCA4	6/28
SMARCA4	SPEN	0.380769212	1.626210794	51	6	11	17	Co_Occurence	SMARCA4, SPEN	6/28
DROSHA	UBR5	0.395485148	1.638685197	49	7	13	16	Co_Occurence	DROSHA, UBR5	7/29
RET	N4BP2	0.442002835	1.652499775	61	3	11	10	Co_Occurence	N4BP2, RET	3/21
EGFR	BRCA2	0.464978844	1.770971905	58	4	10	13	Co_Occurence	BRCA2, EGFR	4/23
N4BP2	NF1	0.512512548	1.625614084	53	5	18	9	Co_Occurence	N4BP2, NF1	5/27
TNC	DNMT1	0.516924099	1.597466089	54	5	14	12	Co_Occurence	DNMT1, TNC	5/26
RANBP2	CREBBP	0.521038578	1.550138674	53	5	17	10	Co_Occurence	CREBBP, RANBP2	5/27
RANBP2	POLE	0.521038578	1.550138674	53	5	17	10	Co_Occurence	POLE, RANBP2	5/27
SPEN	ARID1B	0.533150658	1.465122712	53	5	15	12	Co_Occurence	ARID1B, SPEN	5/27
KAT6B	UBR5	0.533150658	1.465122712	53	5	15	12	Co_Occurence	KAT6B, UBR5	5/27
RANBP2	SMARCA4	0.537024883	1.437867794	52	5	18	10	Co_Occurence	RANBP2, SMARCA4	5/28
CREBBP	ARHGEF10	0.568526041	0	59	0	4	22	Mutually_Exclusive	ARHGEF10, CREBBP	0/26
POLE	ARHGEF10	0.568526041	0	59	0	4	22	Mutually_Exclusive	ARHGEF10, POLE	0/26
ARID1B	ARHGEF10	0.568546784	0	61	0	4	20	Mutually_Exclusive	ARHGEF10, ARID1B	0/24

FLNA	ARHGEF10	0.568546784	0	61	0	4	20	Mutually_Exclusive	ARHGEF10, FLNA	0/24
NCOR2	ARHGEF10	0.568546784	0	61	0	4	20	Mutually_Exclusive	ARHGEF10, NCOR2	0/24
UBR5	ARHGEF10	0.568546784	0	61	0	4	20	Mutually_Exclusive	ARHGEF10, UBR5	0/24
DROSHA	ARHGEF10	0.570393894	0	58	0	4	23	Mutually_Exclusive	ARHGEF10, DROSHA	0/27
NF1	ARHGEF10	0.570393894	0	58	0	4	23	Mutually_Exclusive	ARHGEF10, NF1	0/27
SMARCA4	ARHGEF10	0.570393894	0	58	0	4	23	Mutually_Exclusive	ARHGEF10, SMARCA4	0/27
ARHGEF10	BIRC6	0.570601323	0	62	0	19	4	Mutually_Exclusive	ARHGEF10, BIRC6	0/23
ARHGEF10	DNMT1	0.570601323	0	62	0	19	4	Mutually_Exclusive	ARHGEF10, DNMT1	0/23
ARHGEF10	TSC2	0.570601323	0	62	0	19	4	Mutually_Exclusive	ARHGEF10, TSC2	0/23
ARHGEF10	TERT	0.573040594	1.4593469	66	1	15	3	Co_Occurence	ARHGEF10, TERT	1/18
ARHGEF10	FANCA	0.574132562	0	63	0	18	4	Mutually_Exclusive	ARHGEF10, FANCA	0/22
ARHGEF10	MTOR	0.574132562	0	63	0	18	4	Mutually_Exclusive	ARHGEF10, MTOR	0/22
KAT6B	ARHGEF10	0.579228412	0	64	0	4	17	Mutually_Exclusive	ARHGEF10, KAT6B	0/21
MYH9	ARHGEF10	0.579228412	0	64	0	4	17	Mutually_Exclusive	ARHGEF10, MYH9	0/21
POLD1	ARHGEF10	0.579228412	0	64	0	4	17	Mutually_Exclusive	ARHGEF10, POLD1	0/21
SPEN	ARHGEF10	0.579228412	0	64	0	4	17	Mutually_Exclusive	ARHGEF10, SPEN	0/21
TNC	ARHGEF10	0.579228412	0	64	0	4	17	Mutually_Exclusive	ARHGEF10, TNC	0/21
N4BP2	COL1A1	0.693371123	1.4800373	60	3	11	11	Co_Occurence	COL1A1, N4BP2	3/22
RET	KAT6A	0.703766455	1.356269401	59	3	13	10	Co_Occurence	KAT6A, RET	3/23
RANBP2	COL1A1	0.705995064	1.335990597	59	3	11	12	Co_Occurence	COL1A1, RANBP2	3/23
NCOR1	N4BP2	0.705995064	1.335990597	59	3	11	12	Co_Occurence	N4BP2, NCOR1	3/23
ARID1B	SMARCA4	0.776975103	1.207281595	48	6	17	14	Co_Occurence	ARID1B, SMARCA4	6/31
COL1A1	ARID1B	1	0.867743459	54	3	17	11	Mutually_Exclusive	ARID1B, COL1A1	3/28
SMARCA4	MTOR	1	1.046438827	49	5	13	18	Co_Occurence	MTOR, SMARCA4	5/31
ARHGEF10	NCOR1	1	0	66	0	15	4	Mutually_Exclusive	ARHGEF10, NCOR1	0/19
ARHGEF10	RANBP2	1	0	66	0	15	4	Mutually_Exclusive	ARHGEF10, RANBP2	0/19
ARHGEF10	STAG1	1	0	66	0	15	4	Mutually_Exclusive	ARHGEF10, STAG1	0/19
ARHGEF10	TPR	1	0	66	0	15	4	Mutually_Exclusive	ARHGEF10, TPR	0/19
ARHGEF10	ZMYM3	1	0	66	0	15	4	Mutually_Exclusive	ARHGEF10, ZMYM3	0/19
FAT1	ARHGEF10	1	0.707850831	55	1	3	26	Mutually_Exclusive	ARHGEF10, FAT1	1/29
BCORL1	ARHGEF10	1	1.08663261	62	1	3	19	Co_Occurence	ARHGEF10, BCORL1	1/22
EGFR	ARHGEF10	1	1.348942985	65	1	3	16	Co_Occurence	ARHGEF10, EGFR	1/19
COL3A1	ARHGEF10	1	0	68	0	4	13	Mutually_Exclusive	ARHGEF10, COL3A1	0/17
DCC	ARHGEF10	1	0	68	0	4	13	Mutually_Exclusive	ARHGEF10, DCC	0/17
RET	ARHGEF10	1	0	68	0	4	13	Mutually_Exclusive	ARHGEF10, RET	0/17
EGFR	NCOR2	1	1	52	4	16	13	Mutually_Exclusive	EGFR, NCOR2	4/29
ARHGEF10	AXIN1	1	1.164431209	63	1	18	3	Co_Occurence	ARHGEF10, AXIN1	1/21
ARHGEF10	KAT6A	1	0	65	0	16	4	Mutually_Exclusive	ARHGEF10, KAT6A	0/20
ARHGEF10	NIN	1	0	65	0	16	4	Mutually_Exclusive	ARHGEF10, NIN	0/20

ARHGEF10	SETD1B	1	0	65	0	16	4	Mutually_Exclusive	ARHGEF10, SETD1B	0/20
ARHGEF10	TRRAP	1	0	65	0	16	4	Mutually_Exclusive	ARHGEF10, TRRAP	0/20
ARHGEF10	BRCA2	1	0	67	0	14	4	Mutually_Exclusive	ARHGEF10, BRCA2	0/18
ARHGEF10	COL1A1	1	0	67	0	14	4	Mutually_Exclusive	ARHGEF10, COL1A1	0/18
ARHGEF10	N4BP2	1	0	67	0	14	4	Mutually_Exclusive	ARHGEF10, N4BP2	0/18
ARHGEF10	NSD1	1	0	67	0	14	4	Mutually_Exclusive	ARHGEF10, NSD1	0/18
