# Supplementary material for: Genomic and immunological profiles of small-cell lung cancer between East Asians and Caucasian
Source: Cancer Cell Int. 2022 Apr 29;22:173. doi: 10.1186/s12935-022-02588-w (PMC9052616; doi:10.1186/s12935-022-02588-w)
Supplement: Supplementary file 13 — Additional file 13: Table S4. Related to Additional file 2: Fig. S2b. The results of the co-occurrence/mutual exclusivity of oncogenes/TSGs in the Caucasian cohort (significantly mutated driver genes). [file 12935_2022_2588_MOESM13_ESM.pdf]

Supplementary Table.4 Related to Supplementary Fig. 2b The results of the co-occurrence/mutual exclusivity of oncogenes/TSGs in the Caucasian cohort (significantly mutated driver genes).

gene1	gene2	pValue	oddsRatio	00	11	01	10	Event	pair	event_rati
FANCA	BRCA2	0.026315789	Inf	37	1	0	0	Co_Occurence	BRCA2, FANCA	1/0
ZMYM3	BRCA2	0.026315789	Inf	37	1	0	0	Co_Occurence	BRCA2, ZMYM3	1/0
RNF213	COL3A1	0.026315789	Inf	37	1	0	0	Co_Occurence	COL3A1, RNF213	1/0
ZMYM3	FANCA	0.026315789	Inf	37	1	0	0	Co_Occurence	FANCA, ZMYM3	1/0
RANBP2	KAT6B	0.026315789	Inf	37	1	0	0	Co_Occurence	KAT6B, RANBP2	1/0
SETD1B	NSD1	0.026315789	Inf	37	1	0	0	Co_Occurence	NSD1, SETD1B	1/0
TPR	STAG1	0.026315789	Inf	37	1	0	0	Co_Occurence	STAG1, TPR	1/0
TSC2	COL1A1	0.052631579	Inf	36	1	0	1	Co_Occurence	COL1A1, TSC2	1/1
NCOR2	COL3A1	0.052631579	Inf	36	1	0	1	Co_Occurence	COL3A1, NCOR2	1/1
ARID1B	KAT6A	0.052631579	Inf	36	1	0	1	Co_Occurence	ARID1B, KAT6A	1/1
MTOR	KAT6A	0.052631579	Inf	36	1	0	1	Co_Occurence	KAT6A, MTOR	1/1
ARID1B	KAT6B	0.052631579	Inf	36	1	0	1	Co_Occurence	ARID1B, KAT6B	1/1
DNMT1	N4BP2	0.052631579	Inf	36	1	0	1	Co_Occurence	DNMT1, N4BP2	1/1
AXIN1	NSD1	0.052631579	Inf	36	1	0	1	Co_Occurence	AXIN1, NSD1	1/1
ARID1B	RANBP2	0.052631579	Inf	36	1	0	1	Co_Occurence	ARID1B, RANBP2	1/1
TRRAP	RET	0.052631579	Inf	36	1	0	1	Co_Occurence	RET, TRRAP	1/1
NCOR2	RNF213	0.052631579	Inf	36	1	0	1	Co_Occurence	NCOR2, RNF213	1/1
AXIN1	SETD1B	0.052631579	Inf	36	1	0	1	Co_Occurence	AXIN1, SETD1B	1/1
UBR5	SPEN	0.052631579	Inf	36	1	0	1	Co_Occurence	SPEN, UBR5	1/1
AXIN1	TERT	0.052631579	Inf	36	1	0	1	Co_Occurence	AXIN1, TERT	1/1
DROSHA	CREBBP	0.078947368	Inf	35	1	2	0	Co_Occurence	CREBBP, DROSHA	1/2
NSD1	CREBBP	0.078947368	Inf	35	1	2	0	Co_Occurence	CREBBP, NSD1	1/2
SETD1B	CREBBP	0.078947368	Inf	35	1	2	0	Co_Occurence	CREBBP, SETD1B	1/2
SPEN	CREBBP	0.078947368	Inf	35	1	2	0	Co_Occurence	CREBBP, SPEN	1/2
DCC	EGFR	0.078947368	Inf	35	1	2	0	Co_Occurence	DCC, EGFR	1/2
MTOR	ARID1B	0.103840683	25.10003671	35	1	1	1	Co_Occurence	ARID1B, MTOR	1/2
TERT	ARHGEF10	0.105263158	Inf	34	1	3	0	Co_Occurence	ARHGEF10, TERT	1/3
FAT1	POLD1	0.131578947	Inf	33	1	0	4	Co_Occurence	FAT1, POLD1	1/4
AXIN1	CREBBP	0.153627312	14.08907044	34	1	2	1	Co_Occurence	AXIN1, CREBBP	1/3
UBR5	CREBBP	0.153627312	14.08907044	34	1	2	1	Co_Occurence	CREBBP, UBR5	1/3
BCORL1	EGFR	0.153627312	14.08907044	34	1	2	1	Co_Occurence	BCORL1, EGFR	1/3
UBR5	POLE	0.153627312	14.08907044	34	1	2	1	Co_Occurence	POLE, UBR5	1/3
AXIN1	ARHGEF10	0.201991465	9.669553836	33	1	3	1	Co_Occurence	ARHGEF10, AXIN1	1/4
BCORL1	ARHGEF10	0.201991465	9.669553836	33	1	3	1	Co_Occurence	ARHGEF10, BCOR	1/4
POLE	EGFR	0.224158369	7.391997317	33	1	2	2	Co_Occurence	EGFR, POLE	1/4
FAT1	MTOR	0.248933144	7.26754141	32	1	1	4	Co_Occurence	FAT1, MTOR	1/5

FAT1	UBR5	0.248933144	7.26754141	32	1	1	4	Co_Occurence	FAT1, UBR5	1/5
ARHGEF10	EGFR	0.29065908	4.955830938	32	1	2	3	Co_Occurence	ARHGEF10, EGFR	1/5
POLE	FAT1	0.353247985	3.677238971	31	1	4	2	Co_Occurence	FAT1, POLE	1/6
ARHGEF10	FAT1	0.44564113	2.423382588	30	1	4	3	Co_Occurence	ARHGEF10, FAT1	1/7
ARHGEF10	CREBBP	1	0	31	0	3	4	Mutually_Exclusi ve	ARHGEF10, CREBI	0/7
ARHGEF10	POLE	1	0	31	0	3	4	Mutually_Exclusi ve	ARHGEF10, POLE	0/7
AXIN1	ARID1B	1	0	34	0	2	2	Mutually_Exclusi ve	ARID1B, AXIN1	0/4
BCORL1	ARID1B	1	0	34	0	2	2	Mutually_Exclusi ve	ARID1B, BCORL1	0/4
BIRC6	ARID1B	1	0	34	0	2	2	Mutually_Exclusi ve	ARID1B, BIRC6	0/4
DNMT1	ARID1B	1	0	34	0	2	2	Mutually_Exclusi ve	ARID1B, DNMT1	0/4
MYH9	ARID1B	1	0	34	0	2	2	Mutually_Exclusi ve	ARID1B, MYH9	0/4
NCOR2	ARID1B	1	0	34	0	2	2	Mutually_Exclusi ve	ARID1B, NCOR2	0/4
SMARCA4	ARID1B	1	0	34	0	2	2	Mutually_Exclusi ve	ARID1B, SMARCA4	0/4
TRRAP	ARID1B	1	0	34	0	2	2	Mutually_Exclusi ve	ARID1B, TRRAP	0/4
TSC2	ARID1B	1	0	34	0	2	2	Mutually_Exclusi ve	ARID1B, TSC2	0/4
UBR5	ARID1B	1	0	34	0	2	2	Mutually_Exclusi ve	ARID1B, UBR5	0/4
BCORL1	AXIN1	1	0	34	0	2	2	Mutually_Exclusi ve	AXIN1, BCORL1	0/4
BIRC6	AXIN1	1	0	34	0	2	2	Mutually_Exclusi ve	AXIN1, BIRC6	0/4
DNMT1	AXIN1	1	0	34	0	2	2	Mutually_Exclusi ve	AXIN1, DNMT1	0/4
MTOR	AXIN1	1	0	34	0	2	2	Mutually_Exclusi ve	AXIN1, MTOR	0/4

MYH9	AXIN1	1	0	34	0	2	2	Mutually_Exclusive	AXIN1, MYH9	0/4
NCOR2	AXIN1	1	0	34	0	2	2	Mutually_Exclusive	AXIN1, NCOR2	0/4
SMARCA4	AXIN1	1	0	34	0	2	2	Mutually_Exclusive	AXIN1, SMARCA4	0/4
TRRAP	AXIN1	1	0	34	0	2	2	Mutually_Exclusive	AXIN1, TRRAP	0/4
TSC2	AXIN1	1	0	34	0	2	2	Mutually_Exclusive	AXIN1, TSC2	0/4
UBR5	AXIN1	1	0	34	0	2	2	Mutually_Exclusive	AXIN1, UBR5	0/4
BIRC6	BCORL1	1	0	34	0	2	2	Mutually_Exclusive	BCORL1, BIRC6	0/4
DNMT1	BCORL1	1	0	34	0	2	2	Mutually_Exclusive	BCORL1, DNMT1	0/4
MTOR	BCORL1	1	0	34	0	2	2	Mutually_Exclusive	BCORL1, MTOR	0/4
MYH9	BCORL1	1	0	34	0	2	2	Mutually_Exclusive	BCORL1, MYH9	0/4
NCOR2	BCORL1	1	0	34	0	2	2	Mutually_Exclusive	BCORL1, NCOR2	0/4
SMARCA4	BCORL1	1	0	34	0	2	2	Mutually_Exclusive	BCORL1, SMARCA	0/4
TRRAP	BCORL1	1	0	34	0	2	2	Mutually_Exclusive	BCORL1, TRRAP	0/4
TSC2	BCORL1	1	0	34	0	2	2	Mutually_Exclusive	BCORL1, TSC2	0/4
UBR5	BCORL1	1	0	34	0	2	2	Mutually_Exclusive	BCORL1, UBR5	0/4
DNMT1	BIRC6	1	0	34	0	2	2	Mutually_Exclusive	BIRC6, DNMT1	0/4
MTOR	BIRC6	1	0	34	0	2	2	Mutually_Exclusive	BIRC6, MTOR	0/4
MYH9	BIRC6	1	0	34	0	2	2	Mutually_Exclusive	BIRC6, MYH9	0/4

NCOR2	BIRC6	1	0	34	0	2	2	Mutually_Exclusive	BIRC6, NCOR2	0/4
SMARCA4	BIRC6	1	0	34	0	2	2	Mutually_Exclusive	BIRC6, SMARCA4	0/4
TRRAP	BIRC6	1	0	34	0	2	2	Mutually_Exclusive	BIRC6, TRRAP	0/4
TSC2	BIRC6	1	0	34	0	2	2	Mutually_Exclusive	BIRC6, TSC2	0/4
UBR5	BIRC6	1	0	34	0	2	2	Mutually_Exclusive	BIRC6, UBR5	0/4
MTOR	DNMT1	1	0	34	0	2	2	Mutually_Exclusive	DNMT1, MTOR	0/4
MYH9	DNMT1	1	0	34	0	2	2	Mutually_Exclusive	DNMT1, MYH9	0/4
NCOR2	DNMT1	1	0	34	0	2	2	Mutually_Exclusive	DNMT1, NCOR2	0/4
SMARCA4	DNMT1	1	0	34	0	2	2	Mutually_Exclusive	DNMT1, SMARCA4	0/4
TRRAP	DNMT1	1	0	34	0	2	2	Mutually_Exclusive	DNMT1, TRRAP	0/4
TSC2	DNMT1	1	0	34	0	2	2	Mutually_Exclusive	DNMT1, TSC2	0/4
UBR5	DNMT1	1	0	34	0	2	2	Mutually_Exclusive	DNMT1, UBR5	0/4
MYH9	MTOR	1	0	34	0	2	2	Mutually_Exclusive	MTOR, MYH9	0/4
NCOR2	MTOR	1	0	34	0	2	2	Mutually_Exclusive	MTOR, NCOR2	0/4
SMARCA4	MTOR	1	0	34	0	2	2	Mutually_Exclusive	MTOR, SMARCA4	0/4
TRRAP	MTOR	1	0	34	0	2	2	Mutually_Exclusive	MTOR, TRRAP	0/4
TSC2	MTOR	1	0	34	0	2	2	Mutually_Exclusive	MTOR, TSC2	0/4
UBR5	MTOR	1	0	34	0	2	2	Mutually_Exclusive	MTOR, UBR5	0/4

NCOR2	MYH9	1	0	34	0	2	2	Mutually_Exclusive	MYH9, NCOR2	0/4
SMARCA4	MYH9	1	0	34	0	2	2	Mutually_Exclusive	MYH9, SMARCA4	0/4
TRRAP	MYH9	1	0	34	0	2	2	Mutually_Exclusive	MYH9, TRRAP	0/4
TSC2	MYH9	1	0	34	0	2	2	Mutually_Exclusive	MYH9, TSC2	0/4
UBR5	MYH9	1	0	34	0	2	2	Mutually_Exclusive	MYH9, UBR5	0/4
SMARCA4	NCOR2	1	0	34	0	2	2	Mutually_Exclusive	NCOR2, SMARCA4	0/4
TRRAP	NCOR2	1	0	34	0	2	2	Mutually_Exclusive	NCOR2, TRRAP	0/4
TSC2	NCOR2	1	0	34	0	2	2	Mutually_Exclusive	NCOR2, TSC2	0/4
UBR5	NCOR2	1	0	34	0	2	2	Mutually_Exclusive	NCOR2, UBR5	0/4
TRRAP	SMARCA4	1	0	34	0	2	2	Mutually_Exclusive	SMARCA4, TRRAP	0/4
TSC2	SMARCA4	1	0	34	0	2	2	Mutually_Exclusive	SMARCA4, TSC2	0/4
UBR5	SMARCA4	1	0	34	0	2	2	Mutually_Exclusive	SMARCA4, UBR5	0/4
TSC2	TRRAP	1	0	34	0	2	2	Mutually_Exclusive	TRRAP, TSC2	0/4
UBR5	TRRAP	1	0	34	0	2	2	Mutually_Exclusive	TRRAP, UBR5	0/4
UBR5	TSC2	1	0	34	0	2	2	Mutually_Exclusive	TSC2, UBR5	0/4
CREBBP	FAT1	1	0	30	0	5	3	Mutually_Exclusive	CREBBP, FAT1	0/8
EGFR	FAT1	1	0	30	0	5	3	Mutually_Exclusive	EGFR, FAT1	0/8
ARID1B	FAT1	1	0	31	0	5	2	Mutually_Exclusive	ARID1B, FAT1	0/7

AXIN1	FAT1		1	0	31	0	5	2	Mutually_Exclusive	AXIN1, FAT1	0/7
BCORL1	FAT1		1	0	31	0	5	2	Mutually_Exclusive	BCORL1, FAT1	0/7
BIRC6	FAT1		1	0	31	0	5	2	Mutually_Exclusive	BIRC6, FAT1	0/7
DNMT1	FAT1		1	0	31	0	5	2	Mutually_Exclusive	DNMT1, FAT1	0/7
MYH9	FAT1		1	0	31	0	5	2	Mutually_Exclusive	FAT1, MYH9	0/7
NCOR2	FAT1		1	0	31	0	5	2	Mutually_Exclusive	FAT1, NCOR2	0/7
SMARCA4	FAT1		1	0	31	0	5	2	Mutually_Exclusive	FAT1, SMARCA4	0/7
TRRAP	FAT1		1	0	31	0	5	2	Mutually_Exclusive	FAT1, TRRAP	0/7
TSC2	FAT1		1	0	31	0	5	2	Mutually_Exclusive	FAT1, TSC2	0/7
BRCA2	FAT1		1	0	32	0	5	1	Mutually_Exclusive	BRCA2, FAT1	0/6
COL1A1	FAT1		1	0	32	0	5	1	Mutually_Exclusive	COL1A1, FAT1	0/6
COL3A1	FAT1		1	0	32	0	5	1	Mutually_Exclusive	COL3A1, FAT1	0/6
DCC	FAT1		1	0	32	0	5	1	Mutually_Exclusive	DCC, FAT1	0/6
DROSHA	FAT1		1	0	32	0	5	1	Mutually_Exclusive	DROSHA, FAT1	0/6
FANCA	FAT1		1	0	32	0	5	1	Mutually_Exclusive	FANCA, FAT1	0/6
FLNA	FAT1		1	0	32	0	5	1	Mutually_Exclusive	FAT1, FLNA	0/6
KAT6A	FAT1		1	0	32	0	5	1	Mutually_Exclusive	FAT1, KAT6A	0/6
KAT6B	FAT1		1	0	32	0	5	1	Mutually_Exclusive	FAT1, KAT6B	0/6

N4BP2	FAT1	1	0	32	0	5	1	Mutually_Exclusive	FAT1, N4BP2	0/6
NCOR1	FAT1	1	0	32	0	5	1	Mutually_Exclusive	FAT1, NCOR1	0/6
NF1	FAT1	1	0	32	0	5	1	Mutually_Exclusive	FAT1, NF1	0/6
NIN	FAT1	1	0	32	0	5	1	Mutually_Exclusive	FAT1, NIN	0/6
NSD1	FAT1	1	0	32	0	5	1	Mutually_Exclusive	FAT1, NSD1	0/6
RANBP2	FAT1	1	0	32	0	5	1	Mutually_Exclusive	FAT1, RANBP2	0/6
RET	FAT1	1	0	32	0	5	1	Mutually_Exclusive	FAT1, RET	0/6
RNF213	FAT1	1	0	32	0	5	1	Mutually_Exclusive	FAT1, RNF213	0/6
SETD1B	FAT1	1	0	32	0	5	1	Mutually_Exclusive	FAT1, SETD1B	0/6
SPEN	FAT1	1	0	32	0	5	1	Mutually_Exclusive	FAT1, SPEN	0/6
STAG1	FAT1	1	0	32	0	5	1	Mutually_Exclusive	FAT1, STAG1	0/6
TERT	FAT1	1	0	32	0	5	1	Mutually_Exclusive	FAT1, TERT	0/6
TNC	FAT1	1	0	32	0	5	1	Mutually_Exclusive	FAT1, TNC	0/6
TPR	FAT1	1	0	32	0	5	1	Mutually_Exclusive	FAT1, TPR	0/6
ZMYM3	FAT1	1	0	32	0	5	1	Mutually_Exclusive	FAT1, ZMYM3	0/6
ARID1B	ARHGEF10	1	0	32	0	4	2	Mutually_Exclusive	ARHGEF10, ARID1	0/6
BIRC6	ARHGEF10	1	0	32	0	4	2	Mutually_Exclusive	ARHGEF10, BIRC6	0/6
DNMT1	ARHGEF10	1	0	32	0	4	2	Mutually_Exclusive	ARHGEF10, DNMT	0/6

MTOR	ARHGEF10	1	0	32	0	4	2	Mutually_Exclusive	ARHGEF10, MTOR	0/6
MYH9	ARHGEF10	1	0	32	0	4	2	Mutually_Exclusive	ARHGEF10, MYH9	0/6
NCOR2	ARHGEF10	1	0	32	0	4	2	Mutually_Exclusive	ARHGEF10, NCOR	0/6
SMARCA4	ARHGEF10	1	0	32	0	4	2	Mutually_Exclusive	ARHGEF10, SMAR	0/6
TRRAP	ARHGEF10	1	0	32	0	4	2	Mutually_Exclusive	ARHGEF10, TRRAI	0/6
TSC2	ARHGEF10	1	0	32	0	4	2	Mutually_Exclusive	ARHGEF10, TSC2	0/6
UBR5	ARHGEF10	1	0	32	0	4	2	Mutually_Exclusive	ARHGEF10, UBR5	0/6
BRCA2	ARHGEF10	1	0	33	0	4	1	Mutually_Exclusive	ARHGEF10, BRCA	0/5
COL1A1	ARHGEF10	1	0	33	0	4	1	Mutually_Exclusive	ARHGEF10, COL1A	0/5
COL3A1	ARHGEF10	1	0	33	0	4	1	Mutually_Exclusive	ARHGEF10, COL3A	0/5
DCC	ARHGEF10	1	0	33	0	4	1	Mutually_Exclusive	ARHGEF10, DCC	0/5
DROSHA	ARHGEF10	1	0	33	0	4	1	Mutually_Exclusive	ARHGEF10, DROS	0/5
FANCA	ARHGEF10	1	0	33	0	4	1	Mutually_Exclusive	ARHGEF10, FANCA	0/5
FLNA	ARHGEF10	1	0	33	0	4	1	Mutually_Exclusive	ARHGEF10, FLNA	0/5
KAT6A	ARHGEF10	1	0	33	0	4	1	Mutually_Exclusive	ARHGEF10, KAT6A	0/5
KAT6B	ARHGEF10	1	0	33	0	4	1	Mutually_Exclusive	ARHGEF10, KAT6E	0/5
N4BP2	ARHGEF10	1	0	33	0	4	1	Mutually_Exclusive	ARHGEF10, N4BP2	0/5
NCOR1	ARHGEF10	1	0	33	0	4	1	Mutually_Exclusive	ARHGEF10, NCOR	0/5

NF1	ARHGEF10	1	0	33	0	4	1	Mutually_Exclusive	ARHGEF10, NF1	0/5
NIN	ARHGEF10	1	0	33	0	4	1	Mutually_Exclusive	ARHGEF10, NIN	0/5
NSD1	ARHGEF10	1	0	33	0	4	1	Mutually_Exclusive	ARHGEF10, NSD1	0/5
POLD1	ARHGEF10	1	0	33	0	4	1	Mutually_Exclusive	ARHGEF10, POLD	0/5
RANBP2	ARHGEF10	1	0	33	0	4	1	Mutually_Exclusive	ARHGEF10, RANB	0/5
RET	ARHGEF10	1	0	33	0	4	1	Mutually_Exclusive	ARHGEF10, RET	0/5
RNF213	ARHGEF10	1	0	33	0	4	1	Mutually_Exclusive	ARHGEF10, RNF21	0/5
SETD1B	ARHGEF10	1	0	33	0	4	1	Mutually_Exclusive	ARHGEF10, SETD	0/5
SPEN	ARHGEF10	1	0	33	0	4	1	Mutually_Exclusive	ARHGEF10, SPEN	0/5
STAG1	ARHGEF10	1	0	33	0	4	1	Mutually_Exclusive	ARHGEF10, STAG	0/5
TNC	ARHGEF10	1	0	33	0	4	1	Mutually_Exclusive	ARHGEF10, TNC	0/5
TPR	ARHGEF10	1	0	33	0	4	1	Mutually_Exclusive	ARHGEF10, TPR	0/5
ZMYM3	ARHGEF10	1	0	33	0	4	1	Mutually_Exclusive	ARHGEF10, ZMYM	0/5
EGFR	CREBBP	1	0	32	0	3	3	Mutually_Exclusive	CREBBP, EGFR	0/6
POLE	CREBBP	1	0	32	0	3	3	Mutually_Exclusive	CREBBP, POLE	0/6
ARID1B	CREBBP	1	0	33	0	3	2	Mutually_Exclusive	ARID1B, CREBBP	0/5
BCORL1	CREBBP	1	0	33	0	3	2	Mutually_Exclusive	BCORL1, CREBBP	0/5
BIRC6	CREBBP	1	0	33	0	3	2	Mutually_Exclusive	BIRC6, CREBBP	0/5

DNMT1	CREBBP	1	0	33	0	3	2	Mutually_Exclusive	CREBBP, DNMT1	0/5
MTOR	CREBBP	1	0	33	0	3	2	Mutually_Exclusive	CREBBP, MTOR	0/5
MYH9	CREBBP	1	0	33	0	3	2	Mutually_Exclusive	CREBBP, MYH9	0/5
NCOR2	CREBBP	1	0	33	0	3	2	Mutually_Exclusive	CREBBP, NCOR2	0/5
SMARCA4	CREBBP	1	0	33	0	3	2	Mutually_Exclusive	CREBBP, SMARCA	0/5
TRRAP	CREBBP	1	0	33	0	3	2	Mutually_Exclusive	CREBBP, TRRAP	0/5
TSC2	CREBBP	1	0	33	0	3	2	Mutually_Exclusive	CREBBP, TSC2	0/5
BRCA2	CREBBP	1	0	34	0	3	1	Mutually_Exclusive	BRCA2, CREBBP	0/4
COL1A1	CREBBP	1	0	34	0	3	1	Mutually_Exclusive	COL1A1, CREBBP	0/4
COL3A1	CREBBP	1	0	34	0	3	1	Mutually_Exclusive	COL3A1, CREBBP	0/4
DCC	CREBBP	1	0	34	0	3	1	Mutually_Exclusive	CREBBP, DCC	0/4
FANCA	CREBBP	1	0	34	0	3	1	Mutually_Exclusive	CREBBP, FANCA	0/4
FLNA	CREBBP	1	0	34	0	3	1	Mutually_Exclusive	CREBBP, FLNA	0/4
KAT6A	CREBBP	1	0	34	0	3	1	Mutually_Exclusive	CREBBP, KAT6A	0/4
KAT6B	CREBBP	1	0	34	0	3	1	Mutually_Exclusive	CREBBP, KAT6B	0/4
N4BP2	CREBBP	1	0	34	0	3	1	Mutually_Exclusive	CREBBP, N4BP2	0/4
NCOR1	CREBBP	1	0	34	0	3	1	Mutually_Exclusive	CREBBP, NCOR1	0/4
NF1	CREBBP	1	0	34	0	3	1	Mutually_Exclusive	CREBBP, NF1	0/4

NIN	CREBBP		1	0	34	0	3	1	Mutually_Exclusive	CREBBP, NIN	0/4
POLD1	CREBBP		1	0	34	0	3	1	Mutually_Exclusive	CREBBP, POLD1	0/4
RANBP2	CREBBP		1	0	34	0	3	1	Mutually_Exclusive	CREBBP, RANBP2	0/4
RET	CREBBP		1	0	34	0	3	1	Mutually_Exclusive	CREBBP, RET	0/4
RNF213	CREBBP		1	0	34	0	3	1	Mutually_Exclusive	CREBBP, RNF213	0/4
STAG1	CREBBP		1	0	34	0	3	1	Mutually_Exclusive	CREBBP, STAG1	0/4
TERT	CREBBP		1	0	34	0	3	1	Mutually_Exclusive	CREBBP, TERT	0/4
TNC	CREBBP		1	0	34	0	3	1	Mutually_Exclusive	CREBBP, TNC	0/4
TPR	CREBBP		1	0	34	0	3	1	Mutually_Exclusive	CREBBP, TPR	0/4
ZMYM3	CREBBP		1	0	34	0	3	1	Mutually_Exclusive	CREBBP, ZMYM3	0/4
ARID1B	EGFR		1	0	33	0	3	2	Mutually_Exclusive	ARID1B, EGFR	0/5
AXIN1	EGFR		1	0	33	0	3	2	Mutually_Exclusive	AXIN1, EGFR	0/5
BIRC6	EGFR		1	0	33	0	3	2	Mutually_Exclusive	BIRC6, EGFR	0/5
DNMT1	EGFR		1	0	33	0	3	2	Mutually_Exclusive	DNMT1, EGFR	0/5
MTOR	EGFR		1	0	33	0	3	2	Mutually_Exclusive	EGFR, MTOR	0/5
MYH9	EGFR		1	0	33	0	3	2	Mutually_Exclusive	EGFR, MYH9	0/5
NCOR2	EGFR		1	0	33	0	3	2	Mutually_Exclusive	EGFR, NCOR2	0/5
SMARCA4	EGFR		1	0	33	0	3	2	Mutually_Exclusive	EGFR, SMARCA4	0/5

TRRAP	EGFR	1	0	33	0	3	2	Mutually_Exclusive	EGFR, TRRAP	0/5
TSC2	EGFR	1	0	33	0	3	2	Mutually_Exclusive	EGFR, TSC2	0/5
UBR5	EGFR	1	0	33	0	3	2	Mutually_Exclusive	EGFR, UBR5	0/5
BRCA2	EGFR	1	0	34	0	3	1	Mutually_Exclusive	BRCA2, EGFR	0/4
COL1A1	EGFR	1	0	34	0	3	1	Mutually_Exclusive	COL1A1, EGFR	0/4
COL3A1	EGFR	1	0	34	0	3	1	Mutually_Exclusive	COL3A1, EGFR	0/4
DROSHA	EGFR	1	0	34	0	3	1	Mutually_Exclusive	DROSHA, EGFR	0/4
FANCA	EGFR	1	0	34	0	3	1	Mutually_Exclusive	EGFR, FANCA	0/4
FLNA	EGFR	1	0	34	0	3	1	Mutually_Exclusive	EGFR, FLNA	0/4
KAT6A	EGFR	1	0	34	0	3	1	Mutually_Exclusive	EGFR, KAT6A	0/4
KAT6B	EGFR	1	0	34	0	3	1	Mutually_Exclusive	EGFR, KAT6B	0/4
N4BP2	EGFR	1	0	34	0	3	1	Mutually_Exclusive	EGFR, N4BP2	0/4
NCOR1	EGFR	1	0	34	0	3	1	Mutually_Exclusive	EGFR, NCOR1	0/4
NF1	EGFR	1	0	34	0	3	1	Mutually_Exclusive	EGFR, NF1	0/4
NIN	EGFR	1	0	34	0	3	1	Mutually_Exclusive	EGFR, NIN	0/4
NSD1	EGFR	1	0	34	0	3	1	Mutually_Exclusive	EGFR, NSD1	0/4
POLD1	EGFR	1	0	34	0	3	1	Mutually_Exclusive	EGFR, POLD1	0/4
RANBP2	EGFR	1	0	34	0	3	1	Mutually_Exclusive	EGFR, RANBP2	0/4

RET	EGFR	1	0	34	0	3	1	Mutually_Exclusive	EGFR, RET	0/4
RNF213	EGFR	1	0	34	0	3	1	Mutually_Exclusive	EGFR, RNF213	0/4
SETD1B	EGFR	1	0	34	0	3	1	Mutually_Exclusive	EGFR, SETD1B	0/4
SPEN	EGFR	1	0	34	0	3	1	Mutually_Exclusive	EGFR, SPEN	0/4
STAG1	EGFR	1	0	34	0	3	1	Mutually_Exclusive	EGFR, STAG1	0/4
TERT	EGFR	1	0	34	0	3	1	Mutually_Exclusive	EGFR, TERT	0/4
TNC	EGFR	1	0	34	0	3	1	Mutually_Exclusive	EGFR, TNC	0/4
TPR	EGFR	1	0	34	0	3	1	Mutually_Exclusive	EGFR, TPR	0/4
ZMYM3	EGFR	1	0	34	0	3	1	Mutually_Exclusive	EGFR, ZMYM3	0/4
ARID1B	POLE	1	0	33	0	3	2	Mutually_Exclusive	ARID1B, POLE	0/5
AXIN1	POLE	1	0	33	0	3	2	Mutually_Exclusive	AXIN1, POLE	0/5
BCORL1	POLE	1	0	33	0	3	2	Mutually_Exclusive	BCORL1, POLE	0/5
BIRC6	POLE	1	0	33	0	3	2	Mutually_Exclusive	BIRC6, POLE	0/5
DNMT1	POLE	1	0	33	0	3	2	Mutually_Exclusive	DNMT1, POLE	0/5
MTOR	POLE	1	0	33	0	3	2	Mutually_Exclusive	MTOR, POLE	0/5
MYH9	POLE	1	0	33	0	3	2	Mutually_Exclusive	MYH9, POLE	0/5
NCOR2	POLE	1	0	33	0	3	2	Mutually_Exclusive	NCOR2, POLE	0/5
SMARCA4	POLE	1	0	33	0	3	2	Mutually_Exclusive	POLE, SMARCA4	0/5

TRRAP	POLE	1	0	33	0	3	2	Mutually_Exclusive	POLE, TRRAP	0/5
TSC2	POLE	1	0	33	0	3	2	Mutually_Exclusive	POLE, TSC2	0/5
BRCA2	POLE	1	0	34	0	3	1	Mutually_Exclusive	BRCA2, POLE	0/4
COL1A1	POLE	1	0	34	0	3	1	Mutually_Exclusive	COL1A1, POLE	0/4
COL3A1	POLE	1	0	34	0	3	1	Mutually_Exclusive	COL3A1, POLE	0/4
DCC	POLE	1	0	34	0	3	1	Mutually_Exclusive	DCC, POLE	0/4
DROSHA	POLE	1	0	34	0	3	1	Mutually_Exclusive	DROSHA, POLE	0/4
FANCA	POLE	1	0	34	0	3	1	Mutually_Exclusive	FANCA, POLE	0/4
FLNA	POLE	1	0	34	0	3	1	Mutually_Exclusive	FLNA, POLE	0/4
KAT6A	POLE	1	0	34	0	3	1	Mutually_Exclusive	KAT6A, POLE	0/4
KAT6B	POLE	1	0	34	0	3	1	Mutually_Exclusive	KAT6B, POLE	0/4
N4BP2	POLE	1	0	34	0	3	1	Mutually_Exclusive	N4BP2, POLE	0/4
NCOR1	POLE	1	0	34	0	3	1	Mutually_Exclusive	NCOR1, POLE	0/4
NF1	POLE	1	0	34	0	3	1	Mutually_Exclusive	NF1, POLE	0/4
NIN	POLE	1	0	34	0	3	1	Mutually_Exclusive	NIN, POLE	0/4
NSD1	POLE	1	0	34	0	3	1	Mutually_Exclusive	NSD1, POLE	0/4
POLD1	POLE	1	0	34	0	3	1	Mutually_Exclusive	POLD1, POLE	0/4
RANBP2	POLE	1	0	34	0	3	1	Mutually_Exclusive	POLE, RANBP2	0/4

RET	POLE	1	0	34	0	3	1	Mutually_Exclusive	POLE, RET	0/4
RNF213	POLE	1	0	34	0	3	1	Mutually_Exclusive	POLE, RNF213	0/4
SETD1B	POLE	1	0	34	0	3	1	Mutually_Exclusive	POLE, SETD1B	0/4
SPEN	POLE	1	0	34	0	3	1	Mutually_Exclusive	POLE, SPEN	0/4
STAG1	POLE	1	0	34	0	3	1	Mutually_Exclusive	POLE, STAG1	0/4
TERT	POLE	1	0	34	0	3	1	Mutually_Exclusive	POLE, TERT	0/4
TNC	POLE	1	0	34	0	3	1	Mutually_Exclusive	POLE, TNC	0/4
TPR	POLE	1	0	34	0	3	1	Mutually_Exclusive	POLE, TPR	0/4
ZMYM3	POLE	1	0	34	0	3	1	Mutually_Exclusive	POLE, ZMYM3	0/4
BRCA2	ARID1B	1	0	35	0	2	1	Mutually_Exclusive	ARID1B, BRCA2	0/3
COL1A1	ARID1B	1	0	35	0	2	1	Mutually_Exclusive	ARID1B, COL1A1	0/3
COL3A1	ARID1B	1	0	35	0	2	1	Mutually_Exclusive	ARID1B, COL3A1	0/3
DCC	ARID1B	1	0	35	0	2	1	Mutually_Exclusive	ARID1B, DCC	0/3
DROSHA	ARID1B	1	0	35	0	2	1	Mutually_Exclusive	ARID1B, DROSHA	0/3
FANCA	ARID1B	1	0	35	0	2	1	Mutually_Exclusive	ARID1B, FANCA	0/3
FLNA	ARID1B	1	0	35	0	2	1	Mutually_Exclusive	ARID1B, FLNA	0/3
N4BP2	ARID1B	1	0	35	0	2	1	Mutually_Exclusive	ARID1B, N4BP2	0/3
NCOR1	ARID1B	1	0	35	0	2	1	Mutually_Exclusive	ARID1B, NCOR1	0/3

NF1	ARID1B	1	0	35	0	2	1	Mutually_Exclusive	ARID1B, NF1	0/3
NIN	ARID1B	1	0	35	0	2	1	Mutually_Exclusive	ARID1B, NIN	0/3
NSD1	ARID1B	1	0	35	0	2	1	Mutually_Exclusive	ARID1B, NSD1	0/3
POLD1	ARID1B	1	0	35	0	2	1	Mutually_Exclusive	ARID1B, POLD1	0/3
RET	ARID1B	1	0	35	0	2	1	Mutually_Exclusive	ARID1B, RET	0/3
RNF213	ARID1B	1	0	35	0	2	1	Mutually_Exclusive	ARID1B, RNF213	0/3
SETD1B	ARID1B	1	0	35	0	2	1	Mutually_Exclusive	ARID1B, SETD1B	0/3
SPEN	ARID1B	1	0	35	0	2	1	Mutually_Exclusive	ARID1B, SPEN	0/3
STAG1	ARID1B	1	0	35	0	2	1	Mutually_Exclusive	ARID1B, STAG1	0/3
TERT	ARID1B	1	0	35	0	2	1	Mutually_Exclusive	ARID1B, TERT	0/3
TNC	ARID1B	1	0	35	0	2	1	Mutually_Exclusive	ARID1B, TNC	0/3
TPR	ARID1B	1	0	35	0	2	1	Mutually_Exclusive	ARID1B, TPR	0/3
ZMYM3	ARID1B	1	0	35	0	2	1	Mutually_Exclusive	ARID1B, ZMYM3	0/3
BRCA2	AXIN1	1	0	35	0	2	1	Mutually_Exclusive	AXIN1, BRCA2	0/3
COL1A1	AXIN1	1	0	35	0	2	1	Mutually_Exclusive	AXIN1, COL1A1	0/3
COL3A1	AXIN1	1	0	35	0	2	1	Mutually_Exclusive	AXIN1, COL3A1	0/3
DCC	AXIN1	1	0	35	0	2	1	Mutually_Exclusive	AXIN1, DCC	0/3
DROSHA	AXIN1	1	0	35	0	2	1	Mutually_Exclusive	AXIN1, DROSHA	0/3

FANCA	AXIN1	1	0	35	0	2	1	Mutually_Exclusive	AXIN1, FANCA	0/3
FLNA	AXIN1	1	0	35	0	2	1	Mutually_Exclusive	AXIN1, FLNA	0/3
KAT6A	AXIN1	1	0	35	0	2	1	Mutually_Exclusive	AXIN1, KAT6A	0/3
KAT6B	AXIN1	1	0	35	0	2	1	Mutually_Exclusive	AXIN1, KAT6B	0/3
N4BP2	AXIN1	1	0	35	0	2	1	Mutually_Exclusive	AXIN1, N4BP2	0/3
NCOR1	AXIN1	1	0	35	0	2	1	Mutually_Exclusive	AXIN1, NCOR1	0/3
NF1	AXIN1	1	0	35	0	2	1	Mutually_Exclusive	AXIN1, NF1	0/3
NIN	AXIN1	1	0	35	0	2	1	Mutually_Exclusive	AXIN1, NIN	0/3
POLD1	AXIN1	1	0	35	0	2	1	Mutually_Exclusive	AXIN1, POLD1	0/3
RANBP2	AXIN1	1	0	35	0	2	1	Mutually_Exclusive	AXIN1, RANBP2	0/3
RET	AXIN1	1	0	35	0	2	1	Mutually_Exclusive	AXIN1, RET	0/3
RNF213	AXIN1	1	0	35	0	2	1	Mutually_Exclusive	AXIN1, RNF213	0/3
SPEN	AXIN1	1	0	35	0	2	1	Mutually_Exclusive	AXIN1, SPEN	0/3
STAG1	AXIN1	1	0	35	0	2	1	Mutually_Exclusive	AXIN1, STAG1	0/3
TNC	AXIN1	1	0	35	0	2	1	Mutually_Exclusive	AXIN1, TNC	0/3
TPR	AXIN1	1	0	35	0	2	1	Mutually_Exclusive	AXIN1, TPR	0/3
ZMYM3	AXIN1	1	0	35	0	2	1	Mutually_Exclusive	AXIN1, ZMYM3	0/3
BRCA2	BCORL1	1	0	35	0	2	1	Mutually_Exclusive	BCORL1, BRCA2	0/3

COL1A1	BCORL1	1	0	35	0	2	1	Mutually_Exclusive	BCORL1, COL1A1	0/3
COL3A1	BCORL1	1	0	35	0	2	1	Mutually_Exclusive	BCORL1, COL3A1	0/3
DCC	BCORL1	1	0	35	0	2	1	Mutually_Exclusive	BCORL1, DCC	0/3
DROSHA	BCORL1	1	0	35	0	2	1	Mutually_Exclusive	BCORL1, DROSHA	0/3
FANCA	BCORL1	1	0	35	0	2	1	Mutually_Exclusive	BCORL1, FANCA	0/3
FLNA	BCORL1	1	0	35	0	2	1	Mutually_Exclusive	BCORL1, FLNA	0/3
KAT6A	BCORL1	1	0	35	0	2	1	Mutually_Exclusive	BCORL1, KAT6A	0/3
KAT6B	BCORL1	1	0	35	0	2	1	Mutually_Exclusive	BCORL1, KAT6B	0/3
N4BP2	BCORL1	1	0	35	0	2	1	Mutually_Exclusive	BCORL1, N4BP2	0/3
NCOR1	BCORL1	1	0	35	0	2	1	Mutually_Exclusive	BCORL1, NCOR1	0/3
NF1	BCORL1	1	0	35	0	2	1	Mutually_Exclusive	BCORL1, NF1	0/3
NIN	BCORL1	1	0	35	0	2	1	Mutually_Exclusive	BCORL1, NIN	0/3
NSD1	BCORL1	1	0	35	0	2	1	Mutually_Exclusive	BCORL1, NSD1	0/3
POLD1	BCORL1	1	0	35	0	2	1	Mutually_Exclusive	BCORL1, POLD1	0/3
RANBP2	BCORL1	1	0	35	0	2	1	Mutually_Exclusive	BCORL1, RANBP2	0/3
RET	BCORL1	1	0	35	0	2	1	Mutually_Exclusive	BCORL1, RET	0/3
RNF213	BCORL1	1	0	35	0	2	1	Mutually_Exclusive	BCORL1, RNF213	0/3
SETD1B	BCORL1	1	0	35	0	2	1	Mutually_Exclusive	BCORL1, SETD1B	0/3

SPEN	BCORL1	1	0	35	0	2	1	Mutually_Exclusive	BCORL1, SPEN	0/3
STAG1	BCORL1	1	0	35	0	2	1	Mutually_Exclusive	BCORL1, STAG1	0/3
TERT	BCORL1	1	0	35	0	2	1	Mutually_Exclusive	BCORL1, TERT	0/3
TNC	BCORL1	1	0	35	0	2	1	Mutually_Exclusive	BCORL1, TNC	0/3
TPR	BCORL1	1	0	35	0	2	1	Mutually_Exclusive	BCORL1, TPR	0/3
ZMYM3	BCORL1	1	0	35	0	2	1	Mutually_Exclusive	BCORL1, ZMYM3	0/3
BRCA2	BIRC6	1	0	35	0	2	1	Mutually_Exclusive	BIRC6, BRCA2	0/3
COL1A1	BIRC6	1	0	35	0	2	1	Mutually_Exclusive	BIRC6, COL1A1	0/3
COL3A1	BIRC6	1	0	35	0	2	1	Mutually_Exclusive	BIRC6, COL3A1	0/3
DCC	BIRC6	1	0	35	0	2	1	Mutually_Exclusive	BIRC6, DCC	0/3
DROSHA	BIRC6	1	0	35	0	2	1	Mutually_Exclusive	BIRC6, DROSHA	0/3
FANCA	BIRC6	1	0	35	0	2	1	Mutually_Exclusive	BIRC6, FANCA	0/3
FLNA	BIRC6	1	0	35	0	2	1	Mutually_Exclusive	BIRC6, FLNA	0/3
KAT6A	BIRC6	1	0	35	0	2	1	Mutually_Exclusive	BIRC6, KAT6A	0/3
KAT6B	BIRC6	1	0	35	0	2	1	Mutually_Exclusive	BIRC6, KAT6B	0/3
N4BP2	BIRC6	1	0	35	0	2	1	Mutually_Exclusive	BIRC6, N4BP2	0/3
NCOR1	BIRC6	1	0	35	0	2	1	Mutually_Exclusive	BIRC6, NCOR1	0/3
NF1	BIRC6	1	0	35	0	2	1	Mutually_Exclusive	BIRC6, NF1	0/3

NIN	BIRC6	1	0	35	0	2	1	Mutually_Exclusive	BIRC6, NIN	0/3
NSD1	BIRC6	1	0	35	0	2	1	Mutually_Exclusive	BIRC6, NSD1	0/3
POLD1	BIRC6	1	0	35	0	2	1	Mutually_Exclusive	BIRC6, POLD1	0/3
RANBP2	BIRC6	1	0	35	0	2	1	Mutually_Exclusive	BIRC6, RANBP2	0/3
RET	BIRC6	1	0	35	0	2	1	Mutually_Exclusive	BIRC6, RET	0/3
RNF213	BIRC6	1	0	35	0	2	1	Mutually_Exclusive	BIRC6, RNF213	0/3
SETD1B	BIRC6	1	0	35	0	2	1	Mutually_Exclusive	BIRC6, SETD1B	0/3
SPEN	BIRC6	1	0	35	0	2	1	Mutually_Exclusive	BIRC6, SPEN	0/3
STAG1	BIRC6	1	0	35	0	2	1	Mutually_Exclusive	BIRC6, STAG1	0/3
TERT	BIRC6	1	0	35	0	2	1	Mutually_Exclusive	BIRC6, TERT	0/3
TNC	BIRC6	1	0	35	0	2	1	Mutually_Exclusive	BIRC6, TNC	0/3
TPR	BIRC6	1	0	35	0	2	1	Mutually_Exclusive	BIRC6, TPR	0/3
ZMYM3	BIRC6	1	0	35	0	2	1	Mutually_Exclusive	BIRC6, ZMYM3	0/3
BRCA2	DNMT1	1	0	35	0	2	1	Mutually_Exclusive	BRCA2, DNMT1	0/3
COL1A1	DNMT1	1	0	35	0	2	1	Mutually_Exclusive	COL1A1, DNMT1	0/3
COL3A1	DNMT1	1	0	35	0	2	1	Mutually_Exclusive	COL3A1, DNMT1	0/3
DCC	DNMT1	1	0	35	0	2	1	Mutually_Exclusive	DCC, DNMT1	0/3
DROSHA	DNMT1	1	0	35	0	2	1	Mutually_Exclusive	DNMT1, DROSHA	0/3

FANCA	DNMT1	1	0	35	0	2	1	Mutually_Exclusive	DNMT1, FANCA	0/3
FLNA	DNMT1	1	0	35	0	2	1	Mutually_Exclusive	DNMT1, FLNA	0/3
KAT6A	DNMT1	1	0	35	0	2	1	Mutually_Exclusive	DNMT1, KAT6A	0/3
KAT6B	DNMT1	1	0	35	0	2	1	Mutually_Exclusive	DNMT1, KAT6B	0/3
NCOR1	DNMT1	1	0	35	0	2	1	Mutually_Exclusive	DNMT1, NCOR1	0/3
NF1	DNMT1	1	0	35	0	2	1	Mutually_Exclusive	DNMT1, NF1	0/3
NIN	DNMT1	1	0	35	0	2	1	Mutually_Exclusive	DNMT1, NIN	0/3
NSD1	DNMT1	1	0	35	0	2	1	Mutually_Exclusive	DNMT1, NSD1	0/3
POLD1	DNMT1	1	0	35	0	2	1	Mutually_Exclusive	DNMT1, POLD1	0/3
RANBP2	DNMT1	1	0	35	0	2	1	Mutually_Exclusive	DNMT1, RANBP2	0/3
RET	DNMT1	1	0	35	0	2	1	Mutually_Exclusive	DNMT1, RET	0/3
RNF213	DNMT1	1	0	35	0	2	1	Mutually_Exclusive	DNMT1, RNF213	0/3
SETD1B	DNMT1	1	0	35	0	2	1	Mutually_Exclusive	DNMT1, SETD1B	0/3
SPEN	DNMT1	1	0	35	0	2	1	Mutually_Exclusive	DNMT1, SPEN	0/3
STAG1	DNMT1	1	0	35	0	2	1	Mutually_Exclusive	DNMT1, STAG1	0/3
TERT	DNMT1	1	0	35	0	2	1	Mutually_Exclusive	DNMT1, TERT	0/3
TNC	DNMT1	1	0	35	0	2	1	Mutually_Exclusive	DNMT1, TNC	0/3
TPR	DNMT1	1	0	35	0	2	1	Mutually_Exclusive	DNMT1, TPR	0/3

ZMYM3	DNMT1	1	0	35	0	2	1	Mutually_Exclusive	DNMT1, ZMYM3	0/3
BRCA2	MTOR	1	0	35	0	2	1	Mutually_Exclusive	BRCA2, MTOR	0/3
COL1A1	MTOR	1	0	35	0	2	1	Mutually_Exclusive	COL1A1, MTOR	0/3
COL3A1	MTOR	1	0	35	0	2	1	Mutually_Exclusive	COL3A1, MTOR	0/3
DCC	MTOR	1	0	35	0	2	1	Mutually_Exclusive	DCC, MTOR	0/3
DROSHA	MTOR	1	0	35	0	2	1	Mutually_Exclusive	DROSHA, MTOR	0/3
FANCA	MTOR	1	0	35	0	2	1	Mutually_Exclusive	FANCA, MTOR	0/3
FLNA	MTOR	1	0	35	0	2	1	Mutually_Exclusive	FLNA, MTOR	0/3
KAT6B	MTOR	1	0	35	0	2	1	Mutually_Exclusive	KAT6B, MTOR	0/3
N4BP2	MTOR	1	0	35	0	2	1	Mutually_Exclusive	MTOR, N4BP2	0/3
NCOR1	MTOR	1	0	35	0	2	1	Mutually_Exclusive	MTOR, NCOR1	0/3
NF1	MTOR	1	0	35	0	2	1	Mutually_Exclusive	MTOR, NF1	0/3
NIN	MTOR	1	0	35	0	2	1	Mutually_Exclusive	MTOR, NIN	0/3
NSD1	MTOR	1	0	35	0	2	1	Mutually_Exclusive	MTOR, NSD1	0/3
POLD1	MTOR	1	0	35	0	2	1	Mutually_Exclusive	MTOR, POLD1	0/3
RANBP2	MTOR	1	0	35	0	2	1	Mutually_Exclusive	MTOR, RANBP2	0/3
RET	MTOR	1	0	35	0	2	1	Mutually_Exclusive	MTOR, RET	0/3
RNF213	MTOR	1	0	35	0	2	1	Mutually_Exclusive	MTOR, RNF213	0/3

SETD1B	MTOR	1	0	35	0	2	1	Mutually_Exclusive	MTOR, SETD1B	0/3
SPEN	MTOR	1	0	35	0	2	1	Mutually_Exclusive	MTOR, SPEN	0/3
STAG1	MTOR	1	0	35	0	2	1	Mutually_Exclusive	MTOR, STAG1	0/3
TERT	MTOR	1	0	35	0	2	1	Mutually_Exclusive	MTOR, TERT	0/3
TNC	MTOR	1	0	35	0	2	1	Mutually_Exclusive	MTOR, TNC	0/3
TPR	MTOR	1	0	35	0	2	1	Mutually_Exclusive	MTOR, TPR	0/3
ZMYM3	MTOR	1	0	35	0	2	1	Mutually_Exclusive	MTOR, ZMYM3	0/3
BRCA2	MYH9	1	0	35	0	2	1	Mutually_Exclusive	BRCA2, MYH9	0/3
COL1A1	MYH9	1	0	35	0	2	1	Mutually_Exclusive	COL1A1, MYH9	0/3
COL3A1	MYH9	1	0	35	0	2	1	Mutually_Exclusive	COL3A1, MYH9	0/3
DCC	MYH9	1	0	35	0	2	1	Mutually_Exclusive	DCC, MYH9	0/3
DROSHA	MYH9	1	0	35	0	2	1	Mutually_Exclusive	DROSHA, MYH9	0/3
FANCA	MYH9	1	0	35	0	2	1	Mutually_Exclusive	FANCA, MYH9	0/3
FLNA	MYH9	1	0	35	0	2	1	Mutually_Exclusive	FLNA, MYH9	0/3
KAT6A	MYH9	1	0	35	0	2	1	Mutually_Exclusive	KAT6A, MYH9	0/3
KAT6B	MYH9	1	0	35	0	2	1	Mutually_Exclusive	KAT6B, MYH9	0/3
N4BP2	MYH9	1	0	35	0	2	1	Mutually_Exclusive	MYH9, N4BP2	0/3
NCOR1	MYH9	1	0	35	0	2	1	Mutually_Exclusive	MYH9, NCOR1	0/3

NF1	MYH9	1	0	35	0	2	1	Mutually_Exclusive	MYH9, NF1	0/3
NIN	MYH9	1	0	35	0	2	1	Mutually_Exclusive	MYH9, NIN	0/3
NSD1	MYH9	1	0	35	0	2	1	Mutually_Exclusive	MYH9, NSD1	0/3
POLD1	MYH9	1	0	35	0	2	1	Mutually_Exclusive	MYH9, POLD1	0/3
RANBP2	MYH9	1	0	35	0	2	1	Mutually_Exclusive	MYH9, RANBP2	0/3
RET	MYH9	1	0	35	0	2	1	Mutually_Exclusive	MYH9, RET	0/3
RNF213	MYH9	1	0	35	0	2	1	Mutually_Exclusive	MYH9, RNF213	0/3
SETD1B	MYH9	1	0	35	0	2	1	Mutually_Exclusive	MYH9, SETD1B	0/3
SPEN	MYH9	1	0	35	0	2	1	Mutually_Exclusive	MYH9, SPEN	0/3
STAG1	MYH9	1	0	35	0	2	1	Mutually_Exclusive	MYH9, STAG1	0/3
TERT	MYH9	1	0	35	0	2	1	Mutually_Exclusive	MYH9, TERT	0/3
TNC	MYH9	1	0	35	0	2	1	Mutually_Exclusive	MYH9, TNC	0/3
TPR	MYH9	1	0	35	0	2	1	Mutually_Exclusive	MYH9, TPR	0/3
ZMYM3	MYH9	1	0	35	0	2	1	Mutually_Exclusive	MYH9, ZMYM3	0/3
BRCA2	NCOR2	1	0	35	0	2	1	Mutually_Exclusive	BRCA2, NCOR2	0/3
COL1A1	NCOR2	1	0	35	0	2	1	Mutually_Exclusive	COL1A1, NCOR2	0/3
DCC	NCOR2	1	0	35	0	2	1	Mutually_Exclusive	DCC, NCOR2	0/3
DROSHA	NCOR2	1	0	35	0	2	1	Mutually_Exclusive	DROSHA, NCOR2	0/3

FANCA	NCOR2	1	0	35	0	2	1	Mutually_Exclusive	FANCA, NCOR2	0/3
FLNA	NCOR2	1	0	35	0	2	1	Mutually_Exclusive	FLNA, NCOR2	0/3
KAT6A	NCOR2	1	0	35	0	2	1	Mutually_Exclusive	KAT6A, NCOR2	0/3
KAT6B	NCOR2	1	0	35	0	2	1	Mutually_Exclusive	KAT6B, NCOR2	0/3
N4BP2	NCOR2	1	0	35	0	2	1	Mutually_Exclusive	N4BP2, NCOR2	0/3
NCOR1	NCOR2	1	0	35	0	2	1	Mutually_Exclusive	NCOR1, NCOR2	0/3
NF1	NCOR2	1	0	35	0	2	1	Mutually_Exclusive	NCOR2, NF1	0/3
NIN	NCOR2	1	0	35	0	2	1	Mutually_Exclusive	NCOR2, NIN	0/3
NSD1	NCOR2	1	0	35	0	2	1	Mutually_Exclusive	NCOR2, NSD1	0/3
POLD1	NCOR2	1	0	35	0	2	1	Mutually_Exclusive	NCOR2, POLD1	0/3
RANBP2	NCOR2	1	0	35	0	2	1	Mutually_Exclusive	NCOR2, RANBP2	0/3
RET	NCOR2	1	0	35	0	2	1	Mutually_Exclusive	NCOR2, RET	0/3
SETD1B	NCOR2	1	0	35	0	2	1	Mutually_Exclusive	NCOR2, SETD1B	0/3
SPEN	NCOR2	1	0	35	0	2	1	Mutually_Exclusive	NCOR2, SPEN	0/3
STAG1	NCOR2	1	0	35	0	2	1	Mutually_Exclusive	NCOR2, STAG1	0/3
TERT	NCOR2	1	0	35	0	2	1	Mutually_Exclusive	NCOR2, TERT	0/3
TNC	NCOR2	1	0	35	0	2	1	Mutually_Exclusive	NCOR2, TNC	0/3
TPR	NCOR2	1	0	35	0	2	1	Mutually_Exclusive	NCOR2, TPR	0/3

ZMYM3	NCOR2	1	0	35	0	2	1	Mutually_Exclusive	NCOR2, ZMYM3	0/3
BRCA2	SMARCA4	1	0	35	0	2	1	Mutually_Exclusive	BRCA2, SMARCA4	0/3
COL1A1	SMARCA4	1	0	35	0	2	1	Mutually_Exclusive	COL1A1, SMARCA	0/3
COL3A1	SMARCA4	1	0	35	0	2	1	Mutually_Exclusive	COL3A1, SMARCA	0/3
DCC	SMARCA4	1	0	35	0	2	1	Mutually_Exclusive	DCC, SMARCA4	0/3
DROSHA	SMARCA4	1	0	35	0	2	1	Mutually_Exclusive	DROSHA, SMARC	0/3
FANCA	SMARCA4	1	0	35	0	2	1	Mutually_Exclusive	FANCA, SMARCA4	0/3
FLNA	SMARCA4	1	0	35	0	2	1	Mutually_Exclusive	FLNA, SMARCA4	0/3
KAT6A	SMARCA4	1	0	35	0	2	1	Mutually_Exclusive	KAT6A, SMARCA4	0/3
KAT6B	SMARCA4	1	0	35	0	2	1	Mutually_Exclusive	KAT6B, SMARCA4	0/3
N4BP2	SMARCA4	1	0	35	0	2	1	Mutually_Exclusive	N4BP2, SMARCA4	0/3
NCOR1	SMARCA4	1	0	35	0	2	1	Mutually_Exclusive	NCOR1, SMARCA4	0/3
NF1	SMARCA4	1	0	35	0	2	1	Mutually_Exclusive	NF1, SMARCA4	0/3
NIN	SMARCA4	1	0	35	0	2	1	Mutually_Exclusive	NIN, SMARCA4	0/3
NSD1	SMARCA4	1	0	35	0	2	1	Mutually_Exclusive	NSD1, SMARCA4	0/3
POLD1	SMARCA4	1	0	35	0	2	1	Mutually_Exclusive	POLD1, SMARCA4	0/3
RANBP2	SMARCA4	1	0	35	0	2	1	Mutually_Exclusive	RANBP2, SMARCA	0/3
RET	SMARCA4	1	0	35	0	2	1	Mutually_Exclusive	RET, SMARCA4	0/3

RNF213	SMARCA4		1	0	35	0	2	1	Mutually_Exclusive	RNF213, SMARCA4	0/3
SETD1B	SMARCA4		1	0	35	0	2	1	Mutually_Exclusive	SETD1B, SMARCA	0/3
SPEN	SMARCA4		1	0	35	0	2	1	Mutually_Exclusive	SMARCA4, SPEN	0/3
STAG1	SMARCA4		1	0	35	0	2	1	Mutually_Exclusive	SMARCA4, STAG1	0/3
TERT	SMARCA4		1	0	35	0	2	1	Mutually_Exclusive	SMARCA4, TERT	0/3
TNC	SMARCA4		1	0	35	0	2	1	Mutually_Exclusive	SMARCA4, TNC	0/3
TPR	SMARCA4		1	0	35	0	2	1	Mutually_Exclusive	SMARCA4, TPR	0/3
ZMYM3	SMARCA4		1	0	35	0	2	1	Mutually_Exclusive	SMARCA4, ZMYM3	0/3
BRCA2	TRRAP		1	0	35	0	2	1	Mutually_Exclusive	BRCA2, TRRAP	0/3
COL1A1	TRRAP		1	0	35	0	2	1	Mutually_Exclusive	COL1A1, TRRAP	0/3
COL3A1	TRRAP		1	0	35	0	2	1	Mutually_Exclusive	COL3A1, TRRAP	0/3
DCC	TRRAP		1	0	35	0	2	1	Mutually_Exclusive	DCC, TRRAP	0/3
DROSHA	TRRAP		1	0	35	0	2	1	Mutually_Exclusive	DROSHA, TRRAP	0/3
FANCA	TRRAP		1	0	35	0	2	1	Mutually_Exclusive	FANCA, TRRAP	0/3
FLNA	TRRAP		1	0	35	0	2	1	Mutually_Exclusive	FLNA, TRRAP	0/3
KAT6A	TRRAP		1	0	35	0	2	1	Mutually_Exclusive	KAT6A, TRRAP	0/3
KAT6B	TRRAP		1	0	35	0	2	1	Mutually_Exclusive	KAT6B, TRRAP	0/3
N4BP2	TRRAP		1	0	35	0	2	1	Mutually_Exclusive	N4BP2, TRRAP	0/3

NCOR1	TRRAP	1	0	35	0	2	1	Mutually_Exclusive	NCOR1, TRRAP	0/3
NF1	TRRAP	1	0	35	0	2	1	Mutually_Exclusive	NF1, TRRAP	0/3
NIN	TRRAP	1	0	35	0	2	1	Mutually_Exclusive	NIN, TRRAP	0/3
NSD1	TRRAP	1	0	35	0	2	1	Mutually_Exclusive	NSD1, TRRAP	0/3
POLD1	TRRAP	1	0	35	0	2	1	Mutually_Exclusive	POLD1, TRRAP	0/3
RANBP2	TRRAP	1	0	35	0	2	1	Mutually_Exclusive	RANBP2, TRRAP	0/3
RNF213	TRRAP	1	0	35	0	2	1	Mutually_Exclusive	RNF213, TRRAP	0/3
SETD1B	TRRAP	1	0	35	0	2	1	Mutually_Exclusive	SETD1B, TRRAP	0/3
SPEN	TRRAP	1	0	35	0	2	1	Mutually_Exclusive	SPEN, TRRAP	0/3
STAG1	TRRAP	1	0	35	0	2	1	Mutually_Exclusive	STAG1, TRRAP	0/3
TERT	TRRAP	1	0	35	0	2	1	Mutually_Exclusive	TERT, TRRAP	0/3
TNC	TRRAP	1	0	35	0	2	1	Mutually_Exclusive	TNC, TRRAP	0/3
TPR	TRRAP	1	0	35	0	2	1	Mutually_Exclusive	TPR, TRRAP	0/3
ZMYM3	TRRAP	1	0	35	0	2	1	Mutually_Exclusive	TRRAP, ZMYM3	0/3
BRCA2	TSC2	1	0	35	0	2	1	Mutually_Exclusive	BRCA2, TSC2	0/3
COL3A1	TSC2	1	0	35	0	2	1	Mutually_Exclusive	COL3A1, TSC2	0/3
DCC	TSC2	1	0	35	0	2	1	Mutually_Exclusive	DCC, TSC2	0/3
DROSHA	TSC2	1	0	35	0	2	1	Mutually_Exclusive	DROSHA, TSC2	0/3

FANCA	TSC2	1	0	35	0	2	1	Mutually_Exclusive	FANCA, TSC2	0/3
FLNA	TSC2	1	0	35	0	2	1	Mutually_Exclusive	FLNA, TSC2	0/3
KAT6A	TSC2	1	0	35	0	2	1	Mutually_Exclusive	KAT6A, TSC2	0/3
KAT6B	TSC2	1	0	35	0	2	1	Mutually_Exclusive	KAT6B, TSC2	0/3
N4BP2	TSC2	1	0	35	0	2	1	Mutually_Exclusive	N4BP2, TSC2	0/3
NCOR1	TSC2	1	0	35	0	2	1	Mutually_Exclusive	NCOR1, TSC2	0/3
NF1	TSC2	1	0	35	0	2	1	Mutually_Exclusive	NF1, TSC2	0/3
NIN	TSC2	1	0	35	0	2	1	Mutually_Exclusive	NIN, TSC2	0/3
NSD1	TSC2	1	0	35	0	2	1	Mutually_Exclusive	NSD1, TSC2	0/3
POLD1	TSC2	1	0	35	0	2	1	Mutually_Exclusive	POLD1, TSC2	0/3
RANBP2	TSC2	1	0	35	0	2	1	Mutually_Exclusive	RANBP2, TSC2	0/3
RET	TSC2	1	0	35	0	2	1	Mutually_Exclusive	RET, TSC2	0/3
RNF213	TSC2	1	0	35	0	2	1	Mutually_Exclusive	RNF213, TSC2	0/3
SETD1B	TSC2	1	0	35	0	2	1	Mutually_Exclusive	SETD1B, TSC2	0/3
SPEN	TSC2	1	0	35	0	2	1	Mutually_Exclusive	SPEN, TSC2	0/3
STAG1	TSC2	1	0	35	0	2	1	Mutually_Exclusive	STAG1, TSC2	0/3
TERT	TSC2	1	0	35	0	2	1	Mutually_Exclusive	TERT, TSC2	0/3
TNC	TSC2	1	0	35	0	2	1	Mutually_Exclusive	TNC, TSC2	0/3

TPR	TSC2		1	0	35	0	2	1	Mutually_Exclusive	TPR, TSC2	0/3
ZMYM3	TSC2		1	0	35	0	2	1	Mutually_Exclusive	TSC2, ZMYM3	0/3
BRCA2	UBR5		1	0	35	0	2	1	Mutually_Exclusive	BRCA2, UBR5	0/3
COL1A1	UBR5		1	0	35	0	2	1	Mutually_Exclusive	COL1A1, UBR5	0/3
COL3A1	UBR5		1	0	35	0	2	1	Mutually_Exclusive	COL3A1, UBR5	0/3
DCC	UBR5		1	0	35	0	2	1	Mutually_Exclusive	DCC, UBR5	0/3
DROSHA	UBR5		1	0	35	0	2	1	Mutually_Exclusive	DROSHA, UBR5	0/3
FANCA	UBR5		1	0	35	0	2	1	Mutually_Exclusive	FANCA, UBR5	0/3
FLNA	UBR5		1	0	35	0	2	1	Mutually_Exclusive	FLNA, UBR5	0/3
KAT6A	UBR5		1	0	35	0	2	1	Mutually_Exclusive	KAT6A, UBR5	0/3
KAT6B	UBR5		1	0	35	0	2	1	Mutually_Exclusive	KAT6B, UBR5	0/3
N4BP2	UBR5		1	0	35	0	2	1	Mutually_Exclusive	N4BP2, UBR5	0/3
NCOR1	UBR5		1	0	35	0	2	1	Mutually_Exclusive	NCOR1, UBR5	0/3
NF1	UBR5		1	0	35	0	2	1	Mutually_Exclusive	NF1, UBR5	0/3
NIN	UBR5		1	0	35	0	2	1	Mutually_Exclusive	NIN, UBR5	0/3
NSD1	UBR5		1	0	35	0	2	1	Mutually_Exclusive	NSD1, UBR5	0/3
POLD1	UBR5		1	0	35	0	2	1	Mutually_Exclusive	POLD1, UBR5	0/3
RANBP2	UBR5		1	0	35	0	2	1	Mutually_Exclusive	RANBP2, UBR5	0/3

RET	UBR5	1	0	35	0	2	1	Mutually_Exclusive	RET, UBR5	0/3
RNF213	UBR5	1	0	35	0	2	1	Mutually_Exclusive	RNF213, UBR5	0/3
SETD1B	UBR5	1	0	35	0	2	1	Mutually_Exclusive	SETD1B, UBR5	0/3
STAG1	UBR5	1	0	35	0	2	1	Mutually_Exclusive	STAG1, UBR5	0/3
TERT	UBR5	1	0	35	0	2	1	Mutually_Exclusive	TERT, UBR5	0/3
TNC	UBR5	1	0	35	0	2	1	Mutually_Exclusive	TNC, UBR5	0/3
TPR	UBR5	1	0	35	0	2	1	Mutually_Exclusive	TPR, UBR5	0/3
ZMYM3	UBR5	1	0	35	0	2	1	Mutually_Exclusive	UBR5, ZMYM3	0/3
COL1A1	BRCA2	1	0	36	0	1	1	Mutually_Exclusive	BRCA2, COL1A1	0/2
COL3A1	BRCA2	1	0	36	0	1	1	Mutually_Exclusive	BRCA2, COL3A1	0/2
DCC	BRCA2	1	0	36	0	1	1	Mutually_Exclusive	BRCA2, DCC	0/2
DROSHA	BRCA2	1	0	36	0	1	1	Mutually_Exclusive	BRCA2, DROSHA	0/2
FLNA	BRCA2	1	0	36	0	1	1	Mutually_Exclusive	BRCA2, FLNA	0/2
KAT6A	BRCA2	1	0	36	0	1	1	Mutually_Exclusive	BRCA2, KAT6A	0/2
KAT6B	BRCA2	1	0	36	0	1	1	Mutually_Exclusive	BRCA2, KAT6B	0/2
N4BP2	BRCA2	1	0	36	0	1	1	Mutually_Exclusive	BRCA2, N4BP2	0/2
NCOR1	BRCA2	1	0	36	0	1	1	Mutually_Exclusive	BRCA2, NCOR1	0/2
NF1	BRCA2	1	0	36	0	1	1	Mutually_Exclusive	BRCA2, NF1	0/2

NIN	BRCA2	1	0	36	0	1	1	Mutually_Exclusive	BRCA2, NIN	0/2
NSD1	BRCA2	1	0	36	0	1	1	Mutually_Exclusive	BRCA2, NSD1	0/2
POLD1	BRCA2	1	0	36	0	1	1	Mutually_Exclusive	BRCA2, POLD1	0/2
RANBP2	BRCA2	1	0	36	0	1	1	Mutually_Exclusive	BRCA2, RANBP2	0/2
RET	BRCA2	1	0	36	0	1	1	Mutually_Exclusive	BRCA2, RET	0/2
RNF213	BRCA2	1	0	36	0	1	1	Mutually_Exclusive	BRCA2, RNF213	0/2
SETD1B	BRCA2	1	0	36	0	1	1	Mutually_Exclusive	BRCA2, SETD1B	0/2
SPEN	BRCA2	1	0	36	0	1	1	Mutually_Exclusive	BRCA2, SPEN	0/2
STAG1	BRCA2	1	0	36	0	1	1	Mutually_Exclusive	BRCA2, STAG1	0/2
TERT	BRCA2	1	0	36	0	1	1	Mutually_Exclusive	BRCA2, TERT	0/2
TNC	BRCA2	1	0	36	0	1	1	Mutually_Exclusive	BRCA2, TNC	0/2
TPR	BRCA2	1	0	36	0	1	1	Mutually_Exclusive	BRCA2, TPR	0/2
COL3A1	COL1A1	1	0	36	0	1	1	Mutually_Exclusive	COL1A1, COL3A1	0/2
DCC	COL1A1	1	0	36	0	1	1	Mutually_Exclusive	COL1A1, DCC	0/2
DROSHA	COL1A1	1	0	36	0	1	1	Mutually_Exclusive	COL1A1, DROSHA	0/2
FANCA	COL1A1	1	0	36	0	1	1	Mutually_Exclusive	COL1A1, FANCA	0/2
FLNA	COL1A1	1	0	36	0	1	1	Mutually_Exclusive	COL1A1, FLNA	0/2
KAT6A	COL1A1	1	0	36	0	1	1	Mutually_Exclusive	COL1A1, KAT6A	0/2

KAT6B	COL1A1	1	0	36	0	1	1	Mutually_Exclusive	COL1A1, KAT6B	0/2
N4BP2	COL1A1	1	0	36	0	1	1	Mutually_Exclusive	COL1A1, N4BP2	0/2
NCOR1	COL1A1	1	0	36	0	1	1	Mutually_Exclusive	COL1A1, NCOR1	0/2
NF1	COL1A1	1	0	36	0	1	1	Mutually_Exclusive	COL1A1, NF1	0/2
NIN	COL1A1	1	0	36	0	1	1	Mutually_Exclusive	COL1A1, NIN	0/2
NSD1	COL1A1	1	0	36	0	1	1	Mutually_Exclusive	COL1A1, NSD1	0/2
POLD1	COL1A1	1	0	36	0	1	1	Mutually_Exclusive	COL1A1, POLD1	0/2
RANBP2	COL1A1	1	0	36	0	1	1	Mutually_Exclusive	COL1A1, RANBP2	0/2
RET	COL1A1	1	0	36	0	1	1	Mutually_Exclusive	COL1A1, RET	0/2
RNF213	COL1A1	1	0	36	0	1	1	Mutually_Exclusive	COL1A1, RNF213	0/2
SETD1B	COL1A1	1	0	36	0	1	1	Mutually_Exclusive	COL1A1, SETD1B	0/2
SPEN	COL1A1	1	0	36	0	1	1	Mutually_Exclusive	COL1A1, SPEN	0/2
STAG1	COL1A1	1	0	36	0	1	1	Mutually_Exclusive	COL1A1, STAG1	0/2
TERT	COL1A1	1	0	36	0	1	1	Mutually_Exclusive	COL1A1, TERT	0/2
TNC	COL1A1	1	0	36	0	1	1	Mutually_Exclusive	COL1A1, TNC	0/2
TPR	COL1A1	1	0	36	0	1	1	Mutually_Exclusive	COL1A1, TPR	0/2
ZMYM3	COL1A1	1	0	36	0	1	1	Mutually_Exclusive	COL1A1, ZMYM3	0/2
DCC	COL3A1	1	0	36	0	1	1	Mutually_Exclusive	COL3A1, DCC	0/2

DROSHA	COL3A1	1	0	36	0	1	1	Mutually_Exclusive	COL3A1, DROSHA	0/2
FANCA	COL3A1	1	0	36	0	1	1	Mutually_Exclusive	COL3A1, FANCA	0/2
FLNA	COL3A1	1	0	36	0	1	1	Mutually_Exclusive	COL3A1, FLNA	0/2
KAT6A	COL3A1	1	0	36	0	1	1	Mutually_Exclusive	COL3A1, KAT6A	0/2
KAT6B	COL3A1	1	0	36	0	1	1	Mutually_Exclusive	COL3A1, KAT6B	0/2
N4BP2	COL3A1	1	0	36	0	1	1	Mutually_Exclusive	COL3A1, N4BP2	0/2
NCOR1	COL3A1	1	0	36	0	1	1	Mutually_Exclusive	COL3A1, NCOR1	0/2
NF1	COL3A1	1	0	36	0	1	1	Mutually_Exclusive	COL3A1, NF1	0/2
NIN	COL3A1	1	0	36	0	1	1	Mutually_Exclusive	COL3A1, NIN	0/2
NSD1	COL3A1	1	0	36	0	1	1	Mutually_Exclusive	COL3A1, NSD1	0/2
POLD1	COL3A1	1	0	36	0	1	1	Mutually_Exclusive	COL3A1, POLD1	0/2
RANBP2	COL3A1	1	0	36	0	1	1	Mutually_Exclusive	COL3A1, RANBP2	0/2
RET	COL3A1	1	0	36	0	1	1	Mutually_Exclusive	COL3A1, RET	0/2
SETD1B	COL3A1	1	0	36	0	1	1	Mutually_Exclusive	COL3A1, SETD1B	0/2
SPEN	COL3A1	1	0	36	0	1	1	Mutually_Exclusive	COL3A1, SPEN	0/2
STAG1	COL3A1	1	0	36	0	1	1	Mutually_Exclusive	COL3A1, STAG1	0/2
TERT	COL3A1	1	0	36	0	1	1	Mutually_Exclusive	COL3A1, TERT	0/2
TNC	COL3A1	1	0	36	0	1	1	Mutually_Exclusive	COL3A1, TNC	0/2

TPR	COL3A1	1	0	36	0	1	1	Mutually_Exclusive	COL3A1, TPR	0/2
ZMYM3	COL3A1	1	0	36	0	1	1	Mutually_Exclusive	COL3A1, ZMYM3	0/2
DROSHA	DCC	1	0	36	0	1	1	Mutually_Exclusive	DCC, DROSHA	0/2
FANCA	DCC	1	0	36	0	1	1	Mutually_Exclusive	DCC, FANCA	0/2
FLNA	DCC	1	0	36	0	1	1	Mutually_Exclusive	DCC, FLNA	0/2
KAT6A	DCC	1	0	36	0	1	1	Mutually_Exclusive	DCC, KAT6A	0/2
KAT6B	DCC	1	0	36	0	1	1	Mutually_Exclusive	DCC, KAT6B	0/2
N4BP2	DCC	1	0	36	0	1	1	Mutually_Exclusive	DCC, N4BP2	0/2
NCOR1	DCC	1	0	36	0	1	1	Mutually_Exclusive	DCC, NCOR1	0/2
NF1	DCC	1	0	36	0	1	1	Mutually_Exclusive	DCC, NF1	0/2
NIN	DCC	1	0	36	0	1	1	Mutually_Exclusive	DCC, NIN	0/2
NSD1	DCC	1	0	36	0	1	1	Mutually_Exclusive	DCC, NSD1	0/2
POLD1	DCC	1	0	36	0	1	1	Mutually_Exclusive	DCC, POLD1	0/2
RANBP2	DCC	1	0	36	0	1	1	Mutually_Exclusive	DCC, RANBP2	0/2
RET	DCC	1	0	36	0	1	1	Mutually_Exclusive	DCC, RET	0/2
RNF213	DCC	1	0	36	0	1	1	Mutually_Exclusive	DCC, RNF213	0/2
SETD1B	DCC	1	0	36	0	1	1	Mutually_Exclusive	DCC, SETD1B	0/2
SPEN	DCC	1	0	36	0	1	1	Mutually_Exclusive	DCC, SPEN	0/2

STAG1	DCC	1	0	36	0	1	1	Mutually_Exclusive	DCC, STAG1	0/2
TERT	DCC	1	0	36	0	1	1	Mutually_Exclusive	DCC, TERT	0/2
TNC	DCC	1	0	36	0	1	1	Mutually_Exclusive	DCC, TNC	0/2
TPR	DCC	1	0	36	0	1	1	Mutually_Exclusive	DCC, TPR	0/2
ZMYM3	DCC	1	0	36	0	1	1	Mutually_Exclusive	DCC, ZMYM3	0/2
FANCA	DROSHA	1	0	36	0	1	1	Mutually_Exclusive	DROSHA, FANCA	0/2
FLNA	DROSHA	1	0	36	0	1	1	Mutually_Exclusive	DROSHA, FLNA	0/2
KAT6A	DROSHA	1	0	36	0	1	1	Mutually_Exclusive	DROSHA, KAT6A	0/2
KAT6B	DROSHA	1	0	36	0	1	1	Mutually_Exclusive	DROSHA, KAT6B	0/2
N4BP2	DROSHA	1	0	36	0	1	1	Mutually_Exclusive	DROSHA, N4BP2	0/2
NCOR1	DROSHA	1	0	36	0	1	1	Mutually_Exclusive	DROSHA, NCOR1	0/2
NF1	DROSHA	1	0	36	0	1	1	Mutually_Exclusive	DROSHA, NF1	0/2
NIN	DROSHA	1	0	36	0	1	1	Mutually_Exclusive	DROSHA, NIN	0/2
NSD1	DROSHA	1	0	36	0	1	1	Mutually_Exclusive	DROSHA, NSD1	0/2
POLD1	DROSHA	1	0	36	0	1	1	Mutually_Exclusive	DROSHA, POLD1	0/2
RANBP2	DROSHA	1	0	36	0	1	1	Mutually_Exclusive	DROSHA, RANBP2	0/2
RET	DROSHA	1	0	36	0	1	1	Mutually_Exclusive	DROSHA, RET	0/2
RNF213	DROSHA	1	0	36	0	1	1	Mutually_Exclusive	DROSHA, RNF213	0/2

SETD1B	DROSHA	1	0	36	0	1	1	Mutually_Exclusive	DROSHA, SETD1B	0/2
SPEN	DROSHA	1	0	36	0	1	1	Mutually_Exclusive	DROSHA, SPEN	0/2
STAG1	DROSHA	1	0	36	0	1	1	Mutually_Exclusive	DROSHA, STAG1	0/2
TERT	DROSHA	1	0	36	0	1	1	Mutually_Exclusive	DROSHA, TERT	0/2
TNC	DROSHA	1	0	36	0	1	1	Mutually_Exclusive	DROSHA, TNC	0/2
TPR	DROSHA	1	0	36	0	1	1	Mutually_Exclusive	DROSHA, TPR	0/2
ZMYM3	DROSHA	1	0	36	0	1	1	Mutually_Exclusive	DROSHA, ZMYM3	0/2
FLNA	FANCA	1	0	36	0	1	1	Mutually_Exclusive	FANCA, FLNA	0/2
KAT6A	FANCA	1	0	36	0	1	1	Mutually_Exclusive	FANCA, KAT6A	0/2
KAT6B	FANCA	1	0	36	0	1	1	Mutually_Exclusive	FANCA, KAT6B	0/2
N4BP2	FANCA	1	0	36	0	1	1	Mutually_Exclusive	FANCA, N4BP2	0/2
NCOR1	FANCA	1	0	36	0	1	1	Mutually_Exclusive	FANCA, NCOR1	0/2
NF1	FANCA	1	0	36	0	1	1	Mutually_Exclusive	FANCA, NF1	0/2
NIN	FANCA	1	0	36	0	1	1	Mutually_Exclusive	FANCA, NIN	0/2
NSD1	FANCA	1	0	36	0	1	1	Mutually_Exclusive	FANCA, NSD1	0/2
POLD1	FANCA	1	0	36	0	1	1	Mutually_Exclusive	FANCA, POLD1	0/2
RANBP2	FANCA	1	0	36	0	1	1	Mutually_Exclusive	FANCA, RANBP2	0/2
RET	FANCA	1	0	36	0	1	1	Mutually_Exclusive	FANCA, RET	0/2

RNF213	FANCA	1	0	36	0	1	1	Mutually_Exclusive	FANCA, RNF213	0/2
SETD1B	FANCA	1	0	36	0	1	1	Mutually_Exclusive	FANCA, SETD1B	0/2
SPEN	FANCA	1	0	36	0	1	1	Mutually_Exclusive	FANCA, SPEN	0/2
STAG1	FANCA	1	0	36	0	1	1	Mutually_Exclusive	FANCA, STAG1	0/2
TERT	FANCA	1	0	36	0	1	1	Mutually_Exclusive	FANCA, TERT	0/2
TNC	FANCA	1	0	36	0	1	1	Mutually_Exclusive	FANCA, TNC	0/2
TPR	FANCA	1	0	36	0	1	1	Mutually_Exclusive	FANCA, TPR	0/2
KAT6A	FLNA	1	0	36	0	1	1	Mutually_Exclusive	FLNA, KAT6A	0/2
KAT6B	FLNA	1	0	36	0	1	1	Mutually_Exclusive	FLNA, KAT6B	0/2
N4BP2	FLNA	1	0	36	0	1	1	Mutually_Exclusive	FLNA, N4BP2	0/2
NCOR1	FLNA	1	0	36	0	1	1	Mutually_Exclusive	FLNA, NCOR1	0/2
NF1	FLNA	1	0	36	0	1	1	Mutually_Exclusive	FLNA, NF1	0/2
NIN	FLNA	1	0	36	0	1	1	Mutually_Exclusive	FLNA, NIN	0/2
NSD1	FLNA	1	0	36	0	1	1	Mutually_Exclusive	FLNA, NSD1	0/2
POLD1	FLNA	1	0	36	0	1	1	Mutually_Exclusive	FLNA, POLD1	0/2
RANBP2	FLNA	1	0	36	0	1	1	Mutually_Exclusive	FLNA, RANBP2	0/2
RET	FLNA	1	0	36	0	1	1	Mutually_Exclusive	FLNA, RET	0/2
RNF213	FLNA	1	0	36	0	1	1	Mutually_Exclusive	FLNA, RNF213	0/2

SETD1B	FLNA	1	0	36	0	1	1	Mutually_Exclusive	FLNA, SETD1B	0/2
SPEN	FLNA	1	0	36	0	1	1	Mutually_Exclusive	FLNA, SPEN	0/2
STAG1	FLNA	1	0	36	0	1	1	Mutually_Exclusive	FLNA, STAG1	0/2
TERT	FLNA	1	0	36	0	1	1	Mutually_Exclusive	FLNA, TERT	0/2
TNC	FLNA	1	0	36	0	1	1	Mutually_Exclusive	FLNA, TNC	0/2
TPR	FLNA	1	0	36	0	1	1	Mutually_Exclusive	FLNA, TPR	0/2
ZMYM3	FLNA	1	0	36	0	1	1	Mutually_Exclusive	FLNA, ZMYM3	0/2
KAT6B	KAT6A	1	0	36	0	1	1	Mutually_Exclusive	KAT6A, KAT6B	0/2
N4BP2	KAT6A	1	0	36	0	1	1	Mutually_Exclusive	KAT6A, N4BP2	0/2
NCOR1	KAT6A	1	0	36	0	1	1	Mutually_Exclusive	KAT6A, NCOR1	0/2
NF1	KAT6A	1	0	36	0	1	1	Mutually_Exclusive	KAT6A, NF1	0/2
NIN	KAT6A	1	0	36	0	1	1	Mutually_Exclusive	KAT6A, NIN	0/2
NSD1	KAT6A	1	0	36	0	1	1	Mutually_Exclusive	KAT6A, NSD1	0/2
POLD1	KAT6A	1	0	36	0	1	1	Mutually_Exclusive	KAT6A, POLD1	0/2
RANBP2	KAT6A	1	0	36	0	1	1	Mutually_Exclusive	KAT6A, RANBP2	0/2
RET	KAT6A	1	0	36	0	1	1	Mutually_Exclusive	KAT6A, RET	0/2
RNF213	KAT6A	1	0	36	0	1	1	Mutually_Exclusive	KAT6A, RNF213	0/2
SETD1B	KAT6A	1	0	36	0	1	1	Mutually_Exclusive	KAT6A, SETD1B	0/2

SPEN	KAT6A	1	0	36	0	1	1	Mutually_Exclusive	KAT6A, SPEN	0/2
STAG1	KAT6A	1	0	36	0	1	1	Mutually_Exclusive	KAT6A, STAG1	0/2
TERT	KAT6A	1	0	36	0	1	1	Mutually_Exclusive	KAT6A, TERT	0/2
TNC	KAT6A	1	0	36	0	1	1	Mutually_Exclusive	KAT6A, TNC	0/2
TPR	KAT6A	1	0	36	0	1	1	Mutually_Exclusive	KAT6A, TPR	0/2
ZMYM3	KAT6A	1	0	36	0	1	1	Mutually_Exclusive	KAT6A, ZMYM3	0/2
N4BP2	KAT6B	1	0	36	0	1	1	Mutually_Exclusive	KAT6B, N4BP2	0/2
NCOR1	KAT6B	1	0	36	0	1	1	Mutually_Exclusive	KAT6B, NCOR1	0/2
NF1	KAT6B	1	0	36	0	1	1	Mutually_Exclusive	KAT6B, NF1	0/2
NIN	KAT6B	1	0	36	0	1	1	Mutually_Exclusive	KAT6B, NIN	0/2
NSD1	KAT6B	1	0	36	0	1	1	Mutually_Exclusive	KAT6B, NSD1	0/2
POLD1	KAT6B	1	0	36	0	1	1	Mutually_Exclusive	KAT6B, POLD1	0/2
RET	KAT6B	1	0	36	0	1	1	Mutually_Exclusive	KAT6B, RET	0/2
RNF213	KAT6B	1	0	36	0	1	1	Mutually_Exclusive	KAT6B, RNF213	0/2
SETD1B	KAT6B	1	0	36	0	1	1	Mutually_Exclusive	KAT6B, SETD1B	0/2
SPEN	KAT6B	1	0	36	0	1	1	Mutually_Exclusive	KAT6B, SPEN	0/2
STAG1	KAT6B	1	0	36	0	1	1	Mutually_Exclusive	KAT6B, STAG1	0/2
TERT	KAT6B	1	0	36	0	1	1	Mutually_Exclusive	KAT6B, TERT	0/2

TNC	KAT6B	1	0	36	0	1	1	Mutually_Exclusive	KAT6B, TNC	0/2
TPR	KAT6B	1	0	36	0	1	1	Mutually_Exclusive	KAT6B, TPR	0/2
ZMYM3	KAT6B	1	0	36	0	1	1	Mutually_Exclusive	KAT6B, ZMYM3	0/2
NCOR1	N4BP2	1	0	36	0	1	1	Mutually_Exclusive	N4BP2, NCOR1	0/2
NF1	N4BP2	1	0	36	0	1	1	Mutually_Exclusive	N4BP2, NF1	0/2
NIN	N4BP2	1	0	36	0	1	1	Mutually_Exclusive	N4BP2, NIN	0/2
NSD1	N4BP2	1	0	36	0	1	1	Mutually_Exclusive	N4BP2, NSD1	0/2
POLD1	N4BP2	1	0	36	0	1	1	Mutually_Exclusive	N4BP2, POLD1	0/2
RANBP2	N4BP2	1	0	36	0	1	1	Mutually_Exclusive	N4BP2, RANBP2	0/2
RET	N4BP2	1	0	36	0	1	1	Mutually_Exclusive	N4BP2, RET	0/2
RNF213	N4BP2	1	0	36	0	1	1	Mutually_Exclusive	N4BP2, RNF213	0/2
SETD1B	N4BP2	1	0	36	0	1	1	Mutually_Exclusive	N4BP2, SETD1B	0/2
SPEN	N4BP2	1	0	36	0	1	1	Mutually_Exclusive	N4BP2, SPEN	0/2
STAG1	N4BP2	1	0	36	0	1	1	Mutually_Exclusive	N4BP2, STAG1	0/2
TERT	N4BP2	1	0	36	0	1	1	Mutually_Exclusive	N4BP2, TERT	0/2
TNC	N4BP2	1	0	36	0	1	1	Mutually_Exclusive	N4BP2, TNC	0/2
TPR	N4BP2	1	0	36	0	1	1	Mutually_Exclusive	N4BP2, TPR	0/2
ZMYM3	N4BP2	1	0	36	0	1	1	Mutually_Exclusive	N4BP2, ZMYM3	0/2

NF1	NCOR1	1	0	36	0	1	1	Mutually_Exclusive	NCOR1, NF1	0/2
NIN	NCOR1	1	0	36	0	1	1	Mutually_Exclusive	NCOR1, NIN	0/2
NSD1	NCOR1	1	0	36	0	1	1	Mutually_Exclusive	NCOR1, NSD1	0/2
POLD1	NCOR1	1	0	36	0	1	1	Mutually_Exclusive	NCOR1, POLD1	0/2
RANBP2	NCOR1	1	0	36	0	1	1	Mutually_Exclusive	NCOR1, RANBP2	0/2
RET	NCOR1	1	0	36	0	1	1	Mutually_Exclusive	NCOR1, RET	0/2
RNF213	NCOR1	1	0	36	0	1	1	Mutually_Exclusive	NCOR1, RNF213	0/2
SETD1B	NCOR1	1	0	36	0	1	1	Mutually_Exclusive	NCOR1, SETD1B	0/2
SPEN	NCOR1	1	0	36	0	1	1	Mutually_Exclusive	NCOR1, SPEN	0/2
STAG1	NCOR1	1	0	36	0	1	1	Mutually_Exclusive	NCOR1, STAG1	0/2
TERT	NCOR1	1	0	36	0	1	1	Mutually_Exclusive	NCOR1, TERT	0/2
TNC	NCOR1	1	0	36	0	1	1	Mutually_Exclusive	NCOR1, TNC	0/2
TPR	NCOR1	1	0	36	0	1	1	Mutually_Exclusive	NCOR1, TPR	0/2
ZMYM3	NCOR1	1	0	36	0	1	1	Mutually_Exclusive	NCOR1, ZMYM3	0/2
NIN	NF1	1	0	36	0	1	1	Mutually_Exclusive	NF1, NIN	0/2
NSD1	NF1	1	0	36	0	1	1	Mutually_Exclusive	NF1, NSD1	0/2
POLD1	NF1	1	0	36	0	1	1	Mutually_Exclusive	NF1, POLD1	0/2
RANBP2	NF1	1	0	36	0	1	1	Mutually_Exclusive	NF1, RANBP2	0/2

RET	NF1	1	0	36	0	1	1	Mutually_Exclusive	NF1, RET	0/2
RNF213	NF1	1	0	36	0	1	1	Mutually_Exclusive	NF1, RNF213	0/2
SETD1B	NF1	1	0	36	0	1	1	Mutually_Exclusive	NF1, SETD1B	0/2
SPEN	NF1	1	0	36	0	1	1	Mutually_Exclusive	NF1, SPEN	0/2
STAG1	NF1	1	0	36	0	1	1	Mutually_Exclusive	NF1, STAG1	0/2
TERT	NF1	1	0	36	0	1	1	Mutually_Exclusive	NF1, TERT	0/2
TNC	NF1	1	0	36	0	1	1	Mutually_Exclusive	NF1, TNC	0/2
TPR	NF1	1	0	36	0	1	1	Mutually_Exclusive	NF1, TPR	0/2
ZMYM3	NF1	1	0	36	0	1	1	Mutually_Exclusive	NF1, ZMYM3	0/2
NSD1	NIN	1	0	36	0	1	1	Mutually_Exclusive	NIN, NSD1	0/2
POLD1	NIN	1	0	36	0	1	1	Mutually_Exclusive	NIN, POLD1	0/2
RANBP2	NIN	1	0	36	0	1	1	Mutually_Exclusive	NIN, RANBP2	0/2
RET	NIN	1	0	36	0	1	1	Mutually_Exclusive	NIN, RET	0/2
RNF213	NIN	1	0	36	0	1	1	Mutually_Exclusive	NIN, RNF213	0/2
SETD1B	NIN	1	0	36	0	1	1	Mutually_Exclusive	NIN, SETD1B	0/2
SPEN	NIN	1	0	36	0	1	1	Mutually_Exclusive	NIN, SPEN	0/2
STAG1	NIN	1	0	36	0	1	1	Mutually_Exclusive	NIN, STAG1	0/2
TERT	NIN	1	0	36	0	1	1	Mutually_Exclusive	NIN, TERT	0/2

TNC	NIN	1	0	36	0	1	1	Mutually_Exclusive	NIN, TNC	0/2
TPR	NIN	1	0	36	0	1	1	Mutually_Exclusive	NIN, TPR	0/2
ZMYM3	NIN	1	0	36	0	1	1	Mutually_Exclusive	NIN, ZMYM3	0/2
POLD1	NSD1	1	0	36	0	1	1	Mutually_Exclusive	NSD1, POLD1	0/2
RANBP2	NSD1	1	0	36	0	1	1	Mutually_Exclusive	NSD1, RANBP2	0/2
RET	NSD1	1	0	36	0	1	1	Mutually_Exclusive	NSD1, RET	0/2
RNF213	NSD1	1	0	36	0	1	1	Mutually_Exclusive	NSD1, RNF213	0/2
SPEN	NSD1	1	0	36	0	1	1	Mutually_Exclusive	NSD1, SPEN	0/2
STAG1	NSD1	1	0	36	0	1	1	Mutually_Exclusive	NSD1, STAG1	0/2
TERT	NSD1	1	0	36	0	1	1	Mutually_Exclusive	NSD1, TERT	0/2
TNC	NSD1	1	0	36	0	1	1	Mutually_Exclusive	NSD1, TNC	0/2
TPR	NSD1	1	0	36	0	1	1	Mutually_Exclusive	NSD1, TPR	0/2
ZMYM3	NSD1	1	0	36	0	1	1	Mutually_Exclusive	NSD1, ZMYM3	0/2
RANBP2	POLD1	1	0	36	0	1	1	Mutually_Exclusive	POLD1, RANBP2	0/2
RET	POLD1	1	0	36	0	1	1	Mutually_Exclusive	POLD1, RET	0/2
RNF213	POLD1	1	0	36	0	1	1	Mutually_Exclusive	POLD1, RNF213	0/2
SETD1B	POLD1	1	0	36	0	1	1	Mutually_Exclusive	POLD1, SETD1B	0/2
SPEN	POLD1	1	0	36	0	1	1	Mutually_Exclusive	POLD1, SPEN	0/2

STAG1	POLD1	1	0	36	0	1	1	Mutually_Exclusive	POLD1, STAG1	0/2
TERT	POLD1	1	0	36	0	1	1	Mutually_Exclusive	POLD1, TERT	0/2
TNC	POLD1	1	0	36	0	1	1	Mutually_Exclusive	POLD1, TNC	0/2
TPR	POLD1	1	0	36	0	1	1	Mutually_Exclusive	POLD1, TPR	0/2
ZMYM3	POLD1	1	0	36	0	1	1	Mutually_Exclusive	POLD1, ZMYM3	0/2
RET	RANBP2	1	0	36	0	1	1	Mutually_Exclusive	RANBP2, RET	0/2
RNF213	RANBP2	1	0	36	0	1	1	Mutually_Exclusive	RANBP2, RNF213	0/2
SETD1B	RANBP2	1	0	36	0	1	1	Mutually_Exclusive	RANBP2, SETD1B	0/2
SPEN	RANBP2	1	0	36	0	1	1	Mutually_Exclusive	RANBP2, SPEN	0/2
STAG1	RANBP2	1	0	36	0	1	1	Mutually_Exclusive	RANBP2, STAG1	0/2
TERT	RANBP2	1	0	36	0	1	1	Mutually_Exclusive	RANBP2, TERT	0/2
TNC	RANBP2	1	0	36	0	1	1	Mutually_Exclusive	RANBP2, TNC	0/2
TPR	RANBP2	1	0	36	0	1	1	Mutually_Exclusive	RANBP2, TPR	0/2
ZMYM3	RANBP2	1	0	36	0	1	1	Mutually_Exclusive	RANBP2, ZMYM3	0/2
RNF213	RET	1	0	36	0	1	1	Mutually_Exclusive	RET, RNF213	0/2
SETD1B	RET	1	0	36	0	1	1	Mutually_Exclusive	RET, SETD1B	0/2
SPEN	RET	1	0	36	0	1	1	Mutually_Exclusive	RET, SPEN	0/2
STAG1	RET	1	0	36	0	1	1	Mutually_Exclusive	RET, STAG1	0/2

TERT	RET	1	0	36	0	1	1	Mutually_Exclusive	RET, TERT	0/2
TNC	RET	1	0	36	0	1	1	Mutually_Exclusive	RET, TNC	0/2
TPR	RET	1	0	36	0	1	1	Mutually_Exclusive	RET, TPR	0/2
ZMYM3	RET	1	0	36	0	1	1	Mutually_Exclusive	RET, ZMYM3	0/2
SETD1B	RNF213	1	0	36	0	1	1	Mutually_Exclusive	RNF213, SETD1B	0/2
SPEN	RNF213	1	0	36	0	1	1	Mutually_Exclusive	RNF213, SPEN	0/2
STAG1	RNF213	1	0	36	0	1	1	Mutually_Exclusive	RNF213, STAG1	0/2
TERT	RNF213	1	0	36	0	1	1	Mutually_Exclusive	RNF213, TERT	0/2
TNC	RNF213	1	0	36	0	1	1	Mutually_Exclusive	RNF213, TNC	0/2
TPR	RNF213	1	0	36	0	1	1	Mutually_Exclusive	RNF213, TPR	0/2
ZMYM3	RNF213	1	0	36	0	1	1	Mutually_Exclusive	RNF213, ZMYM3	0/2
SPEN	SETD1B	1	0	36	0	1	1	Mutually_Exclusive	SETD1B, SPEN	0/2
STAG1	SETD1B	1	0	36	0	1	1	Mutually_Exclusive	SETD1B, STAG1	0/2
TERT	SETD1B	1	0	36	0	1	1	Mutually_Exclusive	SETD1B, TERT	0/2
TNC	SETD1B	1	0	36	0	1	1	Mutually_Exclusive	SETD1B, TNC	0/2
TPR	SETD1B	1	0	36	0	1	1	Mutually_Exclusive	SETD1B, TPR	0/2
ZMYM3	SETD1B	1	0	36	0	1	1	Mutually_Exclusive	SETD1B, ZMYM3	0/2
STAG1	SPEN	1	0	36	0	1	1	Mutually_Exclusive	SPEN, STAG1	0/2

TERT	SPEN	1	0	36	0	1	1	Mutually_Exclusive	SPEN, TERT	0/2
TNC	SPEN	1	0	36	0	1	1	Mutually_Exclusive	SPEN, TNC	0/2
TPR	SPEN	1	0	36	0	1	1	Mutually_Exclusive	SPEN, TPR	0/2
ZMYM3	SPEN	1	0	36	0	1	1	Mutually_Exclusive	SPEN, ZMYM3	0/2
TERT	STAG1	1	0	36	0	1	1	Mutually_Exclusive	STAG1, TERT	0/2
TNC	STAG1	1	0	36	0	1	1	Mutually_Exclusive	STAG1, TNC	0/2
ZMYM3	STAG1	1	0	36	0	1	1	Mutually_Exclusive	STAG1, ZMYM3	0/2
TNC	TERT	1	0	36	0	1	1	Mutually_Exclusive	TERT, TNC	0/2
TPR	TERT	1	0	36	0	1	1	Mutually_Exclusive	TERT, TPR	0/2
ZMYM3	TERT	1	0	36	0	1	1	Mutually_Exclusive	TERT, ZMYM3	0/2
TPR	TNC	1	0	36	0	1	1	Mutually_Exclusive	TNC, TPR	0/2
ZMYM3	TNC	1	0	36	0	1	1	Mutually_Exclusive	TNC, ZMYM3	0/2
ZMYM3	TPR	1	0	36	0	1	1	Mutually_Exclusive	TPR, ZMYM3	0/2
