# Supplementary material for: Genomic and immunological profiles of small-cell lung cancer between East Asians and Caucasian
Source: Cancer Cell Int. 2022 Apr 29;22:173. doi: 10.1186/s12935-022-02588-w (PMC9052616; doi:10.1186/s12935-022-02588-w)
Supplement: Supplementary file 14 — Additional file 14: Table S5. Related to Additional file 5: Fig. S5a. The results of the CMap analysis of the East Asian cohort (TP53/RB1 co-mutations vs No alterations in TP53/RB1). [file 12935_2022_2588_MOESM14_ESM.pdf]

Supplementary Table.5 Related to Supplementary Fig. 5a The results of the CMap analysis of the EA cohort (TP53/RB1 co-mutations vs No alterations in TP53/RB1).

rank	cmap name	mean	n	enrichment	p	specificity	percent non-null
1	hycanthone	0.649	4	0.958	0	0.0259	100
2	tanespimycin	-0.373	62	-0.412	0	0.1344	54
3	metronidazole	0.536	5	0.85	0.00018	0.006	100
4	monensin	0.37	6	0.686	0.00242	0.09	83
5	5255229	-0.706	2	-0.965	0.00274	0.0082	100
6	thapsigargin	-0.645	3	-0.886	0.00288	0.0903	100
7	bucladesine	-0.474	6	-0.674	0.00326	0	83
8	pinacidil	0.206	4	0.772	0.00515	0.0076	50
9	SC-560	-0.471	3	-0.834	0.00907	0.0546	100
10	mitoxantrone	0.394	3	0.832	0.00951	0.0643	66
11	16-phenyltetranorprostaglandin E2	-0.439	4	-0.729	0.01094	0.0119	75
12	iproniazid	0.365	5	0.658	0.0124	0.0503	80
13	mianserin	-0.472	5	-0.652	0.01252	0	80
14	xamoterol	-0.372	3	-0.814	0.0126	0.0343	66
15	STOCK1N-35215	0.272	3	0.813	0.01324	0.0337	66
16	5279552	-0.614	2	-0.918	0.01374	0.0119	100
17	tacrine	-0.42	4	-0.71	0.01466	0.0065	75
18	emetine	-0.512	4	-0.701	0.01671	0.2353	75
19	mebhydrolin	0.28	4	0.698	0.01713	0.0104	75
20	levomepromazine	0.498	4	0.697	0.01753	0.1561	75
21	dihydrostreptomycin	0.131	5	0.635	0.01852	0.0137	60
22	withaferin A	0.31	4	0.692	0.0188	0.2421	50
23	isocorydine	0.226	4	0.688	0.02005	0.0121	75
24	diphemanil metilsulfate	-0.342	5	-0.618	0.02169	0.035	80
25	STOCK1N-28457	-0.398	3	-0.779	0.02217	0.0355	66
26	furazolidone	0.382	4	0.681	0.02226	0.0839	75
27	PNU-0230031	-0.24	8	-0.497	0.02383	0.0625	62
28	CP-690334-01	0.262	8	0.497	0.02399	0.1455	50
29	melatonin	0.398	4	0.676	0.02417	0.0321	75
30	irinotecan	0.583	3	0.769	0.0245	0.2909	66
31	piperacillin	-0.396	5	-0.609	0.02577	0.0325	60
32	lycorine	-0.302	5	-0.609	0.02597	0.2333	60
33	0317956-0000	-0.158	8	-0.49	0.0274	0.1073	50
34	NU-1025	-0.562	2	-0.883	0.02744	0.0376	100
35	indometacin	-0.377	8	-0.489	0.0276	0.0217	62
36	alvespimycin	-0.29	12	-0.402	0.02948	0.2101	50
37	terazosin	0.454	4	0.658	0.03171	0.1124	75
38	oxedrine	0.367	4	0.652	0.03416	0.0273	75
39	arecoline	0.404	4	0.646	0.03776	0.0533	75
40	meclocycline	0.378	4	0.643	0.03949	0.05	75
41	hemicholinium	-0.324	4	-0.631	0.04502	0.0722	50
42	ionomycin	0.328	3	0.711	0.04697	0.1211	66
43	cyproterone	0.291	4	0.629	0.04729	0.064	50
44	harmol	0.428	4	0.625	0.04943	0.093	75
45	isoxsuprine	-0.281	5	-0.553	0.05529	0.0206	60
46	MS-275	0.253	2	0.834	0.05563	0.2035	50
47	novobiocin	0.258	9	0.421	0.05755	0.0692	55
48	orciprenaline	-0.412	4	-0.611	0.05835	0.0286	75
49	digoxin	-0.452	4	-0.61	0.05928	0.1837	75
50	etofenamate	0.201	4	0.608	0.06045	0.0614	50
51	puromycin	-0.475	4	-0.604	0.06366	0.2537	75
52	ouabain	-0.466	4	-0.603	0.06461	0.1579	75
53	glafenine	-0.444	4	-0.601	0.06604	0.1521	75
54	scopolamine	0.227	4	0.599	0.06766	0.0606	50

55	cefalotin	-0.408	4	-0.597	0.06925	0.0704	75
56	amoxapine	-0.336	5	-0.533	0.07287	0.1154	60
57	gemfibrozil	-0.259	5	-0.533	0.07293	0.1053	60
58	equilin	0.206	5	0.535	0.0733	0.1207	60
59	5194442	0.313	4	0.59	0.07454	0.1161	75
60	metanephrine	-0.371	5	-0.53	0.0762	0.1083	60
61	desipramine	0.224	4	0.588	0.07665	0.2579	50
62	rolipram	-0.39	4	-0.588	0.0776	0.0592	75
63	methotrexate	0.257	8	0.425	0.07792	0.2711	50
64	proxyphylline	-0.37	4	-0.587	0.07834	0.1782	75
65	albendazole	-0.411	3	-0.652	0.08375	0.1643	66
66	ethosuximide	-0.391	4	-0.58	0.08494	0.0805	75
67	cefepime	0.268	4	0.577	0.08582	0.0645	50
68	exisulind	-0.393	2	-0.788	0.08764	0.069	50
69	AG-012559	-0.472	3	-0.647	0.08875	0.0815	66
70	vanoxerine	0.193	4	0.574	0.0892	0.2031	50
71	5707885	0.04	4	0.573	0.08976	0.3627	50
72	depudecin	0.357	2	0.781	0.09682	0.075	50
73	quinethazone	-0.308	4	-0.568	0.09734	0.2158	75
74	chlortetracycline	0.332	5	0.512	0.09757	0.0107	60
75	pentetic acid	-0.324	5	-0.508	0.09891	0.1063	60
76	parthenolide	0.232	4	0.561	0.10151	0.4311	50
77	(+/-)-catechin	-0.23	4	-0.563	0.10153	0.0641	50
78	dicoumarol	-0.306	6	-0.465	0.10411	0.0333	50
79	dicycloverine	-0.34	5	-0.504	0.10478	0.2137	60
80	dioxybenzone	0.209	4	0.558	0.10519	0.1032	50
81	triflupromazine	0.278	4	0.557	0.10581	0.2245	50
82	estrone	-0.316	4	-0.56	0.10583	0.092	50
83	dl-alpha tocopherol	0.223	4	0.555	0.10768	0.0233	50
84	denatonium benzoate	0.136	4	0.553	0.11047	0.1513	50
85	deptropine	0.273	4	0.552	0.1109	0.1629	50
86	pimethixene	0.372	3	0.624	0.11199	0.152	66
87	(+)-chelidonine	-0.261	4	-0.552	0.11385	0.1308	50
88	sisomicin	0.204	4	0.549	0.1144	0.038	50
89	theobromine	-0.284	4	-0.552	0.11532	0.1317	50
90	dilazep	0.177	5	0.498	0.11577	0.1677	60
91	metoprolol	0.205	4	0.547	0.11747	0.1547	50
92	nomegestrol	0.372	3	0.618	0.118	0.2046	66
93	erastin	0.21	4	0.544	0.12097	0.2023	50
94	valinomycin	-0.286	4	-0.547	0.12129	0.2429	50
95	ethionamide	-0.388	3	-0.614	0.12367	0.1375	66
96	chlorphenamine	-0.221	4	-0.543	0.12515	0.1123	50
97	ascorbic acid	-0.24	4	-0.543	0.1255	0.1012	50
98	dropropizine	-0.312	4	-0.542	0.12638	0.1547	50
99	raubasine	0.243	4	0.539	0.12775	0.1667	50
100	Prestwick-675	-0.252	4	-0.541	0.12801	0.4021	50
101	piroxicam	-0.254	4	-0.54	0.12875	0.2222	50
102	etoposide	0.298	4	0.538	0.12905	0.3795	50
103	daunorubicin	0.324	4	0.536	0.13121	0.4293	50
104	minaprine	-0.335	5	-0.485	0.13177	0.1698	60
105	megestrol	0.186	4	0.535	0.13292	0.2546	50
106	atractyloside	0.281	5	0.484	0.13498	0.4667	60
107	oxymetazoline	-0.304	4	-0.535	0.13555	0.1325	50
108	diphenhydramine	0.323	5	0.482	0.13868	0.4011	60
109	tiapride	0.335	5	0.481	0.13926	0.0956	60
110	Prestwick-857	0.272	4	0.527	0.14305	0.3226	50
111	perphenazine	0.152	5	0.477	0.14621	0.4536	60
112	clofilium tosylate	-0.448	3	-0.596	0.1467	0.1343	66
113	cephaeline	-0.367	5	-0.473	0.1511	0.6807	60
114	alprenolol	-0.264	4	-0.521	0.15341	0.1393	50
115	rofecoxib	0.173	6	0.432	0.15391	0.1314	50

116	amprolium	-0.346	5	-0.468	0.15855	0.2473	60
117	promethazine	0.254	4	0.515	0.15896	0.2292	50
118	piracetam	-0.272	4	-0.517	0.15944	0.1829	50
119	SR-95531	-0.267	4	-0.516	0.16137	0.1902	50
120	desoxycortone	0.279	4	0.513	0.16191	0.1	50
121	niridazole	-0.303	4	-0.514	0.16463	0.1858	50
122	colistin	-0.339	4	-0.512	0.16774	0.3154	50
123	lanatoside C	-0.236	6	-0.424	0.17137	0.3192	50
124	tetrandrine	0.073	4	0.506	0.17329	0.3824	50
125	difenidol	-0.402	3	-0.577	0.17555	0.2944	66
126	penbutolol	0.329	3	0.577	0.17773	0.4124	66
127	colchicine	-0.264	6	-0.419	0.18146	0.2535	50
128	ciprofibrate	0.307	4	0.5	0.18264	0.1908	50
129	pseudopelletierine	0.169	4	0.499	0.18355	0.156	50
130	cinoxacin	-0.276	4	-0.497	0.1905	0.2611	50
131	pirlindole	0.204	3	0.569	0.19107	0.2061	66
132	piperacetazine	0.231	4	0.49	0.19875	0.2914	50
133	atracurium besilate	0.348	3	0.562	0.20277	0.422	66
134	oxyphenbutazone	0.235	4	0.487	0.20486	0.4093	50
135	dobutamine	0.194	4	0.484	0.20973	0.2126	50
136	bacitracin	-0.231	3	-0.558	0.2102	0.2242	66
137	primidone	-0.225	4	-0.482	0.21741	0.2826	50
138	milrinone	-0.396	3	-0.553	0.21811	0.5	66
139	securinine	-0.362	4	-0.479	0.22382	0.3861	50
140	biotin	-0.213	3	-0.546	0.23155	0.1307	66
141	prazosin	0.06	6	0.396	0.23209	0.1936	50
142	L-methionine sulfoximine	-0.35	4	-0.472	0.23885	0.1657	50
143	folic acid	0.331	4	0.469	0.24061	0.375	50
144	domperidone	0.255	4	0.465	0.24998	0.1749	50
145	brinzolamide	-0.169	4	-0.467	0.25035	0.2757	50
146	pipenzolate bromide	0.2	4	0.464	0.25175	0.2555	50
147	digitoxigenin	-0.18	4	-0.466	0.25383	0.402	50
148	diprophylline	-0.22	5	-0.422	0.25619	0.3444	60
149	methoxsalen	-0.14	3	-0.533	0.25751	0.2069	66
150	lasalocid	0.331	4	0.461	0.25815	0.6373	50
151	metitepine	0.071	4	0.461	0.25847	0.4264	50
152	velnacrine	0.286	4	0.457	0.26808	0.1958	50
153	etifenin	0.024	4	0.456	0.26983	0.2246	50
154	trapidil	0.28	3	0.528	0.26991	0.4333	66
155	convolamine	0.27	4	0.451	0.28286	0.189	50
156	PNU-0251126	0.102	6	0.376	0.28364	0.5	50
157	methocarbamol	-0.138	3	-0.521	0.28371	0.3351	66
158	butein	0.253	2	0.623	0.28437	0.2983	50
159	cinchonidine	-0.216	4	-0.447	0.29762	0.2397	50
160	sulfanilamide	-0.321	4	-0.446	0.30084	0.2611	50
161	suramin sodium	0.053	4	0.443	0.30241	0.3082	50
162	diperodon	-0.383	3	-0.51	0.30595	0.2713	66
163	cyclobenzaprine	0.287	4	0.441	0.30828	0.5417	50
164	canrenoic acid	0.119	4	0.44	0.31162	0.3053	50
165	bupropion	0.115	4	0.44	0.31214	0.338	50
166	ethaverine	-0.262	4	-0.441	0.31397	0.3822	50
167	dyclonine	-0.254	4	-0.438	0.32085	0.3015	50
168	nimesulide	-0.147	4	-0.433	0.33621	0.3161	50
169	parbendazole	-0.263	4	-0.432	0.33792	0.3646	50
170	fosfosal	-0.311	4	-0.432	0.3392	0.3392	50
171	loxapine	-0.329	4	-0.431	0.34025	0.2419	50
172	chlorzoxazone	-0.16	4	-0.431	0.34067	0.6332	50
173	naringenin	0.289	4	0.429	0.34132	0.7075	50
174	idazoxan	-0.223	4	-0.428	0.34779	0.4022	50
175	5182598	-0.373	2	-0.584	0.34795	0.6478	50
176	PHA-00846566E	-0.231	3	-0.493	0.34833	0.3352	66

177	karakoline	0.17	6	0.353	0.35173	0.5508	50
178	ampicillin	0.268	4	0.425	0.3525	0.3726	50
179	xylometazoline	-0.316	4	-0.427	0.35312	0.4917	50
180	perhexiline	-0.235	4	-0.427	0.35402	0.4706	50
181	nitrofural	0.21	4	0.423	0.35883	0.3726	50
182	5155877	0.245	4	0.419	0.36943	0.5125	50
183	helveticoside	0.028	6	0.344	0.38203	0.8468	50
184	dorzolamide	-0.265	4	-0.416	0.38376	0.4315	50
185	oxolamine	0.117	4	0.411	0.39426	0.6209	50
186	fusaric acid	-0.303	4	-0.412	0.39561	0.5465	50
187	fluoxetine	0.285	4	0.409	0.39993	0.5729	50
188	quinisocaine	-0.186	4	-0.411	0.40015	0.36	50
189	meteneprost	0.244	4	0.405	0.41192	0.7609	50
190	pridinol	-0.267	4	-0.406	0.41706	0.4546	50
191	naftifine	-0.318	4	-0.405	0.41932	0.4837	50
192	cicloheximide	-0.285	4	-0.402	0.42929	0.6519	50
193	methapyrilene	-0.144	4	-0.401	0.43138	0.4637	50
194	beta-escin	-0.245	6	-0.329	0.43636	0.4711	50
195	pivmecillinam	-0.072	4	-0.399	0.43697	0.5691	50
196	flunisolide	-0.273	6	-0.329	0.4391	0.5118	50
197	epiandrosterone	-0.172	4	-0.398	0.44099	0.5672	50
198	sulfapyridine	-0.158	4	-0.397	0.44224	0.4071	50
199	kaempferol	0.243	4	0.395	0.4461	0.5	50
200	blebbistatin	-0.351	2	-0.524	0.45244	0.696	50
201	diloxanide	0.226	4	0.392	0.45615	0.6919	50
202	tubocurarine chloride	-0.172	4	-0.391	0.46166	0.4822	50
203	ciclacillin	-0.24	4	-0.389	0.46828	0.4581	50
204	hesperidin	-0.264	4	-0.389	0.46904	0.4343	50
205	clofibrate	-0.248	2	-0.511	0.47759	0.3129	50
206	methylbenzethonium chloride	-0.274	6	-0.319	0.47829	0.562	50
207	ketorolac	0.081	4	0.384	0.47998	0.4746	50
208	ondansetron	0.122	4	0.382	0.48674	0.6872	50
209	zoxazolamine	-0.175	4	-0.384	0.48688	0.6717	50
210	bisoprolol	-0.186	4	-0.384	0.48718	0.4807	50
211	buspirone	-0.24	4	-0.383	0.48917	0.4329	50
212	clorsulon	-0.042	4	-0.383	0.48951	0.6667	50
213	colecalciferol	-0.253	4	-0.382	0.49054	0.4239	50
214	thiethylperazine	-0.146	4	-0.382	0.49054	0.3769	50
215	5248896	-0.49	2	-0.5	0.49964	0.7368	50
216	gliquidone	0.218	4	0.378	0.50138	0.4114	50
217	tiratricol	-0.324	4	-0.378	0.50522	0.5529	50
218	5224221	-0.384	2	-0.498	0.50781	0.6842	50
219	estriol	-0.317	4	-0.377	0.50964	0.7376	50
220	mesoridazine	-0.144	4	-0.377	0.51069	0.5027	50
221	salsolidin	-0.278	4	-0.376	0.51189	0.5584	50
222	alcuronium chloride	-0.411	2	-0.497	0.51243	0.5876	50
223	5230742	-0.253	2	-0.495	0.51958	0.6503	50
224	metaraminol	-0.221	4	-0.373	0.52171	0.5419	50
225	gabapentin	-0.274	4	-0.371	0.52895	0.5333	50
226	mepacrine	-0.394	2	-0.492	0.53145	0.4167	50
227	chenodeoxycholic acid	0.236	4	0.369	0.53309	0.7857	50
228	oxybutynin	-0.263	4	-0.37	0.53423	0.6025	50
229	florfenicol	-0.265	4	-0.367	0.54457	0.5346	50
230	ursolic acid	-0.24	4	-0.365	0.55271	0.6715	50
231	norethisterone	-0.331	4	-0.363	0.55979	0.6646	50
232	hymecromone	0.15	4	0.361	0.56116	0.6301	50
233	0198306-0000	0.036	4	0.359	0.56643	0.5441	50
234	oxybenzone	0.248	4	0.359	0.56643	0.7412	50
235	homosalate	-0.29	4	-0.36	0.57005	0.5515	50
236	spaglumic acid	-0.365	2	-0.481	0.57253	0.6559	50

237	CP-863187	0.21	4	0.356	0.57682	0.662	50
238	retrorsine	-0.145	4	-0.357	0.57819	0.5562	50
239	dydrogesterone	-0.268	4	-0.357	0.57962	0.5127	50
240	mephenytoin	0.032	4	0.355	0.58034	0.6509	50
241	phensuximide	0.083	4	0.355	0.58151	0.6274	50
242	racecadotril	-0.153	4	-0.356	0.58298	0.5577	50
243	4,5-dianilinophthalimide	-0.232	2	-0.477	0.58617	0.7989	50
244	chloramphenicol	-0.208	4	-0.354	0.58732	0.6667	50
245	pargyline	-0.271	4	-0.352	0.5951	0.8054	50
246	cyclic adenosine monophosphate	0.176	4	0.351	0.59693	0.7595	50
247	benfluorex	0.037	4	0.35	0.59743	0.6163	50
248	isosorbide	-0.087	4	-0.351	0.59872	0.5489	50
249	piperine	0.229	4	0.35	0.5988	0.5563	50
250	tetramisole	0.043	4	0.349	0.60079	0.4706	50
251	etamivan	-0.284	4	-0.35	0.60325	0.71	50
252	suloctidil	-0.292	4	-0.349	0.60508	0.7065	50
253	5253409	-0.062	2	-0.472	0.60575	0.4359	50
254	nimodipine	0.197	4	0.347	0.60944	0.7037	50
255	methazolamide	-0.275	4	-0.346	0.61613	0.7816	50
256	canadine	0.211	4	0.345	0.61682	0.865	50
257	cromoglicic acid	-0.331	2	-0.466	0.62563	0.6188	50
258	pepstatin	0.066	4	0.342	0.62573	0.6111	50
259	benzylpenicillin	-0.243	4	-0.343	0.62919	0.6464	50
260	arachidonyltrifluoromethane	-0.338	2	-0.462	0.64195	0.634	50
261	esculin	-0.212	4	-0.337	0.64959	0.7225	50
262	bezafibrate	-0.251	4	-0.336	0.65102	0.7673	50
263	quinostatin	-0.334	2	-0.459	0.6513	0.8626	50
264	(-)-isoprenaline	-0.074	4	-0.336	0.65159	0.5571	50
265	thiostrepton	-0.238	4	-0.333	0.66309	0.75	50
266	nialamide	-0.18	4	-0.332	0.66653	0.7761	50
267	chloropyrazine	-0.163	4	-0.327	0.68108	0.8442	50
268	cetirizine	-0.295	4	-0.327	0.68153	0.8599	50
269	azlocillin	-0.17	4	-0.325	0.68692	0.7598	50
270	prasterone	-0.133	4	-0.324	0.69182	0.664	50
271	chloroquine	-0.145	4	-0.323	0.69502	0.7273	50
272	trichlormethiazide	-0.182	4	-0.32	0.70584	0.75	50
273	molindone	-0.26	4	-0.318	0.71046	0.8385	50
274	papaverine	-0.232	4	-0.318	0.71259	0.6793	50
275	imatinib	0.24	2	0.421	0.76592	0.8704	50
276	mercaptopurine	-0.09	2	-0.419	0.77132	0.8082	50
277	W-13	0.221	2	0.4	0.81995	0.8834	50
278	STOCK1N-35874	0.178	2	0.356	0.91085	0.9565	50
279	BW-B70C	0.81	1	0.999	---	---	100
280	tyrphostin AG-1478	0.707	1	0.996	---	---	100
281	cantharidin	-0.775	1	-0.99	---	---	100
282	demecolcine	-0.761	1	-0.987	---	---	100
283	celastrol	-0.749	1	-0.985	---	---	100
284	cytochalasin B	-0.72	1	-0.978	---	---	100
285	MG-132	-0.693	1	-0.969	---	---	100
286	HNMPA-(AM)3	0.574	1	0.966	---	---	100
287	tioguanine	0.561	1	0.961	---	---	100
288	dopamine	-0.66	1	-0.956	---	---	100
289	(-)-catechin	-0.652	1	-0.953	---	---	100
290	gefitinib	0.53	1	0.947	---	---	100
291	decitabine	0.526	1	0.944	---	---	100
292	5213008	-0.605	1	-0.93	---	---	100
293	5666823	0.487	1	0.924	---	---	100
294	1,5-isoquinolinediol	0.477	1	0.919	---	---	100
295	tomelukast	0.476	1	0.919	---	---	100

296	PHA-00665752	-0.535	1	-0.896	---	---	100
297	U0125	0.347	1	0.854	---	---	100
298	tyrphostin AG-825	0	1	0.834	---	---	0
299	2-deoxy-D-glucose	0	1	-0.806	---	---	0
300	camptothecin	0.141	3	0.804	---	---	33
301	topiramate	0	1	-0.8	---	---	0
302	phenanthridinone	0	1	-0.779	---	---	0
303	alfaxalone	0.173	3	0.749	---	---	33
304	reserpine	0.121	3	0.744	---	---	33
305	(-)-atenolol	0.068	4	0.744	---	---	25
306	quinpirole	0	4	-0.744	---	---	0
307	thioguanosine	0	4	0.743	---	---	0
308	12,13-EODE	0	1	-0.74	---	---	0
309	betulin	-0.148	3	-0.734	---	---	33
310	Prestwick-983	0	3	-0.732	---	---	0
311	5186223	0	1	-0.731	---	---	0
312	disopyramide	-0.165	4	-0.729	---	---	25
313	oxamic acid	0	1	0.722	---	---	0
314	sulmazole	-0.229	3	-0.72	---	---	33
315	DL-PPMP	0	1	-0.719	---	---	0
316	5149715	0	1	-0.718	---	---	0
317	timolol	-0.109	4	-0.713	---	---	25
318	dexverapamil	0	1	0.712	---	---	0
319	calmidazolium	0	2	0.705	---	---	0
320	HC toxin	0	1	0.698	---	---	0
321	apigenin	0	4	0.691	---	---	0
322	5162773	0	1	0.69	---	---	0
323	trifluridine	0	4	0.689	---	---	0
324	viomycin	0	4	-0.687	---	---	0
325	vigabatrin	-0.175	3	-0.686	---	---	33
326	piperidolate	0	3	0.683	---	---	0
327	Prestwick-691	-0.132	3	-0.682	---	---	33
328	oligomycin	0	1	-0.68	---	---	0
329	trihexyphenidyl	-0.171	3	-0.674	---	---	33
330	terfenadine	0	3	0.672	---	---	0
331	phenoxybenzamine	0	4	0.672	---	---	0
332	tomatidine	0.11	4	0.67	---	---	25
333	isoflupredone	0	3	-0.659	---	---	0
334	5151277	0	1	-0.658	---	---	0
335	dextromethorphan	0.124	4	0.658	---	---	25
336	5140203	0	1	0.655	---	---	0
337	astemizole	0.136	5	0.655	---	---	20
338	sanguinarine	0	2	0.654	---	---	0
339	5186324	0	1	-0.653	---	---	0
340	H-7	0	4	0.653	---	---	0
341	cortisone	0	3	0.651	---	---	0
342	doxorubicin	0.191	3	0.647	---	---	33
343	felbinac	-0.124	4	-0.646	---	---	25
344	clebopride	-0.108	4	-0.646	---	---	25
345	pararosaniline	0	1	0.645	---	---	0
346	fasudil	0	2	-0.642	---	---	0
347	lidoflazine	-0.136	3	-0.642	---	---	33
348	MK-886	0	2	-0.64	---	---	0
349	trimipramine	0.15	4	0.638	---	---	25
350	nizatidine	-0.119	4	-0.635	---	---	25
351	norcyclobenzaprine	0.09	4	0.634	---	---	25
352	5109870	0	1	0.634	---	---	0
353	clobetasol	0.153	3	0.633	---	---	33
354	phenazone	-0.142	3	-0.63	---	---	33
355	biperiden	0	5	-0.629	---	---	0
356	clorgiline	0.115	4	0.629	---	---	25

357	methylprednisolone	0.109	4	0.629	---	---	25
358	phenyl biguanide	0	1	-0.628	---	---	0
359	splitomicin	0	1	-0.627	---	---	0
360	5114445	0	1	0.626	---	---	0
361	sulindac sulfide	0	1	-0.626	---	---	0
362	menadione	0	2	0.625	---	---	0
363	tobramycin	0.097	4	0.625	---	---	25
364	thiamphenicol	0	5	-0.624	---	---	0
365	fisetin	0	1	0.624	---	---	0
366	STOCK1N-35696	0	2	-0.621	---	---	0
367	remoxipride	0.1	4	0.62	---	---	25
368	amiodarone	0.191	5	0.617	---	---	40
369	ticarcillin	0.208	3	0.616	---	---	33
370	oxytetracycline	0	3	0.615	---	---	0
371	dimethyloxalylglycine	0	1	-0.615	---	---	0
372	mimosine	0	3	-0.613	---	---	0
373	ifenprodil	0	4	0.612	---	---	0
374	scriptaid	0.11	3	0.612	---	---	33
375	metergoline	0.091	4	0.609	---	---	25
376	indoprofen	-0.104	4	-0.607	---	---	25
377	carbimazole	0	3	-0.607	---	---	0
378	F0447-0125	0.078	4	0.606	---	---	25
379	iopamidol	-0.164	4	-0.606	---	---	25
380	droperidol	0.109	4	0.605	---	---	25
381	Gly-His-Lys	0	3	-0.605	---	---	0
382	halofantrine	0	3	-0.603	---	---	0
383	betaxolol	0.207	4	0.602	---	---	25
384	5286656	0	1	0.599	---	---	0
385	phthalylsulfathiazole	0.082	5	0.599	---	---	20
386	piperlongumine	0	2	0.599	---	---	0
387	xylazine	0.116	4	0.595	---	---	25
388	isometheptene	0	4	-0.592	---	---	0
389	8-azaguanine	0	4	0.591	---	---	0
390	selegiline	0	4	0.588	---	---	0
391	medrysone	0.071	6	0.585	---	---	16
392	AH-6809	0	2	-0.585	---	---	0
393	thiamazole	-0.06	6	-0.584	---	---	16
394	cefalonium	0.137	3	0.583	---	---	33
395	fenoterol	0	3	0.582	---	---	0
396	exemestane	0	1	0.582	---	---	0
397	corbadrine	0	4	0.58	---	---	0
398	furosemide	-0.091	4	-0.579	---	---	25
399	valdecoxib	0	3	-0.578	---	---	0
400	artemisinin	0	3	0.578	---	---	0
401	strophanthidin	-0.128	4	-0.577	---	---	25
402	DL-thiorphan	0	2	0.577	---	---	0
403	azacitidine	0	3	0.576	---	---	0
404	verteporfin	0	3	0.576	---	---	0
405	homochlorcyclizine	0.157	4	0.572	---	---	25
406	ebselen	0	3	0.571	---	---	0
407	ivermectin	0.133	5	0.568	---	---	20
408	spiperone	0	2	0.565	---	---	0
409	CP-645525-01	0	3	0.564	---	---	0
410	copper sulfate	0	4	-0.562	---	---	0
411	5152487	0	1	0.561	---	---	0
412	cloxacillin	-0.132	4	-0.56	---	---	25
413	tracazolate	0.134	4	0.56	---	---	25
414	iloprost	-0.19	3	-0.559	---	---	33
415	MG-262	-0.278	3	-0.558	---	---	33
416	chlorambucil	0.127	4	0.558	---	---	25
417	chlorphenesin	-0.089	4	-0.558	---	---	25

418	6-azathymine	0	4	0.557	---	---	0
419	mebendazole	0	5	0.556	---	---	0
420	cycloserine	0.014	4	0.556	---	---	25
421	bethanechol	-0.15	4	-0.55	---	---	25
422	15(S)-15-methylprostaglandin E2	-0.115	4	-0.549	---	---	25
423	demecarium bromide	0	4	-0.549	---	---	0
424	dipyridamole	0.071	6	0.547	---	---	16
425	3-aminobenzamide	0	1	-0.546	---	---	0
426	5252917	0	2	-0.543	---	---	0
427	rottlerin	0	3	0.543	---	---	0
428	clopamide	0	4	-0.541	---	---	0
429	benzocaine	-0.041	4	-0.539	---	---	25
430	cloperastine	0.076	6	0.539	---	---	16
431	amoxicillin	0.056	4	0.538	---	---	25
432	coralyne	0	4	0.537	---	---	0
433	tacrolimus	-0.256	3	-0.535	---	---	33
434	citalopram	0	4	-0.534	---	---	0
435	thiocolchicoside	0	4	0.533	---	---	0
436	sitosterol	0	4	0.531	---	---	0
437	Y-27632	0	2	-0.531	---	---	0
438	oxybuprocaine	0.096	4	0.531	---	---	25
439	lisinopril	0	3	0.53	---	---	0
440	chlorcyclizine	0.151	6	0.53	---	---	33
441	niclosamide	0.1	5	0.53	---	---	20
442	nilutamide	0	4	-0.529	---	---	0
443	syrosingopine	-0.012	4	0.529	---	---	25
444	nortriptyline	0.176	4	0.529	---	---	25
445	nisoxetine	-0.155	4	-0.528	---	---	25
446	ikarugamycin	0.194	3	0.527	---	---	33
447	salsolinol	-0.12	3	-0.527	---	---	33
448	N-acetyl-L-aspartic acid	-0.093	4	0.526	---	---	0
449	oxetacaine	0	5	0.525	---	---	0
450	GW-8510	0	4	0.525	---	---	0
451	hydrochlorothiazide	0	5	-0.524	---	---	0
452	bufexamac	0	4	0.522	---	---	0
453	phenazopyridine	0.161	4	0.521	---	---	25
454	flupentixol	0.155	4	0.52	---	---	25
455	josamycin	-0.053	5	-0.52	---	---	40
456	isoetarine	0	4	0.52	---	---	0
457	N-phenylanthranilic acid	0	1	0.519	---	---	0
458	vinblastine	0	3	-0.518	---	---	0
459	carcinine	0	4	0.518	---	---	0
460	protoveratrine A	0.08	4	0.517	---	---	25
461	bisacodyl	0	4	0.514	---	---	0
462	rifabutin	0	3	0.514	---	---	0
463	sulfinpyrazone	-0.013	4	0.512	---	---	25
464	anisomycin	-0.148	4	-0.512	---	---	25
465	flunarizine	0	4	0.511	---	---	0
466	propranolol	-0.103	4	-0.511	---	---	25
467	terbutaline	0.085	4	0.511	---	---	25
468	ellipticine	0	4	0.51	---	---	0
469	flavoxate	0.133	4	0.51	---	---	25
470	bendroflumethiazide	0.012	6	0.509	---	---	33
471	roxithromycin	0	4	0.509	---	---	0
472	telenzepine	0	4	-0.507	---	---	0
473	nicergoline	0	5	0.506	---	---	0
474	oxprenolol	0	4	0.505	---	---	0
475	protriptyline	0.026	4	0.505	---	---	25
476	ronidazole	0	3	0.505	---	---	0
477	etilefrine	-0.136	4	-0.504	---	---	25

478	piretanide	-0.139	4	-0.504	---	---	25
479	naphazoline	-0.208	5	-0.503	---	---	40
480	Prestwick-1080	0	4	-0.503	---	---	0
481	PF-00539745-00	0	3	-0.503	---	---	0
482	cefoxitin	0	4	-0.502	---	---	0
483	gliclazide	0	4	0.502	---	---	0
484	rescinnamine	0	3	0.501	---	---	0
485	orlistat	-0.218	5	-0.501	---	---	40
486	LM-1685	0.198	3	0.5	---	---	33
487	latamoxef	0.119	3	0.5	---	---	33
488	fenbendazole	-0.106	4	-0.498	---	---	25
489	skimmianine	0	4	0.497	---	---	0
490	pyridoxine	0.131	4	0.497	---	---	25
491	PHA-00767505E	0	4	-0.496	---	---	0
492	bromperidol	0	3	0.495	---	---	0
493	suxibuzone	-0.194	4	0.495	---	---	0
494	hexestrol	0	4	0.495	---	---	0
495	esculetin	0	3	-0.495	---	---	0
496	podophyllotoxin	0	4	-0.493	---	---	0
497	levamisole	0	4	0.492	---	---	0
498	loracarbef	-0.143	4	-0.492	---	---	25
499	acepromazine	0	4	0.491	---	---	0
500	rimexolone	0	4	0.49	---	---	0
501	neostigmine bromide	0	4	-0.49	---	---	0
502	zardaverine	-0.127	4	-0.489	---	---	25
503	vitexin	-0.129	4	-0.489	---	---	25
504	etofylline	0	5	-0.487	---	---	0
505	meglumine	-0.128	4	-0.486	---	---	25
506	naproxen	-0.117	9	-0.485	---	---	33
507	moxonidine	-0.266	3	-0.483	---	---	33
508	(+)-isoprenaline	-0.153	4	-0.483	---	---	25
509	ofloxacin	0.204	5	0.48	---	---	40
510	Prestwick-860	0	4	0.48	---	---	0
511	moroxydine	-0.236	5	-0.479	---	---	40
512	ifosfamide	-0.188	3	-0.478	---	---	33
513	fluspirilene	0	4	0.478	---	---	0
514	bephenium hydroxynaphthoate	0.094	5	0.477	---	---	20
515	cefotetan	0.158	3	0.477	---	---	33
516	betamethasone	0.089	3	0.476	---	---	33
517	cefotiam	0.118	4	-0.473	---	---	0
518	nipecotic acid	0	4	-0.473	---	---	0
519	myosmine	-0.082	6	-0.473	---	---	33
520	triamterene	-0.23	5	-0.473	---	---	40
521	nadide	-0.154	4	-0.472	---	---	25
522	progesterone	0	4	0.472	---	---	0
523	ceforanide	0.007	4	-0.471	---	---	25
524	ipratropium bromide	0	3	0.471	---	---	0
525	clomifene	0	4	0.47	---	---	0
526	spiradoline	-0.109	4	-0.47	---	---	25
527	mexiletine	-0.153	6	-0.47	---	---	33
528	scopoletin	0	2	0.47	---	---	0
529	nadolol	0.108	4	-0.47	---	---	0
530	etacrynic acid	0	3	-0.469	---	---	0
531	PHA-00816795	0	2	0.468	---	---	0
532	pentetrazol	-0.155	4	-0.468	---	---	25
533	cyclizine	0.138	4	0.467	---	---	25
534	procyclidine	-0.12	4	-0.466	---	---	25
535	torasemide	0	4	0.466	---	---	0
536	hesperetin	0.077	5	0.465	---	---	20
537	guanadrel	0.074	5	-0.465	---	---	0

538	ceftazidime	0.154	3	0.465	---	---	33
539	iohexol	0	4	-0.464	---	---	0
540	ketanserin	-0.138	4	-0.463	---	---	25
541	tolfenamic acid	0.132	4	0.463	---	---	25
542	pindolol	-0.151	5	-0.462	---	---	40
543	probenecid	0	4	-0.462	---	---	0
544	adenosine phosphate	0	4	-0.459	---	---	0
545	tetryzoline	0	3	0.459	---	---	0
546	clemizole	0.205	5	0.459	---	---	40
547	dehydrocholic acid	-0.162	5	-0.457	---	---	20
548	beclometasone	-0.145	3	0.457	---	---	0
549	aminohippuric acid	0	4	-0.457	---	---	0
550	caffeic acid	-0.11	3	0.456	---	---	33
551	amphotericin B	0.153	4	0.456	---	---	25
552	eucatropine	0	6	-0.455	---	---	0
553	Prestwick-981	0	3	-0.454	---	---	0
554	vincamine	0.004	6	-0.454	---	---	16
555	isoniazid	-0.145	5	-0.454	---	---	20
556	sulfachlorpyridazine	-0.189	5	-0.453	---	---	40
557	idoxuridine	-0.075	5	-0.453	---	---	20
558	SB-202190	0	5	-0.452	---	---	0
559	bupivacaine	0.157	4	0.451	---	---	25
560	clomipramine	0.148	4	0.451	---	---	25
561	zaprinast	-0.118	4	-0.451	---	---	25
562	corticosterone	0	4	0.45	---	---	0
563	alfadolone	0	3	-0.45	---	---	0
564	ajmaline	-0.068	3	-0.449	---	---	33
565	carbachol	0	4	0.448	---	---	0
566	triflusal	0	3	0.448	---	---	0
567	trimethobenzamide	-0.013	5	-0.448	---	---	20
568	fludroxycortide	-0.163	5	-0.448	---	---	40
569	Prestwick-1083	0.143	3	0.447	---	---	33
570	propafenone	0	4	0.447	---	---	0
571	glycopyrronium bromide	-0.189	5	-0.446	---	---	40
572	buflomedil	0	4	0.446	---	---	0
573	aceclofenac	0.049	4	-0.446	---	---	25
574	cefsulodin	-0.135	4	-0.445	---	---	25
575	procaine	0.084	5	0.444	---	---	20
576	methacholine chloride	0	3	0.443	---	---	33
577	alsterpaullone	0	3	0.442	---	---	0
578	etanidazole	0.071	4	0.441	---	---	25
579	trioxysalen	0	4	0.44	---	---	0
580	0297417-0002B	-0.186	3	0.44	---	---	0
581	SC-58125	0.114	4	0.439	---	---	25
582	dinoprostone	-0.125	4	-0.438	---	---	25
583	iodixanol	0	3	-0.438	---	---	0
584	clofazimine	-0.13	5	-0.438	---	---	20
585	Chicago Sky Blue 6B	0.072	4	-0.436	---	---	0
586	pronetalol	0	4	0.435	---	---	0
587	Prestwick-692	0.051	4	-0.435	---	---	25
588	doxazosin	0	4	0.435	---	---	0
589	BAS-012416453	0.163	3	0.434	---	---	33
590	guanethidine	-0.055	3	0.434	---	---	33
591	benzathine benzylpenicillin	0	4	-0.434	---	---	0
592	troleandomycin	0	4	0.434	---	---	0
593	monobenzone	0	4	0.434	---	---	0
594	mestranol	0.121	4	0.433	---	---	25
595	promazine	0.091	6	0.433	---	---	16
596	tolnaftate	-0.094	5	-0.432	---	---	20
597	cefotaxime	0	5	-0.431	---	---	0

598	sulconazole	0	4	0.43	---	---	0
599	5211181	0	2	-0.43	---	---	0
600	primaquine	0	4	0.429	---	---	0
601	C-75	0	4	0.428	---	---	0
602	diethylcarbamazine	-0.178	4	-0.428	---	---	25
603	sulfadimethoxine	0.064	5	-0.428	---	---	0
604	resveratrol	0.077	9	0.427	---	---	22
605	16,16-dimethylprostaglandin E2	0.123	3	0.427	---	---	33
606	cefazolin	-0.199	5	-0.426	---	---	40
607	butyl hydroxybenzoate	0.07	5	0.426	---	---	40
608	ambroxol	0.021	4	-0.426	---	---	25
609	picrotoxinin	0.117	4	0.426	---	---	25
610	arachidonic acid	-0.061	3	-0.426	---	---	33
611	chrysin	0	3	0.425	---	---	0
612	butirosin	0.1	4	0.425	---	---	25
613	metacycline	0	4	-0.425	---	---	0
614	mepyramine	0.156	4	0.424	---	---	25
615	dantrolene	0.16	6	0.424	---	---	33
616	lobelanidine	0.11	4	0.424	---	---	25
617	aconitine	0	4	-0.423	---	---	0
618	ampyrone	0.061	5	-0.423	---	---	20
619	colforsin	-0.136	5	-0.423	---	---	20
620	guanabenz	-0.168	5	-0.423	---	---	40
621	levopropoxyphene	0	4	-0.422	---	---	0
622	myricetin	0	4	-0.422	---	---	0
623	tiaprofenic acid	-0.108	4	-0.422	---	---	25
624	BCB000040	0	4	-0.422	---	---	0
625	galantamine	0.108	4	0.422	---	---	25
626	butacaine	-0.106	4	-0.421	---	---	25
627	enalapril	0	4	-0.421	---	---	0
628	androsterone	-0.167	4	-0.42	---	---	25
629	gabexate	0	4	-0.419	---	---	0
630	Prestwick-967	-0.169	4	-0.419	---	---	25
631	acetylsalicylsalicylic acid	0.13	4	0.419	---	---	25
632	saquinavir	0	4	-0.418	---	---	0
633	3-acetamidocoumarin	0.12	4	-0.418	---	---	0
634	aminophylline	0	4	0.418	---	---	0
635	delsoline	0.124	4	0.418	---	---	25
636	nicardipine	0.12	4	0.418	---	---	25
637	alpha-ergocryptine	-0.136	6	-0.418	---	---	33
638	flumetasone	0.113	6	0.417	---	---	33
639	estropipate	0	4	-0.417	---	---	0
640	napelline	-0.059	4	0.417	---	---	25
641	AH-23848	-0.212	3	-0.417	---	---	33
642	cyproheptadine	0	5	0.417	---	---	0
643	11-deoxy-16,16-dimethylprostaglandin E2	0.099	4	0.416	---	---	25
644	dimethadione	0	4	-0.415	---	---	0
645	butoconazole	0.168	4	0.414	---	---	25
646	pheneticillin	0.005	4	-0.414	---	---	25
647	liothyronine	0	4	0.414	---	---	0
648	mefloquine	0.096	5	0.413	---	---	40
649	ioxaglic acid	-0.171	3	0.413	---	---	0
650	CP-944629	-0.16	4	-0.413	---	---	25
651	bepridil	-0.135	4	0.413	---	---	0

652	docosahexaenoic acid ethyl ester	0	2	-0.412	---	---	0
653	moxisylyte	-0.094	5	-0.412	---	---	20
654	sertaconazole	0.111	4	0.411	---	---	25
655	Prestwick-665	-0.002	5	0.41	---	---	20
656	tiabendazole	0	4	0.41	---	---	0
657	cinchonine	0.097	4	-0.409	---	---	0
658	gallamine triethiodide	0	5	0.409	---	---	0
659	cyclopenthiazide	-0.159	4	-0.409	---	---	25
660	7-aminocephalosporanic acid	0.165	4	0.409	---	---	25
661	bretylium tosilate	0.101	4	0.409	---	---	25
662	tetraethylenepentamine	-0.176	6	-0.409	---	---	33
663	hyoscyamine	0.086	5	0.408	---	---	20
664	lynestrenol	-0.257	5	-0.408	---	---	40
665	lumicolchicine	0	3	-0.408	---	---	0
666	gossypol	0.065	6	0.407	---	---	16
667	merbromin	0.08	5	-0.407	---	---	0
668	trimetazidine	0	4	0.407	---	---	0
669	adrenosterone	0	4	0.407	---	---	0
670	zomepirac	-0.123	4	-0.406	---	---	25
671	clidinium bromide	0	4	-0.406	---	---	0
672	adiphenine	0.144	5	-0.405	---	---	0
673	prednicarbate	0	3	0.405	---	---	0
674	pentoxyverine	-0.157	4	0.405	---	---	0
675	methanthelinium bromide	-0.142	4	-0.404	---	---	25
676	meclofenamic acid	0	5	-0.404	---	---	0
677	lysergol	-0.053	4	0.402	---	---	25
678	mycophenolic acid	0.192	3	0.402	---	---	33
679	dicloxacillin	0	4	-0.401	---	---	0
680	TTNPB	0	2	0.401	---	---	0
681	pilocarpine	0.149	4	-0.401	---	---	0
682	calcium folinate	0.081	5	-0.398	---	---	0
683	cisapride	0.137	4	0.398	---	---	25
684	pyrazinamide	0	4	0.398	---	---	0
685	physostigmine	0.149	4	0.398	---	---	25
686	tetrahydroalstonine	-0.144	4	-0.397	---	---	25
687	imidurea	-0.281	3	-0.396	---	---	33
688	metrizamide	-0.165	4	-0.396	---	---	25
689	proadifen	-0.111	4	-0.396	---	---	25
690	0179445-0000	-0.135	8	-0.395	---	---	25
691	Prestwick-559	-0.215	3	0.395	---	---	0
692	diphenylpyraline	0.103	6	-0.394	---	---	0
693	terconazole	0	4	0.393	---	---	0
694	ginkgolide A	-0.091	4	0.392	---	---	0
695	nitrendipine	-0.039	5	0.392	---	---	20
696	diazoxide	-0.244	5	-0.392	---	---	40
697	diltiazem	-0.254	5	-0.391	---	---	40
698	ethambutol	0.115	5	0.391	---	---	20
699	geldanamycin	-0.318	15	-0.391	---	---	46
700	bambuterol	0.076	4	-0.39	---	---	0
701	pyrvinium	-0.247	6	-0.389	---	---	33
702	lisuride	0.103	5	-0.387	---	---	0
703	levcycloserine	-0.142	4	-0.387	---	---	25
704	propofol	0	4	0.387	---	---	0
705	rosiglitazone	-0.08	14	-0.386	---	---	14
706	theophylline	0	4	-0.386	---	---	0
707	paroxetine	-0.134	4	-0.386	---	---	25
708	cimetidine	0.083	5	0.386	---	---	40
709	acetazolamide	0	4	0.386	---	---	0

710	yohimbine	-0.072	5	-0.385	---	---	20
711	N-acetyl-L-leucine	0	4	0.385	---	---	0
712	lymecycline	-0.129	4	0.385	---	---	0
713	paromomycin	-0.108	4	-0.385	---	---	25
714	staurosporine	0	4	-0.384	---	---	0
715	zidovudine	-0.153	4	-0.384	---	---	25
716	mafenide	-0.065	5	-0.384	---	---	40
717	altizide	0	4	0.383	---	---	0
718	hexetidine	0.148	4	-0.383	---	---	0
719	sulfamonomethoxine	-0.046	4	-0.383	---	---	25
720	fluorocurarine	0	4	-0.383	---	---	0
721	ursodeoxycholic acid	0	3	0.382	---	---	0
722	flumequine	0.14	4	0.382	---	---	25
723	rotenone	-0.091	4	-0.382	---	---	25
724	griseofulvin	0.197	5	0.382	---	---	40
725	2,6-dimethylpiperidine	-0.08	5	-0.382	---	---	20
726	2-aminobenzenesulfonamide	-0.119	4	-0.381	---	---	25
727	ergocalciferol	0	4	-0.381	---	---	0
728	PNU-0293363	-0.004	3	-0.381	---	---	33
729	antazoline	-0.128	4	-0.381	---	---	25
730	hydrocotarnine	0	4	0.381	---	---	0
731	sulfaphenazole	0.162	4	0.381	---	---	25
732	nifuroxazide	0	4	0.38	---	---	0
733	sulfabenzamide	0.076	4	0.38	---	---	25
734	BCB000039	0	3	0.38	---	---	0
735	disulfiram	-0.089	5	-0.379	---	---	40
736	cinchocaine	0.119	5	0.378	---	---	20
737	oxamniquine	0	4	-0.378	---	---	0
738	hydrocortisone	-0.118	3	-0.378	---	---	33
739	streptozocin	0	4	-0.378	---	---	0
740	nalbuphine	-0.274	5	-0.378	---	---	40
741	meclofenoxate	0.004	6	0.377	---	---	16
742	CP-320650-01	0	8	-0.377	---	---	12
743	naloxone	0	6	0.377	---	---	0
744	harman	0	4	-0.376	---	---	0
745	benzonatate	0	5	0.376	---	---	0
746	aciclovir	-0.004	6	-0.375	---	---	16
747	boldine	-0.116	4	-0.375	---	---	25
748	suprofen	-0.154	4	-0.375	---	---	25
749	sulfacetamide	0	4	0.375	---	---	0
750	NS-398	0.008	3	0.374	---	---	33
751	metoclopramide	-0.142	6	-0.373	---	---	33
752	Prestwick-984	-0.119	4	-0.373	---	---	25
753	molsidomine	0.097	4	0.373	---	---	25
754	tocainide	0.146	4	-0.373	---	---	0
755	vidarabine	-0.031	4	0.373	---	---	25
756	alimemazine	0.131	4	0.373	---	---	25
757	3-nitropropionic acid	-0.141	4	-0.372	---	---	25
758	metrifonate	-0.133	5	-0.372	---	---	40
759	praziquantel	-0.122	4	-0.372	---	---	25
760	fenoprofen	-0.041	6	0.371	---	---	16
761	gentamicin	0.102	4	-0.371	---	---	0
762	natamycin	-0.19	4	-0.371	---	---	25
763	ketoconazole	-0.112	4	0.371	---	---	25
764	isoxicam	0	5	-0.37	---	---	0
765	acemetacin	0	4	-0.37	---	---	0
766	cefalexin	0	5	0.369	---	---	0
767	bergenin	0	4	-0.368	---	---	0
768	rolitetracycline	0	4	0.368	---	---	0

769	iobenguane	-0.204	4	-0.367	---	---	25
770	netilmicin	-0.148	4	-0.367	---	---	25
771	prenylamine	0	4	0.367	---	---	0
772	proguanil	0.123	3	0.367	---	---	33
773	dihydroergocristine	0.107	4	-0.367	---	---	0
774	lithocholic acid	0	6	-0.367	---	---	0
775	pizotifen	0.092	4	0.366	---	---	25
776	thioridazine	0.132	20	0.366	---	---	45
777	alpha-yohimbine	0.116	3	0.366	---	---	33
778	quercetin	-0.148	6	-0.366	---	---	33
779	ticlopidine	-0.11	5	-0.366	---	---	20
780	rifampicin	0	4	-0.365	---	---	0
781	yohimbic acid	0.138	3	-0.365	---	---	0
782	khellin	0	5	-0.364	---	---	0
783	flucytosine	-0.151	4	-0.364	---	---	25
784	ethoxyquin	-0.086	5	-0.363	---	---	20
785	fluphenazine	0.11	18	0.363	---	---	27
786	aminoglutethimide	-0.182	3	-0.363	---	---	33
787	isradipine	0.171	4	0.363	---	---	25
788	levocabastine	0.126	4	0.363	---	---	25
789	decamethonium bromide	0	4	0.362	---	---	0
790	dinoprost	0.138	4	0.362	---	---	25
791	glimepiride	0.068	4	0.362	---	---	25
792	iopromide	0	4	-0.361	---	---	0
793	apomorphine	0.005	5	0.36	---	---	20
794	phenformin	0.065	7	0.36	---	---	28
795	oxaprozin	0	6	0.359	---	---	0
796	alexidine	0	4	-0.358	---	---	0
797	cefamandole	-0.156	4	-0.357	---	---	25
798	metyrapone	-0.176	4	0.357	---	---	0
799	tranylcypromine	0.098	5	0.357	---	---	20
800	bacampicillin	-0.12	4	-0.357	---	---	25
801	diethylstilbestrol	-0.062	6	-0.357	---	---	33
802	trichostatin A	0.118	182	0.357	---	---	30
803	loperamide	0.13	6	0.357	---	---	33
804	co-dergocrine mesilate	0.128	4	-0.356	---	---	0
805	carbamazepine	0.056	8	0.356	---	---	12
806	azapropazone	0.173	3	-0.355	---	---	0
807	dequalinium chloride	-0.197	4	-0.355	---	---	25
808	Prestwick-1103	0.143	4	-0.355	---	---	0
809	propantheline bromide	0.124	4	0.354	---	---	25
810	hecogenin	0.123	4	0.354	---	---	25
811	talampicillin	-0.131	4	-0.354	---	---	25
812	serotonin	0.202	5	0.354	---	---	40
813	alprostadil	0.024	7	-0.353	---	---	14
814	propoxycaine	0.129	4	0.353	---	---	25
815	fluocinonide	0.093	5	0.352	---	---	20
816	guanfacine	0.081	5	0.352	---	---	20
817	chlorhexidine	-0.167	5	-0.351	---	---	40
818	epirizole	-0.099	5	-0.351	---	---	40
819	cefmetazole	0.099	4	0.351	---	---	25
820	roxarsone	-0.084	4	-0.351	---	---	25
821	fursultiamine	0.139	4	0.351	---	---	25
822	hydroquinine	0.113	4	0.351	---	---	25
823	levobunolol	0.023	4	-0.35	---	---	25
824	cobalt chloride	0.07	3	-0.35	---	---	33
825	vorinostat	0.041	12	0.349	---	---	16
826	dipivefrine	0	4	0.349	---	---	0
827	ribavirin	0.095	4	-0.349	---	---	0
828	diflorasone	-0.191	4	-0.349	---	---	25
829	todralazine	0.102	5	0.349	---	---	20

830	ganciclovir	0	4	0.348	---	---	0
831	clotrimazole	-0.117	5	-0.348	---	---	40
832	niflumic acid	-0.138	4	-0.347	---	---	25
833	seneciphylline	0	4	0.347	---	---	0
834	etodolac	-0.144	5	-0.347	---	---	20
835	doxepin	0.222	3	0.347	---	---	33
836	conessine	0	4	-0.347	---	---	0
837	homatropine	0	5	-0.346	---	---	0
838	methylergometrine	-0.191	4	0.346	---	---	0
839	flecainide	-0.068	6	-0.345	---	---	33
840	fendiline	-0.2	3	-0.345	---	---	33
841	oxolinic acid	-0.13	5	-0.344	---	---	40
842	minocycline	-0.267	5	-0.344	---	---	40
843	isoconazole	-0.092	5	-0.344	---	---	20
844	ciclosporin	-0.061	6	-0.344	---	---	16
845	chlorpropamide	-0.189	6	-0.343	---	---	33
846	dosulepin	0.103	4	0.343	---	---	25
847	mevalolactone	0.104	3	0.342	---	---	33
848	CP-319743	-0.081	4	-0.342	---	---	25
849	clozapine	-0.124	17	-0.342	---	---	17
850	glycocholic acid	0.17	4	-0.342	---	---	0
851	risperidone	-0.098	3	0.341	---	---	33
852	repaglinide	-0.167	4	-0.341	---	---	25
853	tolazamide	0	3	-0.341	---	---	0
854	solasodine	-0.081	6	-0.341	---	---	16
855	acenocoumarol	0.022	5	-0.34	---	---	20
856	moracizine	-0.147	4	-0.34	---	---	25
857	piribedil	-0.106	4	-0.34	---	---	25
858	glibenclamide	0	4	0.34	---	---	0
859	nabumetone	0	4	0.339	---	---	0
860	etidronic acid	-0.122	4	0.339	---	---	0
861	lobeline	0.119	4	0.339	---	---	25
862	naftopidil	-0.231	3	-0.338	---	---	33
863	Prestwick-689	-0.186	4	0.338	---	---	0
864	Prestwick-1082	0.185	3	-0.338	---	---	0
865	mecamylamine	-0.016	3	-0.337	---	---	33
866	proxymetacaine	0	4	-0.337	---	---	0
867	chlorogenic acid	0	4	-0.337	---	---	0
868	fipexide	-0.131	3	-0.336	---	---	33
869	doxylamine	-0.243	5	-0.336	---	---	40
870	trimethoprim	-0.192	5	-0.336	---	---	40
871	scopolamine N-oxide	-0.012	5	0.335	---	---	20
872	cefoperazone	0.159	3	0.335	---	---	33
873	iopanoic acid	0.115	4	0.334	---	---	25
874	drofenine	0	4	0.334	---	---	0
875	imipramine	0	4	0.334	---	---	0
876	trazodone	-0.219	3	0.334	---	---	0
877	Prestwick-685	0.086	5	0.333	---	---	20
878	dexpropranolol	-0.31	3	-0.333	---	---	33
879	minoxidil	-0.129	5	-0.332	---	---	20
880	isopropamide iodide	-0.17	4	-0.332	---	---	25
881	enilconazole	-0.021	4	-0.332	---	---	25
882	phenacetin	-0.114	4	0.331	---	---	25
883	prednisone	-0.128	5	-0.331	---	---	20
884	sulfasalazine	0	5	0.331	---	---	0
885	atropine	0	4	0.331	---	---	0
886	naringin	0.078	4	0.331	---	---	25
887	ramipril	0.118	4	0.331	---	---	25
888	trimethadione	0.118	4	-0.331	---	---	0
889	hexylcaine	0	4	-0.33	---	---	0
890	aminocaproic acid	-0.223	3	-0.33	---	---	33

891	tolmetin	-0.153	4	-0.33	---	---	25
892	trifluoperazine	0.001	16	0.329	---	---	12
893	etomidate	-0.273	3	-0.329	---	---	33
894	pyrithyldione	0.101	4	-0.329	---	---	0
895	acacetin	-0.136	6	-0.328	---	---	33
896	fenofibrate	-0.111	3	-0.328	---	---	33
897	flunixin	-0.097	5	0.327	---	---	0
898	PF-01378883-00	-0.001	4	-0.327	---	---	25
899	adipiodone	0	4	-0.327	---	---	0
900	hydrastinine	-0.175	5	-0.327	---	---	40
901	econazole	0.136	4	0.326	---	---	25
902	mefenamic acid	-0.143	5	-0.326	---	---	20
903	pheniramine	0.127	5	0.326	---	---	20
904	hydroflumethiazide	-0.125	5	-0.326	---	---	20
905	pentoxifylline	0.063	5	0.326	---	---	40
906	sulfamethoxazole	0.111	5	0.325	---	---	20
907	cinnarizine	-0.165	4	0.325	---	---	0
908	antimycin A	-0.233	5	-0.325	---	---	40
909	lincomycin	-0.084	3	-0.325	---	---	33
910	cyclopentolate	-0.075	4	0.325	---	---	25
911	cefadroxil	0	4	-0.324	---	---	0
912	benzbromarone	0	3	-0.324	---	---	0
913	finasteride	-0.014	6	-0.324	---	---	33
914	omeprazole	-0.061	4	0.323	---	---	25
915	brompheniramine	0.112	4	0.322	---	---	25
916	lorglumide	-0.024	5	0.322	---	---	20
917	citiolone	-0.026	6	-0.322	---	---	16
918	danazol	0.14	4	0.322	---	---	25
919	pyrantel	-0.191	5	-0.321	---	---	40
920	sulfamerazine	-0.122	5	-0.321	---	---	20
921	AG-028671	-0.162	3	-0.321	---	---	33
922	1,4-chrysenequinone	0	2	0.321	---	---	0
923	Prestwick-682	0.096	4	0.32	---	---	25
924	clemastine	0.212	3	0.32	---	---	33
925	chlorpromazine	-0.039	19	0.32	---	---	10
926	labetalol	-0.127	4	-0.32	---	---	25
927	atropine methonitrate	0.161	3	0.319	---	---	33
928	quipazine	0.07	4	0.318	---	---	25
929	articaine	0.126	3	0.317	---	---	33
930	sulfafurazole	-0.239	5	-0.316	---	---	40
931	metformin	-0.063	10	-0.316	---	---	20
932	lomustine	0.036	4	0.316	---	---	25
933	hydroxyachillin	-0.08	4	-0.316	---	---	25
934	calycanthine	0.035	4	0.315	---	---	25
935	benperidol	0	4	0.315	---	---	0
936	lansoprazole	0.111	4	-0.315	---	---	0
937	proscillaridin	0	3	-0.315	---	---	0
938	meprylcaine	-0.119	4	0.314	---	---	25
939	gelsemine	-0.022	4	0.314	---	---	25
940	guaifenesin	0.002	6	-0.314	---	---	33
941	mifepristone	0	4	0.313	---	---	0
942	cefaclor	0.085	4	-0.313	---	---	0
943	procainamide	0	4	0.313	---	---	0
944	arcaine	0.166	4	0.313	---	---	25
945	Prestwick-920	0.096	4	0.312	---	---	25
946	BCB000038	-0.018	4	0.312	---	---	25
947	diflunisal	0	5	-0.312	---	---	0
948	imipenem	-0.156	4	0.312	---	---	0
949	amikacin	-0.109	4	-0.311	---	---	25
950	Prestwick-664	0.006	6	0.311	---	---	16
951	ranitidine	-0.1	5	-0.311	---	---	20

952	laudanosine	0	4	0.311	---	---	0
953	monorden	-0.223	22	-0.31	---	---	40
954	benzethonium chloride	-0.196	3	-0.31	---	---	33
955	quinidine	0.096	3	0.31	---	---	33
956	pempidine	-0.241	5	-0.31	---	---	40
957	cefuroxime	0	4	-0.309	---	---	0
958	trimethylcolchicinic acid	-0.096	4	-0.309	---	---	25
959	atovaquone	-0.237	3	-0.309	---	---	33
960	fenbufen	0.169	6	0.309	---	---	33
961	troglitazone	0.134	16	0.309	---	---	43
962	amylocaine	0.105	5	0.308	---	---	20
963	letrozole	-0.067	4	-0.308	---	---	25
964	solanine	0	4	0.308	---	---	0
965	Prestwick-674	-0.172	6	-0.308	---	---	33
966	hexamethonium bromide	-0.09	5	0.308	---	---	20
967	AR-A014418	-0.199	3	-0.308	---	---	33
968	profenamine	-0.152	4	-0.308	---	---	25
969	harpagoside	-0.018	4	-0.307	---	---	25
970	nocodazole	-0.121	6	-0.307	---	---	33
971	pipemidic acid	0.107	3	0.307	---	---	33
972	3-acetylcoumarin	-0.17	5	-0.307	---	---	40
973	vinpocetine	-0.161	4	-0.307	---	---	25
974	pirenperone	0.089	5	-0.306	---	---	0
975	0175029-0000	-0.086	6	-0.306	---	---	33
976	10-methoxyharmalan	-0.152	4	-0.306	---	---	25
977	Prestwick-972	-0.159	3	-0.305	---	---	33
978	baclofen	-0.021	5	-0.305	---	---	20
979	oleandomycin	-0.124	5	-0.304	---	---	40
980	ioversol	-0.123	4	0.304	---	---	0
981	etiocholanolone	-0.027	6	-0.304	---	---	16
982	0225151-0000	0.114	3	0.304	---	---	33
983	riboflavin	-0.14	4	-0.303	---	---	25
984	etynodiol	-0.056	4	-0.303	---	---	25
985	dirithromycin	0.182	3	0.303	---	---	33
986	urapidil	0	4	0.302	---	---	0
987	raloxifene	0.121	7	0.302	---	---	42
988	hydralazine	0.066	6	0.302	---	---	16
989	IC-86621	0	4	0.301	---	---	0
990	SR-95639A	0.126	4	-0.3	---	---	0
991	chlorprothixene	0.122	4	0.299	---	---	25
992	testosterone	0	5	0.299	---	---	0
993	propylthiouracil	-0.125	4	0.299	---	---	0
994	betonicine	-0.038	6	0.299	---	---	33
995	spectinomycin	-0.139	4	-0.299	---	---	25
996	dihydroergotamine	-0.008	5	0.298	---	---	20
997	meropenem	-0.146	4	-0.298	---	---	25
998	sulindac	0.193	7	0.298	---	---	42
999	tetroquinone	0	4	0.297	---	---	0
1000	allantoin	-0.096	5	-0.297	---	---	20
1001	metixene	-0.006	4	-0.297	---	---	25
1002	pergolide	0	4	-0.297	---	---	0
1003	prednisolone	0	5	-0.296	---	---	0
1004	AG-013608	-0.011	8	0.296	---	---	12
1005	sulfaquinoxaline	0.043	3	-0.296	---	---	33
1006	sulfamethoxypyridazine	-0.026	5	0.295	---	---	20
1007	budesonide	-0.164	4	0.295	---	---	0
1008	tenoxicam	0.102	4	0.295	---	---	25
1009	betazole	-0.078	5	0.294	---	---	0
1010	streptomycin	0.103	4	-0.294	---	---	0
1011	procarbazine	0	3	0.293	---	---	0
1012	benfotiamine	0	5	0.293	---	---	0

1013	carbinoxamine	-0.15	4	-0.293	---	---	25
1014	tetracaine	0.147	3	0.293	---	---	33
1015	orphenadrine	0.103	6	0.293	---	---	16
1016	acetohexamide	0.109	4	0.292	---	---	25
1017	pyrimethamine	-0.213	5	-0.292	---	---	40
1018	monastrol	-0.059	8	-0.291	---	---	37
1019	SC-19220	0.132	4	0.29	---	---	25
1020	prochlorperazine	0.055	16	0.289	---	---	12
1021	azathioprine	0.019	7	0.289	---	---	28
1022	asiaticoside	0.119	4	-0.289	---	---	0
1023	nifedipine	-0.081	7	0.289	---	---	0
1024	dimenhydrinate	0.122	4	0.289	---	---	25
1025	amantadine	0.148	4	0.289	---	---	25
1026	pregnenolone	-0.2	4	-0.288	---	---	25
1027	alverine	-0.108	4	-0.287	---	---	25
1028	nafcillin	0.098	4	0.287	---	---	25
1029	palmatine	0	4	-0.287	---	---	0
1030	bromopride	-0.087	6	-0.287	---	---	16
1031	dapsone	0.102	5	-0.286	---	---	0
1032	mephentermine	-0.011	5	0.286	---	---	20
1033	clenbuterol	-0.013	5	-0.286	---	---	20
1034	methyldopa	0	5	-0.286	---	---	0
1035	6-bromoindirubin-3'-oxime	0.032	7	-0.286	---	---	14
1036	meticrane	-0.214	5	-0.285	---	---	40
1037	pirinixic acid	-0.03	5	0.285	---	---	20
1038	abamectin	0	4	0.285	---	---	0
1039	nitrofurantoin	0	5	-0.284	---	---	0
1040	fusidic acid	-0.129	4	-0.284	---	---	25
1041	hydrastine hydrochloride	-0.002	4	0.284	---	---	25
1042	epivincamine	-0.063	4	-0.284	---	---	25
1043	octopamine	0	4	0.284	---	---	0
1044	nalidixic acid	0	5	-0.284	---	---	0
1045	fluorometholone	-0.15	4	0.283	---	---	0
1046	sotalol	0	4	0.283	---	---	0
1047	0316684-0000	0.112	4	-0.283	---	---	0
1048	carbenoxolone	0.133	4	0.283	---	---	25
1049	zalcitabine	-0.168	4	-0.283	---	---	25
1050	fluvastatin	-0.151	4	0.282	---	---	0
1051	amitriptyline	0.077	6	-0.282	---	---	0
1052	benzydamine	0	4	-0.282	---	---	0
1053	fenspiride	0.055	5	-0.282	---	---	20
1054	bromocriptine	-0.005	5	0.281	---	---	20
1055	captopril	-0.006	5	0.281	---	---	20
1056	fludrocortisone	0.107	8	0.28	---	---	37
1057	carbarsone	-0.116	4	-0.28	---	---	25
1058	probucol	-0.221	6	-0.28	---	---	33
1059	atropine oxide	0.119	5	0.279	---	---	20
1060	benserazide	0	5	-0.279	---	---	0
1061	eticlopride	0.128	4	0.279	---	---	25
1062	cytisine	0	4	0.278	---	---	0
1063	vancomycin	0.141	4	0.278	---	---	25
1064	triamcinolone	-0.16	5	-0.278	---	---	40
1065	amrinone	-0.036	4	-0.277	---	---	25
1066	clonidine	-0.025	4	0.277	---	---	25
1067	memantine	0.144	4	0.277	---	---	25
1068	Prestwick-1084	-0.139	4	0.277	---	---	0
1069	dexpanthenol	-0.192	4	-0.277	---	---	25
1070	luteolin	-0.099	4	0.276	---	---	0
1071	thioperamide	-0.024	5	-0.276	---	---	20
1072	scoulerine	0.019	4	-0.276	---	---	25

1073	bicuculline	0.144	4	0.275	---	---	25
1074	methoxamine	0.103	4	-0.275	---	---	0
1075	carteolol	-0.003	4	-0.275	---	---	25
1076	crotamiton	-0.121	4	-0.275	---	---	25
1077	CAY-10397	-0.03	3	-0.274	---	---	33
1078	amiprilose	-0.037	4	0.274	---	---	25
1079	thalidomide	0.042	7	0.274	---	---	14
1080	santonin	-0.091	4	-0.274	---	---	25
1081	doxycycline	0.082	5	0.274	---	---	20
1082	PF-00875133-00	0.118	3	0.274	---	---	33
1083	tolazoline	-0.23	5	-0.273	---	---	40
1084	ketoprofen	0.022	6	0.273	---	---	33
1085	levodopa	-0.109	5	-0.273	---	---	40
1086	4-hydroxyphenazone	0.093	5	0.273	---	---	40
1087	riluzole	0	5	-0.273	---	---	0
1088	vinburnine	0.1	4	0.272	---	---	25
1089	tiletamine	0.117	4	-0.272	---	---	0
1090	triprolidine	-0.066	4	0.272	---	---	25
1091	meptazinol	-0.143	4	0.271	---	---	0
1092	0173570-0000	-0.006	6	0.271	---	---	33
1093	chloropyramine	-0.081	4	-0.271	---	---	25
1094	propidium iodide	-0.03	4	0.271	---	---	25
1095	corynanthine	0	3	-0.271	---	---	0
1096	nicotinic acid	0.076	4	0.27	---	---	25
1097	(-)-MK-801	0.11	4	0.27	---	---	25
1098	tinidazole	0.029	6	-0.27	---	---	16
1099	stachydrine	0.133	4	0.27	---	---	25
1100	SB-203580	0.116	5	0.269	---	---	20
1101	pivampicillin	-0.154	4	0.268	---	---	0
1102	thiamine	0.17	3	0.268	---	---	33
1103	aminophenazone	0.081	5	0.267	---	---	20
1104	celecoxib	-0.133	5	-0.267	---	---	40
1105	cotinine	-0.166	6	-0.267	---	---	33
1106	amiloride	0.116	5	0.267	---	---	40
1107	PF-00562151-00	-0.005	8	-0.266	---	---	25
1108	levothyroxine sodium	0.11	4	0.265	---	---	25
1109	sulfaguanidine	-0.021	5	0.264	---	---	20
1110	etamsylate	-0.156	4	-0.264	---	---	25
1111	lovastatin	0.134	4	0.264	---	---	25
1112	pentolonium	-0.097	5	-0.262	---	---	20
1113	norfloxacin	-0.088	5	-0.262	---	---	20
1114	bumetanide	0.007	4	-0.262	---	---	25
1115	epitiostanol	-0.068	4	0.262	---	---	25
1116	heptaminol	0.175	5	0.262	---	---	40
1117	pentamidine	-0.188	5	-0.262	---	---	40
1118	felodipine	-0.164	7	-0.261	---	---	28
1119	phenylpropanolamine	-0.131	4	-0.261	---	---	25
1120	phentolamine	-0.002	7	-0.261	---	---	14
1121	noscapine	0	4	0.26	---	---	0
1122	morantel	-0.069	5	0.26	---	---	20
1123	anabasine	-0.199	3	-0.259	---	---	33
1124	Prestwick-1085	0	4	0.259	---	---	0
1125	betahistine	-0.174	4	-0.259	---	---	25
1126	sulfadiazine	-0.051	5	0.259	---	---	20
1127	midodrine	0.013	5	-0.259	---	---	20
1128	gramine	-0.205	4	-0.259	---	---	25
1129	calcium pantothenate	-0.147	4	-0.258	---	---	25
1130	debrisoquine	-0.017	4	-0.258	---	---	25
1131	paracetamol	0	4	0.258	---	---	0
1132	rilmenidine	0.119	4	-0.257	---	---	0
1133	tetracycline	0.082	5	0.257	---	---	40

1134	kinetin	0	4	-0.256	---	---	0
1135	aztreonam	-0.114	5	-0.256	---	---	20
1136	naltrexone	-0.2	5	-0.256	---	---	40
1137	ethisterone	-0.044	6	-0.256	---	---	16
1138	benzthiazide	0	4	-0.256	---	---	0
1139	tranexamic acid	0.149	5	0.255	---	---	40
1140	flurbiprofen	-0.165	5	-0.255	---	---	40
1141	N6-methyladenosine	0.002	4	-0.255	---	---	25
1142	famotidine	0.082	5	0.255	---	---	40
1143	ethotoin	-0.081	6	-0.255	---	---	16
1144	enoxacin	0	4	0.255	---	---	0
1145	flutamide	-0.059	5	-0.254	---	---	40
1146	tamoxifen	0.005	7	-0.253	---	---	14
1147	halcinonide	0.007	5	0.253	---	---	40
1148	pancuronium bromide	-0.087	4	0.253	---	---	0
1149	pramocaine	-0.082	5	-0.252	---	---	20
1150	spironolactone	-0.05	5	0.252	---	---	20
1151	wortmannin	-0.035	18	-0.252	---	---	22
1152	tyloxapol	-0.081	4	-0.252	---	---	25
1153	phenelzine	-0.069	6	-0.252	---	---	16
1154	ramifenazone	0.11	4	0.252	---	---	25
1155	sparteine	0.149	4	0.252	---	---	25
1156	mefexamide	0.139	4	0.251	---	---	25
1157	harmine	-0.137	4	0.25	---	---	0
1158	naftidrofuryl	-0.186	4	-0.25	---	---	25
1159	carisoprodol	0.204	4	0.25	---	---	25
1160	salbutamol	-0.085	5	-0.25	---	---	40
1161	indapamide	0.034	6	-0.25	---	---	16
1162	sodium phenylbutyrate	0.127	7	0.249	---	---	28
1163	lidocaine	0.119	5	0.249	---	---	20
1164	Prestwick-864	0.149	4	0.248	---	---	25
1165	tridihexethyl	0.185	4	0.248	---	---	25
1166	iocetamic acid	0.008	4	-0.248	---	---	25
1167	carmustine	0.146	3	0.247	---	---	33
1168	zimeldine	-0.018	5	0.247	---	---	20
1169	methyldopate	0.152	4	0.247	---	---	25
1170	kawain	0.087	5	0.247	---	---	20
1171	spiramycin	-0.163	6	-0.247	---	---	33
1172	Trolox C	-0.106	4	-0.247	---	---	25
1173	pirenzepine	-0.188	5	-0.246	---	---	40
1174	ornidazole	0.006	5	0.246	---	---	20
1175	succinylsulfathiazole	0	4	0.246	---	---	0
1176	ciclopirox	0.17	4	0.244	---	---	25
1177	berberine	-0.178	4	0.244	---	---	0
1178	6-benzylaminopurine	-0.071	5	-0.243	---	---	20
1179	fluvoxamine	-0.141	4	0.243	---	---	0
1180	digoxigenin	0.092	5	-0.243	---	---	0
1181	practolol	0.021	4	0.243	---	---	25
1182	PF-00539758-00	0.15	3	0.243	---	---	33
1183	oxantel	0.074	4	0.242	---	---	25
1184	dienestrol	-0.187	3	-0.242	---	---	33
1185	bemegride	0.117	4	-0.242	---	---	0
1186	ciprofloxacin	-0.073	5	-0.242	---	---	40
1187	metamizole sodium	-0.061	6	-0.241	---	---	16
1188	tretinoin	-0.01	22	0.24	---	---	9
1189	canavanine	0.025	3	-0.24	---	---	33
1190	flufenamic acid	-0.194	6	-0.238	---	---	33
1191	estradiol	-0.043	37	-0.238	---	---	21
1192	neomycin	-0.109	5	-0.238	---	---	40
1193	dexamethasone	0.076	8	0.238	---	---	25
1194	Prestwick-1100	0.089	4	0.238	---	---	25

1195	ritodrine	0	4	0.238	---	---	0
1196	harmaline	0.155	4	0.237	---	---	25
1197	noretynodrel	-0.035	4	-0.237	---	---	25
1198	apramycin	-0.185	4	0.237	---	---	0
1199	demeclocycline	-0.017	6	0.237	---	---	16
1200	ricinine	-0.19	4	-0.236	---	---	25
1201	mepenzolate bromide	-0.131	5	-0.236	---	---	40
1202	miconazole	-0.034	5	0.236	---	---	20
1203	cefapirin	0.019	4	0.235	---	---	25
1204	nefopam	-0.111	5	-0.235	---	---	40
1205	ozagrel	0	4	0.235	---	---	0
1206	simvastatin	-0.183	4	-0.235	---	---	25
1207	midecamycin	0.071	5	0.234	---	---	20
1208	dexibuprofen	0.02	4	-0.234	---	---	25
1209	cyanocobalamin	-0.091	4	-0.233	---	---	25
1210	amodiaquine	-0.034	4	0.233	---	---	25
1211	isotretinoin	0.021	4	-0.233	---	---	25
1212	ribostamycin	0	4	0.233	---	---	0
1213	sulfadimidine	0.07	6	0.233	---	---	33
1214	benzamil	-0.01	6	0.232	---	---	33
1215	clioquinol	0.082	5	0.232	---	---	20
1216	valproic acid	0.065	57	0.232	---	---	26
1217	S-propranolol	-0.13	4	-0.231	---	---	25
1218	3-hydroxy-DL-kynurenine	-0.136	6	-0.231	---	---	33
1219	lactobionic acid	0.151	4	0.23	---	---	25
1220	alpha-estradiol	-0.189	16	-0.23	---	---	37
1221	azaperone	-0.14	4	-0.23	---	---	25
1222	leflunomide	-0.028	4	-0.23	---	---	25
1223	acebutolol	0.093	5	-0.23	---	---	0
1224	eldeline	0.046	6	0.23	---	---	16
1225	fluticasone	0.113	4	0.23	---	---	25
1226	monocrotaline	-0.047	4	-0.229	---	---	25
1227	thioproperazine	0.114	5	0.229	---	---	20
1228	terguride	-0.056	8	0.229	---	---	12
1229	meclozine	-0.036	5	0.228	---	---	20
1230	H-89	-0.173	3	-0.228	---	---	33
1231	maprotiline	0.149	4	0.228	---	---	25
1232	kanamycin	0.067	4	0.227	---	---	25
1233	cefixime	0.147	4	0.226	---	---	25
1234	nifurtimox	-0.062	4	0.226	---	---	25
1235	nystatin	0.135	3	0.226	---	---	33
1236	nifenazone	-0.053	5	-0.225	---	---	20
1237	mometasone	-0.173	4	-0.225	---	---	25
1238	mephenesin	-0.135	5	-0.225	---	---	40
1239	lomefloxacin	-0.002	6	0.225	---	---	16
1240	isocarboxazid	-0.007	5	-0.224	---	---	20
1241	butamben	0.081	4	-0.224	---	---	0
1242	glipizide	0.062	5	0.224	---	---	20
1243	verapamil	-0.023	6	0.224	---	---	16
1244	dacarbazine	0	4	0.224	---	---	0
1245	chlormezanone	-0.002	4	-0.224	---	---	25
1246	azacyclonol	-0.014	5	-0.222	---	---	20
1247	nomifensine	-0.123	5	0.222	---	---	0
1248	sulfamethizole	0	4	0.222	---	---	0
1249	PHA-00851261E	0.071	8	0.221	---	---	37
1250	acetylsalicylic acid	-0.056	13	0.22	---	---	7
1251	PHA-00745360	0.119	8	-0.22	---	---	0
1252	betulinic acid	0.13	4	-0.22	---	---	0
1253	sulfadoxine	-0.171	3	-0.219	---	---	33
1254	gibberellic acid	0.104	4	0.217	---	---	25

1255	harmalol	-0.012	3	0.216	---	---	33
1256	clindamycin	-0.026	5	-0.215	---	---	20
1257	diclofenamide	0	4	0.215	---	---	0
1258	metampicillin	0.106	5	-0.215	---	---	0
1259	edrophonium chloride	-0.006	5	-0.214	---	---	20
1260	zuclopenthixol	0.139	4	0.214	---	---	25
1261	metolazone	-0.04	5	-0.213	---	---	20
1262	piromidic acid	-0.107	4	0.213	---	---	0
1263	foliosidine	0	6	-0.212	---	---	16
1264	Prestwick-642	0.132	4	-0.211	---	---	0
1265	prilocaine	-0.063	6	-0.211	---	---	33
1266	R-atenolol	-0.126	4	-0.211	---	---	25
1267	mesalazine	0.024	5	-0.209	---	---	20
1268	N-acetylmuramic acid	0.056	4	0.207	---	---	25
1269	ketotifen	0.092	4	0.207	---	---	25
1270	pralidoxime	0.122	4	0.206	---	---	25
1271	erythromycin	0.089	5	0.205	---	---	20
1272	heliotrine	-0.187	6	-0.205	---	---	33
1273	dizocilpine	0.001	5	-0.204	---	---	20
1274	nordihydroguaiaretic acid	-0.166	15	-0.204	---	---	33
1275	sulfathiazole	0.039	5	-0.203	---	---	20
1276	altretamine	-0.046	4	0.203	---	---	25
1277	tolbutamide	-0.033	7	-0.203	---	---	14
1278	diclofenac	0.077	5	0.2	---	---	20
1279	tonzonium bromide	-0.027	4	-0.199	---	---	25
1280	tremorine	-0.035	4	0.199	---	---	25
1281	paclitaxel	0.006	6	-0.199	---	---	16
1282	sulfametoxydiazine	-0.15	4	-0.197	---	---	25
1283	flucloxacillin	-0.068	4	0.196	---	---	25
1284	chlortalidone	0.081	4	0.194	---	---	25
1285	tropine	0.109	4	0.194	---	---	25
1286	deferoxamine	-0.055	8	0.193	---	---	25
1287	LY-294002	-0.117	61	-0.192	---	---	29
1288	phenindione	-0.157	4	-0.191	---	---	25
1289	sulpiride	-0.034	5	0.19	---	---	20
1290	semustine	-0.127	4	-0.19	---	---	25
1291	picotamide	0.008	5	0.189	---	---	20
1292	genistein	0.082	17	-0.189	---	---	5
1293	capsaicin	-0.013	4	0.185	---	---	25
1294	tribenoside	0.017	4	-0.181	---	---	25
1295	hydroxyzine	0.012	5	-0.181	---	---	20
1296	alfuzosin	-0.107	5	-0.18	---	---	20
1297	famprofazone	-0.087	6	-0.179	---	---	33
1298	levonorgestrel	-0.135	6	-0.178	---	---	33
1299	pioglitazone	0.038	11	-0.178	---	---	9
1300	furaltadone	-0.102	6	-0.176	---	---	33
1301	mebeverine	-0.038	4	-0.176	---	---	25
1302	pimozide	-0.148	4	0.174	---	---	0
1303	fulvestrant	-0.101	40	-0.166	---	---	25
1304	15-delta prostaglandin J2	-0.13	15	-0.164	---	---	26
1305	tropicamide	-0.084	6	0.162	---	---	16
1306	proglumide	-0.012	5	0.154	---	---	20
1307	alclometasone	-0.028	4	-0.132	---	---	25
1308	sirolimus	-0.077	44	-0.115	---	---	20
1309	haloperidol	-0.018	32	-0.113	---	---	12
