# Supplementary material for: Genomic and immunological profiles of small-cell lung cancer between East Asians and Caucasian
Source: Cancer Cell Int. 2022 Apr 29;22:173. doi: 10.1186/s12935-022-02588-w (PMC9052616; doi:10.1186/s12935-022-02588-w)
Supplement: Supplementary file 15 — Additional file 15: Table S6. Related to Additional file 5: Fig. S5aThe results of the CMap analysis of the Caucasian cohort (TP53/RB1 co-mutations vs No alterations in TP53/RB1). [file 12935_2022_2588_MOESM15_ESM.pdf]

Supplementary Table.6 Related to Supplementary Fig. 5a The results of the CMap analysis of the Caucasian cohort (TP53/RB1 co-mutations vs No alterations in TP53/RB1).

rank	cmap name	mean	n	enrichment	p	specificity	percent non-null
1	thioridazine	-0.451	20	-0.598	0	0.0645	80
2	LY-294002	-0.367	61	-0.47	0	0.0614	72
3	trichostatin A	-0.276	182	-0.268	0	0.6238	59
4	sirolimus	-0.283	44	-0.339	0.00008	0.2466	56
5	thapsigargin	0.664	3	0.951	0.0001	0.0955	100
6	alpha-estradiol	-0.43	16	-0.527	0.00012	0	87
7	oxybenzone	0.507	4	0.864	0.00046	0	100
8	semustine	0.488	4	0.854	0.00062	0.0678	100
9	tanespimycin	-0.221	62	-0.252	0.00064	0.4538	50
10	iopamidol	0.457	4	0.834	0.00115	0.0063	75
11	vincamine	0.414	6	0.705	0.00175	0	83
12	ethotoin	-0.473	6	-0.69	0.00215	0.0052	100
13	tonzonium bromide	-0.529	4	-0.807	0.00271	0.0172	100
14	lanatoside C	-0.469	6	-0.68	0.0029	0.0851	83
15	pioglitazone	0.231	11	0.502	0.00394	0.034	63
16	neostigmine bromide	0.294	4	0.783	0.00414	0.0073	50
17	trazodone	-0.574	3	-0.869	0.00443	0.0225	100
18	amoxicillin	-0.567	4	-0.781	0.00471	0.0222	100
19	melatonin	0.45	4	0.776	0.00477	0.0128	75
20	zimeldine	-0.429	5	-0.696	0.00571	0.0275	100
21	prochlorperazine	-0.328	16	-0.408	0.00648	0.1415	68
22	valproic acid	-0.207	57	-0.219	0.00733	0.2688	54
23	xylometazoline	-0.497	4	-0.752	0.00762	0.0166	100
24	cloperastine	-0.413	6	-0.628	0.0083	0.042	83
25	harmine	-0.528	4	-0.735	0.00979	0.0553	100
26	fluphenazine	-0.28	18	-0.373	0.00996	0.1429	61
27	hesperidin	0.32	4	0.729	0.01076	0.0073	50
28	DL-thiorphan	-0.633	2	-0.928	0.01093	0.0227	100
29	merbromin	0.496	5	0.661	0.01192	0.0741	80
30	piperidolate	-0.568	3	-0.817	0.01208	0.0361	100
31	valinomycin	0.196	4	0.72	0.01231	0.1512	50
32	helveticoside	-0.423	6	-0.607	0.01236	0.0779	83
33	sulfafurazole	-0.486	5	-0.652	0.01256	0.0739	100
34	florfenicol	-0.506	4	-0.717	0.01287	0.0252	100
35	digoxigenin	-0.405	5	-0.649	0.01316	0.0952	80
36	xamoterol	0.435	3	0.81	0.01392	0.0181	66
37	trimetazidine	-0.516	4	-0.705	0.01564	0.0924	100
38	cefapirin	0.236	4	0.705	0.01583	0.0154	50
39	proscillaridin	-0.643	3	-0.798	0.01695	0.0546	100
40	naringenin	0.453	4	0.698	0.01721	0.1383	75
41	altizide	0.196	4	0.697	0.01743	0	50
42	blebbistatin	-0.616	2	-0.907	0.01746	0.027	100
43	meteneprost	0.388	4	0.697	0.01757	0.087	75
44	diltiazem	-0.451	5	-0.626	0.01882	0.0729	80
45	bromperidol	-0.553	3	-0.79	0.01893	0.0121	100
46	ergocalciferol	0.321	4	0.688	0.02001	0.0184	50
47	cloxacillin	0.448	4	0.687	0.02035	0.0272	75
48	nadolol	0.488	4	0.686	0.02043	0.2418	75
49	tretinoin	-0.261	22	-0.31	0.02218	0.2206	59
50	loperamide	-0.367	6	-0.57	0.0229	0.1316	83
51	zoxazolamine	-0.487	4	-0.677	0.02407	0.0606	100
52	digitoxigenin	-0.557	4	-0.677	0.02435	0.0784	100
53	dacarbazine	-0.455	4	-0.675	0.02473	0.0259	75
54	oxymetazoline	-0.42	4	-0.673	0.02564	0.0181	100
55	galantamine	-0.464	4	-0.672	0.02598	0.0054	75
56	pilocarpine	0.3	4	0.668	0.02682	0.1132	50

57	vorinostat	-0.327	12	-0.404	0.02796	0.5575	66
58	mepacrine	-0.594	2	-0.882	0.02819	0.0583	100
59	Prestwick-559	-0.539	3	-0.758	0.02924	0.0667	100
60	digoxin	-0.521	4	-0.66	0.03018	0.1122	75
61	gliclazide	-0.437	4	-0.656	0.03223	0.125	75
62	terfenadine	-0.51	3	-0.749	0.03237	0.1376	100
63	indoprofen	0.4	4	0.656	0.03256	0.0679	75
64	solanine	-0.512	4	-0.653	0.03378	0.0656	100
65	zardaverine	0.17	4	0.651	0.03489	0.0652	75
66	mesoridazine	-0.378	4	-0.647	0.0367	0.0437	75
67	ouabain	-0.507	4	-0.645	0.03774	0.1053	75
68	promazine	-0.403	6	-0.536	0.03995	0.0634	83
69	cyproheptadine	-0.332	5	-0.577	0.04055	0.0318	80
70	ambroxol	0.433	4	0.64	0.04074	0.1438	75
71	rilmenidine	-0.153	4	-0.638	0.04098	0.0685	50
72	artemisinin	-0.454	3	-0.727	0.04194	0.0181	100
73	norcyclobenzaprine	-0.444	4	-0.635	0.04337	0.1318	75
74	bumetanide	0.402	4	0.634	0.04404	0.2012	75
75	acemetacin	0.496	4	0.634	0.04434	0.0787	75
76	metixene	-0.354	4	-0.629	0.04657	0.0923	75
77	cetirizine	-0.408	4	-0.628	0.04733	0.1691	75
78	imatinib	0.356	2	0.845	0.04829	0.0309	100
79	dilazep	-0.451	5	-0.563	0.0485	0.0966	80
80	parthenolide	-0.189	4	-0.625	0.04884	0.2621	50
81	lincomycin	0.377	3	0.708	0.04891	0.0236	66
82	danazol	0.368	4	0.626	0.04916	0.0303	75
83	F0447-0125	0.247	4	0.623	0.05047	0.1231	50
84	ketanserin	-0.418	4	-0.622	0.05081	0.0538	75
85	ramipril	0.249	4	0.621	0.05182	0.0301	50
86	ethambutol	-0.296	5	-0.558	0.05187	0.0281	80
87	piretanide	0.249	4	0.62	0.05246	0.053	75
88	hydroflumethiazide	-0.337	5	-0.555	0.05435	0.067	60
89	paromomycin	0.233	4	0.616	0.05546	0.012	50
90	folic acid	0.316	4	0.616	0.05556	0.0568	75
91	colistin	0.415	4	0.615	0.05624	0.1813	75
92	etacrynic acid	-0.412	3	-0.697	0.05671	0.024	100
93	dicoumarol	-0.293	6	-0.51	0.05769	0.0167	50
94	diethylstilbestrol	0.348	6	0.509	0.05778	0.3579	66
95	trifluoperazine	-0.271	16	-0.32	0.05798	0.3846	50
96	astemizole	-0.4	5	-0.549	0.05892	0.2574	80
97	homochlorcyclizine	-0.362	4	-0.61	0.05916	0.125	75
98	nocodazole	-0.244	6	-0.507	0.05983	0.1774	50
99	bisacodyl	-0.432	4	-0.608	0.06077	0.0887	75
100	imidurea	-0.427	3	-0.688	0.06133	0.1081	100
101	S-propranolol	-0.429	4	-0.605	0.06306	0.0366	75
102	daunorubicin	-0.427	4	-0.605	0.06338	0.2456	75
103	5194442	0.305	4	0.604	0.06378	0.0968	75
104	pramocaine	-0.32	5	-0.543	0.06416	0.0617	80
105	ondansetron	0.378	4	0.603	0.06461	0.1453	75
106	cefotiam	0.348	4	0.602	0.06541	0.1329	75
107	sulfametoxydiazine	-0.433	4	-0.597	0.06976	0.1748	75
108	colchicine	-0.355	6	-0.496	0.0698	0.0916	83
109	beta-escin	-0.357	6	-0.496	0.06996	0.1074	66
110	Prestwick-981	-0.438	3	-0.673	0.07023	0.0244	100
111	ciprofibrate	0.219	4	0.592	0.07321	0.0592	75
112	praziquantel	0.316	4	0.591	0.07376	0.0592	75
113	apigenin	-0.437	4	-0.591	0.0749	0.3261	75
114	bromocriptine	-0.388	5	-0.531	0.07516	0.1462	80
115	vitexin	-0.301	4	-0.59	0.07571	0.0405	50
116	sulfadimethoxine	0.359	5	0.532	0.07648	0.3898	60
117	amikacin	0.261	4	0.587	0.07683	0.0177	50

118	amiodarone	-0.362	5	-0.529	0.07686	0.2275	80
119	piperacetazine	-0.271	4	-0.588	0.0776	0.1168	50
120	bacampicillin	0.155	4	0.586	0.07788	0.0904	50
121	(-)-MK-801	-0.403	4	-0.586	0.07961	0.0919	75
122	gabapentin	-0.305	4	-0.586	0.07997	0.0833	75
123	trimipramine	-0.369	4	-0.585	0.08039	0.0236	75
124	5211181	0.333	2	0.798	0.08185	0.0848	50
125	isoetarine	-0.447	4	-0.581	0.08445	0.0988	75
126	chloroquine	-0.331	4	-0.579	0.08645	0.0796	75
127	bepridil	-0.399	4	-0.578	0.08749	0.2121	75
128	perhexiline	-0.422	4	-0.57	0.09501	0.2269	75
129	theobromine	0.074	4	0.567	0.09563	0.0276	50
130	5707885	0.271	4	0.565	0.09698	0.3834	50
131	nitrofural	0.273	4	0.564	0.09893	0.1111	50
132	deptropine	-0.432	4	-0.563	0.10145	0.2239	75
133	procarbazine	-0.289	3	-0.633	0.10187	0.0161	66
134	oxolamine	0.266	4	0.56	0.1036	0.2747	50
135	Prestwick-675	0.255	4	0.559	0.10414	0.1949	50
136	geldanamycin	-0.243	15	-0.302	0.10432	0.5	53
137	salbutamol	-0.429	5	-0.504	0.10498	0.0827	80
138	thiocolchicoside	-0.395	4	-0.56	0.10525	0.2012	75
139	clemastine	0.271	3	0.628	0.10682	0.228	66
140	cotinine	-0.298	6	-0.463	0.10756	0.2188	66
141	atropine methonitrate	-0.293	3	-0.627	0.1076	0.1038	66
142	triflusal	-0.432	3	-0.625	0.10953	0.3128	66
143	decamethonium bromi	0.263	4	0.553	0.10975	0.0559	50
144	gliquidone	0.217	4	0.553	0.11005	0.0633	50
145	iproniazid	0.322	5	0.502	0.11038	0.3129	60
146	aciclovir	0.263	6	0.457	0.11422	0.3789	50
147	hydrastine hydrochlori	-0.232	4	-0.552	0.11486	0.1429	50
148	proadifen	-0.362	4	-0.546	0.12206	0.1739	75
149	dextromethorphan	-0.431	4	-0.546	0.12244	0.2335	75
150	clonidine	-0.468	4	-0.545	0.1229	0.1037	75
151	diloxanide	0.244	4	0.542	0.12334	0.25	50
152	docosahexaenoic acid	0.225	2	0.75	0.12428	0.1443	50
153	prenylamine	-0.362	4	-0.541	0.12801	0.2477	75
154	ifenprodil	-0.444	4	-0.541	0.12861	0.3333	75
155	amodiaquine	-0.372	4	-0.538	0.13215	0.1513	75
156	ampyrone	0.252	5	0.485	0.13253	0.2262	60
157	nortriptyline	-0.219	4	-0.536	0.13392	0.1898	75
158	antazoline	-0.342	4	-0.535	0.13571	0.1509	75
159	alvespimycin	-0.288	12	-0.32	0.1358	0.3841	50
160	hycanthone	-0.342	4	-0.534	0.13603	0.1849	75
161	calcium folinate	0.282	5	0.482	0.13816	0.4751	60
162	imipenem	-0.395	4	-0.532	0.13895	0.3536	75
163	aztreonam	-0.349	5	-0.48	0.1397	0.0729	80
164	methylergometrine	-0.431	4	-0.53	0.14223	0.25	75
165	clomifene	-0.438	4	-0.529	0.14369	0.1957	75
166	triflupromazine	0.356	4	0.525	0.1454	0.2908	50
167	fusidic acid	0.211	4	0.525	0.14633	0.1242	50
168	verapamil	-0.216	6	-0.437	0.14791	0.1188	66
169	glycopyrronium bromid	-0.198	5	-0.475	0.14807	0.1282	80
170	pivmecillinam	0.332	4	0.523	0.14808	0.2366	50
171	proguanil	-0.318	3	-0.595	0.14849	0.1333	66
172	pargyline	-0.393	4	-0.524	0.14936	0.3568	75
173	desoxycortone	0.064	4	0.522	0.14967	0.0909	50
174	flumequine	-0.381	4	-0.523	0.15133	0.1637	75
175	Prestwick-967	0.265	4	0.52	0.15198	0.1077	50
176	prazosin	-0.256	6	-0.432	0.15631	0.121	50
177	latamoxef	-0.404	3	-0.588	0.15886	0.2012	66
178	enalapril	0.159	4	0.515	0.15956	0.1049	50

179	norethisterone	-0.393	4	-0.516	0.16089	0.2195	75
180	calycanthine	0.283	4	0.514	0.16111	0.1302	50
181	amiloride	-0.342	5	-0.465	0.16391	0.2292	80
182	propafenone	-0.391	4	-0.514	0.16394	0.2963	75
183	bufexamac	-0.384	4	-0.514	0.16408	0.3412	75
184	ceftazidime	-0.301	3	-0.583	0.16649	0.0881	66
185	0297417-0002B	-0.413	3	-0.583	0.16675	0.3361	66
186	pergolide	-0.297	4	-0.511	0.16814	0.1704	50
187	tinidazole	-0.247	6	-0.425	0.1695	0.2114	50
188	scopolamine N-oxide	-0.24	5	-0.461	0.17206	0.3175	60
189	furazolidone	0.279	4	0.506	0.17297	0.3871	50
190	thiostrepton	-0.371	4	-0.508	0.17339	0.3426	75
191	dexamethasone	0.007	8	0.366	0.18046	0.1771	50
192	chrysin	-0.381	3	-0.573	0.18218	0.4343	66
193	piperine	0.127	4	0.5	0.18311	0.0775	50
194	pentoxyverine	-0.352	4	-0.501	0.18383	0.4873	75
195	vinpocetine	-0.308	4	-0.501	0.18433	0.3122	75
196	PNU-0293363	0.274	3	0.571	0.1873	0.1698	66
197	lysergol	-0.408	4	-0.498	0.18835	0.1936	75
198	Prestwick-685	-0.319	5	-0.452	0.18977	0.3579	60
199	monobenzone	-0.366	4	-0.495	0.1933	0.2635	75
200	fasudil	0.219	2	0.683	0.2007	0.0593	50
201	etynodiol	0.267	4	0.489	0.20205	0.3416	50
202	mefexamide	-0.236	4	-0.49	0.20301	0.1879	50
203	emetine	-0.257	4	-0.489	0.20341	0.6412	75
204	trimethobenzamide	0.336	5	0.446	0.20461	0.5215	60
205	4,5-dianilinophthalimid	-0.419	2	-0.68	0.2047	0.3386	100
206	trifluridine	-0.377	4	-0.488	0.20665	0.2377	75
207	clomipramine	-0.387	4	-0.487	0.20742	0.3664	75
208	betamethasone	0.21	3	0.558	0.2101	0.1243	66
209	calmidazolium	-0.466	2	-0.675	0.2109	0.3197	100
210	zuclopenthixol	-0.291	4	-0.484	0.21421	0.1395	50
211	arachidonic acid	-0.319	3	-0.554	0.21647	0.359	66
212	seneciphylline	-0.357	4	-0.482	0.21882	0.1718	50
213	pyridoxine	-0.377	4	-0.481	0.22083	0.4335	75
214	rolitetracycline	-0.272	4	-0.481	0.22113	0.3911	50
215	dihydrostreptomycin	-0.27	5	-0.435	0.22565	0.1687	60
216	H-89	-0.241	3	-0.549	0.22666	0.2054	66
217	canadine	0.329	4	0.475	0.22666	0.6012	50
218	cyclopenthiazide	-0.237	4	-0.478	0.22696	0.2108	50
219	coralyne	0.118	4	0.475	0.22829	0.2025	50
220	copper sulfate	0.216	4	0.474	0.22972	0.2727	50
221	clenbuterol	-0.256	5	-0.432	0.23104	0.1854	60
222	nordihydroguaiaretic a	-0.138	15	-0.257	0.23452	0.2778	53
223	fluspirilene	-0.34	4	-0.469	0.24675	0.4425	75
224	pseudopelletierine	-0.367	4	-0.468	0.24898	0.3892	75
225	SC-560	0.151	3	0.538	0.24944	0.483	66
226	ribavirin	0.33	4	0.465	0.24998	0.4563	50
227	alpha-yohimbine	0.189	3	0.537	0.25118	0.3353	66
228	palmatine	-0.19	4	-0.467	0.25169	0.2737	50
229	bucladesine	0.189	6	0.386	0.25625	0.2687	50
230	bergenin	0.302	4	0.462	0.25644	0.2848	50
231	rotenone	-0.216	4	-0.464	0.25648	0.3333	50
232	thalidomide	-0.279	7	-0.36	0.25902	0.4032	57
233	iloprost	0.323	3	0.533	0.25979	0.5263	66
234	butein	0.162	2	0.639	0.25993	0.2866	50
235	terbutaline	-0.223	4	-0.462	0.26098	0.1689	50
236	etomidate	-0.371	3	-0.531	0.26122	0.5714	66
237	citalopram	0.212	4	0.459	0.26374	0.2798	50
238	santonin	-0.18	4	-0.46	0.26523	0.2434	75
239	bupivacaine	-0.226	4	-0.459	0.2679	0.2407	75

240	carteolol	0.231	4	0.457	0.26945	0.4546	50
241	3-acetamidocoumarin	0.339	4	0.456	0.27146	0.6519	50
242	SB-202190	-0.286	5	-0.415	0.27157	0.2463	60
243	cinchonidine	-0.289	4	-0.457	0.27353	0.2123	75
244	bethanechol	-0.241	4	-0.455	0.27848	0.3237	50
245	canavanine	-0.347	3	-0.523	0.2792	0.3144	66
246	epiandrosterone	-0.229	4	-0.453	0.28322	0.3881	75
247	fenoprofen	-0.332	6	-0.377	0.2842	0.648	66
248	nefopam	-0.185	5	-0.409	0.28489	0.2828	60
249	IC-86621	0.126	4	0.449	0.28668	0.3068	50
250	ethosuximide	-0.357	4	-0.451	0.28763	0.3678	75
251	picrotoxinin	-0.246	4	-0.451	0.28901	0.2707	50
252	Trolox C	-0.355	4	-0.45	0.28982	0.3069	75
253	BCB000040	0.258	4	0.447	0.29179	0.3086	50
254	suloctidil	-0.38	4	-0.449	0.29273	0.3587	75
255	propylthiouracil	-0.344	4	-0.448	0.29412	0.5677	75
256	levothyroxine sodium	0.291	4	0.446	0.29575	0.3129	50
257	chloropyramine	-0.164	4	-0.448	0.29658	0.3224	75
258	metitepine	-0.228	4	-0.447	0.2968	0.3391	50
259	chlorcyclizine	-0.229	6	-0.372	0.29749	0.1696	66
260	omeprazole	-0.327	4	-0.447	0.29762	0.6798	50
261	bambuterol	0.157	4	0.445	0.29764	0.3642	50
262	betulinic acid	0.336	4	0.444	0.29999	0.5095	50
263	tiaprofenic acid	-0.185	4	-0.446	0.30044	0.3476	50
264	phensuximide	-0.134	4	-0.444	0.30532	0.4952	75
265	letrozole	0.151	4	0.441	0.30872	0.1964	50
266	orciprenaline	-0.273	4	-0.443	0.30967	0.3286	75
267	dosulepin	-0.22	4	-0.442	0.31061	0.2479	50
268	talampicillin	-0.349	4	-0.441	0.31359	0.4706	75
269	enilconazole	-0.146	4	-0.439	0.32042	0.3292	75
270	tropine	0.185	4	0.437	0.32058	0.3188	50
271	meptazinol	-0.227	4	-0.437	0.32396	0.3568	50
272	tolfenamic acid	-0.289	4	-0.437	0.32439	0.4059	75
273	sulfapyridine	-0.2	4	-0.436	0.32674	0.2832	50
274	PF-00539758-00	-0.243	3	-0.5	0.33036	0.2519	66
275	phenelzine	-0.211	6	-0.36	0.33511	0.2444	50
276	ethisterone	0.143	6	0.358	0.33543	0.4774	50
277	puromycin	-0.327	4	-0.432	0.33707	0.597	75
278	spiramycin	0.068	6	0.357	0.33894	0.3697	50
279	yohimbic acid	-0.29	3	-0.496	0.33942	0.4824	66
280	suramin sodium	0.133	4	0.43	0.33985	0.3562	50
281	acetylsalicylic acid	-0.203	13	-0.247	0.34315	0.5775	53
282	dehydrocholic acid	-0.225	5	-0.387	0.34557	0.3044	60
283	ursolic acid	-0.118	4	-0.428	0.34779	0.4599	75
284	orlistat	-0.232	5	-0.386	0.34909	0.2874	60
285	indapamide	0.021	6	0.353	0.35001	0.2647	50
286	flucloxacillin	0.144	4	0.426	0.35039	0.3701	50
287	arachidonyltrifluoromet	0.329	2	0.58	0.35318	0.4843	50
288	0173570-0000	-0.233	6	-0.353	0.35501	0.5985	50
289	noretynodrel	-0.28	4	-0.426	0.35658	0.363	50
290	Prestwick-1103	0.31	4	0.424	0.35704	0.6522	50
291	albendazole	-0.323	3	-0.489	0.35772	0.4571	66
292	tolmetin	0.15	4	0.423	0.35883	0.25	50
293	labetalol	-0.373	4	-0.425	0.35931	0.3022	75
294	riboflavin	-0.295	4	-0.425	0.35931	0.6754	50
295	cefmetazole	0.15	4	0.423	0.35955	0.2632	50
296	pregnenolone	0.304	4	0.422	0.36096	0.4968	50
297	ketoprofen	-0.203	6	-0.351	0.36286	0.3462	50
298	metergoline	-0.365	4	-0.423	0.36301	0.3304	75
299	nialamide	-0.297	4	-0.422	0.36697	0.4577	75
300	gelsemine	0.12	4	0.42	0.36707	0.2908	50

301	pivampicillin	-0.319	4	-0.421	0.36903	0.4652	75
302	dioxybenzone	-0.334	4	-0.421	0.37043	0.4731	75
303	mifepristone	0.167	4	0.418	0.37252	0.3052	50
304	torasemide	-0.377	4	-0.419	0.37449	0.4406	75
305	molindone	0.137	4	0.417	0.37522	0.325	50
306	benzydamine	-0.261	4	-0.418	0.37713	0.3789	75
307	AR-A014418	-0.257	3	-0.481	0.37838	0.3846	66
308	nimesulide	-0.281	4	-0.416	0.38332	0.4	75
309	sulfacetamide	-0.314	4	-0.415	0.38634	0.4308	75
310	nizatidine	0.28	4	0.413	0.38728	0.3926	50
311	ionomycin	-0.371	3	-0.475	0.39264	0.4508	66
312	Prestwick-642	0.304	4	0.41	0.39565	0.6886	50
313	Prestwick-1084	-0.296	4	-0.412	0.39641	0.7954	50
314	butoconazole	-0.15	4	-0.411	0.39909	0.4351	50
315	pepstatin	0.151	4	0.409	0.39993	0.4167	50
316	clorsulon	0.288	4	0.408	0.40128	0.6725	50
317	guanfacine	-0.287	5	-0.369	0.40284	0.5746	60
318	piroxicam	-0.308	4	-0.408	0.40689	0.4971	75
319	corticosterone	0.222	4	0.405	0.41137	0.5567	50
320	fenoterol	-0.391	3	-0.467	0.41419	0.4641	66
321	propoxycaine	0.1	4	0.402	0.42368	0.3684	50
322	irinotecan	-0.327	3	-0.463	0.42398	0.6739	66
323	adenosine phosphate	-0.178	4	-0.403	0.42547	0.3907	50
324	gemfibrozil	-0.215	5	-0.362	0.42583	0.5316	60
325	propranolol	0.028	4	0.401	0.42732	0.3712	50
326	benperidol	-0.215	4	-0.401	0.43094	0.3988	75
327	niridazole	0.041	4	0.398	0.43576	0.3876	50
328	hexamethonium bromi	-0.216	5	-0.359	0.43757	0.6129	60
329	metrizamide	0.149	4	0.397	0.43828	0.6164	50
330	epitiostanol	0.057	4	0.397	0.43934	0.5067	50
331	pirlindole	-0.311	3	-0.456	0.44399	0.4422	66
332	carbarsone	0.118	4	0.396	0.44407	0.3675	50
333	sisomicin	-0.219	4	-0.397	0.44513	0.3961	50
334	halcinonide	-0.208	5	-0.357	0.44516	0.481	60
335	protriptyline	-0.305	4	-0.396	0.44779	0.5338	50
336	strophanthidin	-0.089	4	-0.396	0.44815	0.3933	50
337	Prestwick-920	-0.227	4	-0.395	0.4493	0.4124	50
338	aconitine	0.164	4	0.394	0.44936	0.3987	50
339	procyclidine	-0.06	4	-0.395	0.45042	0.431	50
340	xylazine	-0.307	4	-0.395	0.45147	0.5	50
341	naringin	-0.32	4	-0.394	0.45304	0.5388	50
342	cyclopentolate	-0.141	4	-0.394	0.4535	0.4563	50
343	0316684-0000	0.027	4	0.392	0.45499	0.3718	50
344	bicuculline	-0.275	4	-0.393	0.45865	0.4859	50
345	selegiline	-0.301	4	-0.392	0.45921	0.4268	50
346	fluvoxamine	-0.252	4	-0.392	0.4613	0.5146	50
347	phenindione	-0.188	4	-0.392	0.4613	0.573	50
348	etilefrine	0.048	4	0.39	0.46233	0.5673	50
349	saquinavir	-0.285	4	-0.391	0.46297	0.4943	50
350	methyldopate	0.075	4	0.389	0.46414	0.6629	50
351	androsterone	-0.246	4	-0.39	0.46581	0.3205	50
352	16-phenyltetranorprost	0.007	4	0.388	0.46679	0.4653	50
353	hyoscyamine	-0.289	5	-0.349	0.47316	0.5591	60
354	cicloheximide	-0.229	4	-0.388	0.47339	0.6815	50
355	chloropyrazine	0.289	4	0.386	0.47385	0.7152	50
356	CP-645525-01	-0.296	3	-0.443	0.47674	0.6194	66
357	oxprenolol	-0.198	4	-0.387	0.47681	0.6634	50
358	glimepiride	-0.188	4	-0.387	0.47749	0.3771	50
359	ofloxacin	-0.022	5	-0.348	0.47982	0.5665	60
360	hesperetin	-0.345	5	-0.347	0.48141	0.5556	60
361	betahistine	-0.294	4	-0.385	0.48223	0.64	50

362	flupentixol	-0.265	4	-0.385	0.48386	0.576	50
363	carcinine	-0.162	4	-0.385	0.48439	0.4385	50
364	alverine	-0.245	4	-0.384	0.48593	0.457	50
365	aminophenazone	-0.275	5	-0.346	0.48603	0.4706	60
366	dipivefrine	-0.271	4	-0.384	0.48718	0.5197	50
367	iocetamic acid	0.103	4	0.381	0.49	0.5852	50
368	cefaclor	0.107	4	0.378	0.50023	0.5833	50
369	ronidazole	-0.347	3	-0.434	0.50052	0.782	66
370	spiradoline	-0.12	4	-0.379	0.50104	0.4651	50
371	pempidine	-0.281	5	-0.342	0.50159	0.5679	60
372	levodopa	-0.163	5	-0.341	0.50305	0.6061	60
373	dobutamine	-0.25	4	-0.378	0.50522	0.529	50
374	quinostatin	-0.422	2	-0.498	0.50555	0.7786	50
375	mephentermine	-0.25	5	-0.34	0.50776	0.3556	60
376	dirithromycin	-0.201	3	-0.431	0.50847	0.4693	66
377	ozagrel	-0.214	4	-0.377	0.51069	0.4913	50
378	STOCK1N-28457	-0.284	3	-0.43	0.51306	0.7005	66
379	ipratropium bromide	-0.369	3	-0.429	0.51661	0.7612	66
380	tetryzoline	-0.247	3	-0.425	0.52634	0.3137	66
381	5253409	0.064	2	0.493	0.5264	0.518	50
382	cromoglicic acid	-0.179	2	-0.493	0.52664	0.5193	50
383	ketoconazole	0.048	4	0.37	0.53029	0.4967	50
384	amoxapine	-0.2	5	-0.334	0.53129	0.5641	60
385	SC-58125	-0.166	4	-0.371	0.53146	0.5684	50
386	15(S)-15-methylprosta	0.168	4	0.368	0.53427	0.6667	50
387	cefotaxime	-0.107	5	-0.332	0.53694	0.5879	60
388	BCB000038	0.102	4	0.366	0.54342	0.4146	50
389	piribedil	-0.349	4	-0.365	0.55205	0.61	50
390	eticlopride	0.249	4	0.363	0.55434	0.8056	50
391	gabexate	0.258	4	0.363	0.55434	0.7025	50
392	monocrotaline	-0.134	4	-0.364	0.55527	0.5134	50
393	probucol	-0.178	6	-0.3	0.55606	0.4785	50
394	SR-95531	0.137	4	0.36	0.56299	0.4698	50
395	quinpirole	0.125	4	0.36	0.56444	0.7514	50
396	proxymetacaine	-0.298	4	-0.361	0.56566	0.5562	50
397	spironolactone	-0.271	5	-0.324	0.56766	0.6405	60
398	LM-1685	-0.14	3	-0.408	0.57329	0.5159	66
399	PHA-00767505E	0.069	4	0.357	0.57334	0.6129	50
400	sitosterol	-0.318	4	-0.358	0.57399	0.8163	50
401	clorgiline	-0.269	4	-0.356	0.58086	0.568	50
402	edrophonium chloride	-0.27	5	-0.32	0.58456	0.6535	60
403	ketorolac	-0.062	4	0.353	0.58752	0.6186	50
404	glafenine	-0.279	4	-0.354	0.58851	0.7602	50
405	nisoxetine	-0.17	4	-0.352	0.59573	0.6646	50
406	raubasine	-0.098	4	-0.351	0.60075	0.5321	50
407	tetramisole	-0.048	4	0.349	0.60252	0.4706	50
408	fenbendazole	-0.252	4	-0.348	0.6105	0.595	50
409	paracetamol	-0.26	4	-0.345	0.61951	0.6573	50
410	estrone	-0.043	4	-0.343	0.62866	0.6264	50
411	meprylcaine	-0.172	4	-0.34	0.6391	0.6084	50
412	cyclizine	-0.233	4	-0.338	0.64557	0.6584	50
413	sulfinpyrazone	-0.268	4	-0.338	0.64557	0.7333	50
414	fipexide	-0.273	3	-0.381	0.65085	0.7624	66
415	cefalotin	-0.263	4	-0.335	0.65396	0.6935	50
416	metaraminol	-0.13	4	-0.335	0.65551	0.6648	50
417	berberine	-0.276	4	-0.334	0.65673	0.7616	50
418	parbendazole	-0.265	4	-0.333	0.66116	0.7569	50
419	meglumine	-0.247	4	-0.333	0.66208	0.6169	50
420	cefalexin	-0.268	5	-0.3	0.66376	0.9101	60
421	flavoxate	-0.286	4	-0.332	0.66464	0.6485	50
422	pyrvinium	-0.264	6	-0.275	0.6671	0.8041	50

423	guanethidine	-0.304	3	-0.373	0.67286	0.7326	66
424	isotretinoin	-0.07	4	-0.329	0.67395	0.7143	50
425	tobramycin	-0.237	4	-0.327	0.68153	0.8371	50
426	SR-95639A	-0.199	4	-0.327	0.68301	0.6395	50
427	sulfasalazine	-0.134	5	-0.293	0.69056	0.694	60
428	Prestwick-665	-0.288	5	-0.293	0.6912	0.8201	60
429	methapyrilene	-0.258	4	-0.324	0.69182	0.6704	50
430	fluvastatin	-0.109	4	-0.324	0.69323	0.6319	50
431	quinisocaine	-0.3	4	-0.323	0.69639	0.648	50
432	nadide	-0.169	4	-0.322	0.69902	0.7093	50
433	5182598	0.08	2	0.444	0.69991	0.8039	50
434	famprofazone	-0.251	6	-0.267	0.70009	0.8391	50
435	estropipate	-0.103	4	-0.321	0.70069	0.7379	50
436	5248896	-0.308	2	-0.443	0.7015	0.8618	50
437	probenecid	-0.157	4	-0.32	0.7067	0.6482	50
438	5255229	-0.206	2	-0.441	0.70691	0.5574	50
439	lorglumide	-0.24	5	-0.289	0.70702	0.9026	60
440	alfaxalone	-0.227	3	-0.36	0.7088	0.7766	66
441	Prestwick-1080	-0.237	4	-0.319	0.70889	0.7989	50
442	isocorydine	-0.107	4	-0.315	0.72315	0.6259	50
443	ornidazole	-0.225	5	-0.285	0.72432	0.8534	60
444	dydrogesterone	-0.165	4	-0.314	0.72546	0.6203	50
445	lidoflazine	-0.057	3	-0.353	0.72783	0.6715	66
446	midecamycin	-0.302	5	-0.282	0.73524	0.7461	60
447	glibenclamide	-0.14	4	-0.31	0.736	0.8794	50
448	sulpiride	-0.038	5	-0.281	0.73864	0.7449	60
449	mimosine	0.054	3	-0.348	0.73942	0.7852	66
450	10-methoxyharmalan	-0.049	4	-0.308	0.74211	0.705	50
451	ticarcillin	-0.353	3	-0.346	0.74758	0.7329	66
452	pentetrazol	-0.215	4	-0.306	0.74975	0.7512	50
453	adrenosterone	-0.241	4	-0.305	0.75241	0.8276	50
454	papaverine	-0.175	4	-0.304	0.7548	0.7547	50
455	idazoxan	-0.257	4	-0.299	0.77177	0.8315	50
456	menadione	-0.291	2	-0.418	0.77552	0.8276	50
457	metanephrine	-0.107	5	-0.271	0.77705	0.8026	60
458	etifenin	-0.255	4	-0.297	0.77883	0.7701	50
459	eucatropine	-0.23	6	-0.246	0.78453	0.9427	50
460	crotamiton	-0.217	4	-0.293	0.79186	0.8373	50
461	piperlongumine	-0.268	2	-0.408	0.80069	0.8861	50
462	meropenem	-0.186	4	-0.29	0.80077	0.8624	50
463	2,6-dimethylpiperidine	-0.18	5	-0.263	0.80445	0.827	60
464	zalcitabine	-0.081	4	-0.287	0.80966	0.75	50
465	ciclopirox	-0.165	4	-0.286	0.81076	0.9048	50
466	spiperone	-0.282	2	-0.404	0.81087	0.8158	50
467	medrysone	-0.297	6	-0.239	0.81419	0.9568	50
468	altretamine	-0.06	4	-0.285	0.81456	0.7452	50
469	clobetasol	-0.266	3	-0.324	0.81562	0.8013	66
470	ricinine	-0.16	4	-0.284	0.81915	0.7651	50
471	progesterone	-0.259	4	-0.283	0.82015	0.8054	50
472	cinoxacin	-0.249	4	-0.283	0.82063	0.8966	50
473	5230742	-0.28	2	-0.399	0.82168	0.8689	50
474	1,4-chrysenequinone	-0.28	2	-0.398	0.82343	0.8516	50
475	scoulerine	-0.138	4	-0.281	0.82568	0.8908	50
476	niflumic acid	-0.218	4	-0.28	0.82781	0.8068	50
477	flufenamic acid	-0.235	6	-0.234	0.8305	0.9143	50
478	practolol	-0.096	4	-0.277	0.83724	0.8469	50
479	remoxipride	-0.234	4	-0.277	0.83773	0.9441	50
480	0198306-0000	-0.231	4	-0.275	0.84271	0.8921	50
481	NU-1025	0.15	2	0.39	0.84274	0.7698	50
482	Y-27632	-0.239	2	-0.388	0.84727	0.9618	50
483	chlorphenesin	-0.232	4	-0.273	0.84861	0.8981	50

484	benfluorex	-0.248	4	-0.273	0.84945	0.7929	50
485	chloramphenicol	-0.215	4	-0.271	0.8557	0.9048	50
486	hecogenin	-0.082	4	-0.27	0.8579	0.8674	50
487	syrosingopine	-0.235	4	-0.266	0.86771	0.8866	50
488	phenazopyridine	-0.283	4	-0.265	0.86972	0.9114	50
489	roxithromycin	-0.221	4	-0.261	0.88086	0.9833	50
490	telenzepine	-0.1	4	-0.26	0.88408	0.8506	50
491	acetylsalicylsalicylic a	-0.081	4	-0.258	0.88945	0.9101	50
492	clofibrate	0.017	2	0.368	0.89008	0.9152	50
493	lobelanidine	-0.227	4	-0.257	0.89061	0.9141	50
494	amantadine	-0.032	4	-0.254	0.89972	0.9085	50
495	(-)-atenolol	-0.205	4	-0.252	0.90272	0.8977	50
496	napelline	-0.193	4	-0.247	0.91738	0.9379	50
497	TTNPB	0.008	2	0.346	0.92737	0.9317	50
498	tomatidine	-0.144	4	-0.242	0.9285	0.9337	50
499	ampicillin	-0.052	4	-0.239	0.93531	0.9434	50
500	bupropion	-0.226	4	-0.234	0.94726	0.9706	50
501	delsoline	-0.24	4	-0.229	0.95528	0.9661	50
502	repaglinide	-0.229	4	-0.227	0.95854	0.9902	50
503	scopolamine	-0.223	4	-0.224	0.96348	0.9456	50
504	proxyphylline	-0.132	4	-0.214	0.97675	0.9753	50
505	Prestwick-1100	-0.2	4	-0.211	0.97955	0.9752	50
506	doxazosin	-0.209	4	-0.206	0.98355	0.9949	50
507	roxarsone	-0.222	4	-0.196	0.99079	1	50
508	tiratricol	-0.117	4	-0.183	0.99556	1	50
509	suxibuzone	-0.075	4	-0.177	0.9971	1	50
510	DL-PPMP	0.936	1	0.999	---	---	100
511	tyrphostin AG-1478	0.783	1	0.992	---	---	100
512	BW-B70C	0.732	1	0.985	---	---	100
513	12,13-EODE	0.705	1	0.98	---	---	100
514	5186324	0.652	1	0.97	---	---	100
515	5186223	0.629	1	0.96	---	---	100
516	5114445	-0.641	1	-0.959	---	---	100
517	5149715	0.62	1	0.958	---	---	100
518	demecolcine	-0.608	1	-0.938	---	---	100
519	gefitinib	-0.584	1	-0.919	---	---	100
520	sulindac sulfide	0.517	1	0.913	---	---	100
521	tomelukast	0.387	1	0.858	---	---	100
522	N-phenylanthranilic ac	0.383	1	0.856	---	---	100
523	PHA-00665752	-0.511	1	-0.852	---	---	100
524	2-deoxy-D-glucose	0.361	1	0.85	---	---	100
525	5666823	0	1	0.835	---	---	0
526	fisetin	0	1	0.833	---	---	0
527	tyrphostin AG-825	0	1	0.832	---	---	0
528	pararosaniline	-0.485	1	-0.825	---	---	100
529	reserpine	0.091	3	0.82	---	---	33
530	doxorubicin	0	3	0.82	---	---	0
531	dopamine	0	1	0.818	---	---	0
532	H-7	0	4	0.816	---	---	0
533	tioguanine	-0.478	1	-0.816	---	---	100
534	5224221	0	2	0.814	---	---	0
535	sanguinarine	0	2	0.812	---	---	0
536	cantharidin	0	1	0.805	---	---	0
537	azacitidine	0	3	0.802	---	---	0
538	mitoxantrone	0	3	0.792	---	---	0
539	GW-8510	0	4	0.79	---	---	0
540	HC toxin	-0.457	1	-0.79	---	---	100
541	exemestane	0	1	0.788	---	---	0
542	verteporfin	0	3	0.787	---	---	0
543	trichlormethiazide	0.087	4	0.781	---	---	25
544	5286656	-0.439	1	-0.775	---	---	100

545	camptothecin	0	3	0.771	---	---	0
546	alsterpaullone	0	3	0.768	---	---	0
547	piromidic acid	0.123	4	0.76	---	---	25
548	cytochalasin B	0	1	0.753	---	---	0
549	dienestrol	0.209	3	0.749	---	---	33
550	BAS-012416453	0.141	3	0.744	---	---	33
551	5109870	0	1	0.742	---	---	0
552	MS-275	0	2	0.732	---	---	0
553	aminoglutethimide	0.174	3	0.726	---	---	33
554	etamsylate	0	4	0.71	---	---	0
555	NS-398	0.187	3	0.707	---	---	33
556	ebselen	0	3	0.699	---	---	0
557	5252917	0	2	0.696	---	---	0
558	moxonidine	0	3	0.69	---	---	0
559	risperidone	0.223	3	0.688	---	---	33
560	dexverapamil	0	1	0.682	---	---	0
561	R-atenolol	0.158	4	0.678	---	---	25
562	chlorambucil	0	4	0.669	---	---	0
563	hexestrol	0	4	0.664	---	---	0
564	5162773	0	1	0.661	---	---	0
565	5213008	0	1	0.661	---	---	0
566	isopropamide iodide	0.144	4	0.659	---	---	25
567	fosfosal	0.112	4	0.657	---	---	25
568	rottlerin	0	3	0.655	---	---	0
569	dimethyloxalylglycine	-0.264	1	-0.655	---	---	100
570	HNMPA-(AM)3	-0.254	1	-0.654	---	---	100
571	3-aminobenzamide	0	1	0.652	---	---	0
572	U0125	-0.228	1	-0.651	---	---	100
573	acepromazine	0	4	0.65	---	---	0
574	depudecin	0	2	0.65	---	---	0
575	dimethadione	0.137	4	0.646	---	---	25
576	5152487	0	1	0.64	---	---	0
577	hydrocotarnine	0.107	4	0.638	---	---	25
578	oligomycin	0	1	0.637	---	---	0
579	nifuroxazide	0.084	4	0.63	---	---	25
580	doxepin	0.179	3	0.628	---	---	33
581	ticlopidine	0.105	5	0.625	---	---	20
582	pimethixene	0.187	3	0.621	---	---	33
583	STOCK1N-35215	0	3	0.617	---	---	0
584	beclometasone	0.191	3	0.613	---	---	33
585	meticrane	0	5	0.61	---	---	0
586	aminocaproic acid	0.214	3	0.608	---	---	33
587	5140203	0	1	0.604	---	---	0
588	cefuroxime	0.131	4	0.603	---	---	25
589	etidronic acid	0.159	4	0.6	---	---	25
590	oxamic acid	0	1	0.599	---	---	0
591	7-aminocephalosporar	0.097	4	0.592	---	---	25
592	lisinopril	0	3	0.582	---	---	0
593	mercaptopurine	0	2	0.58	---	---	0
594	decitabine	0	1	-0.58	---	---	0
595	thioguanosine	0	4	0.578	---	---	0
596	alcuronium chloride	0	2	0.577	---	---	0
597	minocycline	0.235	5	0.571	---	---	40
598	STOCK1N-35696	0	2	-0.57	---	---	0
599	(-)-catechin	0	1	0.57	---	---	0
600	sulfaguanidine	0.131	5	0.569	---	---	20
601	celastrol	0	1	0.569	---	---	0
602	cefoxitin	0.131	4	0.568	---	---	25
603	mebhydrolin	-0.112	4	-0.565	---	---	25
604	azaperone	0.14	4	0.564	---	---	25
605	metacycline	0	4	0.563	---	---	0

606	flutamide	0.11	5	0.562	---	---	20
607	diclofenamide	0.069	4	0.56	---	---	25
608	SB-203580	0.228	5	0.558	---	---	40
609	harmalol	-0.126	3	-0.557	---	---	33
610	lymecycline	0	4	0.557	---	---	0
611	hydralazine	0.145	6	0.556	---	---	16
612	flumetasone	0.017	6	0.554	---	---	16
613	fludroxycortide	0	5	0.552	---	---	0
614	fenbufen	0.218	6	0.552	---	---	33
615	benzonatate	0.011	5	0.55	---	---	20
616	nalidixic acid	0.16	5	0.549	---	---	40
617	etanidazole	0	4	0.548	---	---	0
618	topiramate	0	1	-0.548	---	---	0
619	moxisylyte	0.148	5	0.547	---	---	20
620	iohexol	-0.151	4	-0.546	---	---	25
621	sulfaphenazole	0.114	4	0.544	---	---	25
622	oxantel	0.047	4	0.544	---	---	25
623	1,5-isoquinolinediol	0	1	-0.543	---	---	0
624	Prestwick-972	0	3	-0.54	---	---	0
625	lumicolchicine	-0.18	3	-0.54	---	---	33
626	propantheline bromide	0	4	0.539	---	---	0
627	2-aminobenzenesulfon	-0.073	4	-0.539	---	---	25
628	viomycin	0	4	-0.539	---	---	0
629	midodrine	-0.09	5	-0.537	---	---	20
630	phenyl biguanide	0	1	-0.537	---	---	0
631	norfloxacin	-0.075	5	0.537	---	---	0
632	ethaverine	-0.174	4	0.537	---	---	0
633	cyanocobalamin	0	4	0.537	---	---	0
634	PF-00875133-00	0.184	3	0.536	---	---	33
635	skimmianine	-0.133	4	0.535	---	---	0
636	acetazolamide	0	4	0.533	---	---	0
637	cinnarizine	0.118	4	0.533	---	---	25
638	esculetin	-0.161	3	-0.532	---	---	33
639	etoposide	-0.159	4	-0.528	---	---	25
640	halofantrine	0	3	-0.527	---	---	0
641	maprotiline	-0.1	4	-0.526	---	---	25
642	methoxsalen	0	3	0.526	---	---	0
643	C-75	0	4	0.525	---	---	25
644	butirosin	0	4	0.525	---	---	0
645	tiletamine	0	4	-0.525	---	---	0
646	splitomicin	0	1	0.523	---	---	0
647	thioperamide	0	5	-0.521	---	---	0
648	chlorphenamine	0	4	0.521	---	---	0
649	propofol	-0.042	4	0.519	---	---	25
650	benserazide	-0.184	5	-0.519	---	---	40
651	tacrolimus	0.128	3	0.516	---	---	33
652	lansoprazole	0.21	4	0.515	---	---	25
653	MG-132	0	1	-0.515	---	---	0
654	ethionamide	0.187	3	0.515	---	---	33
655	phenanthridinone	0	1	-0.514	---	---	0
656	pipemidic acid	0	3	0.512	---	---	0
657	dihydroergocristine	-0.131	4	-0.512	---	---	25
658	AG-012559	-0.132	3	-0.511	---	---	33
659	biperiden	0	5	-0.511	---	---	0
660	moroxydine	0	5	0.509	---	---	0
661	pizotifen	-0.107	4	-0.509	---	---	25
662	STOCK1N-35874	0	2	-0.508	---	---	0
663	isoflupredone	0	3	-0.508	---	---	0
664	carmustine	0	3	0.507	---	---	0
665	pyrithyldione	-0.114	4	-0.506	---	---	25
666	dorzolamide	0	4	0.501	---	---	0

667	5151277	0	1	0.501	---	---	0
668	azapropazone	-0.116	3	-0.5	---	---	33
669	prilocaine	0.108	6	0.499	---	---	33
670	bisoprolol	0.143	4	0.498	---	---	25
671	trimethadione	0.146	4	0.498	---	---	25
672	netilmicin	0.192	4	0.497	---	---	25
673	sulfabenzamide	0.19	4	0.497	---	---	25
674	trihexyphenidyl	0	3	-0.497	---	---	0
675	esculin	0.138	4	0.496	---	---	25
676	exisulind	0	2	-0.496	---	---	0
677	Prestwick-1082	0.238	3	0.495	---	---	33
678	gentamicin	0.154	4	0.495	---	---	25
679	vigabatrin	-0.115	3	-0.495	---	---	33
680	moracizine	0	4	0.495	---	---	0
681	fenofibrate	0.05	3	0.494	---	---	33
682	mepyramine	0	4	0.491	---	---	0
683	cefepime	-0.145	4	-0.49	---	---	25
684	guanabenz	-0.08	5	-0.489	---	---	20
685	penbutolol	-0.168	3	-0.488	---	---	33
686	bezafibrate	0	4	0.488	---	---	0
687	mestranol	0.022	4	0.487	---	---	25
688	mephenytoin	0.091	4	0.486	---	---	25
689	Prestwick-682	0.167	4	0.486	---	---	25
690	sertaconazole	0	4	0.486	---	---	0
691	benzocaine	-0.073	4	-0.485	---	---	25
692	liothyronine	-0.112	4	0.485	---	---	0
693	oxybuprocaine	0	4	0.485	---	---	0
694	stachydrine	0	4	0.484	---	---	0
695	pyrimethamine	0.096	5	0.484	---	---	20
696	chlorprothixene	-0.021	4	0.483	---	---	25
697	pirenperone	-0.138	5	-0.482	---	---	40
698	chenodeoxycholic acic	0.164	4	0.48	---	---	25
699	articaine	0.153	3	0.48	---	---	33
700	sparteine	0.113	4	0.479	---	---	25
701	ifosfamide	-0.197	3	-0.478	---	---	33
702	capsaicin	0.086	4	0.478	---	---	25
703	rescinnamine	0	3	0.478	---	---	0
704	dicloxacillin	0	4	0.476	---	---	0
705	lisuride	0.197	5	0.476	---	---	40
706	phenazone	-0.155	3	-0.475	---	---	33
707	hexetidine	-0.114	4	-0.473	---	---	25
708	simvastatin	-0.043	4	0.473	---	---	25
709	tolazoline	0.103	5	0.47	---	---	40
710	gibberellic acid	0.073	4	0.47	---	---	25
711	isocarboxazid	0.129	5	0.468	---	---	40
712	benzylpenicillin	0.007	4	0.468	---	---	25
713	kawain	0.03	5	0.466	---	---	20
714	Prestwick-984	0.095	4	0.464	---	---	25
715	pancuronium bromide	0.008	4	0.464	---	---	25
716	sulconazole	-0.158	4	0.461	---	---	0
717	pindolol	-0.125	5	-0.46	---	---	40
718	quercetin	0.186	6	0.46	---	---	33
719	rifampicin	0.119	4	0.46	---	---	25
720	bacitracin	-0.176	3	-0.459	---	---	33
721	harpagoside	0	4	0.458	---	---	0
722	felbinac	0.163	4	0.458	---	---	25
723	Prestwick-860	-0.123	4	0.457	---	---	0
724	fendiline	0	3	0.456	---	---	0
725	monensin	0	6	-0.456	---	---	0
726	arcaine	0.148	4	0.456	---	---	25
727	nimodipine	-0.165	4	-0.456	---	---	25

728	Prestwick-692	0	4	0.455	---	---	0
729	vancomycin	0	4	-0.455	---	---	0
730	suprofen	0.126	4	0.454	---	---	25
731	prednicarbate	0	3	0.454	---	---	0
732	tetroquinone	0.149	4	0.454	---	---	25
733	(+/-)-catechin	0.162	4	0.454	---	---	25
734	tropicamide	-0.063	6	0.451	---	---	0
735	cobalt chloride	0.174	3	0.451	---	---	33
736	levobunolol	0.12	4	0.451	---	---	25
737	estriol	-0.109	4	0.45	---	---	0
738	tetrahydroalstonine	0.165	4	0.45	---	---	25
739	mevalolactone	0.203	3	0.45	---	---	33
740	16,16-dimethylprostagl	0.13	3	0.45	---	---	33
741	ivermectin	-0.021	5	0.449	---	---	20
742	brompheniramine	0	4	0.448	---	---	0
743	clidinium bromide	0.173	4	0.448	---	---	25
744	clofazimine	0	5	-0.447	---	---	0
745	vinblastine	0.165	3	0.447	---	---	33
746	butyl hydroxybenzoate	0	5	0.447	---	---	0
747	carbimazole	0.269	3	0.446	---	---	33
748	fluocinonide	-0.079	5	0.445	---	---	0
749	ceforanide	0.167	4	0.445	---	---	25
750	nicotinic acid	-0.098	4	0.445	---	---	0
751	naftopidil	-0.09	3	0.443	---	---	33
752	alfuzosin	0.068	5	0.443	---	---	20
753	Prestwick-674	0.042	6	0.443	---	---	33
754	terconazole	0	4	0.441	---	---	0
755	metamizole sodium	-0.082	6	0.441	---	---	0
756	Gly-His-Lys	0.266	3	0.441	---	---	33
757	gramine	-0.148	4	-0.44	---	---	25
758	doxycycline	-0.081	5	-0.44	---	---	20
759	dyclonine	0.078	4	0.44	---	---	25
760	guanadrel	0.121	5	0.439	---	---	20
761	AG-028671	-0.105	3	-0.438	---	---	33
762	tyloxapol	-0.135	4	0.437	---	---	0
763	betonicine	-0.056	6	-0.437	---	---	33
764	bromopride	0	6	0.437	---	---	0
765	atracurium besilate	0.257	3	0.435	---	---	33
766	benzethonium chloride	-0.146	3	-0.435	---	---	33
767	atractyloside	0.186	5	0.435	---	---	40
768	homatropine	-0.075	5	-0.435	---	---	20
769	ellipticine	-0.138	4	0.434	---	---	0
770	cyclobenzaprine	0.222	4	0.433	---	---	25
771	nystatin	-0.084	3	-0.433	---	---	33
772	spaglumic acid	0	2	0.433	---	---	0
773	noscapine	0.141	4	0.433	---	---	25
774	amphotericin B	-0.074	4	-0.432	---	---	25
775	W-13	0	2	0.431	---	---	0
776	MK-886	0	2	0.431	---	---	0
777	ramifenazone	0.126	4	0.43	---	---	25
778	convolamine	0.154	4	0.429	---	---	25
779	carbenoxolone	-0.078	4	-0.427	---	---	25
780	amrinone	0	4	0.427	---	---	0
781	quinidine	0	3	0.427	---	---	0
782	vanoxerine	0.135	4	0.427	---	---	25
783	triamterene	0	5	0.426	---	---	0
784	carbinoxamine	-0.086	4	0.426	---	---	0
785	Prestwick-1083	0	3	0.425	---	---	0
786	oxyphenbutazone	-0.126	4	-0.425	---	---	25
787	tranexamic acid	0.151	5	0.421	---	---	20
788	fluoxetine	-0.113	4	-0.421	---	---	25

789	ikarugamycin	0.17	3	0.42	---	---	33
790	dinoprost	0.174	4	0.42	---	---	25
791	streptomycin	0.118	4	0.419	---	---	25
792	bemegride	0.16	4	0.418	---	---	25
793	N-acetyl-L-aspartic aci	0.13	4	0.418	---	---	25
794	primaquine	0	4	0.418	---	---	0
795	zomepirac	-0.105	4	0.417	---	---	0
796	AH-23848	-0.14	3	-0.417	---	---	33
797	metrifonate	0	5	0.416	---	---	0
798	nitrofurantoin	0.005	5	0.415	---	---	20
799	primidone	0.095	4	0.414	---	---	25
800	isoconazole	0	5	0.414	---	---	0
801	thiamine	0	3	0.413	---	---	0
802	Prestwick-864	0	4	0.412	---	---	0
803	aminophylline	0.009	4	0.412	---	---	25
804	etiocholanolone	0.157	6	0.412	---	---	33
805	oxytetracycline	-0.167	3	0.411	---	---	0
806	amitriptyline	0.13	6	0.411	---	---	16
807	hydrocortisone	-0.24	3	-0.411	---	---	33
808	CP-319743	0.132	4	0.41	---	---	25
809	Prestwick-664	-0.152	6	0.41	---	---	0
810	triprolidine	0	4	0.41	---	---	0
811	pronetalol	-0.081	4	0.409	---	---	0
812	chlormezanone	0.121	4	0.408	---	---	25
813	fludrocortisone	0.169	8	0.408	---	---	37
814	nalbuphine	-0.174	5	-0.408	---	---	40
815	benzthiazide	0.095	4	0.407	---	---	25
816	clebopride	-0.111	4	-0.407	---	---	25
817	pyrantel	-0.2	5	-0.407	---	---	40
818	furaltadone	0.023	6	0.407	---	---	16
819	hydroquinine	-0.104	4	-0.406	---	---	25
820	racecadotril	0.106	4	0.406	---	---	25
821	bretylium tosilate	0.173	4	0.405	---	---	25
822	econazole	0.085	4	0.404	---	---	25
823	guaifenesin	-0.15	6	-0.404	---	---	33
824	sulindac	0.092	7	0.403	---	---	42
825	phthalylsulfathiazole	-0.095	5	0.402	---	---	0
826	tetracaine	0.188	3	0.401	---	---	33
827	diphenhydramine	0.124	5	0.401	---	---	20
828	pipenzolate bromide	-0.022	4	0.401	---	---	25
829	clotrimazole	0.154	5	0.4	---	---	40
830	sulfamethoxazole	-0.215	5	-0.4	---	---	40
831	MG-262	0	3	0.399	---	---	0
832	ranitidine	-0.084	5	0.399	---	---	0
833	buflomedil	0.018	4	0.398	---	---	25
834	hydroxyzine	0	5	0.397	---	---	20
835	thiethylperazine	0.146	4	0.397	---	---	25
836	tolbutamide	0.019	7	0.395	---	---	42
837	rolipram	-0.101	4	-0.394	---	---	25
838	gallamine triethiodide	-0.055	5	0.394	---	---	0
839	cortisone	-0.162	3	0.394	---	---	0
840	tiapride	0.193	5	0.392	---	---	40
841	ajmaline	0.116	3	0.392	---	---	33
842	thiamphenicol	0.251	5	0.392	---	---	40
843	loracarbef	-0.08	4	-0.391	---	---	25
844	captopril	0.117	5	0.39	---	---	40
845	streptozocin	0.212	4	0.39	---	---	25
846	cephaeline	0	5	-0.389	---	---	0
847	meclofenamic acid	-0.066	5	-0.389	---	---	20
848	methocarbamol	0.001	3	0.387	---	---	33
849	memantine	0.107	4	0.386	---	---	25

850	diclofenac	0.086	5	0.386	---	---	40
851	isosorbide	0	4	0.386	---	---	0
852	finasteride	0.16	6	0.386	---	---	33
853	pimozide	-0.093	4	0.385	---	---	0
854	cefalonium	0.057	3	0.384	---	---	33
855	PHA-00816795	0	2	-0.384	---	---	0
856	ioxaglic acid	0	3	0.384	---	---	0
857	benzathine benzylpeni	0.18	4	0.383	---	---	25
858	rifabutin	-0.163	3	-0.382	---	---	33
859	3-acetylcoumarin	0.01	5	0.382	---	---	20
860	lasalocid	0.109	4	0.382	---	---	25
861	drofenine	0	4	0.382	---	---	0
862	solasodine	-0.178	6	-0.381	---	---	33
863	clopamide	0.15	4	0.381	---	---	25
864	harmol	0.093	4	0.38	---	---	25
865	boldine	0	4	0.38	---	---	0
866	timolol	0.119	4	0.379	---	---	25
867	ciclacillin	-0.094	4	-0.378	---	---	25
868	urapidil	-0.119	4	0.377	---	---	0
869	Prestwick-691	0.14	3	0.377	---	---	33
870	iopromide	0	4	0.377	---	---	0
871	isoniazid	0.131	5	0.377	---	---	20
872	ginkgolide A	-0.123	4	0.377	---	---	0
873	chlorogenic acid	0.03	4	-0.376	---	---	25
874	scriptaid	-0.152	3	0.376	---	---	0
875	withaferin A	-0.084	4	-0.376	---	---	25
876	diphenylpyraline	-0.05	6	-0.375	---	---	16
877	karakoline	0.078	6	0.374	---	---	16
878	myricetin	0.132	4	0.374	---	---	25
879	foliosidine	0	6	0.373	---	---	0
880	prednisone	0.173	5	0.372	---	---	40
881	hymecromone	-0.095	4	-0.372	---	---	25
882	corbadrine	-0.128	4	-0.371	---	---	25
883	caffeic acid	-0.083	3	0.37	---	---	33
884	debrisoquine	0	4	0.369	---	---	0
885	isometheptene	0.142	4	0.369	---	---	25
886	theophylline	-0.138	4	-0.369	---	---	25
887	sodium phenylbutyrate	0.003	7	0.368	---	---	28
888	sulmazole	0.128	3	0.368	---	---	33
889	niclosamide	-0.225	5	-0.368	---	---	40
890	cyclic adenosine monc	0.027	4	-0.367	---	---	25
891	sulfadiazine	0.005	5	0.367	---	---	20
892	levocabastine	-0.096	4	-0.367	---	---	25
893	levopropoxyphene	0.072	4	-0.367	---	---	25
894	nifenazone	0.129	5	0.365	---	---	40
895	dl-alpha tocopherol	-0.18	4	-0.364	---	---	25
896	adiphenine	0.146	5	0.364	---	---	20
897	procaine	-0.216	5	-0.363	---	---	40
898	trioxysalen	-0.162	4	0.363	---	---	0
899	sulfadimidine	-0.17	6	-0.362	---	---	33
900	CP-690334-01	-0.016	8	-0.362	---	---	37
901	aminohippuric acid	-0.016	4	0.361	---	---	25
902	demecarium bromide	0.103	4	0.361	---	---	25
903	denatonium benzoate	-0.127	4	-0.361	---	---	25
904	atropine oxide	0.144	5	0.361	---	---	40
905	metoprolol	0.14	4	0.361	---	---	25
906	trimethoprim	-0.017	5	0.361	---	---	20
907	fluorometholone	0.01	4	0.36	---	---	25
908	vidarabine	-0.123	4	-0.36	---	---	25
909	fusaric acid	-0.174	4	-0.36	---	---	25
910	anisomycin	0	4	0.359	---	---	0

911	chlortalidone	-0.05	4	-0.359	---	---	25
912	phenylpropanolamine	-0.096	4	-0.359	---	---	25
913	dipyridamole	-0.113	6	0.358	---	---	16
914	Prestwick-857	0	4	0.358	---	---	0
915	pentoxifylline	0.108	5	0.358	---	---	20
916	atropine	-0.136	4	-0.358	---	---	25
917	(+)-chelidonine	0.162	4	0.357	---	---	25
918	methyldopa	0	5	0.357	---	---	0
919	trimethylcolchicinic aci	0.102	4	0.356	---	---	25
920	trapidil	0.087	3	0.356	---	---	33
921	cinchonine	-0.037	4	-0.356	---	---	25
922	mecamylamine	-0.144	3	-0.355	---	---	33
923	dapsone	0.195	5	0.355	---	---	40
924	nomifensine	0.035	5	0.355	---	---	20
925	naproxen	0.165	9	0.354	---	---	33
926	oxedrine	0.128	4	0.354	---	---	25
927	pentolonium	0.05	5	0.354	---	---	40
928	anabasine	-0.156	3	0.352	---	---	0
929	podophyllotoxin	0.175	4	0.351	---	---	25
930	oxybutynin	-0.004	4	0.351	---	---	25
931	canrenoic acid	-0.013	4	0.35	---	---	25
932	aceclofenac	0.008	4	-0.35	---	---	25
933	Prestwick-983	0.182	3	0.35	---	---	33
934	celecoxib	0.206	5	0.35	---	---	40
935	phenoxybenzamine	-0.126	4	0.35	---	---	0
936	piracetam	-0.16	4	-0.35	---	---	25
937	hemicholinium	-0.153	4	-0.349	---	---	25
938	benzbromarone	0.129	3	0.349	---	---	33
939	heliotrine	-0.074	6	0.348	---	---	16
940	etofenamate	-0.137	4	0.348	---	---	0
941	demeclocycline	-0.128	6	0.348	---	---	16
942	pirinixic acid	0.053	5	0.347	---	---	40
943	resveratrol	-0.107	9	0.347	---	---	0
944	naftidrofuryl	0.096	4	0.346	---	---	25
945	flurbiprofen	-0.08	5	-0.346	---	---	20
946	chlorpropamide	0.05	6	0.346	---	---	33
947	amprolium	-0.065	5	0.346	---	---	40
948	retrorsine	-0.003	4	0.344	---	---	25
949	khellin	0.071	5	0.344	---	---	40
950	dequalinium chloride	-0.15	4	0.343	---	---	0
951	heptaminol	0.213	5	0.343	---	---	40
952	equilin	0.057	5	0.342	---	---	20
953	acenocoumarol	-0.069	5	-0.342	---	---	40
954	diprophylline	-0.171	5	-0.341	---	---	40
955	oxetacaine	-0.083	5	0.341	---	---	20
956	thiamazole	-0.137	6	-0.341	---	---	33
957	naphazoline	-0.056	5	0.341	---	---	0
958	lomustine	0.074	4	0.34	---	---	25
959	amylocaine	0.026	5	0.34	---	---	20
960	amiprilose	-0.07	4	0.34	---	---	0
961	cefotetan	-0.094	3	0.34	---	---	0
962	8-azaguanine	-0.21	4	0.339	---	---	0
963	nafcillin	0.029	4	0.339	---	---	25
964	luteolin	-0.168	4	-0.339	---	---	25
965	quipazine	0.092	4	0.339	---	---	25
966	cefamandole	0.073	4	0.338	---	---	25
967	11-deoxy-16,16-dimeth	-0.023	4	0.338	---	---	25
968	tolnaftate	0.194	5	0.338	---	---	40
969	rimexolone	-0.148	4	0.338	---	---	0
970	metoclopramide	-0.062	6	0.336	---	---	33
971	famotidine	-0.028	5	0.335	---	---	20

972	glycocholic acid	0.016	4	0.335	---	---	25
973	megestrol	-0.073	4	0.334	---	---	0
974	fursultiamine	-0.074	4	0.334	---	---	0
975	tremorine	-0.009	4	0.334	---	---	25
976	rofecoxib	-0.05	6	-0.333	---	---	16
977	betulin	0.154	3	0.333	---	---	33
978	mebendazole	-0.253	5	-0.333	---	---	40
979	staurosporine	-0.063	4	0.333	---	---	25
980	PF-00539745-00	-0.023	3	0.333	---	---	33
981	alprenolol	-0.147	4	-0.331	---	---	25
982	molsidomine	-0.075	4	0.331	---	---	0
983	nipecotic acid	-0.115	4	0.331	---	---	0
984	dicycloverine	0.041	5	0.33	---	---	20
985	levomepromazine	0.085	4	0.329	---	---	25
986	quinethazone	-0.085	4	0.329	---	---	0
987	PHA-00745360	-0.042	8	-0.328	---	---	12
988	scopoletin	-0.213	2	0.328	---	---	0
989	benfotiamine	-0.222	5	-0.327	---	---	40
990	nabumetone	-0.006	4	0.327	---	---	25
991	difenidol	-0.203	3	0.327	---	---	0
992	diperodon	0.083	3	0.327	---	---	33
993	ethoxyquin	-0.194	5	-0.326	---	---	40
994	mycophenolic acid	-0.006	3	0.327	---	---	33
995	methazolamide	-0.137	4	0.326	---	---	0
996	monastrol	0.008	8	0.326	---	---	12
997	novobiocin	-0.01	9	0.326	---	---	11
998	carisoprodol	-0.016	4	0.325	---	---	25
999	tolazamide	-0.073	3	-0.325	---	---	33
1000	brinzolamide	-0.079	4	0.325	---	---	0
1001	pheneticillin	0.054	4	0.324	---	---	25
1002	BCB000039	-0.166	3	-0.324	---	---	33
1003	sulfamethoxypyridazin	-0.179	5	-0.323	---	---	40
1004	calcium pantothenate	-0.135	4	-0.322	---	---	25
1005	dropropizine	-0.07	4	0.322	---	---	25
1006	lactobionic acid	-0.081	4	0.322	---	---	0
1007	5155877	0.009	4	0.322	---	---	25
1008	harman	-0.081	4	0.321	---	---	0
1009	methylprednisolone	-0.127	4	-0.321	---	---	25
1010	baclofen	-0.098	5	-0.32	---	---	40
1011	abamectin	0.03	4	0.32	---	---	25
1012	protoveratrine A	-0.032	4	0.32	---	---	25
1013	triamcinolone	0.034	5	0.32	---	---	40
1014	fluorocurarine	-0.154	4	0.32	---	---	0
1015	alclometasone	-0.033	4	-0.319	---	---	25
1016	erastin	-0.082	4	-0.318	---	---	25
1017	etamivan	-0.028	4	0.318	---	---	25
1018	troglitazone	-0.006	16	0.318	---	---	25
1019	flunisolide	-0.16	6	0.317	---	---	0
1020	kanamycin	0.077	4	0.317	---	---	25
1021	citiolone	-0.139	6	0.317	---	---	0
1022	ciprofloxacin	0.037	5	0.317	---	---	20
1023	serotonin	-0.068	5	-0.317	---	---	40
1024	cycloserine	-0.133	4	-0.317	---	---	25
1025	cyproterone	-0.135	4	0.316	---	---	0
1026	felodipine	-0.094	7	0.316	---	---	0
1027	leflunomide	0.016	4	-0.316	---	---	25
1028	isradipine	-0.083	4	0.315	---	---	0
1029	enoxacin	-0.115	4	-0.315	---	---	25
1030	levamisole	-0.155	4	0.315	---	---	0
1031	sotalol	-0.084	4	0.315	---	---	0
1032	AH-6809	-0.167	2	0.315	---	---	0

1033	ioversol	-0.16	4	-0.314	---	---	25
1034	pyrazinamide	-0.108	4	-0.314	---	---	25
1035	betazole	-0.223	5	-0.314	---	---	40
1036	6-bromoindirubin-3'-ox	0.049	7	0.313	---	---	28
1037	milrinone	-0.224	3	-0.313	---	---	33
1038	0175029-0000	-0.153	6	0.313	---	---	0
1039	pirenzepine	-0.154	5	0.312	---	---	0
1040	sulfamonomethoxine	0.136	4	0.312	---	---	25
1041	carbachol	-0.157	4	0.312	---	---	0
1042	flecainide	0.047	6	0.312	---	---	33
1043	chlorzoxazone	-0.058	4	0.311	---	---	25
1044	betaxolol	-0.002	4	0.311	---	---	25
1045	proglumide	0.02	5	0.311	---	---	20
1046	N-acetyl-L-leucine	-0.14	4	0.311	---	---	0
1047	phenformin	0.066	7	0.311	---	---	28
1048	dinoprostone	0.057	4	0.31	---	---	25
1049	diflunisal	0.05	5	0.31	---	---	20
1050	cefsulodin	-0.035	4	0.31	---	---	25
1051	mepenzolate bromide	-0.08	5	-0.309	---	---	20
1052	valdecoxib	-0.114	3	0.309	---	---	0
1053	arecoline	-0.006	4	-0.309	---	---	25
1054	rosiglitazone	0.091	14	0.308	---	---	42
1055	metyrapone	-0.121	4	0.308	---	---	0
1056	cimetidine	0.015	5	0.308	---	---	20
1057	kaempferol	0.047	4	0.308	---	---	25
1058	levonorgestrel	-0.204	6	0.307	---	---	0
1059	metronidazole	0.147	5	0.307	---	---	40
1060	profenamine	-0.095	4	-0.307	---	---	25
1061	colforsin	-0.045	5	0.306	---	---	20
1062	6-azathymine	-0.178	4	0.306	---	---	0
1063	mometasone	0.055	4	0.306	---	---	25
1064	troleandomycin	-0.135	4	0.306	---	---	0
1065	budesonide	-0.018	4	0.305	---	---	25
1066	homosalate	-0.16	4	0.304	---	---	0
1067	tranylcypromine	-0.195	5	0.303	---	---	0
1068	PNU-0230031	0.084	8	0.303	---	---	25
1069	bendroflumethiazide	-0.116	6	0.302	---	---	0
1070	cefadroxil	-0.088	4	0.302	---	---	0
1071	cefazolin	0.032	5	0.301	---	---	20
1072	meclofenoxate	-0.177	6	-0.3	---	---	33
1073	flunarizine	-0.144	4	0.3	---	---	0
1074	doxylamine	-0.06	5	0.299	---	---	20
1075	acetohexamide	0.067	4	0.299	---	---	25
1076	josamycin	-0.05	5	-0.298	---	---	40
1077	diethylcarbamazine	0.051	4	0.298	---	---	25
1078	lithocholic acid	-0.009	6	0.298	---	---	16
1079	azacyclonol	0.065	5	0.298	---	---	40
1080	6-benzylaminopurine	-0.035	5	0.297	---	---	20
1081	idoxuridine	0.017	5	0.297	---	---	20
1082	laudanosine	-0.008	4	0.297	---	---	25
1083	buspirone	-0.049	4	0.297	---	---	25
1084	myosmine	0.079	6	0.297	---	---	33
1085	methacholine chloride	-0.201	3	0.296	---	---	0
1086	PHA-00851261E	0.027	8	0.296	---	---	25
1087	clemizole	-0.105	5	0.294	---	---	0
1088	3-hydroxy-DL-kynureni	0.078	6	0.294	---	---	16
1089	terazosin	0.088	4	0.293	---	---	25
1090	nicardipine	-0.012	4	0.293	---	---	25
1091	etofylline	-0.147	5	0.293	---	---	20
1092	atovaquone	-0.204	3	0.292	---	---	0
1093	hydrochlorothiazide	0.131	5	0.292	---	---	40

1094	ursodeoxycholic acid	-0.213	3	0.292	---	---	0
1095	sulfamethizole	-0.105	4	0.291	---	---	25
1096	clofilium tosylate	-0.213	3	-0.291	---	---	33
1097	benzamil	-0.035	6	-0.291	---	---	33
1098	pinacidil	-0.054	4	0.291	---	---	25
1099	disulfiram	0.118	5	0.29	---	---	40
1100	methoxamine	-0.011	4	-0.29	---	---	25
1101	hydrastinine	0.047	5	0.289	---	---	20
1102	lidocaine	-0.016	5	0.289	---	---	40
1103	sulfadoxine	-0.125	3	-0.288	---	---	33
1104	nicergoline	-0.228	5	-0.288	---	---	40
1105	15-delta prostaglandin	-0.085	15	-0.288	---	---	33
1106	desipramine	0.077	4	0.287	---	---	25
1107	piperacillin	-0.073	5	-0.287	---	---	40
1108	naftifine	0.06	4	0.287	---	---	25
1109	cefoperazone	-0.023	3	-0.286	---	---	33
1110	perphenazine	-0.194	5	-0.286	---	---	40
1111	adipiodone	-0.133	4	-0.286	---	---	25
1112	dimenhydrinate	0.082	4	-0.286	---	---	25
1113	procainamide	-0.161	4	0.286	---	---	0
1114	iodixanol	0.066	3	0.286	---	---	33
1115	metformin	-0.074	10	-0.286	---	---	30
1116	ganciclovir	-0.108	4	-0.285	---	---	25
1117	sulfaquinoxaline	0.062	3	0.285	---	---	33
1118	L-methionine sulfoximi	-0.103	4	0.285	---	---	25
1119	salsolinol	-0.108	3	-0.284	---	---	33
1120	cytisine	0.019	4	-0.284	---	---	25
1121	lovastatin	-0.044	4	0.284	---	---	25
1122	asiaticoside	-0.018	4	0.282	---	---	25
1123	flunixin	-0.17	5	-0.282	---	---	20
1124	eldeline	-0.124	6	0.281	---	---	0
1125	yohimbine	-0.134	5	-0.281	---	---	20
1126	naltrexone	0.107	5	0.28	---	---	40
1127	hydroxyachillin	-0.101	4	-0.28	---	---	25
1128	PNU-0251126	-0.002	6	-0.28	---	---	33
1129	PHA-00846566E	-0.128	3	0.279	---	---	0
1130	cisapride	-0.156	4	-0.278	---	---	25
1131	Prestwick-689	-0.014	4	0.278	---	---	25
1132	raloxifene	-0.125	7	-0.278	---	---	28
1133	tridihexethyl	-0.129	4	-0.278	---	---	25
1134	3-nitropropionic acid	-0.187	4	0.277	---	---	0
1135	isoxicam	0.044	5	-0.277	---	---	20
1136	colecalciferol	-0.225	4	0.276	---	---	0
1137	nomegestrol	0.049	3	0.276	---	---	33
1138	pralidoxime	0.011	4	0.274	---	---	25
1139	meclozine	-0.186	5	0.274	---	---	0
1140	clindamycin	-0.094	5	-0.274	---	---	40
1141	fluticasone	-0.095	4	-0.273	---	---	25
1142	azathioprine	-0.172	7	0.273	---	---	0
1143	mefloquine	-0.18	5	-0.273	---	---	40
1144	griseofulvin	-0.041	5	0.273	---	---	20
1145	oleandomycin	-0.091	5	-0.273	---	---	20
1146	minoxidil	0.043	5	0.273	---	---	20
1147	5279552	-0.214	2	0.272	---	---	0
1148	flucytosine	0.032	4	0.272	---	---	25
1149	epirizole	-0.088	5	-0.272	---	---	20
1150	alprostadil	0.064	7	0.271	---	---	42
1151	metampicillin	0.124	5	0.271	---	---	40
1152	0225151-0000	-0.131	3	0.271	---	---	0
1153	testosterone	0.036	5	0.271	---	---	40
1154	CP-944629	0.003	4	0.27	---	---	25

1155	biotin	-0.132	3	0.27	---	---	0
1156	pheniramine	-0.081	5	0.27	---	---	0
1157	CP-320650-01	0.066	8	0.27	---	---	25
1158	isoxsuprine	-0.2	5	-0.269	---	---	40
1159	spectinomycin	-0.009	4	0.269	---	---	25
1160	hexylcaine	-0.13	4	-0.268	---	---	25
1161	loxapine	0.038	4	0.268	---	---	25
1162	alpha-ergocryptine	-0.086	6	-0.268	---	---	33
1163	4-hydroxyphenazone	0.071	5	0.268	---	---	40
1164	gossypol	0.014	6	0.268	---	---	16
1165	co-dergocrine mesilate	-0.098	4	-0.266	---	---	25
1166	tacrine	-0.113	4	-0.266	---	---	25
1167	picotamide	-0.111	5	0.266	---	---	20
1168	azlocillin	-0.154	4	0.265	---	---	0
1169	erythromycin	-0.047	5	0.265	---	---	20
1170	dexpropranolol	-0.16	3	-0.264	---	---	33
1171	tetracycline	0.054	5	0.264	---	---	20
1172	tetraethylenepentamin	-0.09	6	0.264	---	---	16
1173	SC-19220	-0.132	4	-0.263	---	---	25
1174	lomefloxacin	0.011	6	0.263	---	---	16
1175	Prestwick-1085	-0.15	4	-0.263	---	---	25
1176	paroxetine	-0.109	4	-0.262	---	---	25
1177	apramycin	-0.145	4	0.262	---	---	0
1178	mexiletine	-0.081	6	0.261	---	---	0
1179	dizocilpine	-0.125	5	-0.261	---	---	20
1180	iopanoic acid	-0.078	4	0.26	---	---	25
1181	velnacrine	-0.028	4	0.26	---	---	25
1182	kinetin	-0.102	4	0.259	---	---	0
1183	ribostamycin	-0.148	4	0.259	---	---	0
1184	mafenide	0.081	5	0.259	---	---	40
1185	miconazole	-0.231	5	0.259	---	---	0
1186	CAY-10397	-0.064	3	-0.259	---	---	33
1187	imipramine	-0.113	4	-0.257	---	---	25
1188	etodolac	-0.138	5	0.257	---	---	0
1189	ketotifen	0.04	4	-0.256	---	---	25
1190	sulfachlorpyridazine	-0.146	5	-0.255	---	---	40
1191	alfadolone	-0.005	3	0.253	---	---	33
1192	tribenoside	-0.103	4	0.253	---	---	0
1193	CP-863187	-0.119	4	-0.251	---	---	25
1194	alimemazine	-0.095	4	0.251	---	---	25
1195	cefixime	-0.143	4	-0.251	---	---	25
1196	furosemide	0.004	4	-0.251	---	---	25
1197	diazoxide	-0.003	5	0.25	---	---	20
1198	butacaine	0.041	4	0.25	---	---	25
1199	salsolidin	-0.157	4	-0.25	---	---	25
1200	domperidone	0.105	4	0.25	---	---	25
1201	nifurtimox	-0.144	4	0.249	---	---	0
1202	levcycloserine	-0.185	4	0.249	---	---	0
1203	phenacetin	-0.163	4	0.248	---	---	0
1204	N-acetylmuramic acid	-0.016	4	-0.247	---	---	25
1205	droperidol	0.009	4	0.247	---	---	25
1206	tubocurarine chloride	-0.169	4	0.247	---	---	0
1207	minaprine	-0.063	5	0.247	---	---	20
1208	dexpanthenol	-0.161	4	0.247	---	---	0
1209	diflorasone	-0.141	4	0.246	---	---	0
1210	cinchocaine	-0.069	5	0.246	---	---	20
1211	methylbenzethonium c	-0.04	6	0.246	---	---	16
1212	chlorhexidine	-0.076	5	0.246	---	---	20
1213	succinylsulfathiazole	-0.104	4	-0.246	---	---	25
1214	lycorine	0.035	5	0.245	---	---	40
1215	todralazine	-0.078	5	0.245	---	---	20

1216	natamycin	-0.203	4	0.245	---	---	0
1217	sulfamerazine	0.082	5	0.244	---	---	40
1218	dihydroergotamine	-0.122	5	-0.244	---	---	20
1219	oxolinic acid	-0.2	5	-0.244	---	---	40
1220	vinburnine	-0.041	4	0.243	---	---	25
1221	methanthelinium bromi	-0.017	4	0.242	---	---	25
1222	N6-methyladenosine	-0.058	4	-0.242	---	---	25
1223	fenspiride	-0.151	5	-0.241	---	---	40
1224	metolazone	-0.106	5	-0.241	---	---	40
1225	nilutamide	0.019	4	0.24	---	---	25
1226	promethazine	-0.183	4	-0.24	---	---	25
1227	neomycin	0.025	5	0.24	---	---	20
1228	terguride	-0.045	8	0.239	---	---	25
1229	indometacin	-0.081	8	-0.239	---	---	37
1230	(+)-isoprenaline	-0.135	4	0.239	---	---	0
1231	zaprinast	0.034	4	0.239	---	---	25
1232	pridinol	-0.107	4	0.238	---	---	0
1233	corynanthine	-0.098	3	0.238	---	---	33
1234	tracazolate	-0.143	4	-0.238	---	---	25
1235	disopyramide	0.054	4	0.238	---	---	25
1236	iobenguane	-0.125	4	-0.236	---	---	25
1237	propidium iodide	-0.076	4	0.236	---	---	25
1238	methotrexate	0.015	8	0.236	---	---	25
1239	phentolamine	0.049	7	0.236	---	---	28
1240	zidovudine	0.025	4	0.235	---	---	25
1241	lobeline	-0.066	4	-0.234	---	---	25
1242	thioproperazine	-0.083	5	-0.233	---	---	40
1243	riluzole	-0.121	5	0.233	---	---	0
1244	butamben	0.053	4	0.232	---	---	25
1245	carbamazepine	-0.087	8	0.232	---	---	12
1246	sulfathiazole	-0.117	5	-0.232	---	---	40
1247	oxamniquine	0.052	4	0.23	---	---	25
1248	physostigmine	-0.001	4	0.23	---	---	25
1249	nitrendipine	-0.111	5	0.23	---	---	20
1250	oxaprozin	-0.141	6	0.228	---	---	16
1251	octopamine	-0.008	4	0.228	---	---	25
1252	estradiol	0.124	37	0.227	---	---	35
1253	ciclosporin	0.057	6	-0.227	---	---	33
1254	harmaline	-0.013	4	-0.226	---	---	25
1255	tocainide	-0.01	4	-0.226	---	---	25
1256	mesalazine	0.052	5	0.225	---	---	20
1257	PF-01378883-00	-0.012	4	0.225	---	---	25
1258	prasterone	-0.029	4	0.223	---	---	25
1259	tenoxicam	-0.063	4	-0.223	---	---	25
1260	wortmannin	-0.16	18	-0.222	---	---	38
1261	AG-013608	-0.068	8	0.222	---	---	12
1262	clioquinol	-0.093	5	-0.222	---	---	20
1263	bephenium hydroxyna	-0.12	5	0.222	---	---	20
1264	pentamidine	-0.146	5	0.221	---	---	0
1265	(-)-isoprenaline	-0.156	4	0.22	---	---	0
1266	sulfanilamide	-0.081	4	-0.22	---	---	25
1267	acacetin	-0.034	6	0.22	---	---	16
1268	ritodrine	-0.123	4	-0.22	---	---	25
1269	allantoin	-0.06	5	0.22	---	---	20
1270	securinine	-0.075	4	-0.219	---	---	25
1271	antimycin A	-0.199	5	0.217	---	---	0
1272	naloxone	-0.166	6	0.217	---	---	0
1273	apomorphine	-0.221	5	0.217	---	---	0
1274	morantel	-0.194	5	-0.215	---	---	40
1275	genistein	0.074	17	0.212	---	---	29
1276	fulvestrant	-0.23	40	-0.21	---	---	47

1277	mianserin	-0.194	5	-0.209	---	---	40
1278	tetrandrine	0.017	4	-0.209	---	---	25
1279	Chicago Sky Blue 6B	-0.029	4	0.208	---	---	25
1280	epivincamine	0.017	4	0.207	---	---	25
1281	0317956-0000	-0.009	8	-0.206	---	---	37
1282	pentetic acid	0.017	5	-0.205	---	---	20
1283	prednisolone	-0.058	5	-0.205	---	---	40
1284	clozapine	-0.069	17	-0.202	---	---	35
1285	alexidine	-0.144	4	-0.2	---	---	25
1286	ascorbic acid	0.002	4	0.199	---	---	25
1287	tiabendazole	-0.117	4	0.198	---	---	0
1288	lynestrenol	-0.048	5	0.197	---	---	20
1289	nifedipine	-0.044	7	0.197	---	---	14
1290	conessine	-0.002	4	0.196	---	---	25
1291	dexibuprofen	-0.03	4	0.195	---	---	25
1292	tamoxifen	-0.088	7	0.194	---	---	14
1293	chlortetracycline	-0.153	5	-0.193	---	---	40
1294	deferoxamine	-0.137	8	0.193	---	---	12
1295	meclocycline	-0.019	4	0.192	---	---	25
1296	mephenesin	-0.116	5	0.186	---	---	20
1297	haloperidol	-0.066	32	-0.185	---	---	31
1298	acebutolol	-0.079	5	0.183	---	---	20
1299	mebeverine	-0.007	4	-0.182	---	---	25
1300	mefenamic acid	-0.042	5	0.181	---	---	20
1301	paclitaxel	-0.098	6	0.18	---	---	16
1302	dantrolene	-0.124	6	-0.178	---	---	33
1303	orphenadrine	-0.064	6	0.175	---	---	16
1304	chlorpromazine	-0.202	19	-0.17	---	---	42
1305	monorden	-0.105	22	-0.168	---	---	40
1306	0179445-0000	-0.123	8	-0.166	---	---	25
1307	glipizide	-0.068	5	0.16	---	---	20
1308	diphemanil metilsulfate	-0.058	5	0.151	---	---	20
1309	PF-00562151-00	-0.013	8	0.139	---	---	25
