# Supplementary material for: Genomic and immunological profiles of small-cell lung cancer between East Asians and Caucasian
Source: Cancer Cell Int. 2022 Apr 29;22:173. doi: 10.1186/s12935-022-02588-w (PMC9052616; doi:10.1186/s12935-022-02588-w)
Supplement: Supplementary file 9 — Additional file 9: Figure S9. Heatmap depicting the correlation rho between the proportion of immune cells and mutations in each DDR pathway and TMB in the East Asian (a) and Caucasian (b) cohorts. [file 12935_2022_2588_MOESM9_ESM.pdf]

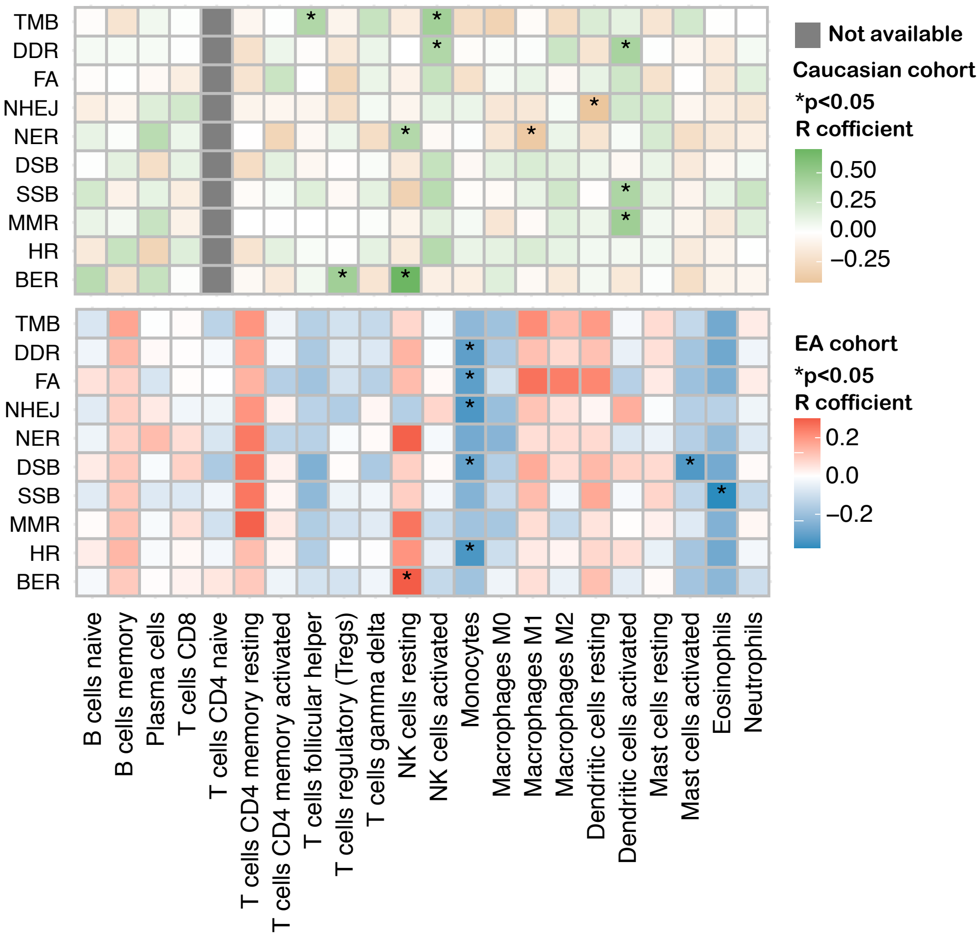

TMB
DDR
FA
NHEJ
NER
DSB
SSB
MMR
HR
BER
Not available
Caucasian cohort
*p<0.05
R cofficient
0.50
0.25
0.00
−0.25
EA cohort
*p<0.05
R cofficient
0.2
0.0
−0.2
B cells naive
B cells memory
Plasma cells
T cells CD8
T cells CD4 naive
T cells CD4 memory resting
T cells CD4 memory activated
T cells follicular helper
T cells regulatory (Tregs)
T cells gamma delta
NK cells resting
NK cells activated
Monocytes
Macrophages M0
Macrophages M1
Macrophages M2
Dendritic cells resting
Dendritic cells activated
Mast cells resting
Mast cells activated
Eosinophils
Neutrophils
