# Supplementary material for: Genomic and immunological profiles of small-cell lung cancer between East Asians and Caucasian
Source: Cancer Cell Int. 2022 Apr 29;22:173. doi: 10.1186/s12935-022-02588-w (PMC9052616; doi:10.1186/s12935-022-02588-w)
Supplement: Supplementary file 16 — Additional file 16: Supplemental methods. [file 12935_2022_2588_MOESM16_ESM.pdf]

Supplementary methods

EA SCLC patient tissue processing, gene sequencing and data processing

① DNA extractions and WES

DNA was extracted from each formalin-fixed and paraffin-embedded (FFPE) specimen (n = 83) and matched germline specimen (n = 83) using the Gentra Puregene DNA Extraction Kit (Qiagen) following the protocol provided by the manufacturer. WES was performed with DNA extracted from FFPE specimens and matched normal samples. Short insert DNA libraries were prepared with the TruSeq DNA PCR-free Samples Preparation Kit (Illumina) for paired-end sequencing at a minimum read length of 2 x 100 bp. Human DNA libraries were sequenced with the aim of obtaining an average coverage of 300X for both tumor and matched normal tissues.

② RNA extractions and RNA sequencing

For RNA extractions, tissue sections were first lysed and homogenized with the TissueLyser (Qiagen). Subsequent RNA extractions were performed with the Qiagen RNeasy Mini Kit according to the instructions provided by the manufacturer. The RNA quality was assessed with a Bioanalyzer 2100 DNA Chip 7500 (Agilent Technologies), and samples with an RNA integrity number (RIN) of over 7 were further analyzed by RNA-seq. All sequencing reactions were performed on an Illumina HiSeq 2000 instrument (Illumina, San Diego, CA, USA). RNA-seq was performed with RNA extracted from FFPE samples (n=59). cDNA libraries were prepared from poly(A)-selected RNA by applying the Illumina TruSeq protocol for mRNA. The

libraries were then sequenced with a 2 x 100 bp paired-end protocol.

③ Data processing

The raw sequencing reads of human samples acquired from WES were aligned to the reference human genome (NCBI38/hg38). All sequence data were analyzed for read counts, quality values, GC content, and all other relevant parameters with FastQC (v0.10.1). The alignment was performed with the Burrows-Wheeler Aligner (BWA, version 0.6.1-r104)(1). Concordant read pairs were identified as potential PCR duplicates and were subsequently masked in the alignment file. Additionally, an estimation of human DNA library contamination was implemented to enhance the sensitivity and specificity of mutation calling. SAMtools mpileup (0.1.19)(2) was used to locate nonreference positions in tumor and germline samples. After removal of terminal adaptor sequences and low-quality data, reads were mapped to hg38 and aligned using BWA (version 0.6.1-r104). MuTect2 (3.4–46-gbc02625)(3) was employed to call somatic small indels and SNVs. We used HISAT2 (version 2.1.0)(4) with the default setting to map the RNA-seq data to the human reference genome (NCBI38/hg38). We aggregated the read counts at the gene level using HTSeq(5).

Reference

1. Li H, Durbin R. Fast and accurate short read alignment with Burrows-Wheeler transform. *Bioinformatics* (2009) **25**:1754–1760. doi:10.1093/bioinformatics/btp324

2. Li H, Handsaker B, Wysoker A, Fennell T, Ruan J, Homer N, Marth G, Abecasis G, Durbin R. The Sequence Alignment/Map format and SAMtools. *Bioinformatics (Oxford, England)* (2009) **25**:2078–2079. doi:10.1093/bioinformatics/btp352

3. Cibulskis K, Lawrence MS, Carter SL, Sivachenko A, Jaffe D, Sougnez C, Gabriel S, Meyerson M, Lander ES, Getz G. Sensitive detection of somatic point mutations in impure and heterogeneous cancer samples. *Nature Biotechnology* (2013) **31**:213–219. doi:10.1038/nbt.2514

4. Kim D, Langmead B, Salzberg SL. HISAT: a fast spliced aligner with low memory requirements. *Nature methods* (2015) **12**:357–360. doi:10.1038/nmeth.3317

5. Anders S, Pyl PT, Huber W. HTSeq--a Python framework to work with high-throughput sequencing data. *Bioinformatics (Oxford, England)* (2015) **31**:166–169. doi:10.1093/bioinformatics/btu638
